# Efficacy and safety of six Chinese patent medicines for elderly functional constipation: a network meta-analysis

**DOI:** 10.3389/fmed.2026.1728217

**Published:** 2026-03-31

**Authors:** Xinyu Wang, Min Wang, Guishan Liu, Riming Zhong, Lei Liu, Qiuming Chen, Rui Li

**Affiliations:** 1 Shantou Hospital of Traditional Chinese Medicine, Affiliated to Guangzhou University of Chinese Medicine, Shantou, China; 2 Affiliated Hospital of Nanjing University of Chinese Medicine, Nanjing, China

**Keywords:** Chinese patent medicine, complementary therapy, elderly, functional constipation, network meta-analysis, randomized controlled trial

## Abstract

**Background:**

Functional constipation (FC) is a prevalent condition in the elderly that greatly reduces quality of life. Combined treatment using Chinese Patent Medicine (CPM) and Western Medicine (WM) is frequently employed; however, the comparative efficacy and safety of various CPMs are unclear.

**Objective:**

To assess and compare the efficacy and safety of six regularly used CPMs combined with WM in the treatment of elderly FC patients.

**Methods:**

A systematic search was performed in eight electronic databases from their inception through May 31, 2025. Two reviewers independently evaluated studies, gathered data, and calculated the risk of bias using the Risk of Bias 2 tool. The level of evidence certainty was rated using GRADE. The Bayesian network meta-analysis (NMA) was conducted using R software (version 4.5.0). The effect sizes were shown as relative risk, mean difference, standardized mean difference, and 95% credible intervals (CrIs). Interventions were ranked according to the surface under the cumulative ranking curve (SUCRA).

**Results:**

Twenty-three randomized controlled trials (2,065 participants) involving six CPMs were included. The certainty of evidence for all outcomes was rated as low or very low, and an unclear or high risk of bias, which may have overestimated treatment effects. CPMs plus WM generally improved overall response compared with WM alone. SUCRA rankings suggested that Congrong Tongbian Oral Liquid + WM and Liuwei Anxiao Capsule + WM had higher probabilities of being among the top options for overall response; however, several comparisons were informed by few trials, and the evidence was of low/very low certainty. Therefore, SUCRA-based rankings should be considered exploratory and hypothesis-generating rather than definitive indicators of clinical superiority. Adverse-event reporting was inconsistent and mostly involved mild reactions (e.g., abdominal pain, diarrhea, bloating); however, definitions and ascertainment were often not described and participant-level incidence was frequently unavailable, limiting comparative safety conclusions, and the absence of reported serious adverse events should not be interpreted as evidence that serious events did not occur.

**Conclusion:**

CPMs plus WM may offer potential benefits for elderly patients with FC. However, given the low/very low certainty of evidence, prevalent risk-of-bias concerns, and limitations in safety reporting, definitive comparative conclusions or practice recommendations cannot currently be made. These findings should be regarded as exploratory signals requiring confirmation in adequately powered, rigorously designed multicenter randomized controlled trials with stringent adverse-event monitoring and extended follow-up. Accordingly, no strong clinical recommendations favoring any specific CPM can be made based on current evidence.

**Systematic review registration:**

https://www.crd.york.ac.uk/PROSPERO/view/CRD420251087886, identifier CRD420251087886.

## 1 Introduction

Functional Constipation (FC), also referred to as chronic idiopathic constipation, is a subtype of chronic constipation and is primarily characterized by difficulty in defecation, incomplete evacuation, and reduced defecation frequency (1, 2). Unlike organic constipation and drug-induced constipation, FC is not caused by identifiable organ damage or adverse drug reactions, yet it exhibits the highest prevalence among the three types (3). According to epidemiological data, the global prevalence of FC in individuals over the age of 60 is 33.5%, with the proportion increasing progressively with age (4). As the global transition into an aging society accelerates and expectations for quality of life continue to rise, FC has been shown to significantly impair the quality of life of elderly patients. In elderly patients, abdominal distension, abdominal pain, and decreased appetite are commonly observed. Additionally, associations have been identified with sleep disorders, anxiety, depression, and social avoidance, which are among the psychological and behavioral symptoms (5). These conditions further intensify the family care burden and medical expenses, inevitably worsening the economic burden on the healthcare system (6). The pathophysiology of FC is complex and multifactorial. Contributing factors include genetics, lifestyle, diet, dysfunction of the enteric nervous system (ENS), and alterations in neurotransmitters. These mechanisms may result in insufficient colonic propulsion, reduced rectal sensitivity, and defecation disorders (1). Currently, Western medical treatment for FC primarily involves the use of bulk-forming laxatives and osmotic laxatives, such as lactulose and polyethylene glycol (1). Stimulant laxatives are now less frequently used as first-line treatment due to the common occurrence of adverse reactions such as drug dependence and electrolyte imbalance (7). Although bulk-forming laxatives and osmotic laxatives are recommended as first-line options in clinical guidelines, their therapeutic effectiveness remains uncertain for some patients. In addition, these agents may also cause potential adverse reactions, including abdominal distension, esophageal obstruction, and colonic obstruction (8). Newer laxatives, including linaclotide, prucalopride, and lubiprostone, exhibit more effective bowel-stimulating effects compared to traditional agents. However, their use may be limited in certain patients due to the frequent occurrence of adverse reactions such as diarrhea, abdominal distension, nausea, and headache, as well as the relatively high cost of these medications (8, 9). In elderly patients, the risk of adverse drug reactions is further increased due to reduced hepatic first-pass metabolism and renal clearance, highlighting the urgent need for safer and more effective prevention and treatment strategies. Chinese patent medicines (CPMs) offer distinct advantages in managing FC in the elderly, as they are grounded in the principle of holistic regulation and aim to achieve treating both symptoms and root causes through multi-target actions—such as enhancing intestinal motility, modulating intestinal secretion, and improving intestinal microbiota (10). In addition, they are easy to administer, cost-effective, and generally more acceptable to older patients. However, the current evidence supporting the efficacy of CPMs remains significantly limited. Challenges include variability in the quality of related randomized controlled trials (RCTs), inadequate safety reporting, and a lack of comprehensive high-quality evidence synthesis, all of which hinder the identification of optimal clinical treatment strategies. A network meta-analysis (NMA) will be conducted to evaluate the effectiveness and safety of six commonly used CPMs for the treatment of elderly FC. These include Maren-formula CPMs (Maren Wan, Maziren Wan, Maren Runchang Wan, Maren Soft Capsules, and Maren Capsules), Qirong Runchang Oral Liquid, Liuwei Anxiao Capsule, Congrong Tongbian Oral Liquid, Simo Decoction Oral Liquid, and Shouhui Tongbian Capsule. This study aims to compare CPMs combined with WM using a Bayesian NMA and to provide exploratory comparative signals rather than definitive evidence of superiority, given expected limitations in the underlying trial evidence.

## 2 Materials and methods

### 2.1 Study design

This systematic review was prospectively registered in the International Prospective Register of Systematic Reviews (PROSPERO; Registration No. CRD420251087886), in accordance with established protocols to ensure methodological transparency and reproducibility.

### 2.2 Search strategy

In accordance with the guidelines of the *Cochrane Systematic Review Manual*, the search strategy was jointly developed by two researchers. Relevant literature was retrieved from the inception of each database up to May 31, 2025, including China National Knowledge Infrastructure (CNKI), Chinese Biomedical Literature Database (Sinomed), VIP Database (VIP), Wanfang Data, PubMed, Embase, The Cochrane Library, and Web of Science. Tailored search strategies were formulated based on the specific retrieval rules of each database. Specific search terms included aged, elderly, older adults, constipation, functional constipation, chronic constipation, drug therapy, drugs, Chinese herbal medicine, Chinese patent medicine, proprietary Chinese medicine, and herbal formulas, as well as Maren Soft Capsule, Ma Ren Wan, Maren Runchang Pill, Liuwei Anxiao Capsule, Qirong Runchang Oral Liquid, Congrong Tongbian Oral Liquid, Simo Decoction Oral Liquid, and Shouhui Tongbian Capsule, along with their relevant synonyms.

### 2.3 Inclusion and exclusion criteria

#### 2.3.1 Inclusion criteria

Studies were included only if they met all of the following criteria: (1) Study type: RCTs; studies published in Chinese or English were included; (2) Study subjects: Participants were required to be aged 60 years or older, with no restrictions on gender, underlying conditions, or constipation syndrome classification. Eligibility was determined based on the diagnostic criteria of FC Rome III, FC Rome IV, the Chinese Chronic Constipation Expert Consensus (2019), the “Diagnosis and Treatment Standards for Elderly Functional Constipation Patients” issued by the Chinese Medical Association, or other widely accepted diagnostic standards. (3) Interventions: The control group was treated with standard western pharmacotherapy, with or without the addition of a placebo, in accordance with relevant clinical medication guidelines. The experimental group received CPMs in combination with conventional Western drug therapy. The Chinese patent medicines included Maren-formula CPMs, Qirong Runchang Oral Liquid, Liuwei Anxiao Capsule, Congrong Tongbian Oral Liquid, Simo Decoction Oral Liquid, and Shouhui Tongbian Capsule.

#### 2.3.2 Exclusion criteria

Studies were excluded for any of the following reasons: (1) Literature with incomplete, inaccurate, or duplicate data, or for which the original data were unavailable; (2) Literature in which the diagnosis of FC does not adhere to recognized diagnostic criteria; (3) Literature containing erroneous or missing data; (4) Literature types including dissertations, conference proceedings, reviews, meta-analyses, animal studies, case reports, and academic monographs; (5) Literature in which the outcome measures are inconsistent with the design of this study; (6) Literature involving non-pharmacological interventions, acupoint application, traditional Chinese medicine injections, combined use of multiple CPMs, or lacking the use of CPM as an intervention.

### 2.4 Literature screening and data extraction

Two reviewers independently screened records and extracted data using a standardized form for subsequent Bayesian network meta-analysis (NMA). Disagreements were resolved through discussion, with consultation of a third reviewer when consensus could not be reached.

The extracted outcomes included: (1) overall clinical effective rate, as reported in each trial; (2) recurrence rate; (3) functional constipation (FC) symptom scores (difficulty in defecation, abdominal discomfort, stool consistency, and defecation frequency); and (4) serum gastrointestinal hormones, including gastrin (GAS), motilin (MTL), and substance P (SP).

Because definitions of overall clinical effectiveness varied across studies and were often insufficiently described, comparability may be limited. This outcome was therefore interpreted cautiously, and trial-level definitions are summarized in Supplementary Table S1. A restriction-based sensitivity analysis limited to trials using strictly comparable definitions was not feasible due to incomplete reporting and the risk of compromising network connectivity.

For FC symptom outcomes, scoring instruments and anchors were heterogeneous and sometimes incompletely reported. Accordingly, these outcomes were synthesized using standardized mean differences (SMDs) and interpreted cautiously; trial-level definitions are provided in Supplementary Table S2. Restriction-based sensitivity analyses were not feasible for similar reasons.

Handling of multi-arm trials: In three-arm trials including CPM + WM, WM alone, and CPM alone, we extracted only the CPM + WM versus WM comparison to align with our add-on research question. The CPM-alone arm was not incorporated into the network. Consequently, each multi-arm trial contributed a single contrast to the NMA, and adjustment for within-trial correlation (e.g., shared-control splitting or covariance modeling) was not required.

### 2.5 Safety data extraction and synthesis

Safety information was collected from each trial as reported, focusing on adverse events (AEs), including AE type/description and event counts. Where explicitly stated, we recorded whether AE definitions were prespecified, how AEs were ascertained (e.g., active surveillance vs. spontaneous reporting), and participant-level AE incidence (number of participants experiencing ≥ 1 AE). Due to heterogeneity and incomplete reporting in AE definitions and ascertainment procedures, together with the frequent lack of participant-level AE incidence, safety data were not considered sufficiently comparable for quantitative synthesis. Accordingly, safety outcomes were summarized descriptively rather than pooled. Where feasible, we additionally reported episode-based AE rates and calculated episode-based RRs with 95% confidence intervals for CPM + WM versus WM comparisons; these estimates were presented descriptively and were not pooled.

### 2.6 Risk of bias assessment

The Risk of Bias 2 (RoB2) tool developed by the Cochrane Collaboration was employed to assess the risk of bias in the included studies (11). The assessment covered the following domains: 1. bias arising from the randomization process; 2. bias due to deviations from intended interventions; 3. bias in the measurement of outcomes; 4. bias due to missing outcome data; 5. bias in the selection of the reported results; 6. overall bias. Each study was evaluated based on these domains and classified as having a high, unclear, or low risk of bias. Grading of Recommendations Assessment, Development and Evaluation (GRADE) was used to assess the quality of evidence. All evaluations and result summaries were independently conducted by two researchers. Any disagreements were resolved through discussion with the full research team.

### 2.7 Statistical analysis

The NMA was conducted within a Bayesian framework in R (version 4.5.0) using the gemtc package, applying a random-effects consistency model. For dichotomous outcomes, we specified a binomial likelihood with a log link to estimate risk ratios (RRs) or a logit link to estimate odds ratios (ORs). For continuous outcomes, we used a normal likelihood with an identity link, summarizing treatment effects as mean differences (MDs) or SMDs, as appropriate. Priors were set to the default non-informative priors in the gemtc package. MCMC sampling was run using 4 chains with 20,000 adaptation (burn-in) iterations and 50,000 sampling iterations per chain (thinning = 1). Convergence was evaluated using trace/density plots and Brooks-Gelman-Rubin diagnostics; corresponding plots are provided in Supplementary Figures S1–S4. For outcomes where lower values indicate benefit (e.g., recurrence and symptom scores), ranking direction was set accordingly. Because the network was sparse and no direct comparison included ≥ 10 studies, formal assessment of small-study effects/publication bias (e.g., funnel plots) was not performed. As the network contained no closed loops, statistical assessment of inconsistency between direct and indirect evidence was not feasible; therefore, indirect estimates relied primarily on the transitivity assumption. Robustness of the primary outcome was examined using leave-one-out sensitivity analyses. For figure presentation, the “+” symbol in some intervention names was replaced with an underscore “_” in figures due to gemtc limitations, and forest-plot labels for the four FC symptom indicators were displayed as SMD.

### 2.8 Qualitative assessment of transitivity

Transitivity is essential for the validity of indirect comparisons in NMA. Because the evidence network had an open structure (i.e., no closed loops), formal inconsistency testing was not feasible; therefore, we conducted a qualitative assessment of transitivity based on clinical and methodological characteristics of included trials. Prespecified potential effect modifiers were compared across treatment nodes, including diagnostic criteria, age distribution, baseline constipation duration (years), treatment duration (weeks), outcome domains and assessment timepoints (including recurrence follow-up windows), and the type/regimen of WM comparators. We also considered heterogeneity in the operational definitions/measurement instruments for key outcomes, particularly the investigator-defined overall clinical effective rate and non-uniform symptom scoring instruments and criteria across trials (including defecation difficulty, abdominal discomfort, stool consistency, and defecation frequency scores). Variability in CPM formulations and dosing/administration was additionally considered based on Supplementary Table S3 describing components, indications, contraindications, and precautions of the CPMs. Given incomplete reporting of key modifiers and outcome definitions in multiple trials, together with sparse evidence for some nodes, quantitative adjustment (e.g., network meta-regression) was not performed. Accordingly, indirect comparisons and SUCRA-based rankings were interpreted cautiously as exploratory and hypothesis-generating.

## 3 Results

### 3.1 Literature search results

**FIGURE 1:**
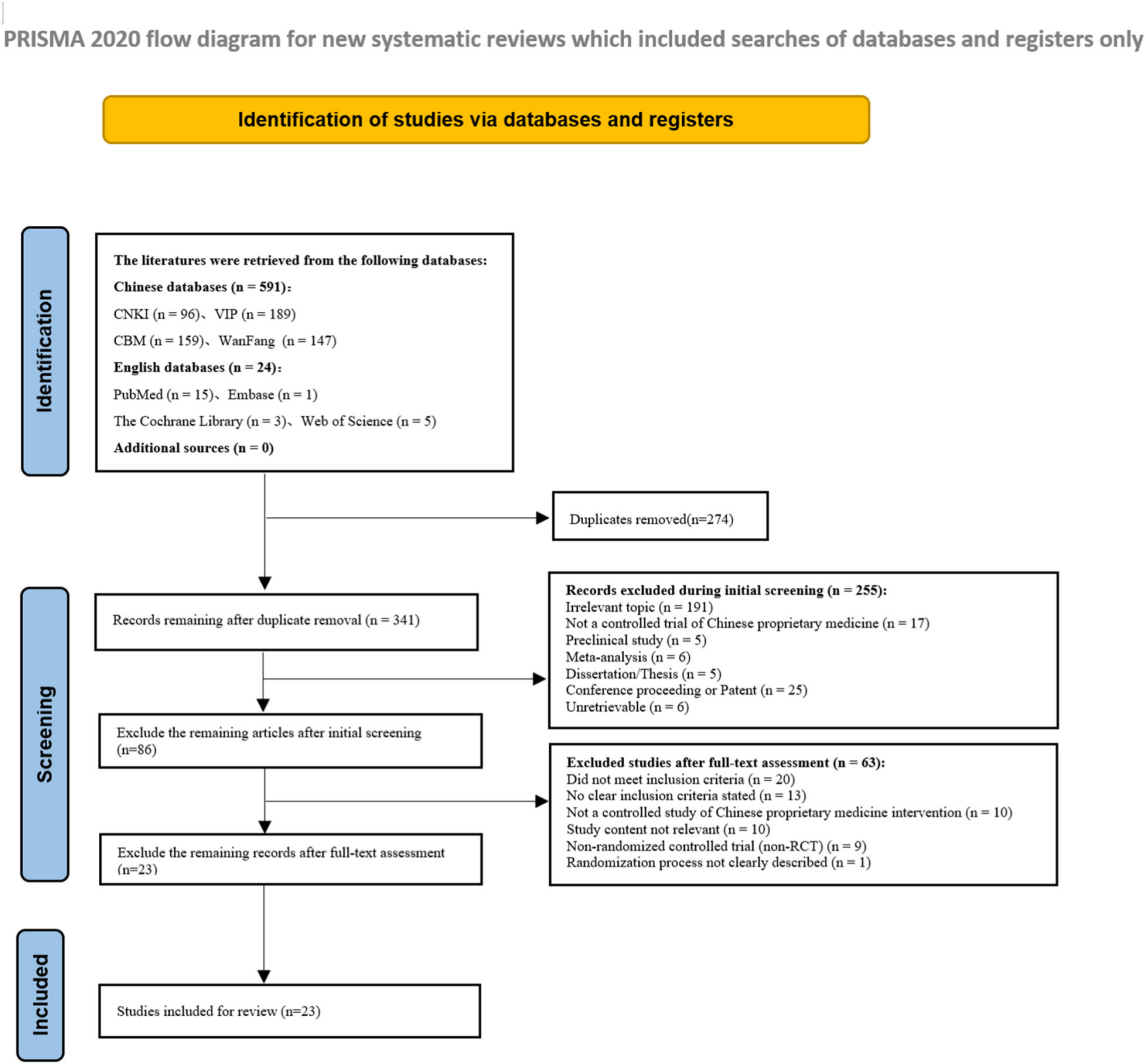
PRISMA flow diagram for literature screening of included studies.

A total of 615 articles were initially identified through a preliminary search, including 591 in Chinese and 24 in English. After removing 274 duplicates, an initial screening based on titles and abstracts was conducted. As a result, 255 articles were excluded, including case reports, experimental studies (such as animal and cell experiments), reviews, systematic evaluations and meta-analyses, conference proceedings, patents, dissertations, and studies that were inaccessible. A secondary screening was then performed through full-text review, leading to the exclusion of 63 articles that were non-RCTs, did not meet the inclusion or exclusion criteria, or did not clearly indicate whether randomization was applied. In total, 592 articles were excluded. Ultimately, 23 articles were included. The detailed literature screening process is illustrated in Figure 1.

### 3.2 Characteristics of included studies

A total of 23 RCTs (12–34) were included, comprising 1,029 participants in the control group and 1,036 in the experimental group. All studies reported clearly defined diagnostic criteria. Specifically, seven studies (20, 22, 24, 25, 27, 30, 34) employed the FC Rome IV diagnostic criteria; 13 studies (13–19, 21, 28, 29, 31–33) used the FC Rome III criteria; one study (12) followed the 2019 expert consensus on Chinese chronic constipation issued by the Functional Gastrointestinal Disorders Group of the Chinese Society of Gastroenterology; and two studies (23, 26) adopted the “Diagnostic and Treatment Standards for Elderly Functional Constipation Patients” proposed by the Chinese Medical Association. The included literature involved six types of CPMs: Maren-formula CPMs + WM in seven studies (12–18); Qirong Runchang Oral Liquid + WM in three studies (30–32); Liuwei Anxiao Capsule + WM in three studies (27–29); Congrong Tongbian oral liquid + WM in two studies (33, 34); Simo Decoction Oral Liquid + WM in three studies (19–21); and Shouhui Tongbian Capsule + WM in five studies (22–26). A total of 10 types of Western medicine pharmacotherapy were included in the control group, comprising Bifidobacterium triple viable capsules, compound Lactobacillus acidophilus, (succinate) prucalopride, itopride, cisapride, (citrate) mosapride, polyethylene glycol electrolyte powder, bisacodyl enteric-coated tablets, glycerin enema, calcium polycarbophil capsules, and lactulose. In all included studies, the intervention was compared in the form of CPM + WM versus WM alone. The basic characteristics of the included studies are presented in Table 1.

**TABLE 1:** Clinical and demographic characteristics of studies included in NMA.

First author	Number of cases		Age (years)		Disease duration (years)		Intervention Details		Recurrence assessment timepoint (weeks after end of treatment)	Outcome indicators
	Experimental group	Control group	Experimental group	Control group	Experimental group	Control group	Experimental group	Control group		
Zhu et al. (12)	37	36	59.82 ± 5.60	59.78 ± 5.55	7.01 ± 1.65	7.15 ± 1.69	MR, 6 g, bid BTVC, 0.42 g, bid Course = 4 weeks	BTVC, 0.42 g, bid Course = 4 weeks	12	①⑥⑦⑧
Liu et al. (13)	49	49	64.47 ± 1.35	64.24 ± 1.15	3.36 ± 1.14	3.12 ± 1.01	MR, 9 g, bid PRU-SUC, 1–R mg, qd (age-adjusted) Course = 1 weeks	PRU-SUC, 1UC mg, qd (age-adjusted) Course = 1 weeks	NR	①②③④⑤⑧
Chen and Li (14)	64	63	71.97 ± 5.66	70.45 ± 4.92	NR	NR	MR, 9 g, bid LAC, 5 g, tid Course = 5 weeks	LAC, 5 g, tid Course = 5 weeks	24	①
Li and Ouyang (15)	30	30	66.93 ± 5.41	66.8 ± 4.7	5.57 ± 3.84	5.63 ± 3.97	MR, 9 g, qd PEG-ELS, 13.7 g, bid Course = 2 weeks	PEG-ELS, 13.7 g, bid Course = 2 weeks	2	①
Zhang (16)	30	30	70.2 ± 2.8	71.8 ± 2.5	NR	NR	MR, 1.8 g, tid CIS, 10 mg, tid Course = 3 weeks	CIS, 10 mg, tid Course = 3 weeks	NR	①
Li et al. (17)	32	32	76±5	75±4	NR	NR	MR, 1.05 g, tid CIS, 10 mg, tid Course = 2 weeks	CIS, 10 mg, tid Course = 2 weeks	NR	①
Guan and Zu (18)	28	26	NR	NR	NR	NR	MR, 12 g, bid ITO, 50 mg, tid Course = 2 weeks	ITO, 50 mg, tid Course = 2 weeks	NR	①
Mo (19)	34	34	73.92 ± 5.78	74.04 ± 5.91	5.75 ± 1.21	5.72 ± 1.18	SMT, 20 mg, tid CLA, 1 g, tid Course = 4 weeks	CLA, 1 g, tid Course = 4 weeks	NR	①②③④⑤
Wang and Gao (20)	135	134	72.62 ± 11.16	71.95 ± 10.49	2.86 ± 1.34	2.94 ± 1.38	SMT, 20 mg, tid LAC, 15–30 mg, qd Course = 4 weeks	LAC, 15–30 mg, qd Course = 4 weeks	12	①
Ma and Cao (21)	53	53	69.34 ± 10.81	69.83 ± 9.18	3.4 ± 1.3	3.7 ± 1.0	SMT, 20 mg, tid BIS-EC, 5 mg, qd Course = 4 weeks	BIS-EC, 5 mg, qd Course = 4 weeks	24	①
Gao et al. (22)	45	45	64.14 ± 6.98	63.96 ± 6.58	2.65 ± 0.42	2.61 ± 0.40	SHTB, 0.7 g, tid GLY-EN, 10 mL, qd Course = 2 weeks	GLY-EN, 10 mL, qd Course = 2 weeks	4	①
Gu (23)	45	45	59.7 ± 5.9	60.9 ± 6.0	NR	NR	SHTB, 0.7 g, tid LAC, 15 mg, bid Course = 4 weeks	LAC, 15 mg, bid Course = 4 weeks	4	①②④⑤
Tan et al. (24)	61	61	71. 81 ± 5.26	72. 69 ± 5.03	4.32 ± 1.4	4.16 ± 1.45	SHTB, 0.7 g, tid BTVC, 0.42 g, tid Course = 4 weeks	BTVC, 0.42 g,tid Course = 4 weeks	12	①②③⑥⑦⑧
Yuan et al. (25)	75	75	70.86 ± 5.16	70.29 ± 4.38	6.26 ± 1.32	6.17 ± 1.28	SHTB, 0.7 g, tid LAC, 20 mg, qd Course = 2 weeks	LAC, 20 mg, qd Course = 2 weeks	NR	①②③⑤⑦⑧
Zhou and Zhou (26)	49	59	67.55 ± 8.74	68.21 ± 9.15	2.75 ± 0.52	2.83 ± 0.56	SHTB, 0.7 g, tid LAC, 15 mg, bid Course = 4 weeks	LAC, 15 mg, bid Course = 4 weeks	4	①②④⑤
Zheng (27)	31	31	68.15 ± 3.53	67.26 ± 3.62	2.87 ± 0.41	2.57 ± 0.38	LWAX, 1.5 g, tid PRU, 2 mg, tid Course = 4 weeks	PRU, 2 mg, tid Course = 4 weeks	8	①②③④⑤⑥⑦⑧
Jiang and Dong (28)	39	38	70.68 ± 11.09	70.86 ± 10.52	2.95 ± 0.81	2.63 ± 0.48	LWAX, 1.5 g, tid PRU, 2 mg, tid Course = 4 weeks	PRU, 2 mg, tid Course = 4 weeks	8	①②③④⑤⑥⑦⑧
Zhang (29)	48	46	NR	NR	NR	NR	LWAX, 1.5 g, tid MOS-CIT, 5 mg, tid Course = 4 weeks	MOS-CIT, 5 mg, tid Course = 4 weeks	12	①
Liu et al. (30)	25	25	73.27 ± 6.42	73.44 ± 6.73	6.2 ± 2.85	6.10 ± 2.68	QRRC, 20 mg, tid PEG-ELS, 10 g, tid Course = 2 weeks	PEG-ELS, 10 g, tid Course = 2 weeks	2	①
Xu et al. (31)	30	30	72.17 ± 7.56	73.33 ± 7.3	6.10 ± 3.61	5.90 ± 3.6	QRRC, 20 mg, tid CPC, 1.2 g, tid Course = 1 week	CPC, 1.2 g, tid Course = 1 week	1	①
Shi (32)	40	40	81 ± 9	80 ± 9	NR	NR	QRRC, 20 mg, tid LAC, 30–60 mg, qd (titrate after 2 d) Course = 4 weeks	LAC, 30–60 mg, qd (titrate after 2 d) Course = 4 weeks	16	①
Gu et al. (33)	30	30	88.8 ± 6.5	88.9 ± 6.2	NR	NR	CRTB, 10 mg, bid LAC, 15 mg, bid Course = 4 weeks	LAC, 15 mg,bid Course = 4 weeks	NR	①
Fu (34)	20	20	NR	NR	NR	NR	CRTB, 10 mg, bid MOS, 5 mg, tid Course = 2 weeks	MOS, 5 mg, tid Course = 2 weeks	NR	①

1. Outcome indicators: ① overall clinical efficacy rate; ② difficulty in defecation score; ③ stool consistency score; ④ defecation frequency score; ⑤ abdominal discomfort symptom score; ⑥ GAS; ⑦ MTL; ⑧ SP. 2. NR, not reported; MR, Maren-formula CPMs (including Maren Pill/Maziren Pill/Maren Soft Capsule/Maren Capsule/Maren Runchang Pill); SM, Simo Decoction Oral Liquid; SHTB, Shouhui Tongbian Capsule; LWAX, Liuwei Anxiao Capsule; QRRC, Qirong Runchang Oral Liquid; CRTB, Congrong Tongbian Oral Liquid; BTVC, Bifidobacterium triple viable capsules; CLA, compound Lactobacillus acidophilus; PRU-SUC, prucalopride succinate; PRU, Prucalopride; ITO, itopride; CIS, cisapride; MOS-CIT, mosapride citrate; MOS, mosapride; PEG-ELS, polyethylene glycol electrolyte solution/powder; BIS-EC, bisacodyl enteric-coated tablets; GLY-EN, glycerin enema; CPC, calcium polycarbophil capsules; LAC, lactulose. 2. Dose note: Prucalopride was administered with age adjustment: 2 mg once daily for patients aged 60–65 years; 1 mg once daily for patients > 65 years, titratable to 2 mg once daily if needed. 3. Dose note: Lactulose was administered as 30 mL once daily with breakfast, and increased to 60 mL once daily if there was no obvious effect after 2 days, as reported. 4. Recurrence follow-up was calculated from the end of treatment unless otherwise stated.

### 3.3 Findings from qualitative assessment of transitivity

Based on Table 1, participants were generally older adults, with trial-level mean age ranging from approximately 60–89 years. Treatment duration was short (1–5 weeks, most commonly 2–4 weeks), indicating reasonable comparability with respect to age and treatment length. Baseline constipation duration ranged from about 2.6 to 7.2 years; however, several trials did not report this information, which may reflect differences in baseline chronicity and severity. WM comparators were heterogeneous, including prokinetics, osmotic laxatives, stimulant laxatives, bulking agents, enemas, and probiotics, and dosing regimens were inconsistently reported. Outcome assessment was also not fully uniform. Overall clinical effectiveness relied on investigator-defined response criteria with substantial variation across studies (Supplementary Table S1). Symptom outcomes were measured using different scoring scales and definitions for defecation difficulty, abdominal discomfort, stool consistency, and defecation frequency, and some studies did not report scoring details (Supplementary Table S2). In addition, recurrence, when reported, was assessed at different follow-up windows (approximately 2–24 weeks after treatment), while many trials did not report recurrence follow-up. Taken together, these clinical and methodological differences may function as effect modifiers and could challenge the transitivity assumption. Therefore, indirect comparisons and SUCRA-based rankings were interpreted cautiously and considered exploratory.

### 3.4 Quality assessment of included studies

**FIGURE 2:**
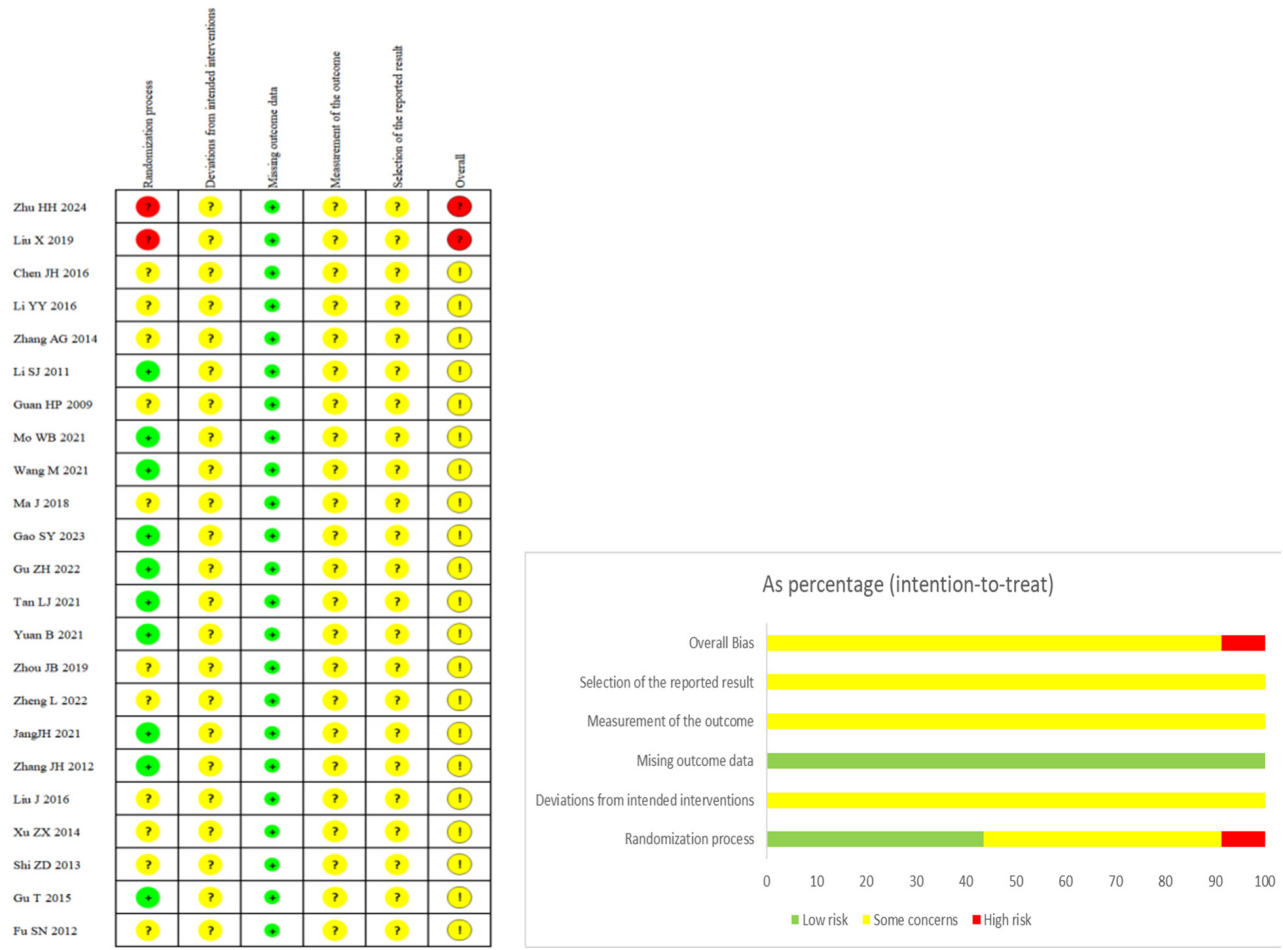
Risk of bias graph indicating the review authors’ rating regarding the risk of bias across all included studies.

1. Bias in the randomization process was assessed as follows: low risk was identified in nine studies (17, 19, 20, 22–25, 28, 33) that employed the random number table method. An unclear risk was assigned to one study (14) that referred to the “principle of randomization,” one study (27) that used “computerized random grouping,” and 10 studies (15, 16, 18, 21, 26, 29–32, 34) that mentioned “randomization” without specifying the method used. High risk was identified in one study (12) that used “different medication schemes” and another (13) that assigned groups based on odd or even hospital admission numbers. 2. Bias due to deviations from intended interventions was assessed as unclear risk in all included studies, as none reported blinding of participants or investigators, and study protocols were unavailable. 3. A low risk of bias due to missing outcome data was identified, as none of the included studies reported any missing outcome data. The risk associated with the other two types of bias remains unclear. The risk of bias for each included study is presented in Figure 2.

### 3.5 Quality of evidence

The certainty of evidence was assessed using GRADE (Table 2) and was low or very low across outcomes. A major reason was study quality: many trials provided limited information on randomization concealment and blinding, which is especially problematic for investigator-defined response criteria and symptom scores and may lead to overestimation of benefit. Confidence was also reduced because the included studies were not fully comparable, with differences in WM comparator regimens and variable outcome definitions or scoring anchors, which can contribute to variability in effect estimates. For several outcomes, the evidence base was small and the resulting intervals were wide (sometimes crossing the null), making the size of any benefit hard to judge clinically. Long-term questions were even harder to answer: treatment courses were short, and recurrence follow-up windows and assessment time points were inconsistent or not clearly reported, so durability and relapse prevention remain uncertain. Finally, selective reporting cannot be ruled out because key outcome and methodological details were often missing. Taken together, these limitations mean the SUCRA rankings should be read as exploratory, hypothesis-generating signals rather than proof of comparative superiority.

**TABLE 2:** Grade.

Certainty assessment							Effect	Quality	Importance
No of studies	Design	Risk of bias	Inconsistency	Indirectness	Imprecision	Other considerations	Effect size (95%CI)		
Overall clinical efficacy rate									
23	RCTs	Serious	No serious	No serious	No serious	Reporting bias	RR = 1.22 95%CI: 1.16; 1.27	UU○○ Low	
Recurrence rate									
11	RCTs	Serious	No serious	No serious	Serious	None	RR = 0.44 95%CI: 0.35; 0.56	UU○○ low	
Difficulty in defecation score									
8	RCTs	Serious	No serious	No serious	No serious	Reporting bias	SMD = −1.17 95%CI: −1.52; −0.83	UU○○ Low	
Abdominal discomfort symptom score									
7	RCTs	Serious	Serious	No serious	No serious	Reporting bias	SMD = −1.3 95%CI: −1.93; −0.67	UUU○ Very low	
Stool consistency score									
6	RCTs	Serious	Veryserious	No serious	Serious	Reporting bias	SMD = −1.80 95%CI: -4.41; 0.82	UUU○ Very low	
Defecation frequency score									
6	RCTs	Serious	Very serious	No serious	Serious	Reporting bias	SMD = −1.93 95%CI: −3.28; 0.57	UUU○ Very low	
GAS									
4	RCTs	serious	Very serious	No serious	Serious	Reporting bias	MD = 6.63 95%CI: 3.90; 9.37	UUU○ Very low	
MTL									
5	RCTs	Serious	Very serious	No serious	Serious	Reporting bias	MD = 34.66 95%CI: 23.59; 45.73	UUU○ Very low	
SP									
6	RCTs	Serious	Very serious	No serious	No serious	Reporting bias	MD = 9.14 95%CI: 5.81;12.47	UUU○ Very low	

## 4 Network meta-analysis

Given the sparse structure of several networks, the predominance of low/very low certainty of evidence, and prevalent risk-of-bias concerns, NMA effect estimates and SUCRA rankings are interpreted cautiously throughout this section and are presented primarily for exploratory, hypothesis-generating purposes.

### 4.1 Overall clinical efficacy rate

**FIGURE 3:**
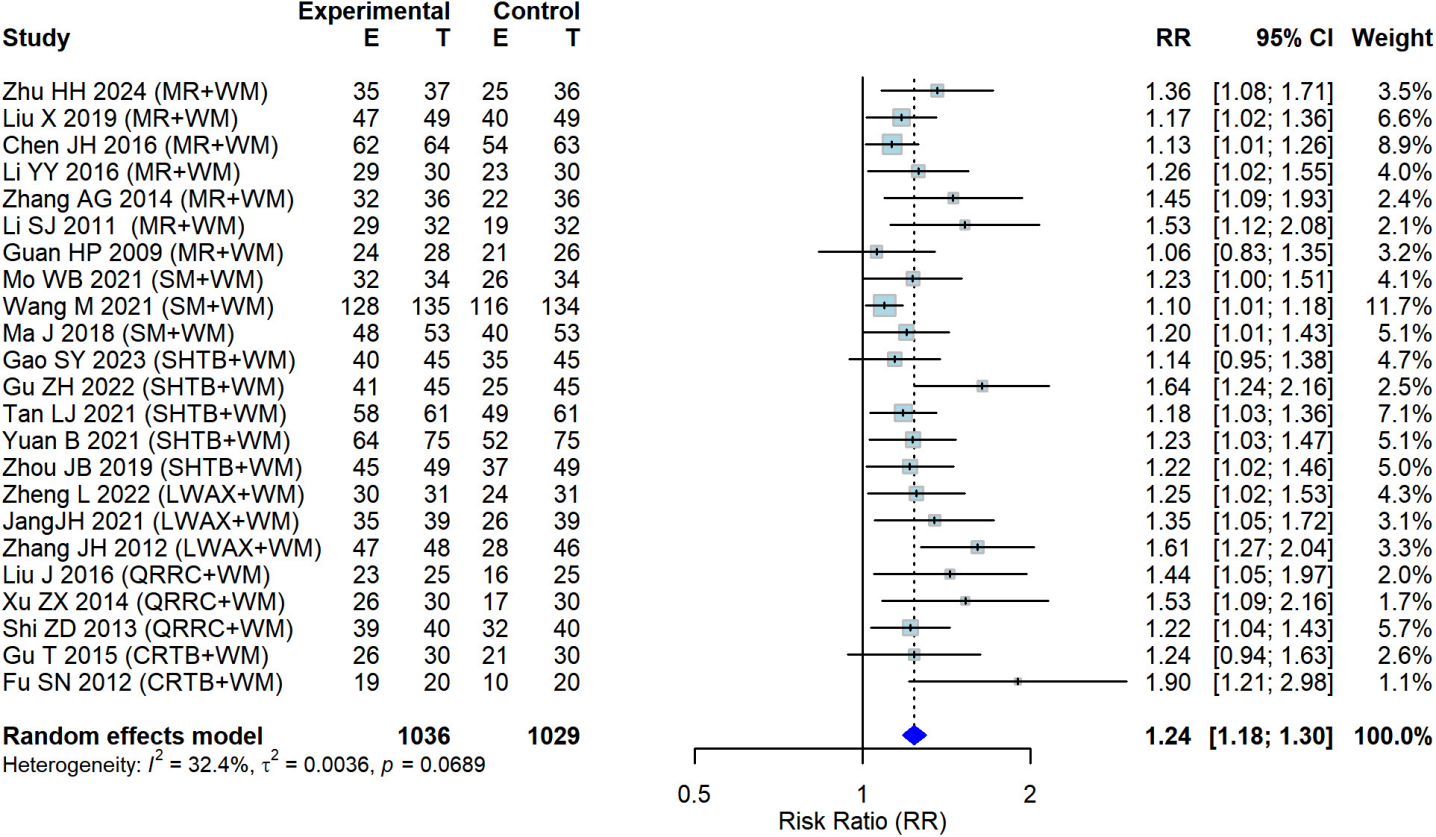
Forest plot of overall clinical effective rate: comparison between intervention (CPMs+WM) and control (WM alone) groups (RR, risk ratio; CI, confidence interval).

A total of 23 studies (12–34), involving seven intervention categories, reported the overall clinical effective rate. Figure 3 shows the forest plot of the pairwise random-effects meta-analysis pooling comparisons of CPMs + WM versus WM alone. Overall, CPMs + WM was associated with a higher effective rate than WM alone (RR = 1.24; 95% CI: 1.18–1.30; *P* < 0.001). Between-study heterogeneity was low to moderate (*I*^2^ = 32.4%; Cochran’s Q test *P* = 0.0689). Potential sources of this mild heterogeneity include differences in WM comparator regimens, variation in treatment duration, and non-uniform investigator-defined response criteria across trials (Table 1 and Supplementary Table S1).

#### 4.1.1 Network evidence graph

**FIGURE 4:**
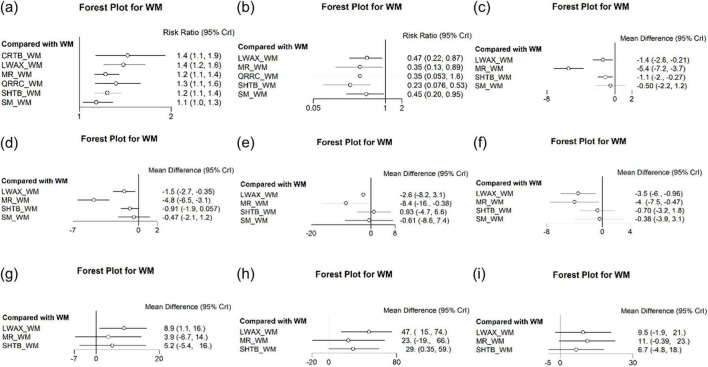
Forest plots of different interventions compared with WM alone: **(a)** overall clinical efficacy rate; **(b)** recurrence rate; **(c)** difficulty in defecation score; **(d)** abdominal discomfort symptom score; **(e)** stool consistency score; **(f)** defecation frequency score; **(g)** GAS; **(h)** MTL; **(i)** SP.

**FIGURE 5:**
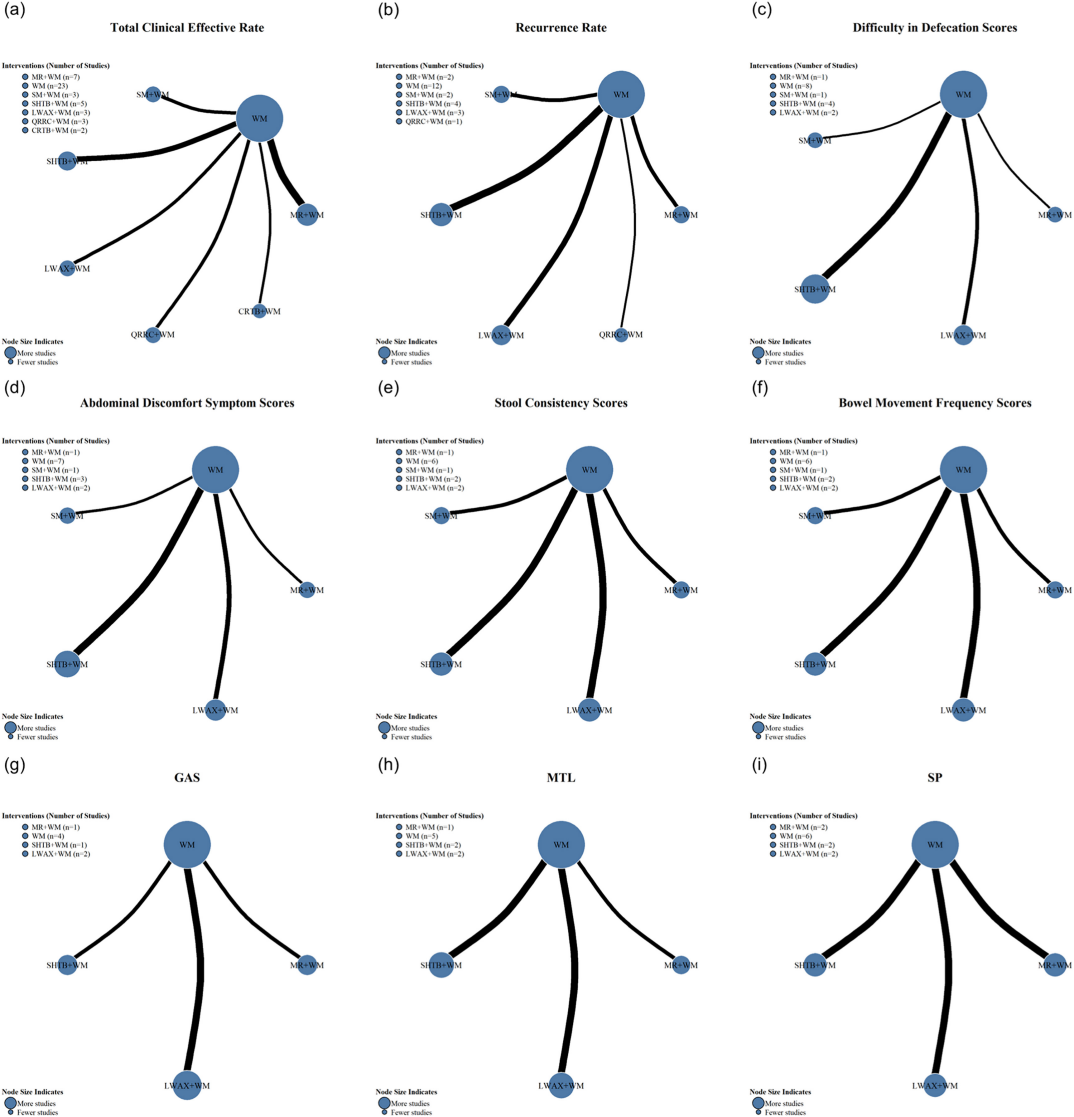
Network diagrams of interventions for elderly functional constipation for the following outcomes: **(a)** overall clinical efficacy rate; **(b)** recurrence rate; **(c)** difficulty in defecation score; **(d)** abdominal discomfort symptom score; **(e)** stool consistency score; **(f)** defecation frequency score; **(g)** GAS; **(h)** MTL; **(i)** SP.

Overall clinical effectiveness was reported in 23 RCTs, involving seven interventions: Maren-formula CPMs + WM, Qirong Runchang Oral Liquid + WM, Liuwei Anxiao Capsule + WM, Congrong Tongbian Oral Liquid + WM, Simo Decoction Oral Liquid + WM, Shouhui Tongbian Capsule + WM, and WM alone. The evidence network is shown in Figure 4a; nodes represent interventions and edges represent direct comparisons, with edge thickness reflecting the number of included studies. Forest plots comparing WM with each intervention are presented in Figure 5a.

#### 4.1.2 NMA

**FIGURE 6:**
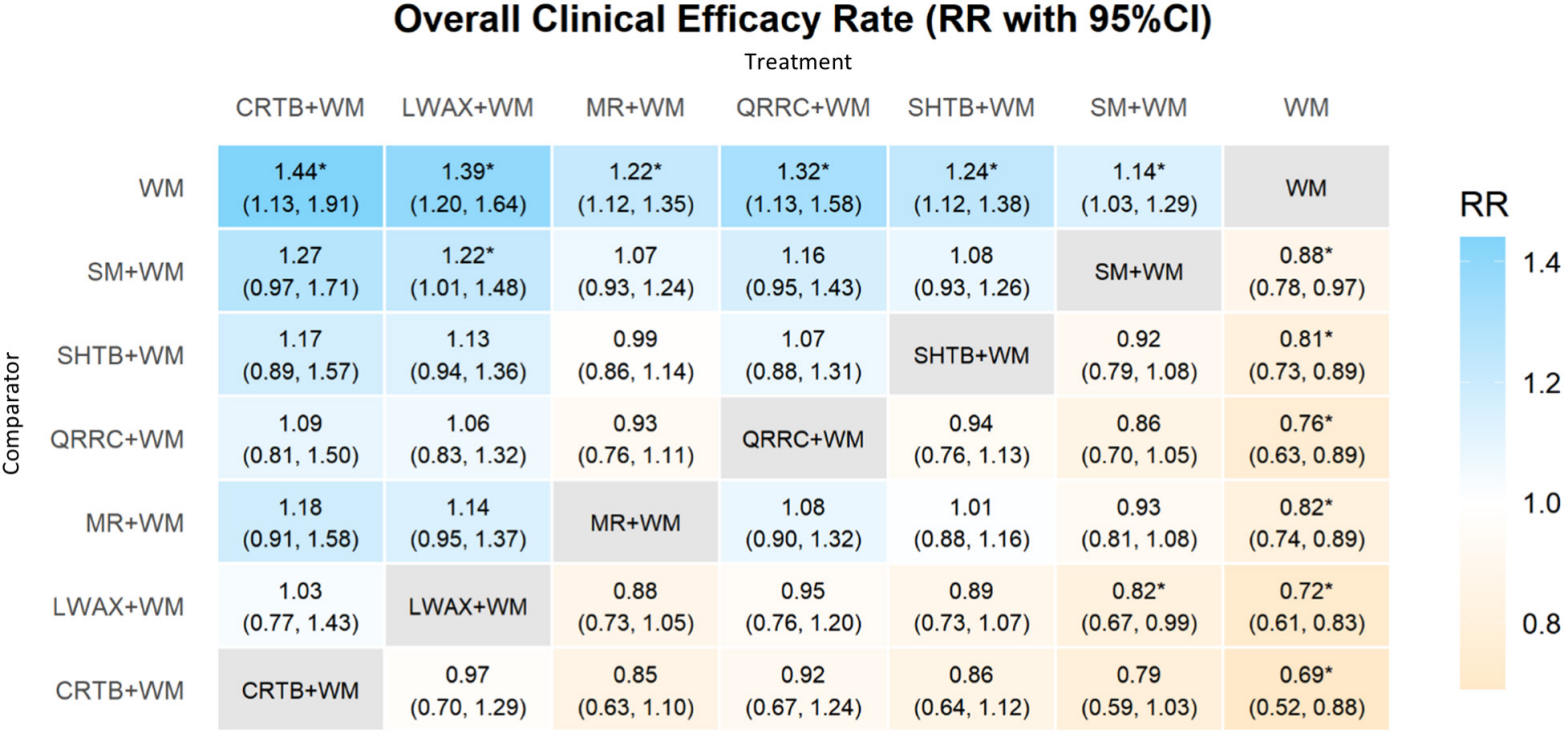
Heatmap of network meta-analysis for overall clinical efficacy rate. * indicates that the 95% credible interval (CrI) excludes the null value (RR/OR excludes 1; MD/SMD excludes 0).

A random-effects Bayesian NMA was conducted to allow pairwise comparisons among six CPM + WM regimens and WM alone. A total of 21 pairwise comparisons were estimated, and 7 comparisons had 95% CrIs excluding the null. For overall clinical effectiveness, the network meta-analysis suggested that the following CPM + WM combinations may be associated with higher response than WM alone (all evidence rated low/very low certainty): Liuwei Anxiao Capsule + WM (RR = 1.39, 95% CrI: 1.20–1.64); Maren-formula CPMs + WM (RR = 1.22, 95% CrI: 1.12–1.35); Congrong Tongbian Oral Liquid + WM (RR = 1.44, 95% CrI: 1.13–1.91); Simo Decoction Oral Liquid + WM (RR = 1.14, 95% CrI: 1.03–1.29); Shouhui Tongbian Capsule + WM (RR = 1.24, 95% CrI: 1.21–1.38); and Qirong Runchang Oral Liquid + WM (RR = 1.32, 95% CrI: 1.13–1.68). Compared with Simo Decoction Oral Liquid + WM, Liuwei Anxiao Capsule + WM showed a higher response in the network estimate (RR = 1.22, 95% CrI: 1.01–1.48), with low/very low certainty of evidence. Heterogeneity in investigator-defined response criteria and differences in WM regimens and treatment duration may partly explain between-study variability. Several comparisons showed wide 95% CrIs, indicating imprecision and limited clinical interpretability; therefore, effect magnitudes should not be over-interpreted (Figure 6).

### 4.1.3 Efficacy ranking

**FIGURE 7:**
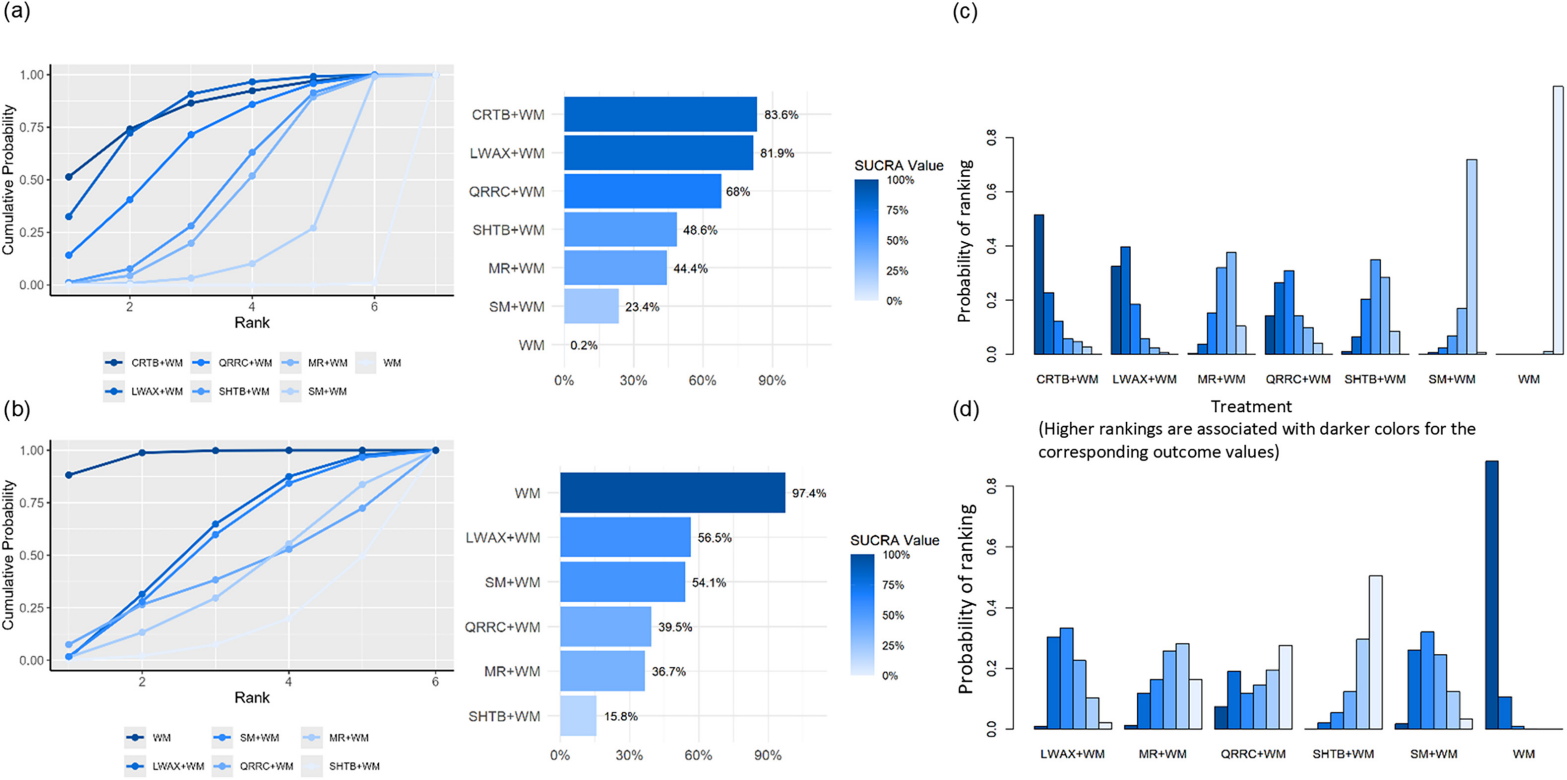
**(a)** SUCRA plot of different interventions for overall clinical efficacy rate; **(b)** SUCRA plot of different interventions for recurrence rate; **(c)** probability ranking bar plot of overall clinical efficacy rate; **(d)** probability ranking bar plot of recurrence rate.

Based on SUCRA, ranking probabilities for overall clinical effective rate were Congrong Tongbian Oral Liquid + WM (83.6%) > Liuwei Anxiao Capsule + WM (81.9%) > Qirong Runchang Oral Liquid + WM (68.0%) > Shouhui Tongbian Capsule + WM (48.6%) > Maren-formula CPMs + WM (44.4%) > Simo Decoction Oral Liquid + WM (23.4%) > WM (0.2%) (Figures 7a,c). SUCRA rankings are presented descriptively and should be considered exploratory.

#### 4.1.4 Leave-one-out sensitivity analysis

**FIGURE 8:**
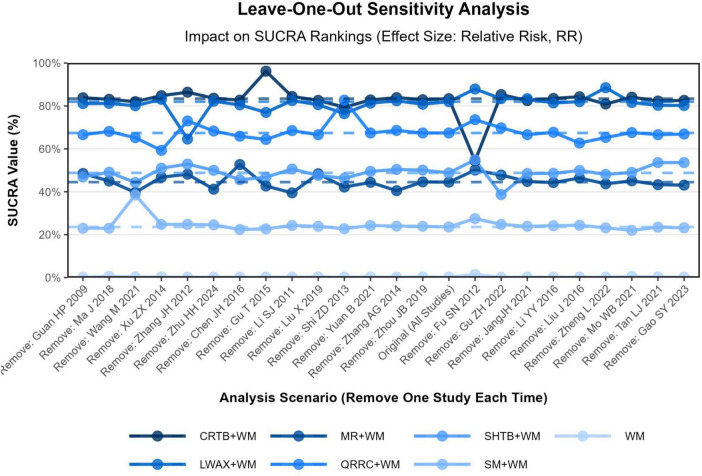
Leave-One-Out sensitivity analysis of overall clinical efficacy rate (changes in SUCRA values).

A leave-one-out sensitivity analysis was conducted by sequentially excluding each study and recalculating RRs and SUCRA values. The SUCRA ranking of the seven interventions remained broadly similar across iterations, with only minor changes after omitting individual studies. No single trial appeared to dominate the SUCRA pattern; however, this approach does not address limitations shared across trials (e.g., lack of blinding) and does not increase the certainty of evidence. The leave-one-out plot is provided in Figure 8.

### 4.2 Recurrence rate

#### 4.2.1 Network evidence graph

Recurrence rates were reported in 12 (14, 15, 20–24, 26–29, 32) RCTs, involving six interventions: Maren-formula CPMs + WM, Qirong Runchang Oral Liquid + WM, Liuwei Anxiao Capsule + WM, Simo Decoction Oral Liquid + WM, Shouhui Tongbian Capsule + WM, and WM alone. The evidence network is illustrated in Figure 4b, and the forest plot comparing WM with each intervention is shown in Figure 5b.

#### 4.2.2 NMA

**FIGURE 9:**
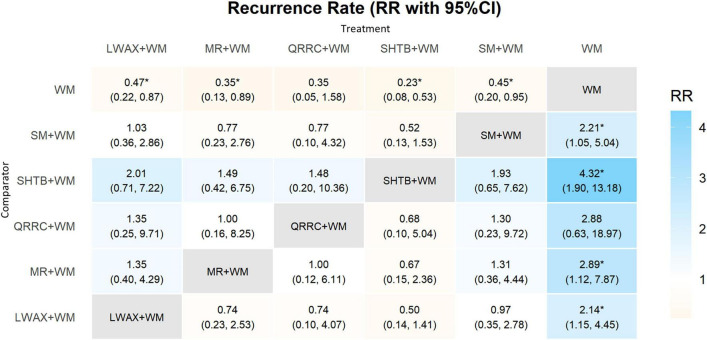
Heatmap of network meta-analysis for recurrence rate. * indicates that the 95% credible interval (CrI) excludes the null value (RR/OR excludes 1; MD/SMD excludes 0).

The NMA was conducted using a random-effects model, allowing for pairwise comparisons between five CPMs + WM and WM alone. A total of 15 pairwise comparisons were estimated, and 4 comparisons had 95% CrIs excluding the null (all evidence rated low/very low certainty). Compared with WM alone, the following CPM + WM combinations may be associated with lower recurrence: Liuwei Anxiao Capsule + WM (RR = 0.47, 95% CrI: 0.22–0.87); Maren-formula CPMs + WM (RR = 0.35, 95% CrI: 0.13–0.89); Shouhui Tongbian Capsule + WM (RR = 0.23, 95% CrI: 0.08–0.53); and Simo Decoction Oral Liquid + WM (RR = 0.45, 95% CrI: 0.20–0.95). Recurrence follow-up windows were heterogeneous or not reported, which limits comparability across trials. Several comparisons also had wide 95% CrIs due to sparse data, indicating substantial imprecision and limited clinical interpretability (Figure 9).

#### 4.2.3 Efficacy ranking

Based on SUCRA, ranking probabilities for recurrence (from more favorable to less favorable, where lower SUCRA indicates a lower recurrence risk) were: Shouhui Tongbian Capsule + WM (15.6%) < Maren-formula CPMs + WM (36.9%) < Qirong Runchang Oral Liquid + WM (39.5%) < Simo Decoction Oral Liquid + WM (54.6%) < Liuwei Anxiao Capsule + WM (56.2%) < WM (97.2%) (Figures 7b, d). SUCRA rankings are presented descriptively and should be considered exploratory.

### 4.3 Symptom scores for FC

#### 4.3.1 Network evidence graph

Defecation difficulty scores were reported in eight RCTs (13, 19, 23–28), while abdominal discomfort symptom scores were reported in seven RCTs (13, 19, 23, 25–28). Five RCTs (13, 19, 25, 27, 28) reported stool consistency score, and six RCTs (13, 19, 23, 26–28) reported defecation frequency score. All included RCTs involved four types of interventions. The evidence networks are shown in Figures 4c–f, and the corresponding forest plots comparing WM with each intervention are presented in Figures 5c–f.

#### 4.3.2 NMA

**FIGURE 10:**
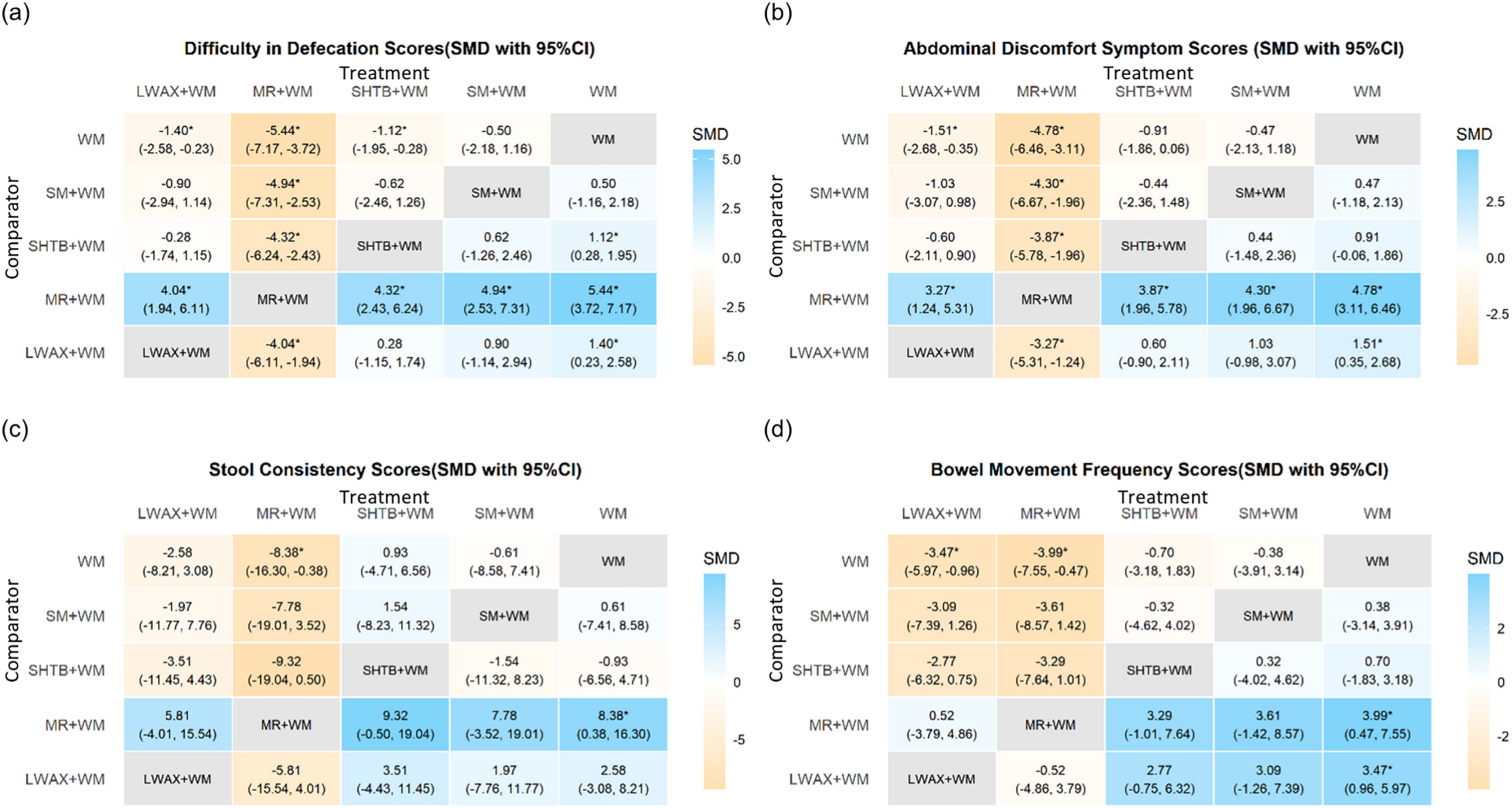
Heat map of network meta-analysis for symptom scores of different interventions; **(a)** difficulty in defecation score; **(b)** abdominal discomfort symptom score; **(c)** stool consistency score; **(d)** defecation frequency score. * Indicates that the 95% credible interval (CrI) excludes the null value (RR/OR excludes 1; MD/SMD excludes 0).

FC symptom outcomes were synthesized using SMDs under a random-effects model. For each symptom outcome, the network included four CPM + WM regimens and WM alone (10 possible pairwise comparisons per outcome). As shown in Figure 10 (upper triangle), negative SMDs indicate lower symptom scores and therefore favor CPMs + WM over WM alone. For defecation difficulty, CrIs excluding 0 were observed for Liuwei Anxiao Capsule + WM (SMD = −1.40, 95% CrI: −2.58 to −0.23), Maren-formula CPMs + WM (SMD = −5.44, 95% CrI: −7.17 to −3.72), and Shouhui Tongbian Capsule + WM (SMD = −1.12, 95% CrI: −1.95 to −0.28). For abdominal discomfort, CrIs excluding 0 were observed for Liuwei Anxiao Capsule + WM (SMD = −1.51, 95% CrI: −2.68 to −0.35) and Maren-formula CPMs + WM (SMD = −4.78, 95% CrI: −6.46 to −3.11). For stool consistency, only Maren-formula CPMs + WM showed a CrI excluding 0 versus WM alone (SMD = −8.38, 95% CrI: −16.30 to −0.38). For defecation frequency, CrIs excluding 0 were observed for Liuwei Anxiao Capsule + WM (SMD = −3.47, 95% CrI: −5.97 to −0.96) and Maren-formula CPMs + WM (SMD = −3.99, 95% CrI: −7.55 to −0.47). Notably, Several comparisons had extremely wide 95% CrIs, indicating severe imprecision due to sparse networks, with some intervention nodes informed by only one trial, which limits clinical interpretability (Figure 10). Given the subjective nature of symptom scoring and the risk of bias in many trials, the corresponding effect estimates and SUCRA rankings should be interpreted cautiously.

#### 4.3.3 Efficacy ranking

**FIGURE 11:**
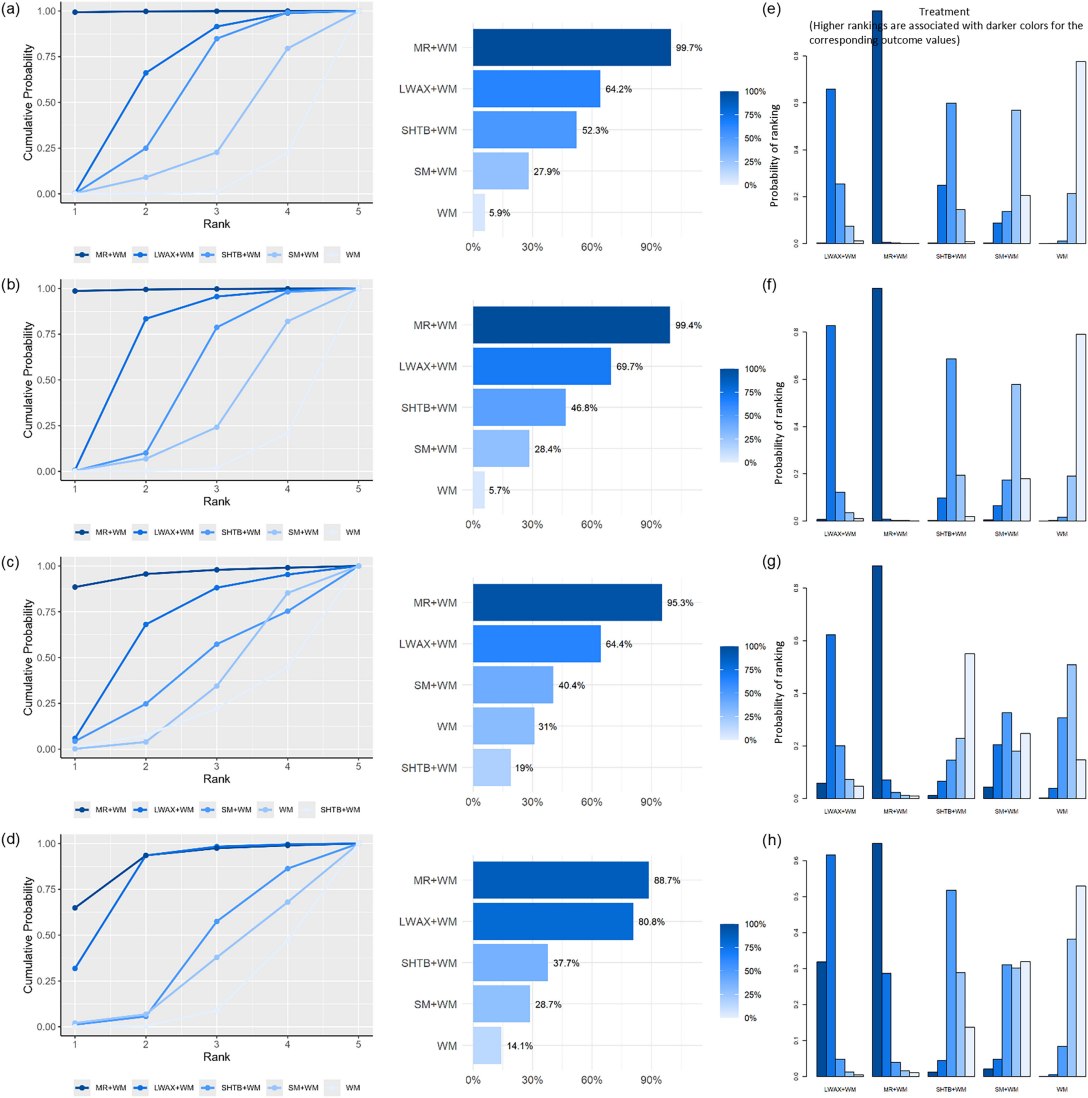
**(a)** SUCRA plot for difficulty in defecation score; **(b)** SUCRA plot for abdominal discomfort symptom score; **(c)** SUCRA plot for stool consistency score; **(d)** SUCRA plot for defecation frequency score; **(e)** Probability ranking bar plot of difficulty in defecation score; **(f)** Probability ranking bar plot of abdominal discomfort symptom score; **(g)** Probability ranking bar plot of stool consistency score; **(h)** Probability ranking bar plot of defecation frequency score.

Across the four symptom-score outcomes, SUCRA ranking probabilities suggested Maren-formula CPMs + WM generally had higher probabilities of being among the more favorable options (Figures 11a–h). SUCRA rankings are presented descriptively and should be considered exploratory.

### 4.4 Serum gastrointestinal hormone levels

#### 4.4.1 Network evidence graph

Gas was reported in four studies (12, 24, 27, 28), MTL in five studies (12, 24, 25, 27, 28), and SP in six studies (12, 13, 24, 25, 27, 28), each involving three interventions. The evidence networks are shown in Figures 4g–i, and the corresponding forest plots comparing WM with each intervention are presented in Figures 5g–i.

#### 4.4.2 NMA

**FIGURE 12:**
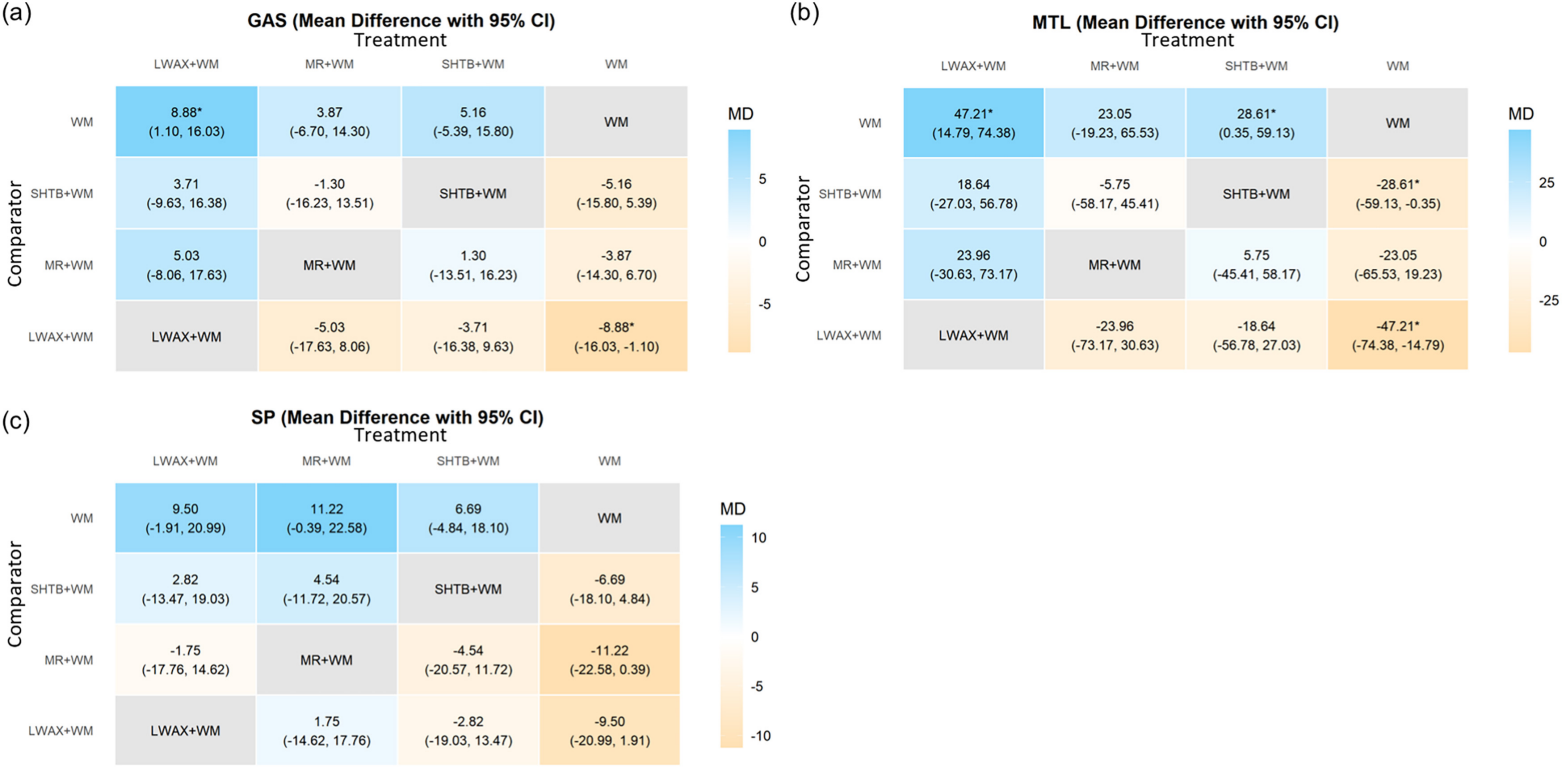
Heat map of network meta-analysis for serum gastrointestinal hormone levels of different interventions: **(a)** GAS; **(b)** MTL; **(c)** SP. * Indicates that the 95% credible interval (CrI) excludes the null value (RR/OR excludes 1; MD/SMD excludes 0).

The NMA was conducted using MDs under a random-effects model. For the three serum gastrointestinal hormone outcomes, networks included three CPMs + WM combinations and WM alone, yielding six possible pairwise comparisons per outcome. For GAS, Liuwei Anxiao Capsule + WM showed an increase versus WM alone with the 95% CrI excluding 0 (MD = 8.88, 95% CrI: 1.10–16.03). For MTL, Liuwei Anxiao Capsule + WM also showed an increase versus WM alone with the 95% CrI excluding 0 (MD = 47.21, 95% CrI: 47.21–74.38). For SP, none of the six pairwise comparisons had 95% CrIs excluding 0. These hormone networks included few studies, and several comparisons had wide 95% CrIs, indicating substantial imprecision and limited clinical interpretability (Figure 12).

#### 4.4.3 Efficacy ranking

**FIGURE 13:**
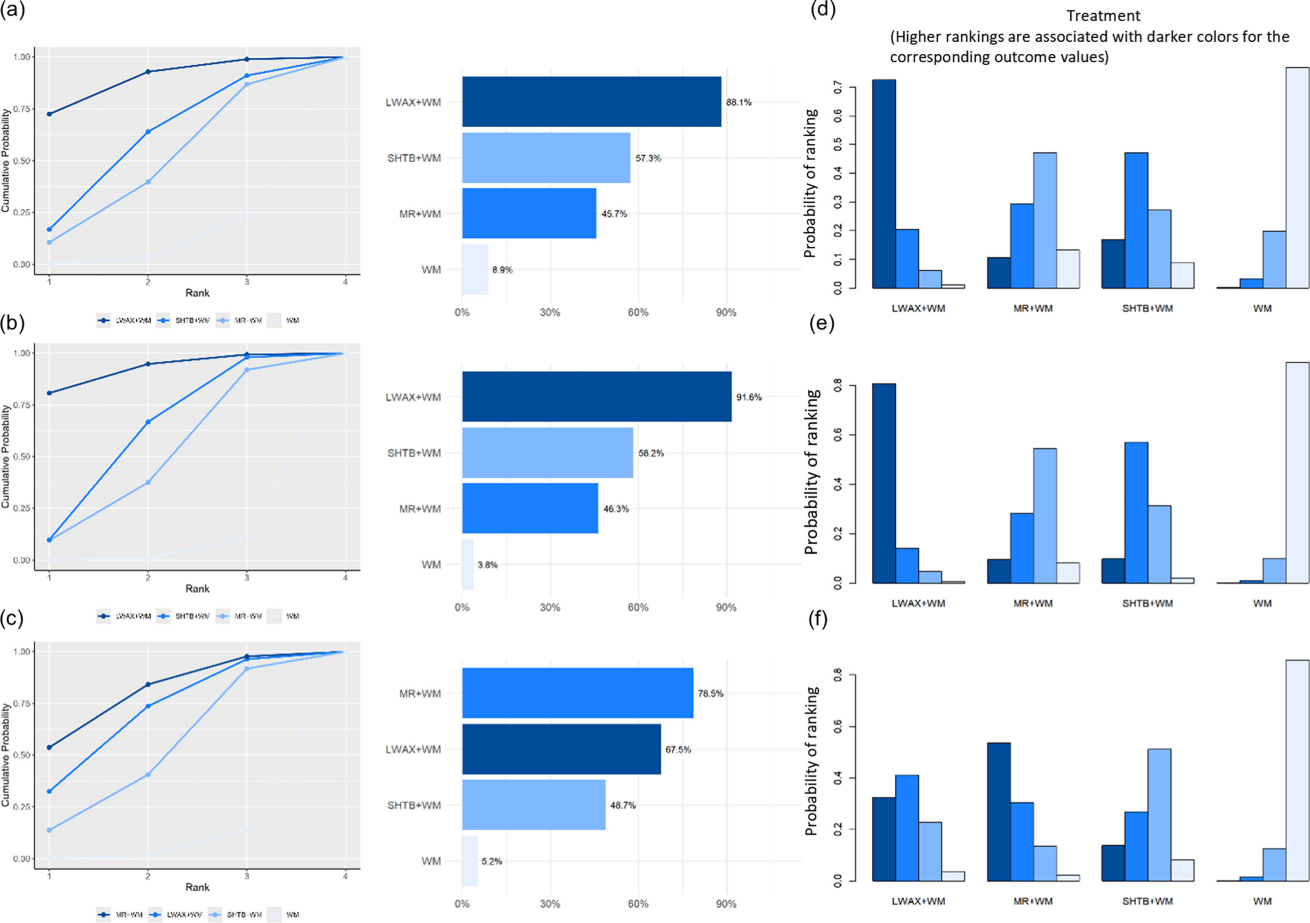
**(a)** SUCRA plot for GAS; **(b)** SUCRA plot for MTL; **(c)** SUCRA plot for SP; **(d)** probability ranking bar plot of GAS; **(e)** probability ranking bar plot of MTL; **(f)** probability ranking bar plot of SP

SUCRA ranking probabilities suggested Liuwei Anxiao Capsule + WM tended to rank higher for GAS and MTL, whereas Maren-formula CPMs + WM tended to rank higher for SP (Figures 13a–f). SUCRA rankings are presented descriptively and should be considered exploratory.

## 5 Adverse reactions

A total of 14 studies (14, 15, 18–20, 22, 24, 25, 28–31, 33, 34) reported adverse reactions during the intervention. Reporting was heterogeneous and often lacked prespecified AE definitions or systematic collection methods. These included three studies of Maren-formula CPMs, two studies of Simo Decoction Oral Liquid, two studies of Qirong Runchang Oral Liquid, two studies of Congrong Tongbian Oral Liquid, three studies of Shouhui Tongbian Capsule, and two studies of Liuwei Anxiao Capsule. Reported reactions were predominantly mild gastrointestinal symptoms (e.g., abdominal pain, diarrhea, bloating, nausea, and vomiting), and were often described as resolving after drug discontinuation or symptomatic management. No serious adverse events were reported in the included trials. To provide a clearer descriptive safety profile, Table 3 summarizes trial-level AE reporting and episode counts, and Table 4 provides an episode-based summary by intervention, including episode rates per 100 participants and episode-based rate ratios (RRs) with 95% CIs for CPM + WM versus WM (restricted to trials with numeric AE reporting). Because participant-level AE incidence (number of patients experiencing ≥ 1 AE) was frequently unavailable and some participants may have experienced more than one event, these episode-based summaries should be interpreted cautiously and do not represent incidence-based comparisons across trials. Moreover, AE definitions and ascertainment procedures were rarely standardized or clearly described, limiting comparability between studies. Therefore, we did not pool safety outcomes, and comparative safety conclusions remain uncertain. The absence of reported serious AEs should not be interpreted as evidence that serious AEs did not occur.

**TABLE 3:** Occurrence of adverse reactions in included studies.

Intervention	References	Sample		Number of cases (experimental group/control group)											Outcome of adverse reaction
		**Experimental group**	**control group**	**Abdominal pain**	**Diarrhea**	**Abdominal bloating**	**Abdominal discomfort**	**Nausea**	**Vomiting**	**Loose stools**	**Dry mouth**	**Mental fatigue and physical weakness**	**Dizzi-ness**	**Increased flatulence**	
MR+WM	Chen and Li (14)	64	63	3/7	–	–	–	–	–	–	–	–	–	–	NR
MR+WM	Li and Ouyang (15)	30	30	1/0	1/1	–	–	–	–	–	–	–	–	–	Resolved without treatment
SMT+WM	Mo (19)	34	34	0/1	–	–	–	–	–	1/0	–	–	–	–	NR
SMT+WM	Wang and Gao (20)	135	134	–	2/1	2/3	–	2/3	1/2	–	–	–	–	–	NR
SHTB+WM	Gao et al. (22)	45	45	1/2	2/2	2/3	–	–	–	–	–	0/1	–	–	NR
SHTB+WM	Tan et al. (24)	61	61	2/1	–	–	–	1/2	1/2	–	–	–	–	–	Resolved with symptomatic treatment
SHTB+WM	Yuan et al. (25)	75	75	1/2	–	0/2	–	–	–	–	–	–	–	1/0	Resolved after drug discontinuation
LWAX+WM	Jiang and Dong (28)	39	39	–	–	–	–	–	–	–	–	–	1/0	–	NR
LWAX+WM	Zhang (29)	48	46	2/3	3/1	–	–	–	–	–	2/2	–	–	–	Patients tolerated the symptoms following dose reduction or withdrawal within 2–3 days
MR+WM	Guana and Zu (18)	28	26	A small number of cases	–	–	A small number of cases	–	–	–	–	–	–	–	NR
QRRC+WM	Liu et al. (30)	25	25	–	1/0	–	1/4	–	–	–	–	–	–	–	Resolved after drug discontinuation
QRRC+WM	Xu et al. (31)	30	30	0/1	2/0	–	–	–	–	–	–	–	–	–	NR
CRTB+WM	Gu (33)	30	30	1/0	2/2	–	–	–	–	–	–	–	–	–	Resolved after drug discontinuation
CRTB+WM	Fu (34)	20	20	0/3	1/0	0/0	–	–	–	–	–	–	–	–	NR

**TABLE 4:** Episode-based summary of adverse events by intervention (restricted to trials with numeric AE reporting).

Intervention	Participants (CPM+WM)	Participants (WM)	Reported AE episodes (CPM+WM vs. WM)	Episodes per 100 participants (CPM+WM vs. WM)	RR (episode rate)	95% CI
CRTB + WM	50	50	4 vs. 5	8.0 vs. 10.0	0.80	0.21–2.98
LWAX + WM	87	85	8 vs. 6	9.2 vs. 7.1	1.30	0.45–3.75
MR + WM	122	119	5 vs. 8	4.1 vs. 6.7	0.61	0.20–1.86
QRRC + WM	55	55	4 vs. 5	7.3 vs. 9.1	0.80	0.21–2.98
SHTB + WM	181	181	11 vs. 17	6.1 vs. 9.4	0.65	0.30–1.38
SMT + WM	169	168	8 vs. 10	4.7 vs. 6.0	0.80	0.31–2.02

AE, adverse event. In this table reports episode-based AE counts/rates (episodes/participants), which may overestimate participant-level incidence. RRs (95% CIs) are episode-based rate ratios and are presented descriptively (not pooled) due to heterogeneous AE definitions/ascertainment and frequent lack of participant-level incidence; rates are not time-adjusted.

## 6 Discussion

FC is a functional bowel disorder characterized by reduced bowel movement frequency and difficulty in defecation, and it can markedly impair quality of life (35). It is considered a prototypical disorder of gut–brain interaction within the biopsychosocial model, with pathophysiology commonly involving delayed colonic transit, rectal evacuation dysfunction, intestinal microbiota imbalance, and neurosecretory disturbances along the brain–gut axis (36). Reduced colonic motility may decrease high-amplitude propagating contractions, leading to prolonged fecal retention and excessive water absorption; this has been linked to fewer interstitial cells of Cajal and altered intestinal neurotransmitters (e.g., 5-HT, VIP, and NO) (37). Defecatory disorders can also arise from inadequate rectal propulsive force or increased outlet resistance due to pelvic floor–anal sphincter discoordination, with anorectal manometry often showing impaired relaxation and elevated sensory thresholds (36). The gut microbiota—an important component of the brain–gut axis—may contribute via the microbiota–gut–brain axis through neuroendocrine, immune, and inflammatory pathways (38–40), and interactions with circadian rhythms have also been described (41). ICCs may further modulate gut–brain signaling and gastrointestinal motility (42). while dysregulated neuropeptides/hormones (e.g., CRF, SP, and VIP) can suppress colonic motility and increase visceral hypersensitivity (43). The interaction of these mechanisms results in more persistent symptoms in elderly FC patients. In clinical practice, the first-line treatment for elderly FC primarily involves permeability agents and stimulant laxatives. However, with prolonged use, patients tend to develop tolerance to commonly used laxatives, leading to recurrent and persistent difficulty in defecation. Therefore, there is an urgent need for a safe, multi-targeted alternative that can be used in long-term therapeutic cycles and effectively address the three-dimensional dysregulation involving colonic motility, outlet resistance, and gut microbiota.

In this Bayesian network meta-analysis, we synthesized evidence from 23 randomized controlled trials comparing six commonly used CPM formulations added to Western medicine (WM) versus WM alone for elderly functional constipation. Overall, CPMs as add-on therapy were associated with improved clinical outcomes compared with WM alone. Safety evidence was less informative because adverse events were inconsistently defined and reported. Although available reports generally described mild events, episode-based rate ratios (Table 4) were imprecise (wide 95% CIs, often crossing 1), precluding firm comparative safety conclusions. Given the low/very low certainty of evidence and the frequent high/unclear risk of bias, these findings warrant cautious interpretation and should be considered preliminary.

Risk of bias may have influenced the estimated effects. Most trials provided insufficient information on allocation concealment and lacked blinding of participants, personnel, and outcome assessors. Because key outcomes—particularly investigator-defined overall clinical effectiveness and symptom scores—are susceptible to performance and detection bias, treatment benefits may be overestimated. In addition, unavailable protocols and incomplete reporting raise concerns about selective reporting. Accordingly, both effect estimates and SUCRA-based rankings are best viewed as signals to guide future confirmatory trials rather than definitive evidence of comparative superiority.

From a longitudinal perspective, the evidence base is limited in its ability to inform durability of response and recurrence prevention. Treatment durations were generally short (often 1–5 weeks), and post-treatment recurrence assessment was variably timed or not reported, with follow-up windows ranging approximately from 2 to 24 weeks when available. In a chronic, relapsing condition such as FC, short or heterogeneous follow-up may overstate sustained benefit and miss late relapse. Future adequately powered multicenter RCTs should incorporate longer follow-up, prespecified recurrence definitions, and standardized assessment schedules.

Stability of secondary-outcome networks is an additional concern. Several secondary outcomes (e.g., symptom scores and gastrointestinal hormones) were informed by few trials, and some intervention nodes were supported by only one study in specific outcome networks. Under such sparse and imbalanced networks, both effect estimates and SUCRA ranking probabilities are inherently unstable and may shift as new evidence accumulates. While the leave-one-out analysis suggests that primary-outcome rankings were not driven by a single study, it does not address imprecision and fragility in secondary outcomes.

Against this background, mechanistic and pharmacological studies may provide supportive biological context for potential CPM effects, while remaining distinct from the clinical evidence synthesized here. Modern medical research suggests that traditional Chinese medicine (TCM) may exert therapeutic effects on FC by modulating interstitial cells of Cajal (ICC), gastrointestinal hormones, gut microbiota, aquaporin proteins, neurotransmitters, and related pathways (44). Several studies have further reported that Chinese patent medicine (CPM) formulations, typically administered as compound prescriptions, may improve intestinal function, as reflected by outcomes such as overall clinical effective rate and colonic transit function (45). In this context, external experimental, pharmacological, and clinical reports offer biological plausibility that several CPMs evaluated in this study may act through multi-target mechanisms, which may help contextualize (but do not confirm) the exploratory signals observed in the NMA. For example, Congrong Tongbian Oral Liquid has been reported to influence bile acid metabolism via the CYP8B1/FXR pathway and to be associated with restoration of the ICC network (e.g., increased c-kit expression) in experimental models (46). Liuwei Anxiao Capsule has been reported to improve slow transit constipation symptoms, potentially through modulation of the GALNT1/TGF-β1 pathway, reduction of inflammatory factors, and effects on ICC phenotypes, and it may also relate to changes in gastrointestinal hormone levels reported in prior studies (47). Shouhui Tongbian Capsule has been reported to modulate gastrointestinal hormones and to be associated with changes in cellular energy–related enzymes (e.g., ATP synthase and isocitrate dehydrogenase activity), which may relate to ICC function, enteric neural signaling, and intestinal peristalsis (48). Maren-formula CPMs have been discussed in network/pharmacological analyses with proposed constituents (e.g., kaempferol, nobiletin, aloe-emodin) and potential targets/pathways (e.g., AKT1, IL-6, VEGFA, TNF; PI3K-Akt, and AGE-RAGE signaling) (49). Animal studies further reported enhanced intestinal transit with concomitant changes in serum motilin (MTL), gastrin (GAS), acetylcholinesterase (AChE), and substance P (SP), and reduced somatostatin expression (50). While microbiome analyses suggested partial correction of dysbiosis with shifts in multiple taxa (51). Qirong Runchang Oral Liquid has been reported to promote intestinal motility and increase defecation frequency (52). Simo Decoction Oral Liquid is described in expert consensus as a prokinetic agent acting across the gastrointestinal tract and is often used in combination with other agents (e.g., lactulose or polyethylene glycol), with clinical reports describing potential benefits when combined with WM regimens such as probiotics or mosapride (53, 54). Importantly, these mechanistic observations are derived from external experimental/clinical literature and were not directly synthesized as outcomes in the present NMA; therefore, they should be interpreted as supportive context rather than confirmatory evidence of clinical superiority. In addition, the absence of patient-centered outcomes limits interpretation of clinical meaningfulness. Most trials focused on investigator-defined response criteria and symptom scores, whereas constipation-specific quality-of-life measures and patient global assessments were rarely reported. Future trials should incorporate validated, patient-centered endpoints alongside clinical and biochemical outcomes to better inform treatment decisions in elderly patients.

### 6.1 Limitations

(1) Low certainty and risk of bias: The certainty of evidence for all outcomes was rated as low or very low by GRADE, and most trials were judged at unclear/high risk of bias (e.g., limited blinding and incomplete reporting of randomization and allocation concealment). These limitations may have inflated effect estimates.

(2) Short duration and limited follow-up: Most trials had short treatment and follow-up periods (often 1–8 weeks). Recurrence assessment, when reported, occurred at variable post-treatment time points (approximately 2–24 weeks), and follow-up windows were frequently unreported. These limitations restrict conclusions regarding sustained efficacy, long-term recurrence prevention, and longer-term safety in elderly patients.

(3) Heterogeneity relevant to transitivity: WM comparator regimens and dosing varied and were often insufficiently described. CPM dosing/administration and precautions differed across products (Supplementary Material 3). Baseline constipation duration was frequently unreported. Outcome definitions and measurement approaches were also non-uniform, particularly for the investigator-defined overall clinical effective rate and symptom-score anchors (Supplementary Materials 1, 2). Collectively, these factors may act as effect modifiers and could threaten the transitivity assumption.

(4) Fragility of secondary-outcome networks: Several secondary outcomes were informed by few studies (often *n* ≤ 8), and some interventions were represented by only one trial within specific outcome networks (e.g., symptom-score domains). Consequently, effect estimates and SUCRA-based ranking probabilities for secondary outcomes may be unstable and could change materially as new evidence accumulates.

(5) Safety reporting limitations: Adverse-event reporting was heterogeneous, with limited information on prespecified definitions or ascertainment procedures, and participant-level AE incidence (number of patients with ≥ 1 AE) was often unavailable. Therefore, AE data were not comparable across trials and were not pooled. We reported episode-based AE rates and episode-based RRs with 95% CIs descriptively by intervention (Table 4); however, these summaries may overestimate participant-level incidence and were imprecise. The absence of reported serious AEs should not be interpreted as evidence that serious AEs did not occur.

(6) Open network and inability to assess inconsistency: Because the network had an open structure (no closed loops), statistical assessment of inconsistency was not feasible; therefore, indirect comparisons relied primarily on the transitivity assumption.

(7) Constraints on sensitivity analyses and meta-regression: Restriction-based sensitivity analyses limited to strictly comparable outcome definitions were not feasible without substantially compromising network connectivity. Quantitative adjustment (e.g., network meta-regression) was also not performed because of sparse data and incomplete reporting of key effect modifiers.

Accordingly, indirect estimates and SUCRA-based rankings should be interpreted cautiously and considered exploratory. Future adequately powered, rigorously designed multicenter RCTs should prioritize standardized WM comparators, prespecified and patient-centered outcomes (including quality of life), longer follow-up, and prespecified active adverse-event monitoring. If applicable, trials should also incorporate TCM syndrome differentiation.

## 7 Conclusion

This Bayesian network meta-analysis provides an exploratory synthesis comparing six CPM formulations combined with WM versus WM alone for elderly functional constipation. Although CPM + WM regimens generally appeared to improve outcomes compared with WM alone, the certainty of evidence was consistently low/very low and risk-of-bias concerns were prevalent, which may have inflated estimated effects. SUCRA-based rankings suggested potential comparative signals across outcomes; however, these rankings are hypothesis-generating and should not be interpreted as definitive indicators of clinical superiority or used to justify preferential selection of specific CPMs. Safety conclusions remain uncertain because adverse-event definitions and ascertainment methods were heterogeneous and often incompletely reported, and participant-level AE incidence was frequently unavailable; episode-based RRs were imprecise (wide 95% CIs, often crossing 1). Therefore, robust comparative safety inferences cannot be made, and the absence of reported serious AEs should not be interpreted as evidence that serious events did not occur. Long-term efficacy and recurrence prevention also remain unclear due to short, variable, and frequently unreported follow-up. Future adequately powered, rigorously designed multicenter RCTs with standardized and patient-centered outcomes, longer follow-up, and prespecified, actively collected adverse-event monitoring are needed to confirm these signals. Until higher-certainty evidence is available, clinical decision-making should remain guided by existing guideline recommendations.

## Data Availability

The original contributions presented in the study are included in the article/Supplementary material, further inquiries can be directed to the corresponding authors.

## References

[1] Drossman D. Functional gastrointestinal disorders: history, pathophysiology, clinical features, and rome iv. *Gastroenterology.* (2016) 150:1262–79. 10.1053/j.gastro.2016.02.032 27144617

[2] Lee K. The clinical implications of overlap between constipation and common functional gastrointestinal disorders. *J Neurogastroenterol Motil.* (2017) 23:485–6. 10.5056/jnm17111 28992672 PMC5628979

[3] Black C Ford A. Chronic idiopathic constipation in adults: epidemiology, pathophysiology, diagnosis and clinical management. *Med J Aust.* (2018) 209:86–91. 10.5694/mja18.00241 29996755

[4] Fabrizio A Alimi Y Kumar A. Methods of evaluation of anorectal causes of obstructed defecation. *Clin Colon Rectal Surg.* (2016) 30:46–56. 10.1055/s-0036-1593427 28144212 PMC5179274

[5] Bouras E Vazquez-Roque M. Epidemiology and management of chronic constipation in elderly patients. *Clin Interv Aging.* (2015) 10:919. 10.2147/CIA.S54304 26082622 PMC4459612

[6] Bharucha A Lacy B. Mechanisms, evaluation, and management of chronic constipation. *Gastroenterology.* (2020) 158:1232–49. 10.1053/j.gastro.2019.12.034 31945360 PMC7573977

[7] Ford A Suares N. Effect of laxatives and pharmacological therapies in chronic idiopathic constipation: systematic review and meta-analysis. *Gut.* (2011) 60:209–18. 10.1136/gut.2010.227132 21205879

[8] Chen M Hou X Xiong L. Chinese expert consensus on chronic constipation (2019, guangzhou). *Chung Hua Hsiao Hua Tsa Chih.* (2019) 39:577–98. 10.3760/cma.j.issn.0254-1432.2019.09.001

[9] Lembo A Schneier H Shiff S Kurtz C MacDougall J Jia X et al. Two randomized trials of linaclotide for chronic constipation. *N Engl J Med.* (2011) 365:527–36. 10.1056/NEJMoa1010863 21830967

[10] Khakisahneh S Wang J Zhang X Choi Y Han S Song E et al. Comparative evaluation of two herbal formulas for gastrointestinal function and gut microbiota modulation in rats with loperamide-induced dyspepsia. *Sci Rep.* (2025) 15:31186. 10.1038/s41598-025-15574-9 40854940 PMC12379153

[11] Zhu T Liu J Liu C Hua C. Risk of bias assessment tool 2.0 for cluster-randomized trials and crossover trials(revised version 2021): an interpretation. *Chinese J Evid-Based Med.* (2022) 22:842–52. 10.7507/1672-2531.202201070 38268090

[12] Zhu H Feng Z Yan L Huang X Zhou A Mao L. Clinical study on maren pills combined with bifidobacterium triple viable bacteria in treatment of elderly chronic functional constipation. *Drugs Clinic.* (2024) 39:2591–5. 10.7501/j.issn.1674-5515.2024.10.022

[13] Liu X Wang W Xia P Cai F. Clinical study on maren pills combined with srucalopride succinate in treatment of senile chronic constipation. *Drugs Clinic.* (2019) 34:3329–32. 10.7501/j.issn.1674-5515.2019.11.029

[14] Chen J Li Z. Combined use of maren pills and lactulose in the treatment of 64 elderly patients with functional constipation. *J Guangxi Univer Chinese Med.* (2016) 19:26–8.

[15] Li Y Ouyang J. Clinical observation of polyethylene glycol electrolyte powder combined with maren pills for senile functional constipation. *Chinese J Clin Rational Drug Use.* (2016) 9:95–6. 10.15887/j.cnki.13-1389/r.2016.19.051

[16] Zhang A. Cisapride combined with maren soft capsules for senile constipation. *J North Pharm.* (2014) 11:41.

[17] Li S Hu X Luo M. Maren capsules combined with cisapride in the treatment of 32 elderly patients with functional constipation. *Hunan J Traditional Chinese Med.* (2011) 27:19–20. 10.16808/j.cnki.issn1003-7705.2011.04.010

[18] Guan H Zu Z. Clinical observation of itopride combined with maren runchang pills in the treatment of 79 elderly patients with functional constipation. *J Aerospace Med.* (2009) 19:94. 10.3969/j.issn.2095-1434.2009.08.060

[19] Mo W. Clinical observation of simo decoction oral liquid combined with compound lactobacillus acidophilus tablets in the treatment of senile constipation. *China’s Naturopathy.* (2021) 29:92–4. 10.19621/j.cnki.11-3555/r.2021.1230

[20] Wang M Gao X. Analysis on the curative effect of lactulose combined with simotang oral liquid on senile constipation. *Syst Med.* (2021) 6:153–5. 10.19368/j.cnki.2096-1782.2021.11.153

[21] Ma J Cao X. Clinical observation of simotang oral liquid combined with bisacodyl in treatment of elderly functional constipation. *Drugs Clinic.* (2018) 33:2608–10. 10.7501/j.issn.1674-5515.2018.10.030

[22] Gao S Yang Y Liu R Wu B. Efficacy of shouhui tongbian capsules combined with flupentixol and melitracen in the treatment of elderly patients with chronic functional constipation accompanied with depression. *Chinese J Rational Drug Use.* (2023) 20:95–100. 10.3969/j.issn.2096-3327.2023.08.017

[23] Gu Z. Clinical observation of shouhui tongbian capsule in treating functional constipation in elderly patients. *China Sci Technol Database.* (2022):56–9.

[24] Tan L Wang Y Zhou B Si Z. Effect of shouhui tongbian capsules and bifidobacterium triple viable bacteria capsules in treatment of senile functional constipation differentiated as deficiency of qi and yin syndrome. *J Clin Med Pract.* (2021) 25:71–83. 10.7619/jcmp.20210985

[25] Yuan B Zhang J Wang S Shi X Li S. Effect of shouhui tongbian capsule combined with lactulose oral liquidon anxiety, depression and serum gastrointestinal hormones in patientswith senile functional constipation. *Xian Dai Sheng Wu Yi Xue Jin Zhan.* (2021) 21:3281–4. 10.13241/j.cnki.pmb.2021.17.018

[26] Zhou J Zhou J. Observation on the curative effect of shouhui tongbian capsule in the treatment of senile functional constipation. *Chinese General Pract.* (2019) 22:149–51.

[27] Zheng L. Observation on the effect of liuwei anxiao capsule combined with plucapride in the treatment of functional constipation in the elderly. *Capital Med.* (2023) 30:135–8. 10.3969/j.issn.1005-8257.2023.22.054

[28] Jiang J Dong Y. Efficacy of liuwei anxiao capsule combined with prucalopride on functional constipation in older patients. *Int J Geriatr.* (2021) 42:49–53. 10.3969/j.issn.1674-7593.2021.01.014

[29] Zhang JH. A effect analysis of combining liuweianxiao capsule with mospride citrate tablets for functional constipation in old patients. *J Math Med.* (2012) 25:330–1. 10.3969/j.issn.1004-4337.2012.03.030

[30] Liu J Zhang L De J. Clinical efficacy of qirong runchang oral liquid combined with forlax in elderly patients with constipation. *E-Journal Transl Med.* (2016) 3:25–6.

[31] Xu Z Lu J Wu S Cai L. Clinical study of qirong runchang oral liquid combined with polyglucosamine capsules in the treatment of senile functional constipation. *J Pract Traditional Chinese Med.* (2014) 30:854. 10.3969/j.issn.1004-2814.2014.09.051

[32] Shi Z. Clinical efficacy of qirong runchang oral liquid combined with lactulose in the treatment of very elderly patients with functional constipation: a randomized controlled trial. *J China Pharm.* (2013) 24:2184–6. 10.6039/j.issn.1001-0408.2013.23.26

[33] Gu T Yu Y Zhang Z Yu X Yao J. The effect of congrong purgative oral solution combined with lactulose on the treatment of elderly constipation patients. *Geriatr Health Care.* (2015) 21:109–11. 10.3969/j.issn.1008-8296.2015-14

[34] Fu S. Efficacy and safety of mosapride combined with congrong purgative oral solution in treatment of senile constipation. *China Modern Doctor.* (2012) 50:70–1. 10.3969/j.issn.1673-9701.2012.07.033

[35] Nelson A Camilleri M Chirapongsathorn S Vijayvargiya P Valentin N Shin A et al. Comparison of efficacy of pharmacological treatments for chronic idiopathic constipation: a systematic review and network meta-analysis. *Gut.* (2017) 66:1611–22. 10.1136/gutjnl-2016-311835 27287486

[36] Guo Q Luo J. Progress in diagnosis and treatment comorbidities of functional constipation and anxiety and depression in elder patient. *J Modern Med Health.* (2024) 40:2136–40. 10.3969/j.issn.1009-5519.2024.12.033

[37] Zhan Y Liu Y Jiang J Lu L Xu H Tang X. Functional constipation—research progress on gut microbiota-related motility disorders. *Chinese J Integr Traditional Western Med Digestion.* (2019) 27:557–62. 10.3969/j.issn.1671-038X.2019.07.18

[38] Zhang C Duan L. Recent advances on effect and mechanism of gut microbiota in development of enteric nervous system. *Weichangbingxue He Ganbingxue Zazhi.* (2022) 31:502–7. 10.3969/j.issn.1006-5709.2022.05.005

[39] Gray T Fasina Y Harrison S Chang E Chang A Maldonado-Devincci A et al. Exploring the impact of a high-fat diet on the serotonin signaling in gut-brain axis. *Nutr Neurosci.* (2025) 29:40–54. 10.1080/1028415X.2025.2539320 40853783

[40] Xu M. Analysis of correlation of sleep disturbance with anxiety, depression, and quality of life in elderly patients with chronic constipation based on“brain-gut axis” theory. *Shi Jie Hua Ren Xiao Hua Za Zhi.* (2020) 28:129–34. 10.11569/wcjd.v28.i4.129

[41] Zuo X Wu Q. Relationship between intestinal diseases and circadian rhythm disorders: a review. *J Pract Med.* (2022) 38:2363–6. 10.3969/j.issn.1006-5725.2022.18.020

[42] Guo Q Yang G Qin W Zhang S Sun H Yu Y et al. Research progress on the pathogenesis of functional constipation and acupuncture interventions. *J Liaoning Univer Traditional Chinese Med.* (2022) 24:203–6. 10.13194/j.issn.1673-842x.2022.11.042

[43] Zhang M Li J Shang L Zhou F Yang S. Research progress on the effects of dietary nutrition on digestive psychosomatic disorders based on microbiota-gut-brain axis. *Chinese J Integr Traditional Western Med Digest.* (2023) 31:29–36. 10.3969/j.issn.1671-038X.2023.01.06

[44] Cai M An R Yang Y Mao H. Research progress on the mechanisms of traditional chinese medicine in treating functional constipation by regulating cajal interstitial cells. *Hebei J Traditional Chinese Med.* (2021) 43:1387–91. 10.3969/j.issn.1002-2619.2021.08.034

[45] Ji L Fan Y Li L Bai H Weng L Zhao P. Efficacy and safety of chinese herbal compound in the treatment of functional constipation. *Medicine.* (2020) 99:e22456. 10.1097/MD.0000000000022456 32991483 PMC7523752

[46] Yin H Gao X Yang H Xu Z Wang X Wang X et al. Total alditols from cistanche deserticola attenuate functional constipation by regulating bile acid metabolism. *J Ethnopharmacol.* (2024) 320:117420. 10.1016/j.jep.2023.117420 37967778

[47] Liu C Wang Y Wang J Yu Y Zhang P Zhao X et al. Study on clinical efficacy and mechanism of mosapride combined with liuwei anxiao capsule in the treatment of slow transit constipationof rats. *J Clin Exp Med.* (2022) 21:13–7. 10.3969/j.issn.1671-4695.2022.01.004

[48] Gong L Du H Guo X Li J Zhu X Shen X et al. Shouhui tongbian capsule in treatment of constipation: treatment and mechanism development. *Chin Herb Med.* (2024) 16:239–47. 10.1016/j.chmed.2023.05.006 38706823 PMC11064553

[49] Ren X Zhou X. Study on the mechanism of maren pill in treating constipation based on network pharmacology. *J China Prescription Drug.* (2024) 22:10–3. 10.3969/j.issn.1671-945X.2024.01.004

[50] Gao Z Fu L Bai W Liang J. Protective effects of medicinal plant-derived metabolites on slow transit constipation via the ens-icc-smc pathway. *Front Pharmacol.* (2025) 16:1598806. 10.3389/fphar.2025.1598806 40567379 PMC12188447

[51] Qi Y Niu M Zhang L. Study on the effects of ma ren wan on gut microbiota in rats with functional constipation based on high-throughput sequencing technology. *Chinese Traditional Patent Med.* (2023) 45:2388–92. 10.3969/j.issn.1001-1528.2023.07.04

[52] Zhang Y Yuan B Zhou Z. Study on the effect of qirong runchang oral liquid in the treatment of chronic constipation in the elderly. *China Pract Med.* (2020) 15:107–9. 10.14163/j.cnki.11-5547/r.2020.24.046

[53] Bian L Zou D Ke X Lan Y Tang Y Li J et al. Expert consensus on treatment of chronic gastrointestinal diseases and gastrointestinal disorders after abdominal operation by simotang oral liqui. *Chinese Arch Traditional Chinese Med.* (2021) 39:254–8. 10.13193/j.issn.1673-7717.2021.07.063

[54] Ren L Yan M Ren S. Clinical efficacy of si mo tang oral liquid combined with quadruple therapy in patients with dyspepsia from *helicobacter* pylori-associated gastritis with liver-stomach qi stagnation syndrome. *Chinese Traditional Patent Med.* (2025) 47:2477–80. 10.3969/j.issn.1001-1528.2025.07.059

