# Supplementary material for: Efficacy and safety of six Chinese patent medicines for elderly functional constipation: a network meta-analysis
Source: Front Med (Lausanne). 2026 Mar 31;13:1728217. doi: 10.3389/fmed.2026.1728217 (PMC13085306; doi:10.3389/fmed.2026.1728217)
Supplement: Supplementary file 1 [file Data_Sheet_1.zip › Supplementary_Material/Network analysis script (R code).docx]

library(gemtc)

library(reshape2)

library(ggplot2)

# Network plot code with plus signs

library(igraph) # For network graph analysis and visualization

library(gemtc) # For network meta-analysis

library(extrafont) # For managing fonts in graphics

loadfonts(device = "win") # Initialize font system to ensure Times New Roman is available

setwd("D:/working directory") # Set working directory

data <- read.csv("data.csv", sep = ",", header = TRUE) # Read data

network <- mtc.network(data)

# Create network graph

g <- make_empty_graph(directed = FALSE) # Create undirected graph

all_treatments <- unique(data$treatment) # Get all unique treatment names

g <- add_vertices(g, nv = length(all_treatments), name = all_treatments) # Add vertices

# Calculate total occurrence count for each treatment across all studies

treatment_counts <- table(data$treatment)

cat("Treatment occurrence statistics:\n")

print(treatment_counts)

# Add occurrence count as vertex attribute

V(g)$count <- as.numeric(treatment_counts[V(g)$name])

studies <- unique(data$study) # Get all unique study IDs

for(s in studies) { # Iterate through each study to create connections (edges) between treatments

treatments_in_study <- data$treatment[data$study == s] # Get all treatments in current study

treatments_in_study <- unique(treatments_in_study) # Ensure uniqueness

# If study has multiple treatments, create all possible connections between them

if(length(treatments_in_study) > 1) {

pairs <- combn(treatments_in_study, 2, simplify = TRUE) # Generate all pairwise combinations

for(i in 1:ncol(pairs)) { # Iterate through each treatment pair

v1_id <- which(V(g)$name == pairs[1, i]) # Find vertex IDs

v2_id <- which(V(g)$name == pairs[2, i])

# Check if vertices are already connected

if(are_adjacent(g, v1_id, v2_id)) {

eid <- get_edge_ids(g, c(v1_id, v2_id)) # If already connected, increase weight (co-occurrence count)

E(g)$weight[eid] <- E(g)$weight[eid] + 1

} else { # If not connected, create new edge with initial weight

g <- add_edges(g, c(v1_id, v2_id), weight = 1)

}

}

}

}

# Set vertex attributes

V(g)$color <- "#4E79A7" # Vertex fill color (dark blue)

V(g)$frame.color <- "white" # Vertex border color

V(g)$label.color <- "black" # Vertex label color

V(g)$label.family <- "serif" # Vertex label font family

V(g)$label.cex <- 0.8

# Set vertex size - based on treatment occurrence count in studies

if(length(V(g)) > 0 && max(V(g)$count) > 0) {

V(g)$size <- 10 + (V(g)$count / max(V(g)$count)) * 30

} else {

V(g)$size <- 15 # Default size

}

# Set edge attributes

if("weight" %in% edge_attr_names(g)) { # Edge width proportional to weight (co-occurrence count)

E(g)$width <- 1 + (E(g)$weight / max(E(g)$weight)) * 10

} else {

E(g)$width <- 2 # Default width

}

E(g)$color <- "black" # Edge color

layout <- layout_in_circle(g) # Circular layout

V(g)$display_label <- gsub("_", "+", V(g)$name) # Create display labels: replace underscores with plus signs

# Save as high-quality image

tiff("network.tiff",

width = 15, height = 15, # Width 15cm (adjustable between 8.5-20cm, e.g., 12/18)

units = "cm", # Units changed to centimeters to match requirements directly

res = 300, # 300dpi resolution (high quality, academic publication standard)

compression = "lzw") # LZW compression: reduces file size without quality loss

par(mai = c(0, 0, 1, 0), # Set graphic margins

family = "serif") # Global font settings

# Plot network graph

plot(g,

layout = layout, # Use circular layout

vertex.frame.color = "white", # Vertex border color

vertex.label = V(g)$display_label, # Use converted labels

edge.curved = 0.2, # Slightly curved edges to avoid overlap

main = "Recurrence Rate", # Main title

cex.main = 1.2) # Main title font size

# Add legend including vertex size explanation - using converted labels

legend("topleft",

legend = paste0(V(g)$display_label, " (n=", V(g)$count, ")"), # Use converted labels

pt.bg = V(g)$color, # Legend point background color

pch = 21, # Legend point shape (filled circle)

pt.cex = 1.2, # Legend point size

cex = 0.7, # Legend text size

bty = "n", # No border

title = "Interventions (Number of Studies)", # Legend title

title.font = 2) # Legend title bold

# Add vertex size explanation

legend("bottomleft",

legend = c("More studies", "Fewer studies"), # Explanation text

pt.cex = c(2.5, 1.0), # Point size examples

pch = 21, # Point shape

pt.bg = "#4E79A7", # Point color

cex = 0.7, # Text size

bty = "n", # No border

title = "Node Size Indicates", # Explanation title

title.font = 2) # Title bold

dev.off()
