# Supplementary material for: Efficacy and safety of six Chinese patent medicines for elderly functional constipation: a network meta-analysis
Source: Front Med (Lausanne). 2026 Mar 31;13:1728217. doi: 10.3389/fmed.2026.1728217 (PMC13085306; doi:10.3389/fmed.2026.1728217)
Supplement: Supplementary file 1 [file Data_Sheet_1.zip › Supplementary_Material/RR some analysis script (R code).docx]

setwd("D:/working directory")

library(coda)

library(gemtc)

library(reshape2)

library(ggplot2)

library(scales)

data <- read.csv("data.csv", sep=",", header=T)

print(head(data)) # Check if data is read correctly

network <- mtc.network(data)

plot(network) # Check network structure

model <- mtc.model(network, type="consistency",

n.chain=4, likelihood="binom", link="log", linearModel="random")

results <- mtc.run(model, n.adapt = 20000, n.iter = 50000, thin = 1)

plot(results) # Check model running results

gelman.plot(results) # Check convergence

resultanohe <- mtc.anohe(network, n.adapt = 20000, n.iter = 50000, thin = 1,

n.chain=4, likelihood="binom", link="log", linearModel="random")

plot(summary(resultanohe))

# Create treatment name mapping function: convert underscores to plus signs

convert_underscore_to_plus <- function(names) {

gsub("_", "+", names)

}

# Save forest plot as high-quality TIFF

treatments <- unique(network$treatments$description)

converted_treatments <- convert_underscore_to_plus(treatments)

for(i in 1:length(treatments)) {

tiff(paste0("Recurrence_rate_forest_", converted_treatments[i], ".tiff"),

width = 15, height = 10, units = "cm", res = 300)

forest(relative.effect(results, treatments[i]))

title(main = paste("Forest Plot for", converted_treatments[i]))

dev.off() # Ensure graphics device is closed

}

a <- round(exp(relative.effect.table(results)), 2)

rownames(a) <- convert_underscore_to_plus(rownames(a))

colnames(a) <- convert_underscore_to_plus(colnames(a))

write.csv(a, "leaguetable.csv")

ranks <- rank.probability(results, preferredDirection = 1)

rownames(ranks) <- convert_underscore_to_plus(rownames(ranks))

print(ranks)

# Create SCI-style blue gradient palette - reversed color depth

sci_blue_palette <- colorRampPalette(c("#004C99", "#0066CC", "#1A80FF", "#4D9CFF", "#80B8FF", "#B3D4FF", "#E6F0FF"))

# Point 4: Save rank probability plot as high-quality TIFF - using SCI blue

tiff("Recurrence_stacked.tiff", width = 20, height = 15, units = "cm", res = 300)

par(mar = c(4, 4, 2, 6), cex = 0.8) # Reduce margins and font size

plot(ranks, col = sci_blue_palette(ncol(ranks)))

dev.off() # Ensure graphics device is closed

tiff("Recurrence_beside.tiff", width = 20, height = 10, units = "cm", res = 300)

par(mar = c(4, 4, 2, 6), cex = 0.8) # Reduce margins and font size

plot(ranks, beside = TRUE, col = sci_blue_palette(ncol(ranks)))

dev.off() # Ensure graphics device is closed

# Calculate SUCRA values

sucra_values <- sucra(ranks)

# Create refined SUCRA plot

a <- ranks[,]

b <- reshape2::melt(a)

c <- as.data.frame(b)

# Convert variable names

c$Var1 <- convert_underscore_to_plus(c$Var1)

# Sort by SUCRA values, placing better performing ones first

sucra_order <- names(sort(sucra_values, decreasing = TRUE))

c$Var1 <- factor(c$Var1, levels = sucra_order)

p <- ggplot(c, aes(x=Var2, y=value, colour=Var1)) +

geom_line(linewidth=1) +

scale_color_manual(values = sci_blue_palette(length(unique(c$Var1))))

p2 <- p + geom_point(size=2) +

labs(x="Rank", y="Probability") +

theme(axis.text = element_text(size=10),

axis.title = element_text(size=12),

axis.line = element_line(color="black"),

legend.title = element_blank(),

legend.position = "bottom",

legend.text = element_text(size=8))

ggsave("Recurrence_probability.tiff", p2, width = 15, height = 12, units = "cm", dpi = 300)

# Cumulative probability plot

d <- t(apply(ranks, 1, cumsum))

e <- reshape2::melt(d)

f <- as.data.frame(e)

# Convert variable names

f$Var1 <- convert_underscore_to_plus(f$Var1)

# Sort by SUCRA values

f$Var1 <- factor(f$Var1, levels = sucra_order)

p <- ggplot(f, aes(x=Var2, y=value, colour=Var1)) +

geom_line(linewidth=1) +

scale_color_manual(values = sci_blue_palette(length(unique(f$Var1))))

p2 <- p + geom_point(size=2) +

labs(x="Rank", y="Cumulative Probability") +

theme(axis.text = element_text(size=10),

axis.title = element_text(size=12),

axis.line = element_line(color="black"),

legend.title = element_blank(),

legend.position = "bottom",

legend.text = element_text(size=8))

ggsave("Recurrence_cumulative_probability.tiff", p2, width = 13, height = 10, units = "cm", dpi = 300)

# SUCRA value processing (key fix: retain original values for color mapping)

sucra_values <- sucra(ranks)

print(sucra_values)

# Convert SUCRA values to data frame, including original values and percentages

sucra_df <- data.frame(

Treatment = convert_underscore_to_plus(names(sucra_values)),

SUCRA_percent = paste0(round(sucra_values * 100, 1), "%"),

SUCRA_value = sucra_values # Retain original values for color mapping

)

# Print formatted SUCRA table

cat("\nSUCRA Values (Percentage):\n")

print(sucra_df %>% select(Treatment, SUCRA_percent), row.names = FALSE)

# Save SUCRA bar plot as high-quality TIFF - colors linked to SUCRA values (core fix)

tiff("sucra_barplot.tiff", width = 13, height = 9, units = "cm", res = 300)

# Sort by SUCRA values

sucra_df_sorted <- sucra_df %>% arrange(SUCRA_value)

# Create bar plot with colors linked to SUCRA values

sucra_plot <- ggplot(sucra_df_sorted, aes(x = reorder(Treatment, SUCRA_value),

y = SUCRA_value,

fill = SUCRA_value)) + # Color determined by SUCRA value

geom_bar(stat = "identity") +

geom_text(aes(label = SUCRA_percent),

hjust = -0.2, size = 3) +

# Set color gradient: higher SUCRA values correspond to darker colors

scale_fill_gradientn(

colors = rev(sci_blue_palette(7)), # Reverse palette so high values correspond to dark colors

limits = c(0, 1),

labels = scales::percent_format(accuracy = 1),

name = "SUCRA Value"

) +

scale_y_continuous(limits = c(0, 1.1),

labels = scales::percent_format(accuracy = 1)) +

coord_flip() +

labs(title = "",#SUCRA Values (Surface Under the Cumulative Raking Curve)

x = "", # Interventions

y = "") + # SUCRA Value

theme_minimal() +

theme(

legend.position = "right", # Show legend to explain color meaning

axis.text = element_text(size = 10),

axis.title = element_text(size = 12),

plot.title = element_text(size = 14, hjust = 0.5),

legend.text = element_text(size = 8),

legend.title = element_text(size = 10)

)

print(sucra_plot)

dev.off()

# Create heatmap

library(tidyverse)

library(ggplot2)

# Define conversion function: convert underscores to plus signs

# Create dichotomous data RR value heatmap (replacing original SMD heatmap)

# Note: RR (Relative Risk) is used for dichotomous outcomes (such as recurrence rate, incidence rate),

# RR=1 indicates no effect, RR<1 indicates intervention reduces risk, RR>1 indicates intervention increases risk

# 1. Read league table data (ensure data is in RR value format: "RR value (95%CI lower limit,95%CI upper limit)")

# If data is in log(RR) format, need to remove exp() conversion step; here we assume reading already exponentiated RR values

df <- read.csv("leaguetable.csv", row.names = 1, stringsAsFactors = FALSE)

# 2. Uniformly process intervention names (underscore/dot to plus sign, maintain name consistency)

convert_name <- function(names) {

names <- gsub("_", "+", names) # Underscore to plus sign (e.g., "A_B"→"A+B")

names <- gsub("\\.", "+", names) # Dot to plus sign (e.g., "A.B"→"A+B")

return(names)

}

rownames(df) <- convert_name(rownames(df)) # Row names (reference interventions)

colnames(df) <- convert_name(colnames(df)) # Column names (comparison interventions)

# 3. Data reshaping and RR value specific processing (core modification: adapt to dichotomous RR logic)

plot_data <- df %>%

rownames_to_column("row_var") %>% # Add column: reference interventions (rows)

pivot_longer(

cols = -row_var,

names_to = "col_var", # Add column: comparison interventions (columns)

values_to = "value" # Original values: "RR (95%CI)" string

) %>%

mutate(

# 3.1 Extract RR value (point estimate): supports positive numbers (RR has no negative values, characteristic of dichotomous data)

rr_value = as.numeric(str_extract(value, "\\d+\\.?\\d*")), # Match "1.23" or "0.89" format

# 3.2 Extract 95% confidence interval (CI): split upper and lower limits

ci_raw = str_extract(value, "\\((.*?)\\)"), # Extract "(0.72,1.05)" format

ci_clean = gsub("[()]", "", ci_raw), # Remove parentheses→"0.72,1.05"

ci_lower = as.numeric(word(ci_clean, 1, sep = ",")), # CI lower limit (e.g., 0.72)

ci_upper = as.numeric(word(ci_clean, 2, sep = ",")), # CI upper limit (e.g., 1.05)

# 3.3 Dichotomous RR value significance judgment (core logic modification)

# Rule: CI not containing 1 is significant (RR≠1, intervention differs from reference); containing 1 is not significant

is_significant = case_when(

row_var == col_var ~ FALSE, # Diagonal (same intervention): no comparison, not significant

is.na(ci_lower) | is.na(ci_upper) ~ FALSE, # CI missing: not significant

ci_upper < 1 | ci_lower > 1 ~ TRUE, # CI does not contain 1: significant (mark with *)

TRUE ~ FALSE # CI contains 1: not significant

),

# 3.4 Generate display text (annotate RR value and CI, add * for significant results)

display_text = case_when(

row_var == col_var ~ row_var, # Diagonal: only display intervention name

is_significant ~ sprintf("%.2f*\n(%.2f, %.2f)", # Significant: RR value** + CI

rr_value, ci_lower, ci_upper),

TRUE ~ sprintf("%.2f\n(%.2f, %.2f)", # Not significant: only RR value + CI

rr_value, ci_lower, ci_upper)

)

)

# 4. Create dichotomous RR value heatmap (visual design adapted to RR statistical significance)

tiff("league_table_heatmap_RR.tiff", width = 18, height = 10, units = "cm", res = 300)

# Dynamically adjust text size: more interventions, smaller text (avoid overlap)

text_size <- ifelse(length(unique(plot_data$row_var)) > 5, 2.5, 3)

heatmap_plot <- ggplot(plot_data, aes(x = col_var, y = row_var, fill = rr_value)) +

# Heatmap main body: white borders separate cells

geom_tile(color = "white", size = 0.5) +

# Text annotation: RR value, CI, significance marker

geom_text(aes(label = display_text), size = text_size, color = "black") +

# Color mapping (core modification: centered at RR=1, matching dichotomous meaning)

scale_fill_gradient2(

low = "#FFE0B2", # Light orange: RR<1 (intervention reduces risk, good effect)

mid = "white", # White: RR=1 (no effect, reference baseline)

high = "#81D4FA", # Light blue: RR>1 (intervention increases risk, poor effect)

midpoint = 1, # Color center: RR=1 (dichotomous effect size no-effect point)

na.value = "gray90",# Missing values: gray

name = "RR", # Legend name: clearly indicates relative risk

guide = guide_colorbar(barwidth = 1, barheight = 10) # Legend dimensions

) +

# x-axis on top (following scientific heatmap convention: top columns=comparison interventions, left rows=reference interventions)

scale_x_discrete(position = "top") +

# Fixed aspect ratio (avoid cell stretching deformation)

coord_fixed(ratio = 0.5) +

# Chart annotation (adapted to dichotomous outcome, clearly indicating RR meaning)

labs(

x = "", # x-axis: interventions compared to reference

y = "", # y-axis: interventions as baseline

title = "Recurrence Rate (RR with 95%CI)" # Title: outcome + effect size type

) +

# Theme optimization (concise, scientific style)

theme_minimal() +

theme(

axis.text.x = element_text(angle = 0, hjust = 0.5, size = 8), # x-axis text centered horizontally

axis.text.y = element_text(size = 8), # y-axis text size

panel.grid = element_blank(), # Hide grid lines (avoid interfering with heatmap)

legend.position = "right", # Legend on right side

plot.title = element_text(hjust = 0.5, face = "bold", size = 12)# Title centered, bold

)

print(heatmap_plot)

dev.off()
