# Supplementary material for: Efficacy and safety of six Chinese patent medicines for elderly functional constipation: a network meta-analysis
Source: Front Med (Lausanne). 2026 Mar 31;13:1728217. doi: 10.3389/fmed.2026.1728217 (PMC13085306; doi:10.3389/fmed.2026.1728217)
Supplement: Supplementary file 1 [file Data_Sheet_1.zip › Supplementary_Material/Supplement Figure S1-S4.docx]

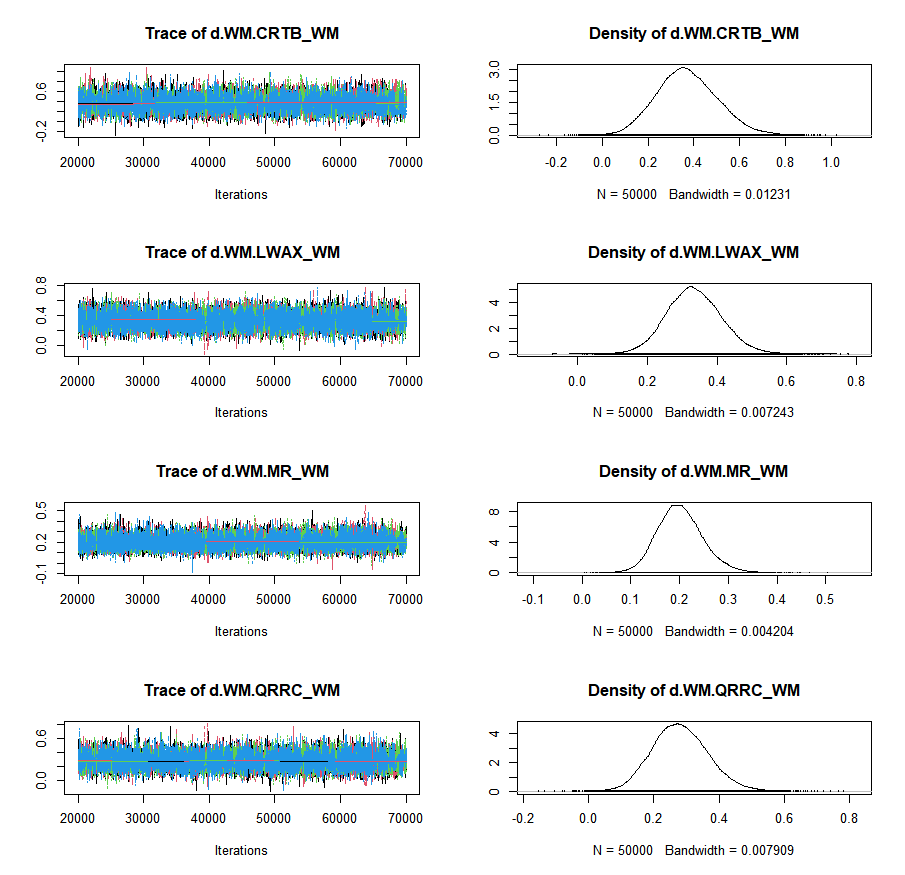

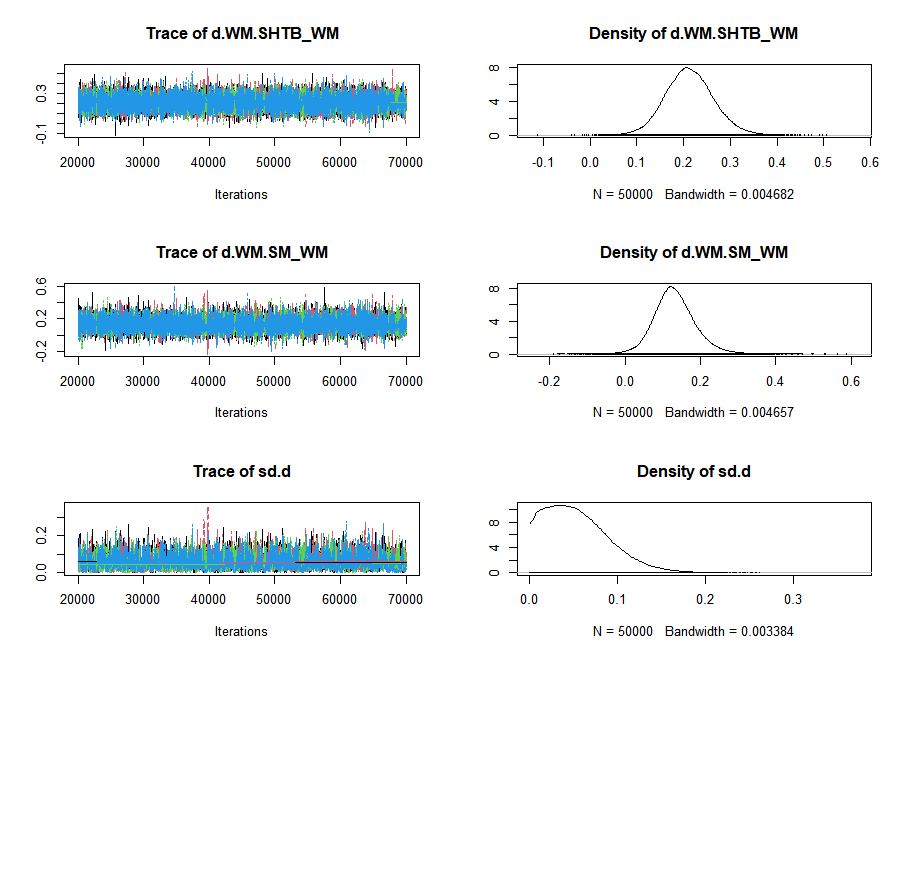

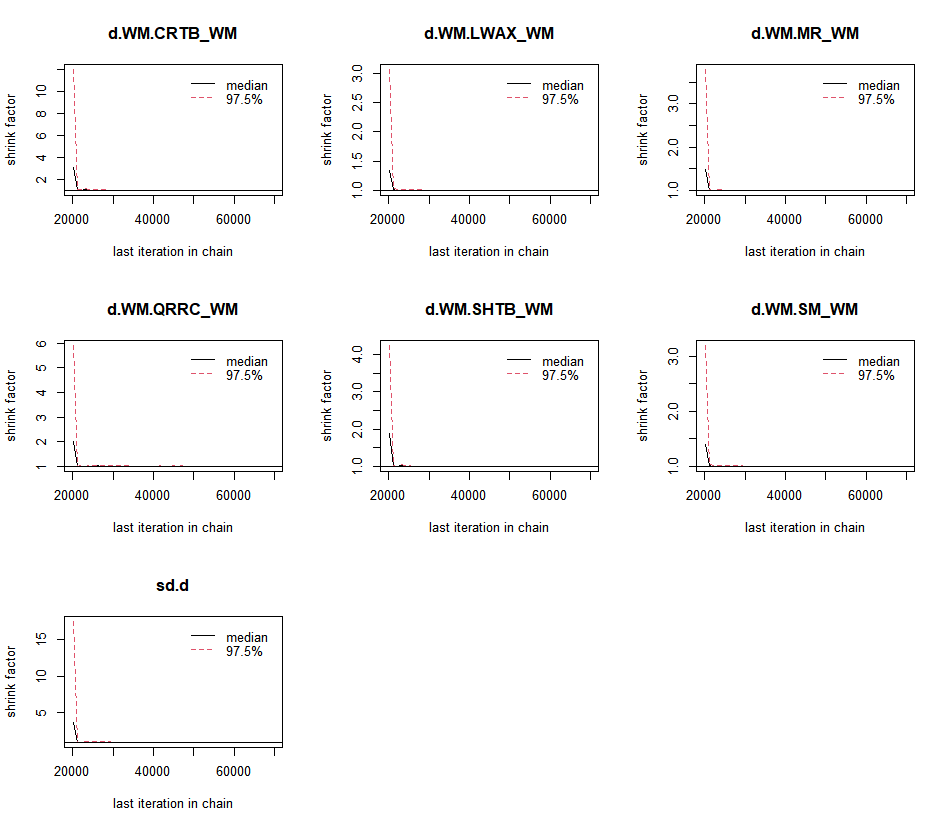


Supplementary Figure S1. MCMC trace plots and posterior density plots for the Bayesian NMA model of overall clinical effective rate.


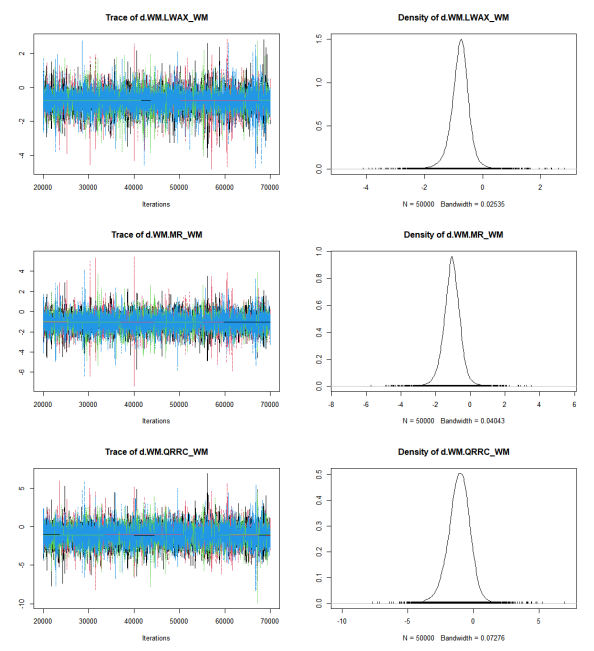

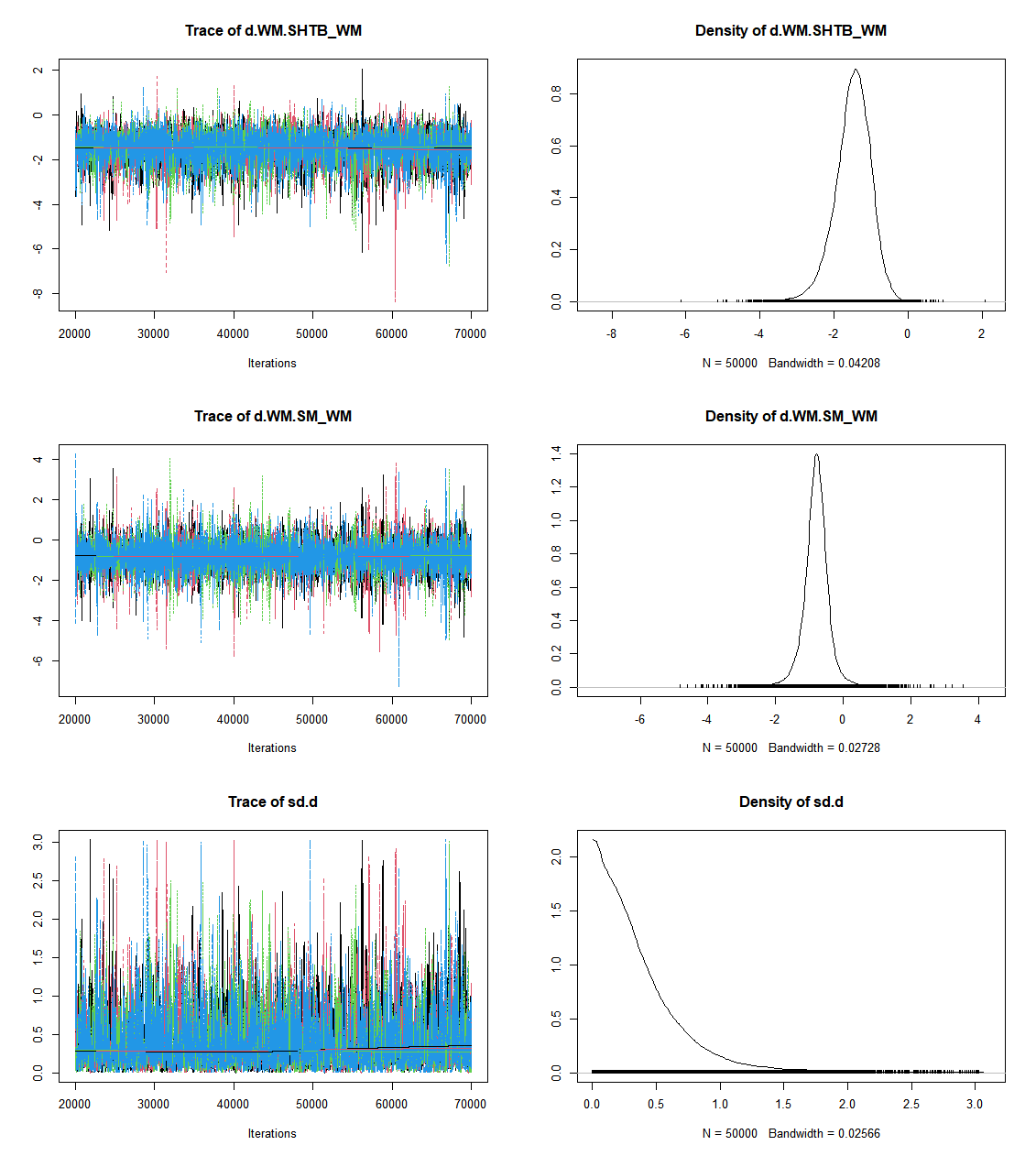

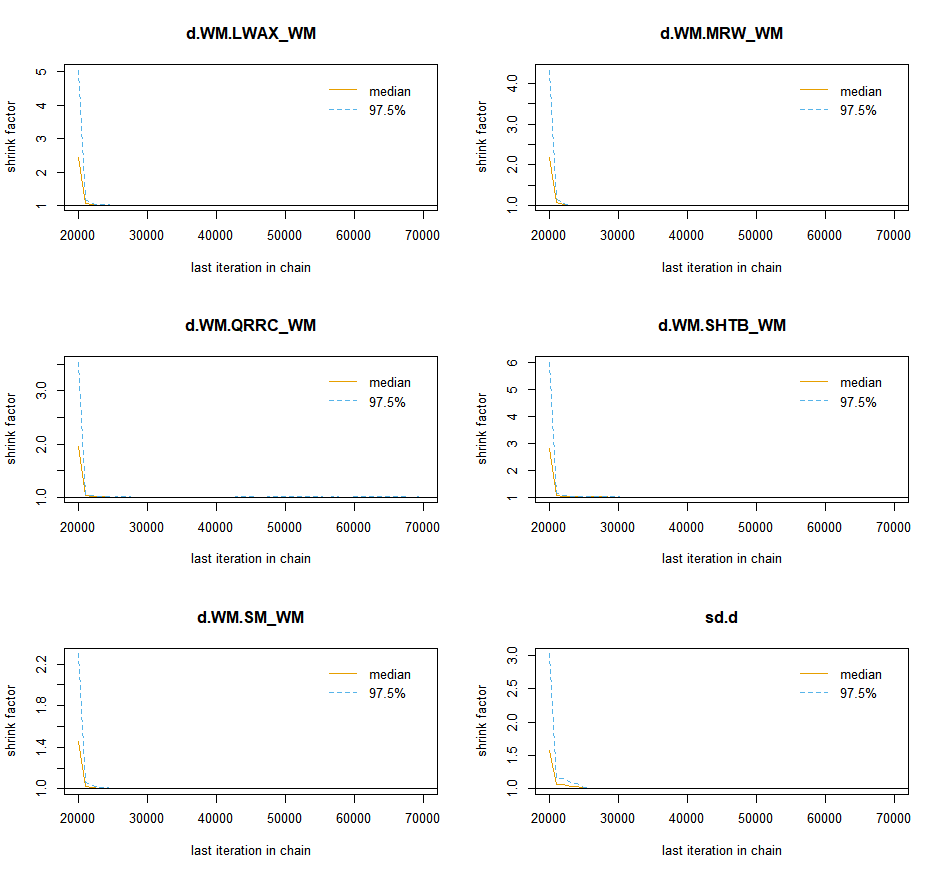


Supplementary Figure S2. MCMC trace plots and posterior density plots for the Bayesian NMA model of recurrence rate.


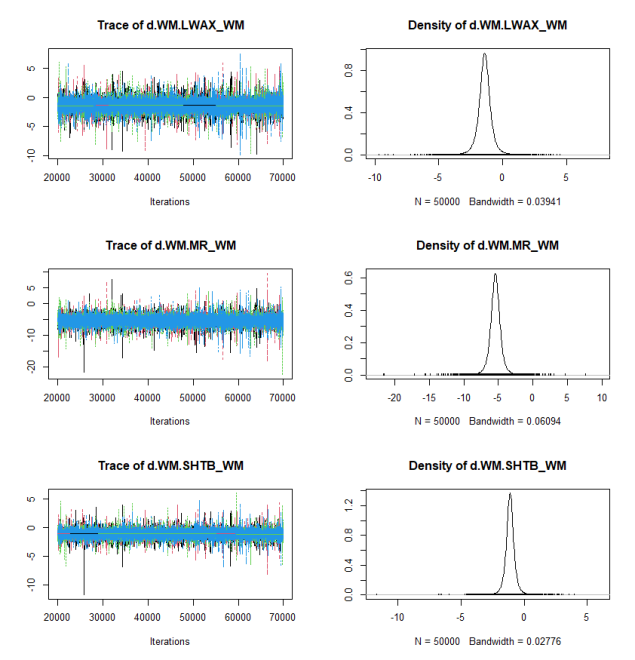

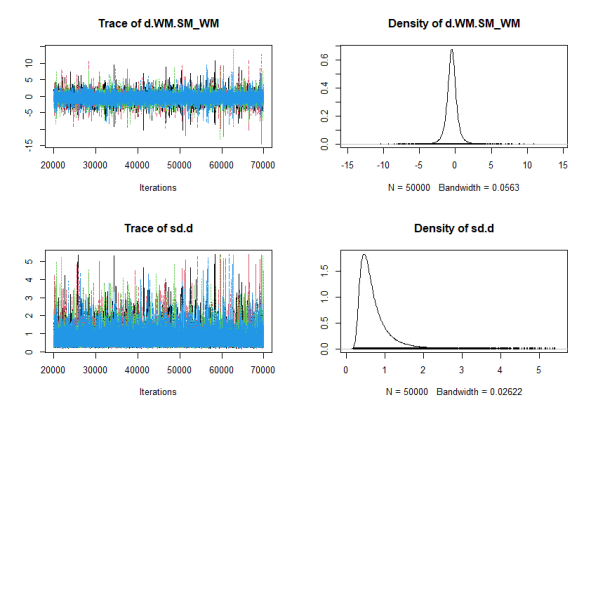

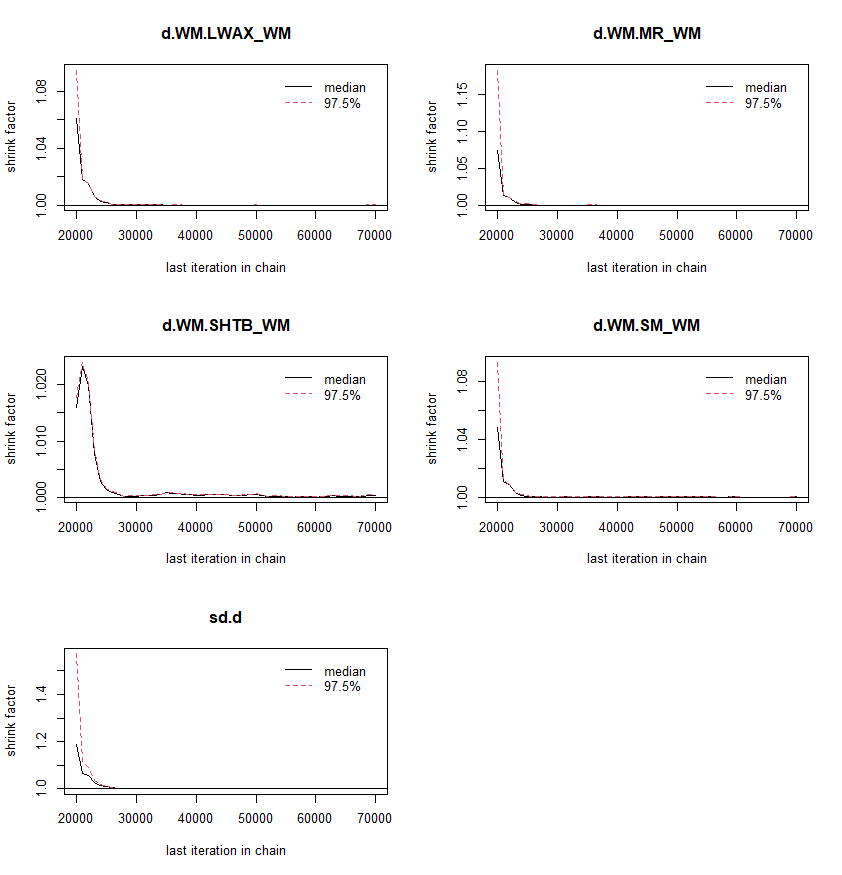


defecation difficulty


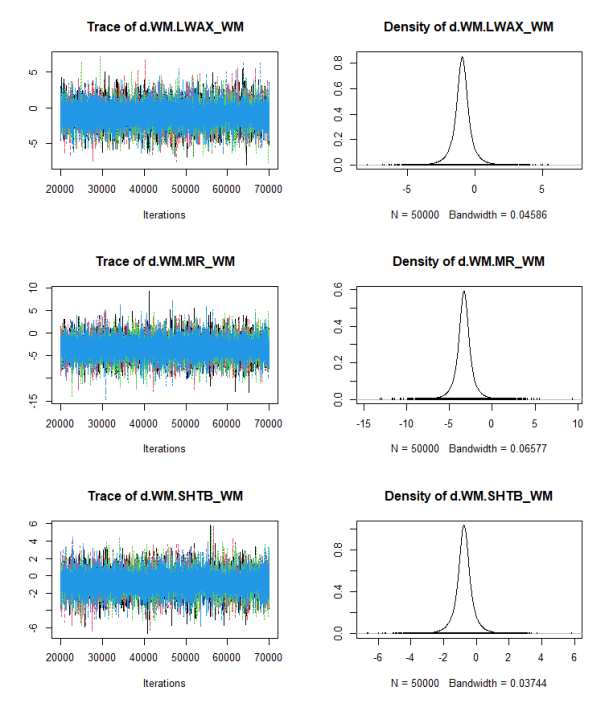

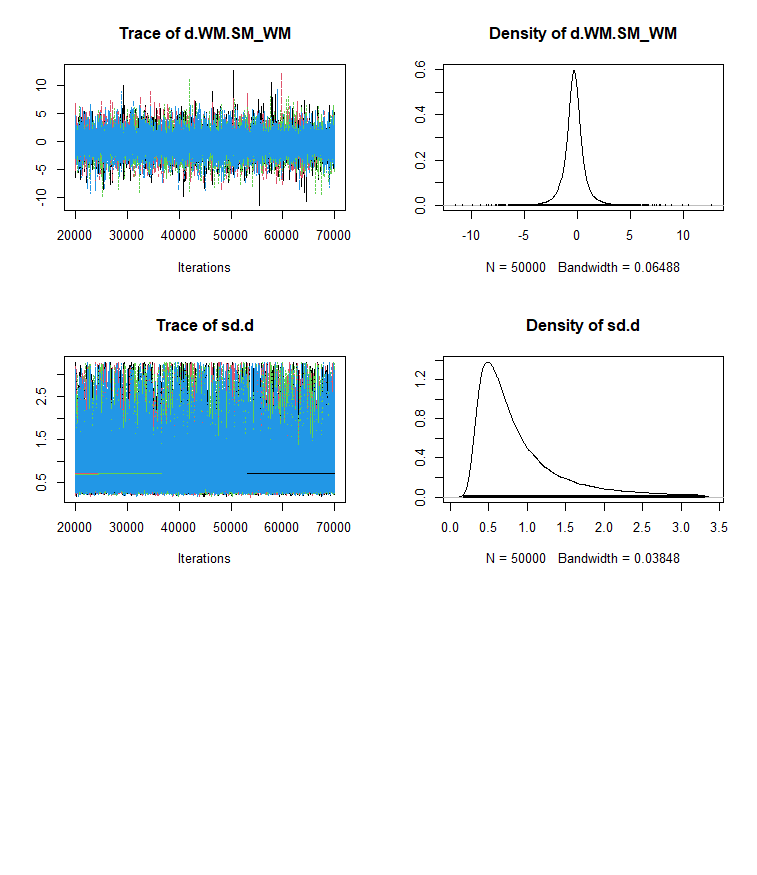

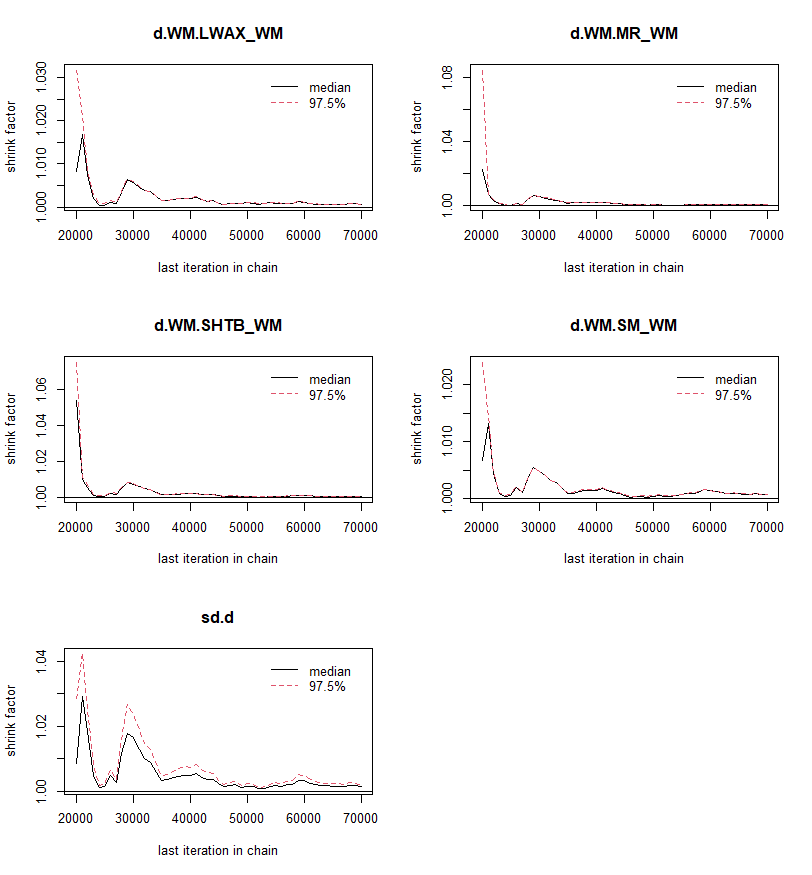


abdominal discomfort


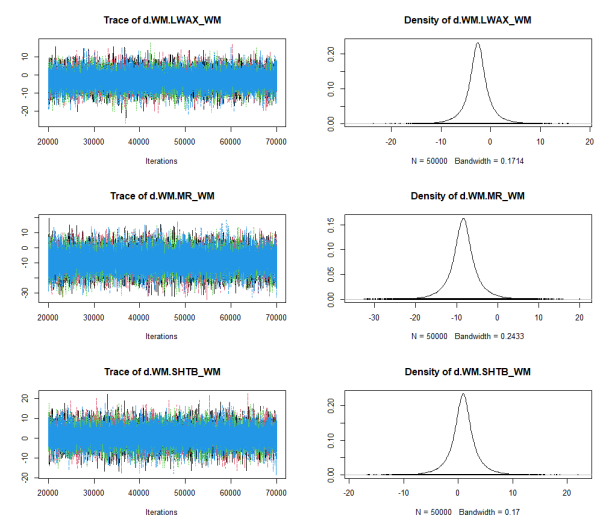

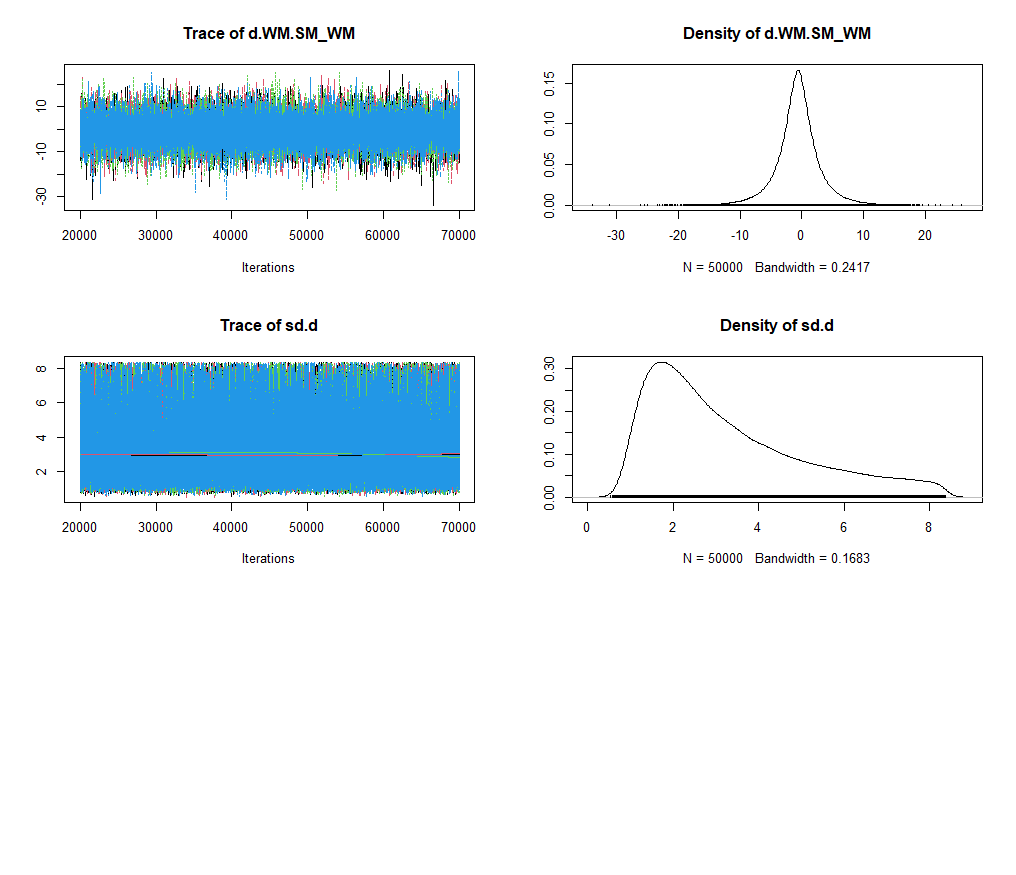

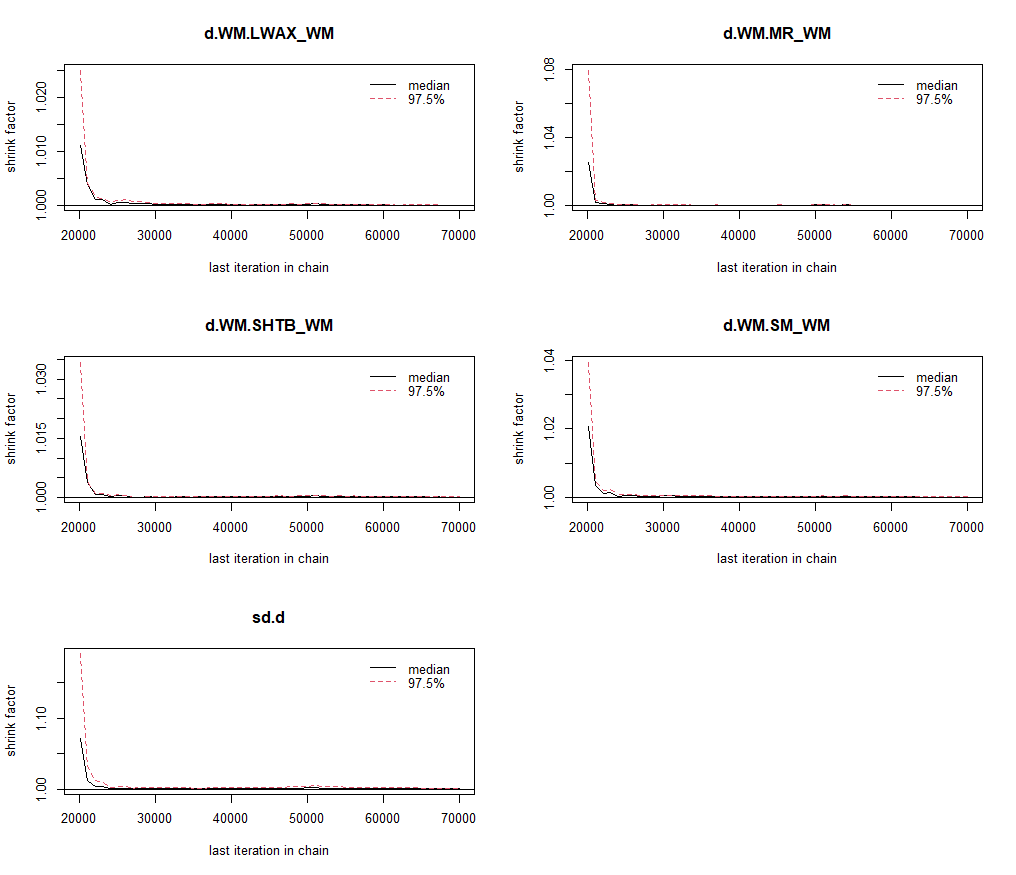


stool consistency


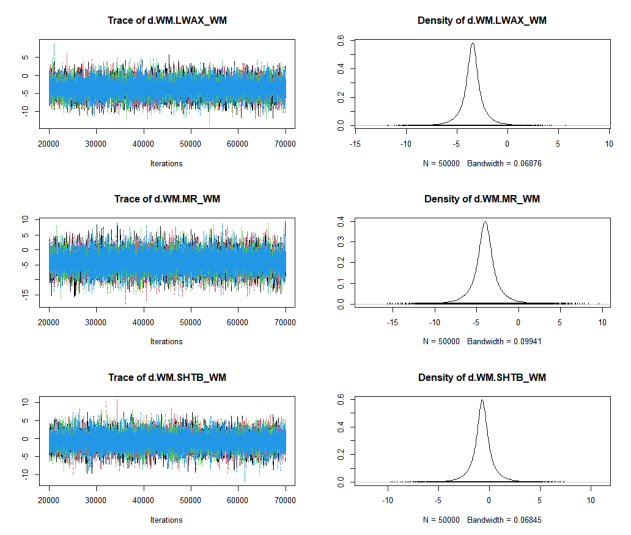

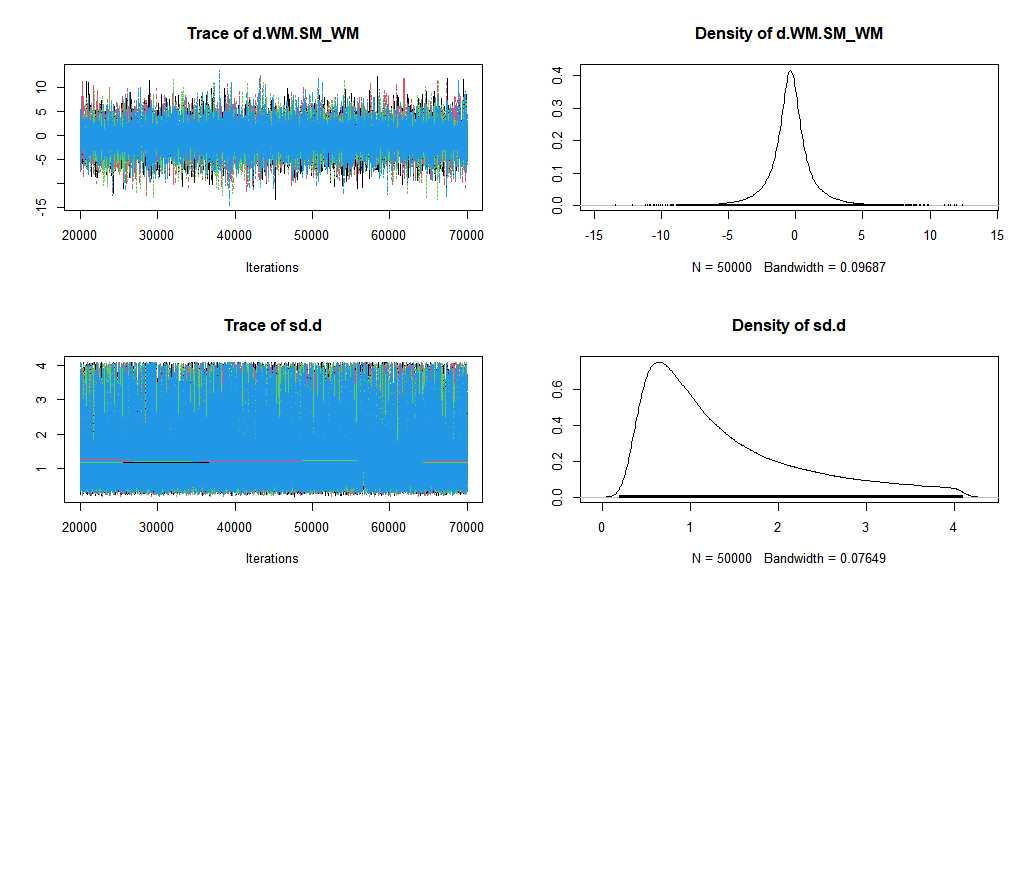

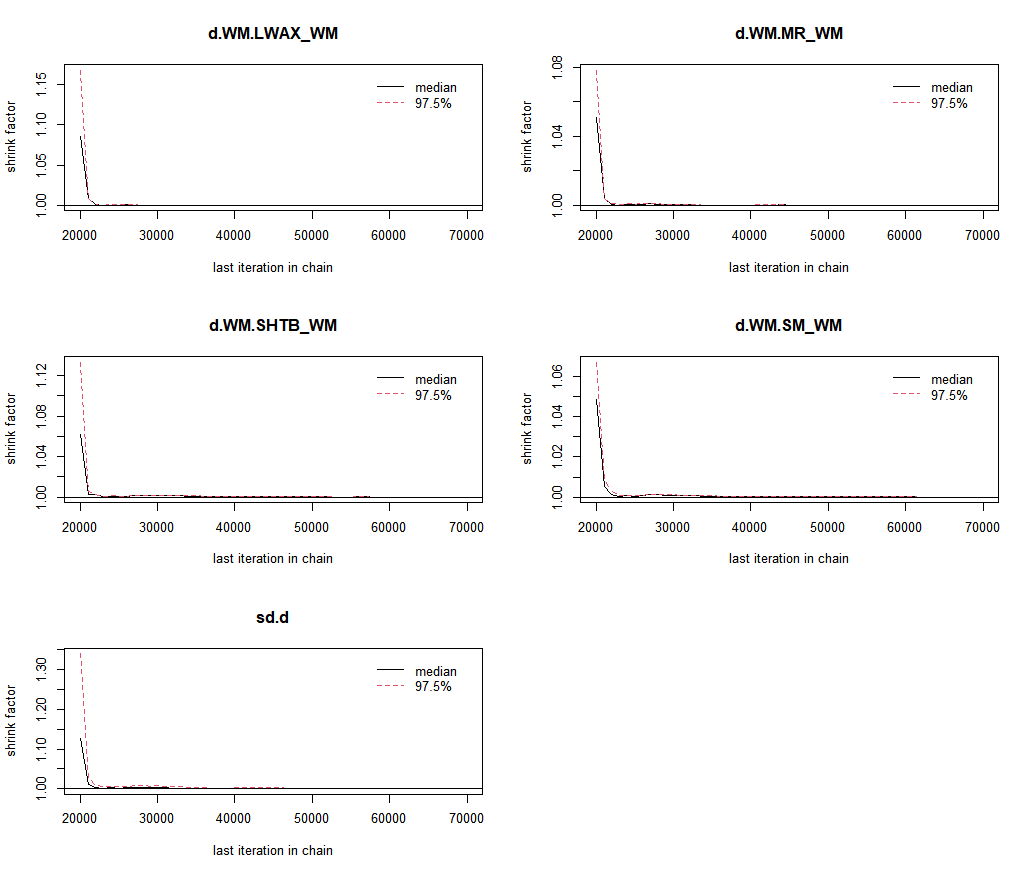


defecation frequency

Supplementary Figure S3. MCMC trace plots and posterior density plots for the Bayesian NMA models of symptom-score outcomes (defecation difficulty, abdominal discomfort, stool consistency, and defecation frequency).


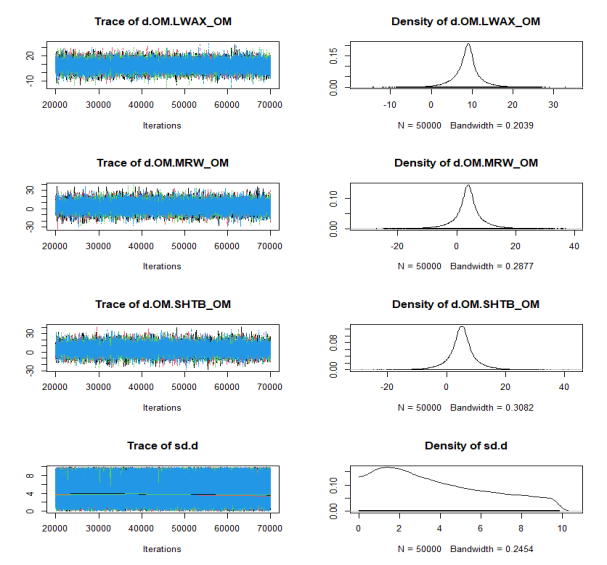

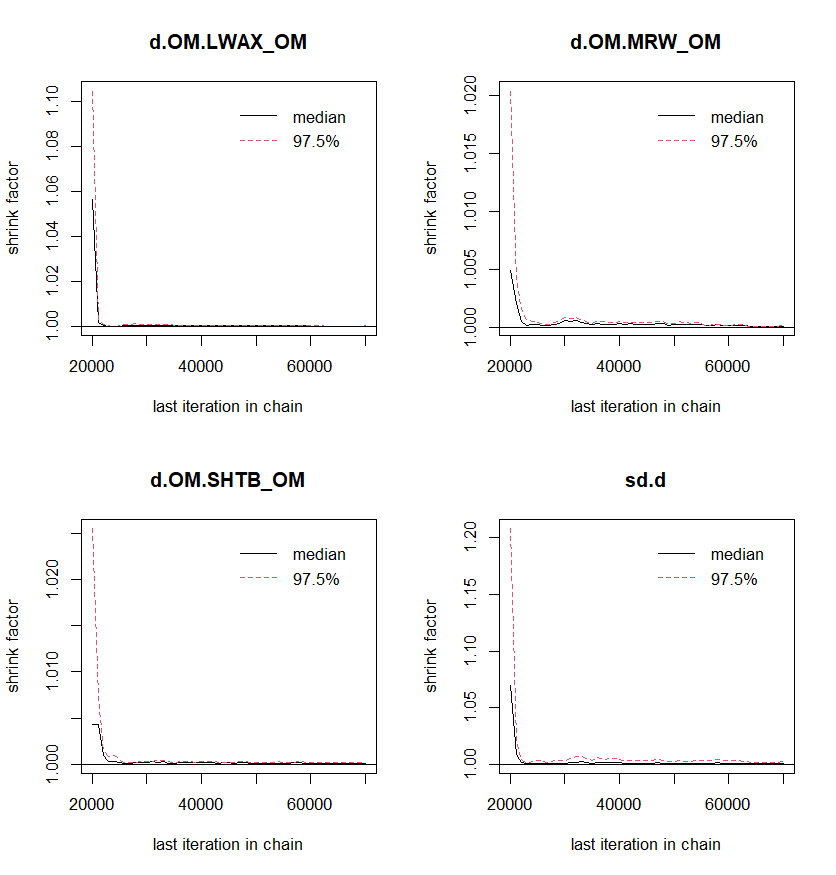

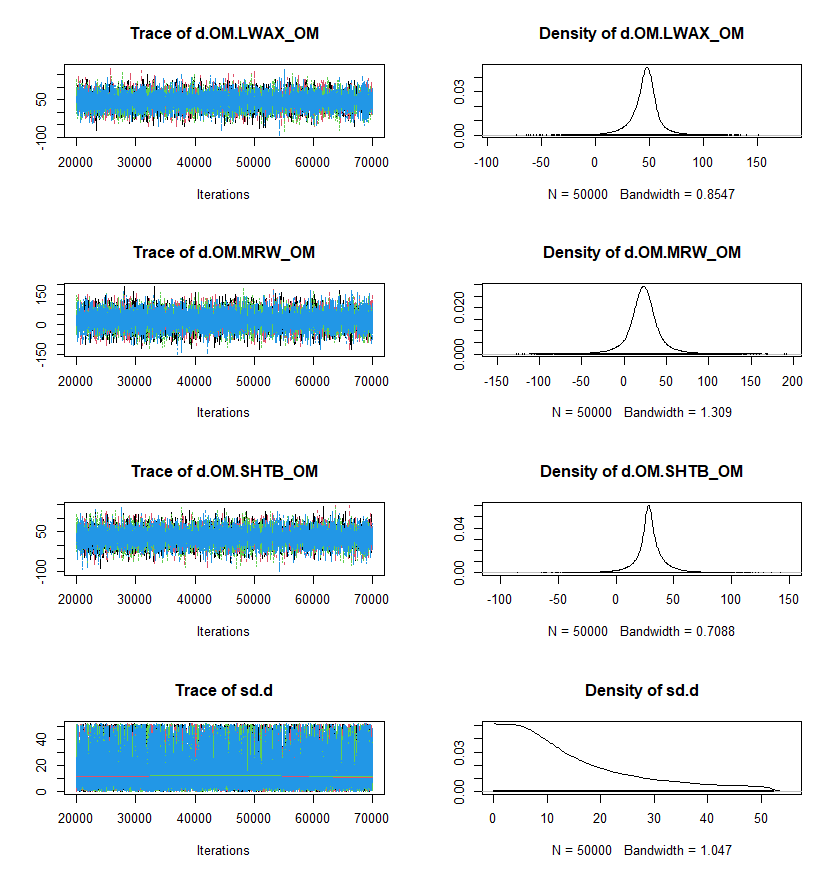

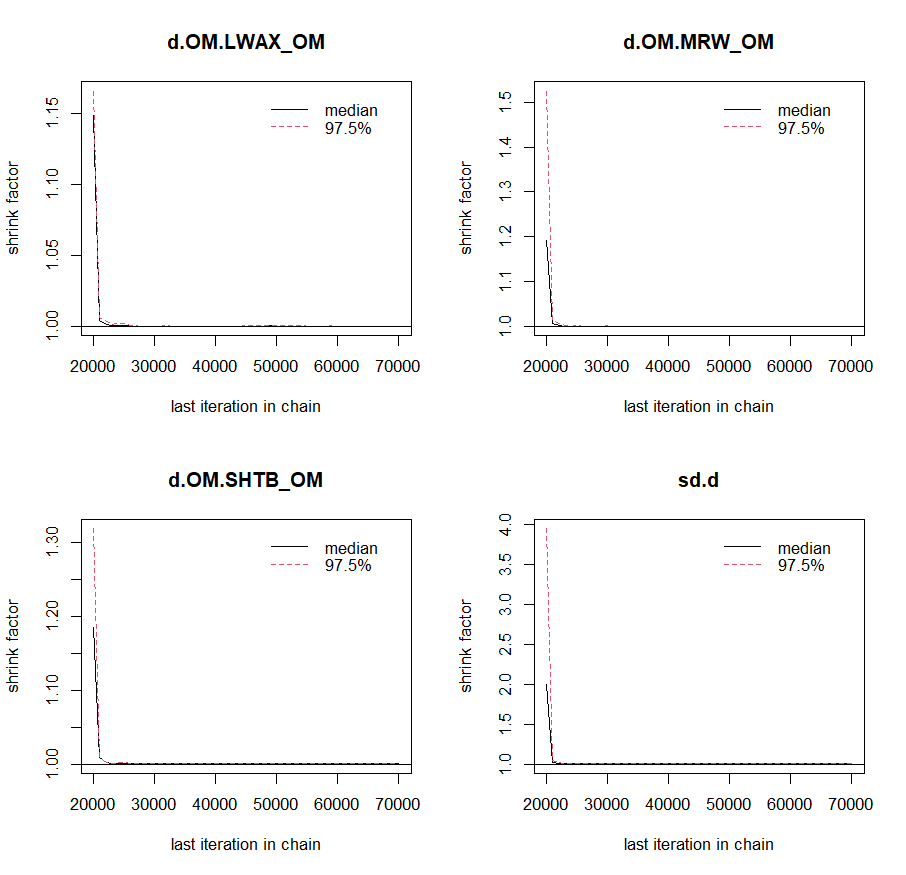


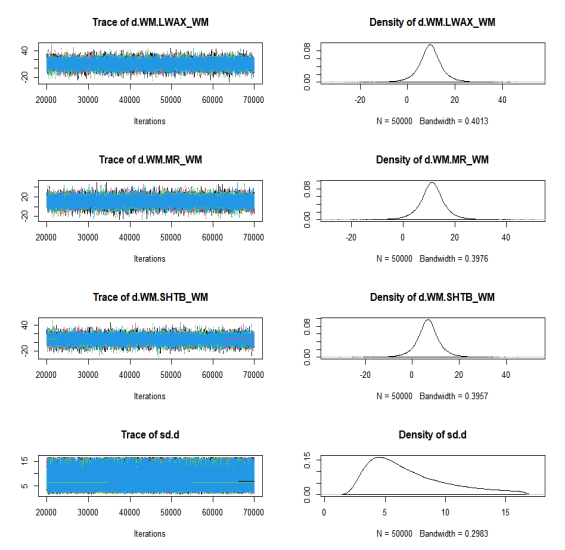

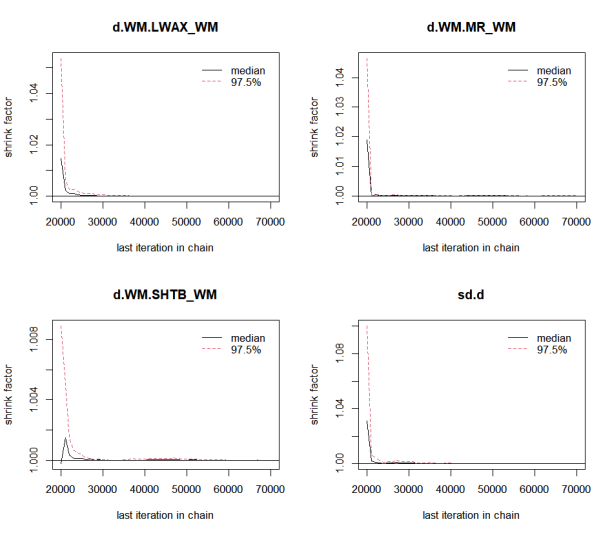


Supplementary Figure S4. MCMC trace plots and posterior density plots for the Bayesian NMA models of serum gastrointestinal hormones (GAS, MTL, and SP).
