# Supplementary material for: Efficacy and safety of six Chinese patent medicines for elderly functional constipation: a network meta-analysis
Source: Front Med (Lausanne). 2026 Mar 31;13:1728217. doi: 10.3389/fmed.2026.1728217 (PMC13085306; doi:10.3389/fmed.2026.1728217)
Supplement: Supplementary file 1 [file Data_Sheet_1.zip › Supplementary_Material/Supplement table 1.docx]

**Supplementary Table 1**

**Caption (EN): Trial-level definitions of overall clinical effective rate (investigator-defined composite endpoint) in included randomized controlled trials.**

| **Study First author year** | **Response categories** | **Operational criteria** | **Threshold formula** | **Trial numerator for effective rate** |
| --- | --- | --- | --- | --- |
| Zhu H., 2024 | Cured;  Improved;  Not cured | Cured: ≥1 BM within 2 d, stool moistened, smooth defecation, short-term no recurrence;  Improved: ≥1 BM within 3 d, stool moistened but not smooth;  Not cured: no improvement. | Total effective rate = (Cured + Improved)/Total×100% | Cured + Improved |
| Liu X., 2019 | Clinical cure; Markedly effective; Effective; Ineffective | Based on efficacy index (% change in symptom/sign score): cure ≥95%; markedly effective ≥70%;  effective ≥30%; otherwise ineffective. | Efficacy index = (post-score − pre-score)/pre-score; Effective rate=(Cure+Markedly+Effective)/Total×100% | Clinical cure + Markedly effective + Effective |
| Chen J., 2016 | Markedly effective; Effective; Ineffective | Marked: formed/soft stool, smooth, ~1/day;  Effective: formed but slightly hard, ~1 per 1–2 d;  Ineffective: still dry/hard, difficult; frequency not improved. | Total effective rate = (Markedly effective + Effective)/Total×100% | Markedly effective + Effective |
| Li Y., 2016 | Cured;  Markedly effective; Effective; Ineffective | Symptom score improvement %: cured = symptoms disappeared; markedly effective ≥80%;  effective 50–<80%;  ineffective <50%. | Improvement% = (pre-score − post-score)/pre-score ×100%; Total effective rate=(Cured+Markedly effective +Effective)/Total×100% | Cured + Markedly effective + Effective |
| Zhang A., 2014 | Significant; Effective; Ineffective | Efficacy index: significant ≥75%; effective 25–<75%;  ineffective <25%. | Efficacy index = (pre total score − post total score)/pre total score ×100% | Significant + Effective |
| Li S., 2011 | Markedly effective; Effective; Ineffective | Efficacy index: markedly effective ≥75%;  effective 25–<75%;  ineffective <25%. | Efficacy index=(pre symptom score − post symptom score)/pre symptom score ×100% | Markedly effective + Effective |
| Guan H., 2009 | Markedly effective; Effective; Ineffective | Marked: (i) frequency normal (1 per 1–2 d), (ii) normal formed/soft stool, (iii) smooth defecation without discomfort;  Effective: meets 1–2 items; Ineffective: no obvious improvement. | Total effective rate= Markedly effective /Total ×100% | Markedly effective |
| Mo W., 2021 | Markedly effective; Effective; Ineffective | Marked: 1/day, effortless, normal stool (formed soft, no hard); Effective: >3/week and stool from hard→soft;  Ineffective: otherwise. | Total effective rate=( Markedly effective +Effective)/Total ×100% | Markedly effective + Effective |
| Wang M., 2021 | Cured;  Improved; Ineffective | Cured: normal BM begins within 0.5 month, 1–2/day, soft, smooth, symptoms resolved, no recurrence after stopping;  Improved: normal BM begins within 0.5–1 month, 1–2/day, soft but slight difficulty;  Ineffective: still abnormal after 1 month. | Total effective rate = (Cured + Improved)/ Total ×100% | Cured + Improved |
| Ma J., 2018 | Cured;  Improved;  Not cured | Cured: BM within 2 d, soft, smooth; Improved: BM within 3 d, stool moistened, less smooth but improved; Not cured: no/limited improvement. | Total effective rate=(Cured+Improved)/Total×100% | Cured + Improved |
| Gao S., 2023 | Cured;  Markedly effective; Effective; Ineffective | Composite using spontaneous BM frequency + stool/effort + symptoms + first defecation time: cure ≥2/d and first BM ≤12h;  marked: 3/week–<2/d, first BM 12h–1d;  effective ≤2/week, occasional laxative, first BM 1–2d;  ineffective >2d and no improvement/worse. | Total effective rate=(Cured+Markedly effective+Effective)/Total ×100% | Cured + Markedly effective + Effective |
| Gu Z., 2022 | Markedly effective; Effective;  Ineffective | Marked: BM within 2 d, stool soft/moist, smooth, no distension; Effective: BM within 3 d, stool softer, shorter defecation time, distension improved;  Ineffective: no improvement. | Total effective rate = (Marked effective + Effective)/Total×100% | Markedly effective + Effective |
| Tan L., 2021 | Cured;  Markedly effective; Effective; Ineffective | Cured: >3/week, normal stool, all symptoms gone;  Marked: stool basically normal, interval ≤3 d, symptoms markedly reduced;  Effective: interval shortened by 1 d + stool improved;  Ineffective: no improvement. | Total effective rate=(Cured+Markedly effective +Effective)/Total ×100% | Cured + Markedly effective + Effective |
| Yuan B., 2021 | Markedly effective; Progress; Ineffective | Marked: frequency normal (1–2/d), soft stool, easy;  Progress: 1–2 per 3 d, stool softer, mild obstruction;  Ineffective: no improvement/worse. | Total effective rate = (Markedly effective + Progress)/Total×100% | Markedly effective + Progress |
| Zhou J., 2019 | Markedly effective; Effective; Ineffective | Marked: BM within 2 d, stool moist/soft, smooth, no distension; Effective: BM within 3 d, stool softer, shorter time, distension improved; Ineffective: no improvement. | Total effective rate = (Markedly effective + Effective)/Total×100% | Markedly effective + Effective |
| Zheng L., 2023 | Markedly effective; Effective; Ineffective | Marked: ≥1/day, soft/no hard, effortless, symptoms (abdominal pain/distension etc.) disappear; Effective: frequency increased, stool improved, symptoms improved; Ineffective: not meeting above. | Total effective rate = (Markedly effective + Effective)/Total×100% | Markedly effective + Effective |
| Jiang J., 2021 | Markedly effective; Effective; Ineffective | Constipation symptom score (stool form, frequency, difficulty, other discomfort). Improvement%=(pre−post)/pre×100%. Marked ≥80%; effective 60–80%; ineffective <60%. | Total effective rate=(Markedly effective +Effective)/Total ×100% | Markedly effective + Effective |
| Zhang J., 2012 | Markedly effective; Effective; Ineffective | Marked: within 4 weeks, ≥1/day formed soft stool, effortless, appetite ↑, abdominal pain/distension gone; Effective: ≥2 indicators improved; Ineffective: no obvious improvement. | Total effective rate=(Markedly effective +Effective)/Total ×100% | Markedly effective + Effective |
| Liu J., 2016 | Cured;  Improved; Ineffective | Cured: BM within 2 d, stool moistened, smooth, short-term no recurrence;  Improved: BM within 3 d, stool moistened, less smooth;  Ineffective: no improvement in constipation/associated symptoms. | Total effective rate=( Cured + Improved)/Total ×100% | Cured + Improved |
| Xu Z., 2014 | Cured;  Improved; Ineffective | Cured: BM within 2 d, smooth, soft stool, short-term no recurrence; Improved: BM within 3 d, stool lubricated, less smooth;  Ineffective: no improvement. | Total effective rate=( Cured + Improved)/Total ×100% | Cured + Improved |
| Shi Z., 2013 | Markedly effective; Effective; Ineffective | Marked: 1/d, Bristol 4–6, defecation difficulty score=0;  Effective: >3/week, Bristol 2–3, difficulty=1;  Ineffective: <3/week, Bristol 1–2, difficulty=2–3. | Total effective rate=(Markedly effective +Effective)/Total ×100% | Markedly effective + Effective |
| Gu T., 2015 | Markedly effective; Effective; Ineffective | Marked: 1–2/day formed or loose stool, no discomfort;  Effective: 1 formed stool every 2–3 days;  Ineffective: no improvement in stool frequency or stool consistency after treatment, or symptoms remained unchanged/worsened. | Total effective rate=(Markedly effective +Effective)/Total ×100% | Markedly effective + Effective |
| Fu S., 2012 | Markedly effective; Effective; Ineffective | Marked: effortless and 1/d within 2 weeks, Bristol 4–6; Effective: >3/week, Bristol 2–3; Ineffective: <3/week, Bristol 1–2. | Total effective rate=(Markedly effective +Effective)/Total ×100% | Markedly effective + Effective |

Note: BM = bowel movement
