# Supplementary material for: Efficacy and safety of six Chinese patent medicines for elderly functional constipation: a network meta-analysis
Source: Front Med (Lausanne). 2026 Mar 31;13:1728217. doi: 10.3389/fmed.2026.1728217 (PMC13085306; doi:10.3389/fmed.2026.1728217)
Supplement: Supplementary file 1 [file Data_Sheet_1.zip › Supplementary_Material/Supplement table 2.docx]

**Supplementary Table 2**

**Caption (EN): Trial-level definitions and scoring anchors for constipation symptom-score outcomes (defecation difficulty, abdominal discomfort, stool consistency, and defecation frequency) in included trials.**

| **Study First author year** | **Defecation difficulty score** | **Stool consistency score** | **Defecation frequency score** | **abdominal discomfort symptom scores** |
| --- | --- | --- | --- | --- |
| Liu X. 2019 | Defecation difficulty score: none (0), occasional (1), sometimes (2), frequent (3). | Stool consistency (Bristol): Types IV-VII=0, Type III=1, Type II=2, Type I=3 (higher=worse constipation). | Defecation interval: 1-2 d=0, 3 d=1, 4-5 d=2, >5 d=3. | Abdominal discomfort score: none (0), occasional (1), sometimes (2), frequent (3). |
| Mo W. 2021 | Defecation difficulty score: none (0), occasional (1), sometimes (2), frequent (3). | Stool consistency (Bristol): Types IV-VII=0, Type III=1, Type II=2, Type I=3 (higher=worse constipation). | Defecation interval: 1-2 d=0, 3 d=1, 4-5 d=2, >5 d=3. | Abdominal discomfort score: none (0), occasional (1), sometimes (2), frequent (3). |
| Gu Z. 2022 | Assessed (defecation difficulty), but the scoring instrument/scale range/anchors were not reported (NR). | - | Assessed (spontaneous bowel movement frequency), but numeric scoring criteria were not reported (NR). | Assessed (abdominal discomfort); however, the scoring criteria were not reported (NR). |
| Tan L. 2021 | Defecation difficulty score: none (0), occasional (1), sometimes (2), frequent (3). | Bristol stool form scale 1-7; each level scored 1-7 points (1=separate hard lumps; 7=watery). | - | - |
| Yuan B. 2021 | Defecation difficulty: none/mild/moderate/severe scored 0/1/2/3. | Stool consistency (Bristol): Type 1/2/3/4-7 scored 0/1/2/3. | - | Abdominal discomfort score: none/mild/moderate/severe scored 0/1/2/3. |
| Zhou J. 2019 | Defecation difficulty: 0=spontaneous; 1=needs effort; 2=great effort; 3=needs perianal massage/manual evacuation. | - | Spontaneous bowel movement frequency: 0=1/1-2 d; 1=1/3-4 d; 2=1/5-6 d; 3=1/≥1 week; 4=none. | Composite symptoms (abdominal pain, distension, incomplete evacuation): none=0; 1 symptom=1; 2 symptoms=2; 3 symptoms=3. |
| Zheng L. 2023 | Defecation difficulty: 0=spontaneous; 1=after effort; 2=very hard effort; 3=needs massage/manual assistance. | Stool form: 0=smooth sausage; 1=sausage with cracks; 2=lumpy; 3=pellets/nut-like hard balls. | Defecation frequency: 0=1/1-2 d; 1=1/2-4 d; 2=1/5-6 d; 3=1/≥7 d. | Composite symptoms (abdominal pain, distension, incomplete evacuation): none=0; 1 symptom=1; 2 symptoms=2; 3 symptoms=3. |
| Jiang J. 2021 | Defecation difficulty: 0=spontaneous; 1=after effort; 2=great effort; 3=needs massage/manual assistance. | Stool form: 0=smooth sausage; 1=sausage with cracks; 2=lumpy; 3=pellets/nut-like hard balls. | Defecation frequency: 0=1/1-2 d; 1=1/3-4 d; 2=1/5-6 d; 3=1/7 d. | Composite symptoms (abdominal pain, distension, incomplete evacuation): none=0; 1 symptom=1; 2 symptoms=2; 3 symptoms=3. |

Notes: NR = assessed/reported but scoring criteria were not reported. - = not assessed or not applicable.
