# Supplementary material for: Efficacy and safety of six Chinese patent medicines for elderly functional constipation: a network meta-analysis
Source: Front Med (Lausanne). 2026 Mar 31;13:1728217. doi: 10.3389/fmed.2026.1728217 (PMC13085306; doi:10.3389/fmed.2026.1728217)
Supplement: Supplementary file 1 [file Data_Sheet_1.zip › Supplementary_Material/Supplement table 3.docx]

Supplement Table 3

Caption (EN): Dosing, administration, and key precautions/contraindications of the included Chinese patent medicines (CPMs) as reported in trials and/or product information.

The detailed information of the components, indications, adverse reactions, contraindications, and precautions of these Chinese patent medicines

| **Medicines vocabularies** | **Component** | **Indication** | **Adverse Reaction** | **Taboo** | **Notice** |
| --- | --- | --- | --- | --- | --- |
| Maren-formula CPMs | Fire hemp seeds, bitter apricot kernels, rhubarb, fried bitter orange peel, ginger-processed magnolia bark, fried white peony root. The auxiliary ingredient is refined honey. | Moistens the intestines and promotes bowel movement.It is indicated for constipation due to intestinal heat and fluid deficiency, characterized by dry, hard stools that are difficult to pass and accompanied by abdominal fullness and discomfort. It is also used for habitual constipation presenting with the aforementioned pattern. | No obvious adverse reactions | remains unclear | 1. Maintain a bland diet during medication. Avoid alcohol and spicy foods. 2. Concomitant use with other tonifying/herbal supplements is not advised. 3. Patients with severe chronic conditions (e.g., hypertension, cardiac disease, liver or kidney dysfunction, diabetes) should use this product under medical supervision. 4. Special populations (children, pregnant or breastfeeding women, the elderly, and debilitated individuals) should use it under medical guidance. 5. Adhere strictly to the prescribed dosage and duration. Long-term use is not recommended. 6. Discontinue and consult a physician if symptoms persist after 3 days of administration. 7. This product is contraindicated in patients with a known allergy to any of its components. Caution is advised for individuals with a general allergic predisposition. 8. Do not use if the physical properties of the product (e.g., color, consistency) have altered. 9. Keep out of reach of children. When used in children, administration must be under direct adult supervision. |
| Qirong Runchang Oral Liquid | Astragalus (fried), Cistanche deserticola, Atractylodes macrocephala, Codonopsis pilosula, Rehmannia glutinosa, Scrophularia ningpoensis, Ophiopogon japonicus, Angelica sinensis, Polygonatum sibiricum (processed), Morus alba fruit, Sesamum indicum (black), Cannabis sativa seed, Prunus armeniaca kernel, Fructus Aurantii (fried with bran), Honey. | Tonify qi and nourish yin, strengthen the spleen and nourish the kidneys, moisten the intestines and promote bowel movements.For constipation arising from deficiency patterns due to both qi and yin deficiency, insufficiency of the spleen and kidney, and loss of moisture in the large intestine. | remains unclear | remains unclear | Contraindicated in cases of actual heat disease. Discontinue use during colds or fever. Use with caution in pregnant women. |
| Liuwei Anxiao Capsule | Saussurea costus, Rheum officinale, Zingiber officinale, Calcined Cinnabar, Terminalia chebula, Potash. | Strengthens the spleen and harmonizes the stomach, relieves food stagnation and eliminates accumulation, promotes blood circulation and alleviates pain. Indicated for stomach distension and fullness, indigestion, constipation,and dysmenorrhea. | remains unclear | Not suitable for children or pregnant women. | 1. Not suitable for patients with chronic illness-induced weakness and stomach pain. 2. Patients with hypertension, heart disease, kidney disease, or edema should take under a physician's guidance. 3. Follow the recommended dosage; elderly patients should take under a physician's guidance. 4. If symptoms do not improve after three days of use, seek medical attention promptly. 5. Consult a physician before long-term continuous use. 6. Do not use if allergic to this product; those with allergic constitutions should use with caution. 7. Do not use if the product's appearance has changed. 8. Keep out of reach of children. |
| Congrong Tongbian Oral Liquid | Cistanche deserticola, Polygonum multiflorum, Ziziphus jujuba (fried with wheat bran), honey. | Moistens the intestines and relieves constipation. For elderly constipation and postpartum constipation. | 1. Digestive System: Nausea, vomiting, stomach pain, bloating, stomach discomfort, acid reflux, abdominal pain, diarrhea, dark urine, etc. There have been reports of oral He Shou Wu and its compound preparations posing a risk of liver damage. 2. Nervous System: Headache, dizziness. 3. Skin and Appendages: Rash, itching. | 1. Contraindicated in patients with hepatic insufficiency. 2. Contraindicated in pregnant women. 3. Not recommended for patients with a personal history of liver injury associated with this product or its constituent drugs. | 1. Pregnant women should use with caution. Young and healthy individuals should not use this medication for constipation. 2. Discontinue immediately if loose stools occur during use. 3. This product contains Polygonum multiflorum. During treatment, monitor for clinical manifestations associated with liver damage. 4. If abnormal liver biochemical indicators or symptoms such as generalized fatigue, loss of appetite, aversion to greasy foods, nausea, dark urine, jaundice, or skin yellowing occur—which may indicate liver injury—or if pre-existing abnormal liver biochemical tests worsen, discontinue use immediately and seek medical attention. 5. Use with caution in patients with abnormal liver biochemical indicators or a history of liver disease. 6. Strictly adhere to the dosage and administration instructions in the package insert; do not exceed the recommended dose or use continuously for extended periods. 7. Avoid concomitant use with other hepatotoxic medications. 8. If symptoms do not improve after three days of treatment or if new symptoms develop, seek medical attention promptly. 9. Prolonged storage may cause minor precipitation that disperses upon shaking. Shake well before use; this does not affect efficacy. 10. 1 11. Contraindicated in patients with known hypersensitivity to this product; use with caution in those with allergic constitutions. 12. 1 13. Do not use if product appearance has changed. 14. 1 15. Children must use under adult supervision. 16. 1 17. Keep out of reach of children. |
| Simo Decoction Oral Liquid | Saussurea costus, Citrus aurantium peel, Areca catechu, Lindera root | Promotes smooth flow of qi and alleviates counterflow, eliminates food stagnation and relieves pain. Indicated for infants and young children with milk/food stagnation syndrome, presenting with abdominal distension, abdominal pain, restless crying, poor appetite, diarrhea or constipation; for middle-aged and elderly individuals with qi stagnation and food accumulation syndrome, presenting with epigastric and abdominal fullness, abdominal pain, and constipation; and for promoting gastrointestinal recovery after abdominal surgery. | Diarrhea, nausea, vomiting, abdominal pain, abdominal discomfort, dry mouth, rash, itching, dizziness, palpitations, hypersensitivity reactions, flushing, fever, fatigue, edema, chills, throat discomfort, etc. There have been isolated reports of abnormal liver function following administration. | 1. Do not use if allergic to this product or any of its ingredients. 2. Do not use during pregnancy. 3. Do not use in patients with intestinal obstruction, intestinal tumors, or following gastrointestinal surgery. | 1. For general surgical patients, take the first dose 12 hours after surgery, followed by a second dose 6 hours later. Subsequent doses should be taken as directed or as prescribed by your doctor. 2. During winter, warm the bottle in lukewarm water for 5-8 minutes before taking the medication. 3. Minor sedimentation in the solution is normal. Shake well before use to ensure efficacy. 4. Use with caution in diabetic patients. 5. Use with caution in individuals with allergic constitutions. 6. Adhere strictly to the indicated therapeutic functions, dosage, and administration. Discontinue use and seek immediate medical attention if adverse reactions occur during treatment. |
| Shouhui Tongbian Capsule | Polygonum multiflorum, Aloe vera, Cassia seeds, Ginseng, Goji berries, Donkey-hide gelatin, Zizyphus jujuba fruit, Atractylodes macrocephala. | Nourishes yin and boosts qi, purges turbidity and relieves constipation. Indicated for functional constipation diagnosed in Traditional Chinese Medicine as qi and yin deficiency with internal accumulation of pathogenic toxins, presenting with constipation, abdominal distension, dry mouth and throat, fatigue and lassitude, five-heart heat, red or pale tongue with white or greasy coating, and deep-fine or slippery-rapid pulse. | Mild abdominal pain and diarrhea may occur, which typically resolve upon reducing or discontinuing the medication. | 1. Contraindicated in patients with hepatic insufficiency. 2. Contraindicated in patients with a history of liver injury caused by He Shou Wu or preparations containing He Shou Wu. 3. Contraindicated in pregnant and lactating women. | 1. Administer according to prescribed dosage, frequency, and duration. Avoid exceeding recommended doses or prolonged use. 2. Monitor liver biochemical indicators during treatment. 3. Use with caution in patients with abnormal liver function or history of liver disease. 4. Use with caution in patients with a family history of liver injury caused by He Shou Wu or He Shou Wu-containing preparations. 5. Avoid concomitant use with hepatotoxic medications. 6. One patient in clinical trials exhibited decreased total white blood cell count; causality with the investigational drug remains undetermined. 7. In Phase II clinical trials, the high-dose group (3 capsules three times daily) reported one case each of mild liver function abnormalities and ECG abnormalities; causality with the investigational drug remains undetermined. |

*Note: This document is for formatting and reference only. For clinical use, please consult the official package insert.*
