# Supplementary figures and images for: Efficacy and safety of six Chinese patent medicines for elderly functional constipation: a network meta-analysis
Source: Front Med (Lausanne). 2026 Mar 31;13:1728217. doi: 10.3389/fmed.2026.1728217 (PMC13085306; doi:10.3389/fmed.2026.1728217)

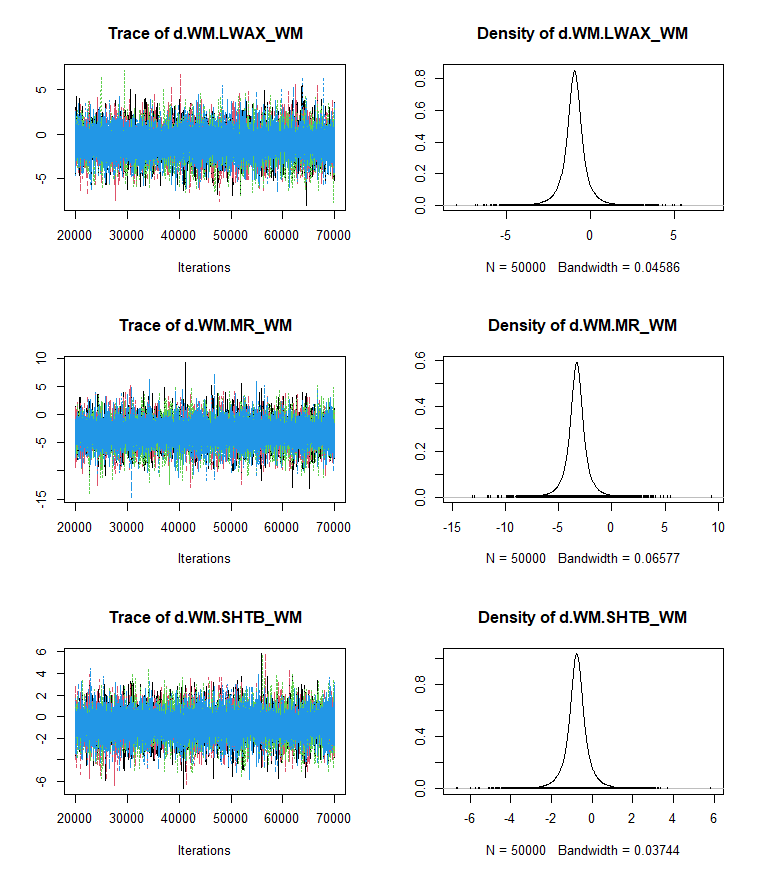

Supplement: Supplementary file 1 [file Data_Sheet_1.zip › Supplementary_Material/Supplementary Figure/abdominal discomfort symptom score/Figure 1.tiff]

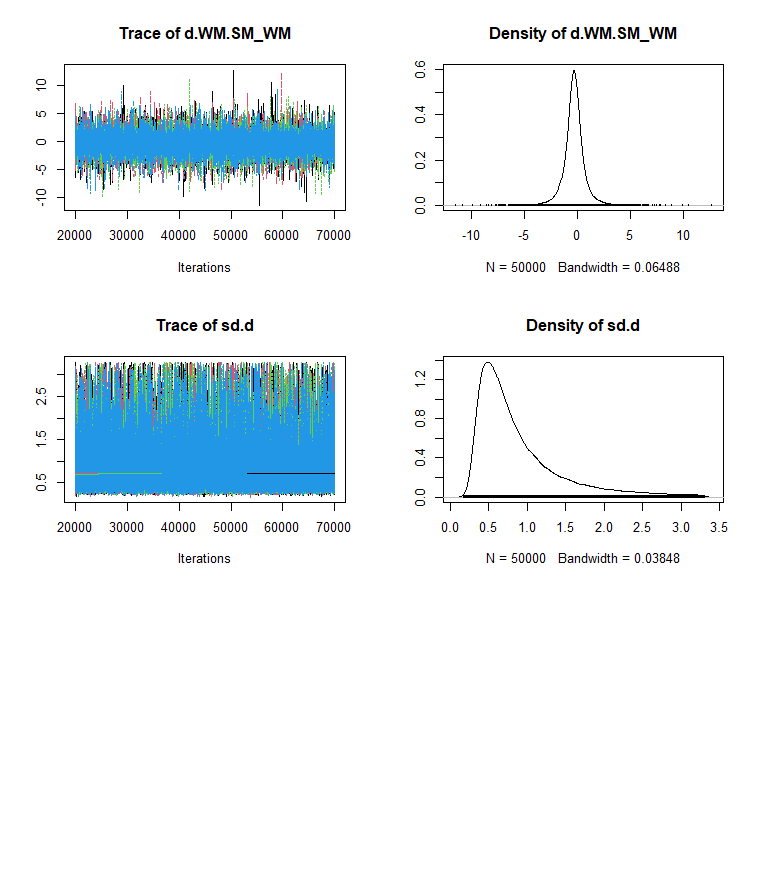

Supplement: Supplementary file 1 [file Data_Sheet_1.zip › Supplementary_Material/Supplementary Figure/abdominal discomfort symptom score/Figure 2.tiff]

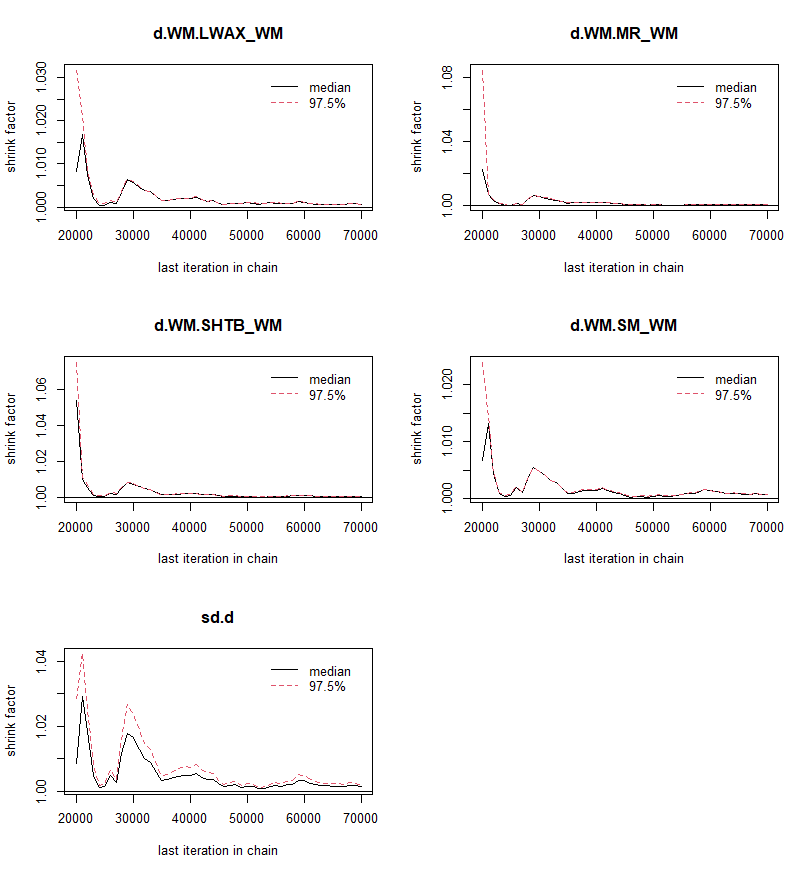

Supplement: Supplementary file 1 [file Data_Sheet_1.zip › Supplementary_Material/Supplementary Figure/abdominal discomfort symptom score/Figure 3.tiff]

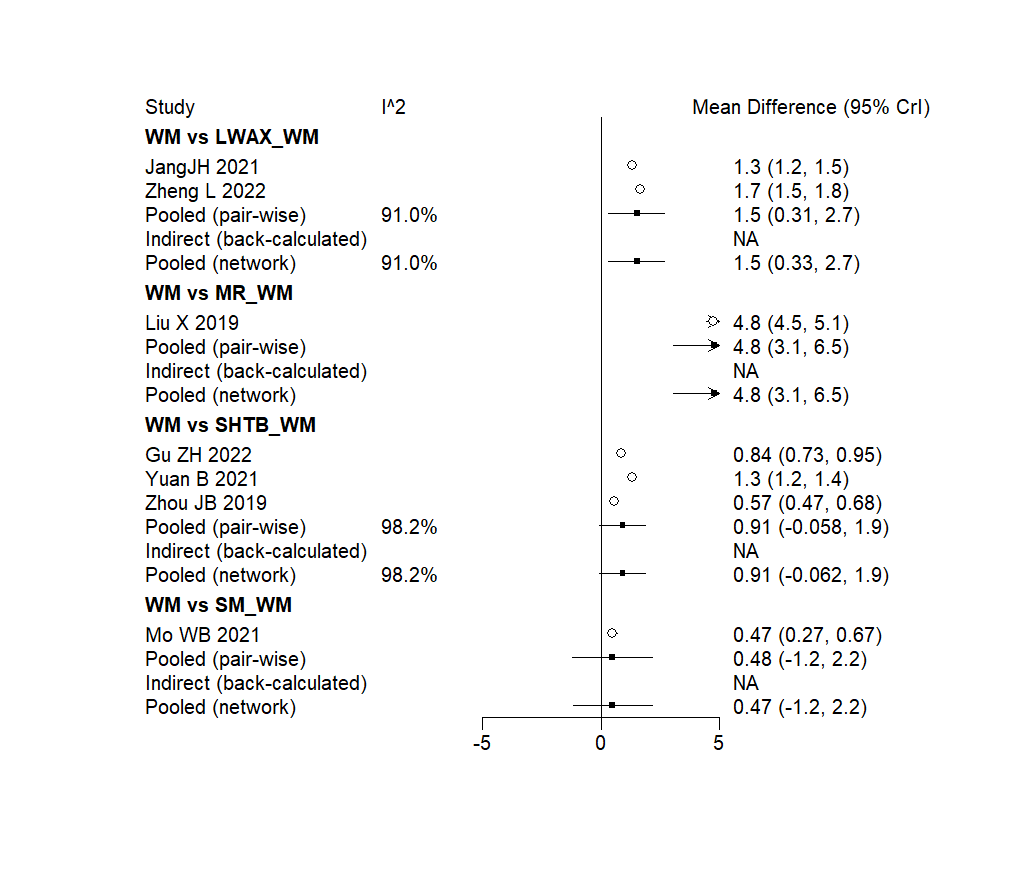

Supplement: Supplementary file 1 [file Data_Sheet_1.zip › Supplementary_Material/Supplementary Figure/abdominal discomfort symptom score/Figure 4.tiff]

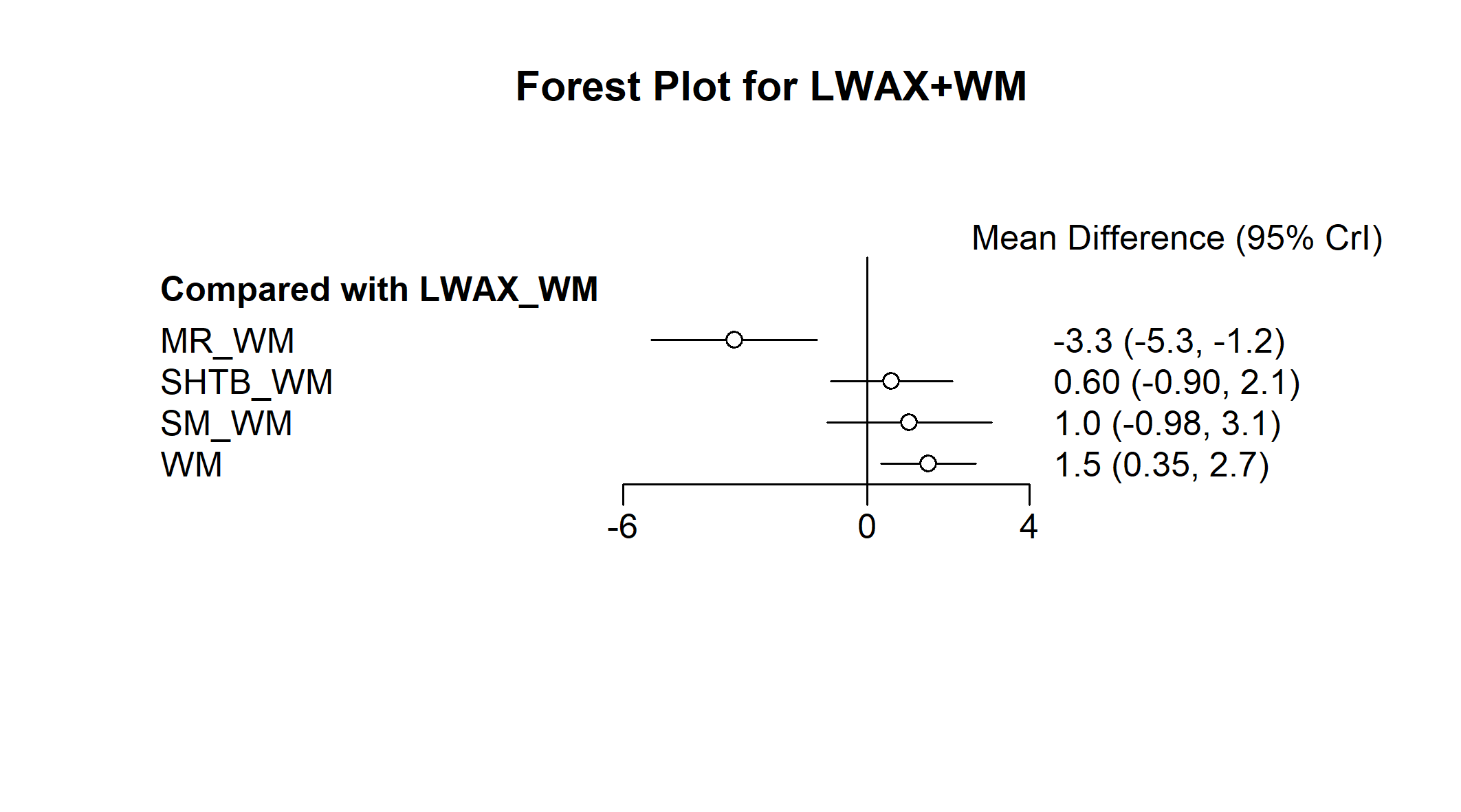

Supplement: Supplementary file 1 [file Data_Sheet_1.zip › Supplementary_Material/Supplementary Figure/abdominal discomfort symptom score/Figure 5.tiff]

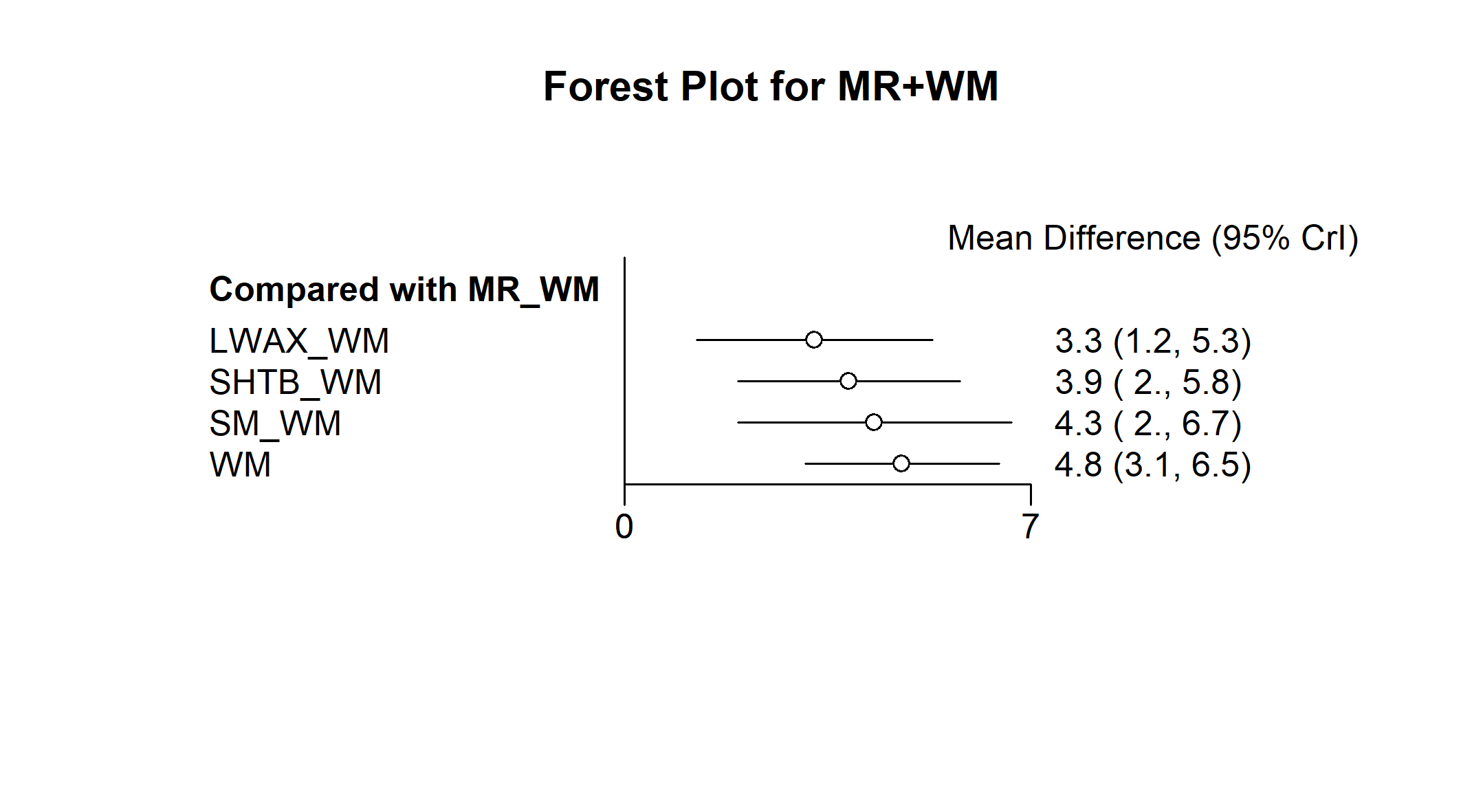

Supplement: Supplementary file 1 [file Data_Sheet_1.zip › Supplementary_Material/Supplementary Figure/abdominal discomfort symptom score/Figure 6.tiff]

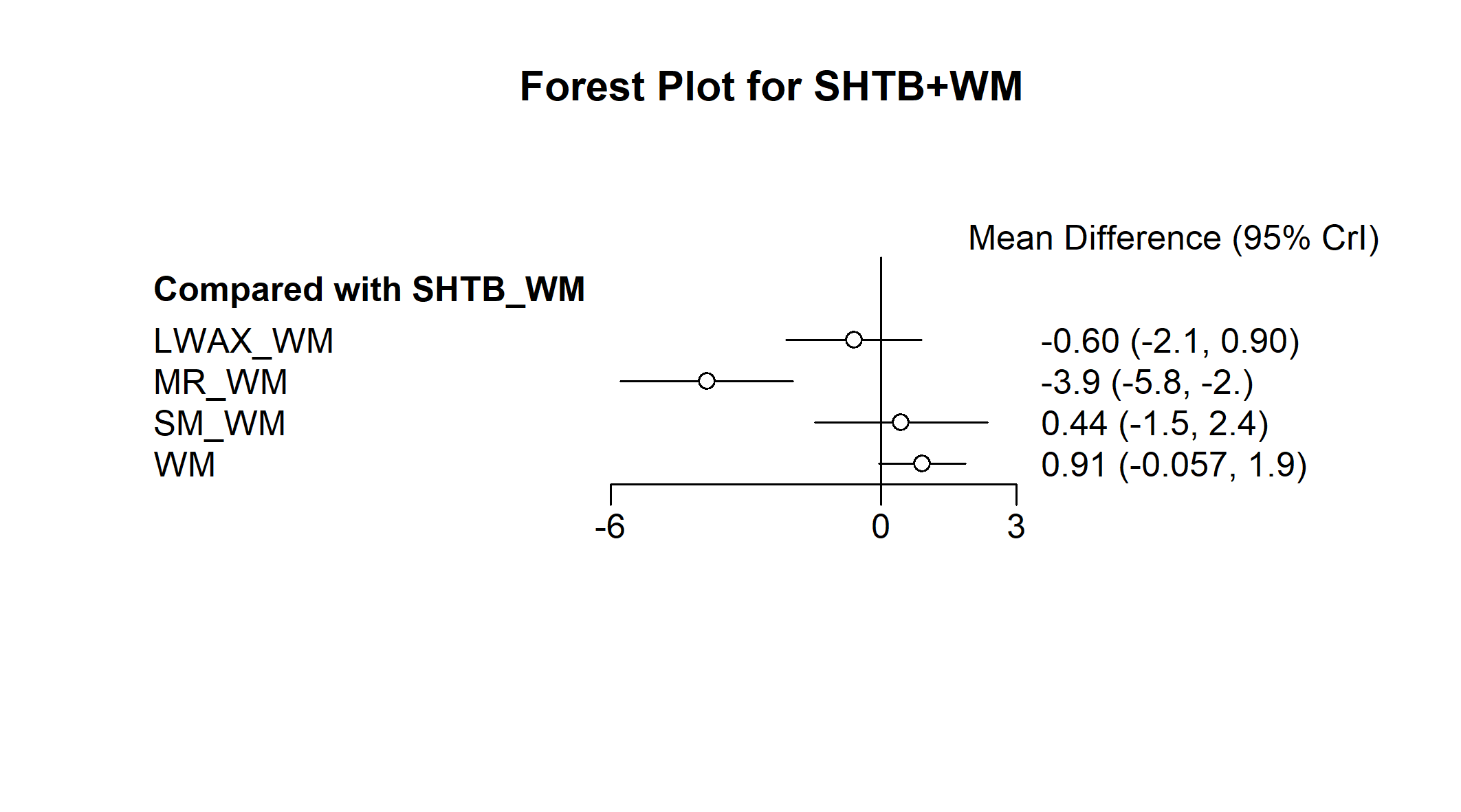

Supplement: Supplementary file 1 [file Data_Sheet_1.zip › Supplementary_Material/Supplementary Figure/abdominal discomfort symptom score/Figure 7.tiff]

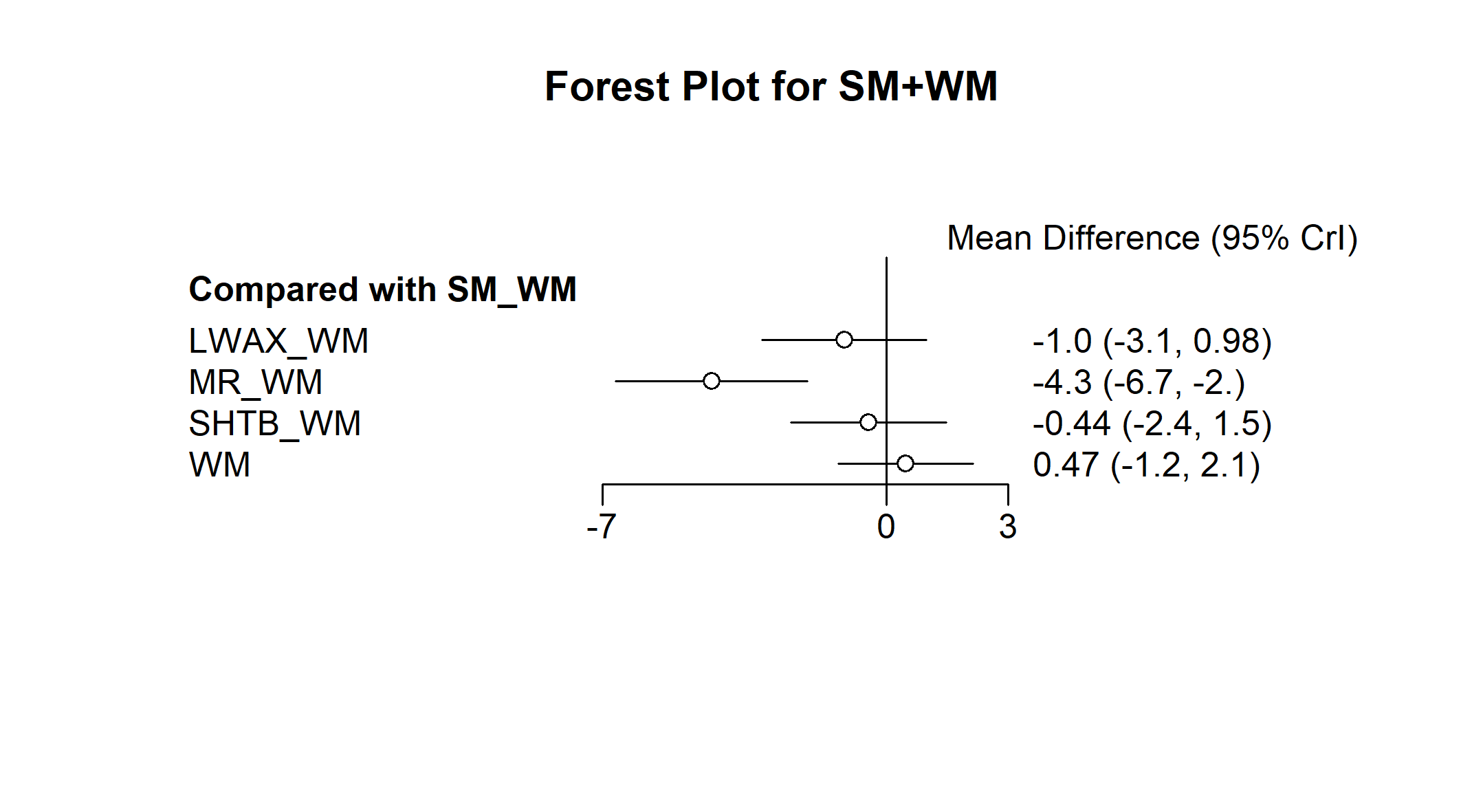

Supplement: Supplementary file 1 [file Data_Sheet_1.zip › Supplementary_Material/Supplementary Figure/abdominal discomfort symptom score/Figure 8.tiff]

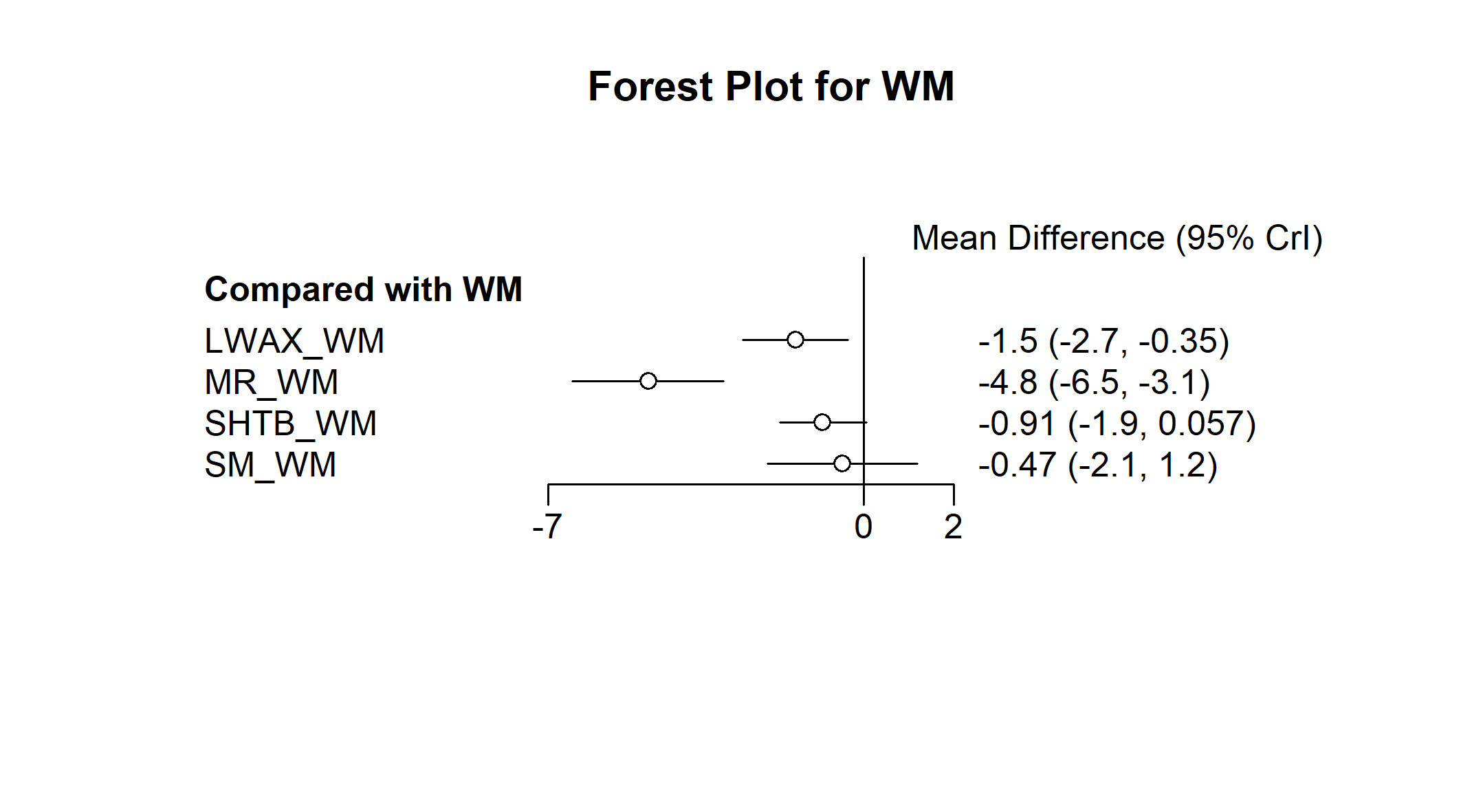

Supplement: Supplementary file 1 [file Data_Sheet_1.zip › Supplementary_Material/Supplementary Figure/abdominal discomfort symptom score/Figure 9.tiff]

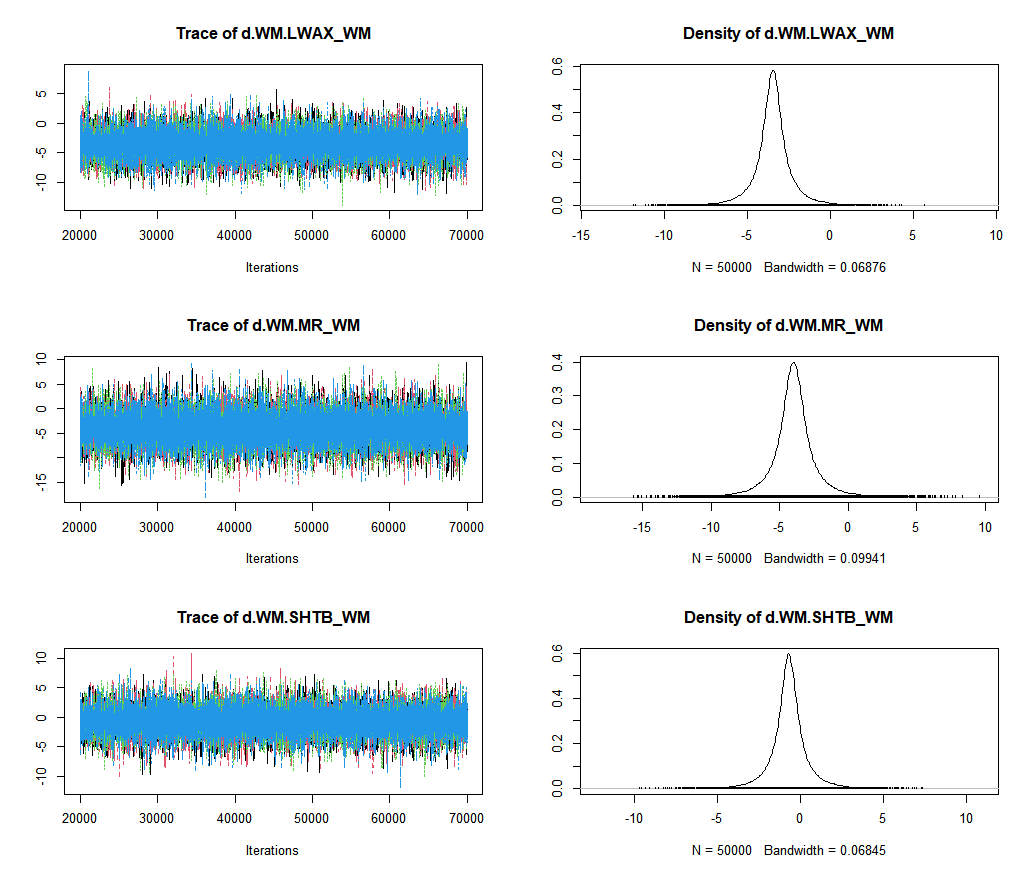

Supplement: Supplementary file 1 [file Data_Sheet_1.zip › Supplementary_Material/Supplementary Figure/defecation frequency score/Figure 1.tiff]

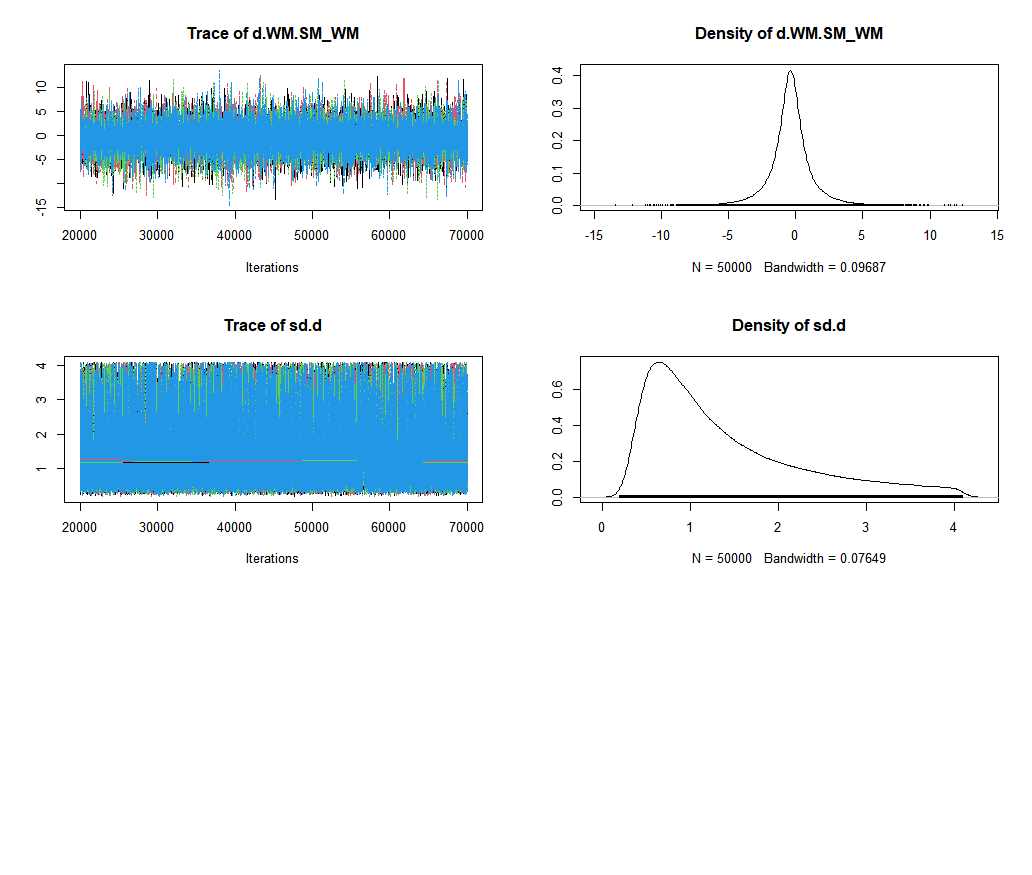

Supplement: Supplementary file 1 [file Data_Sheet_1.zip › Supplementary_Material/Supplementary Figure/defecation frequency score/Figure 2.tiff]

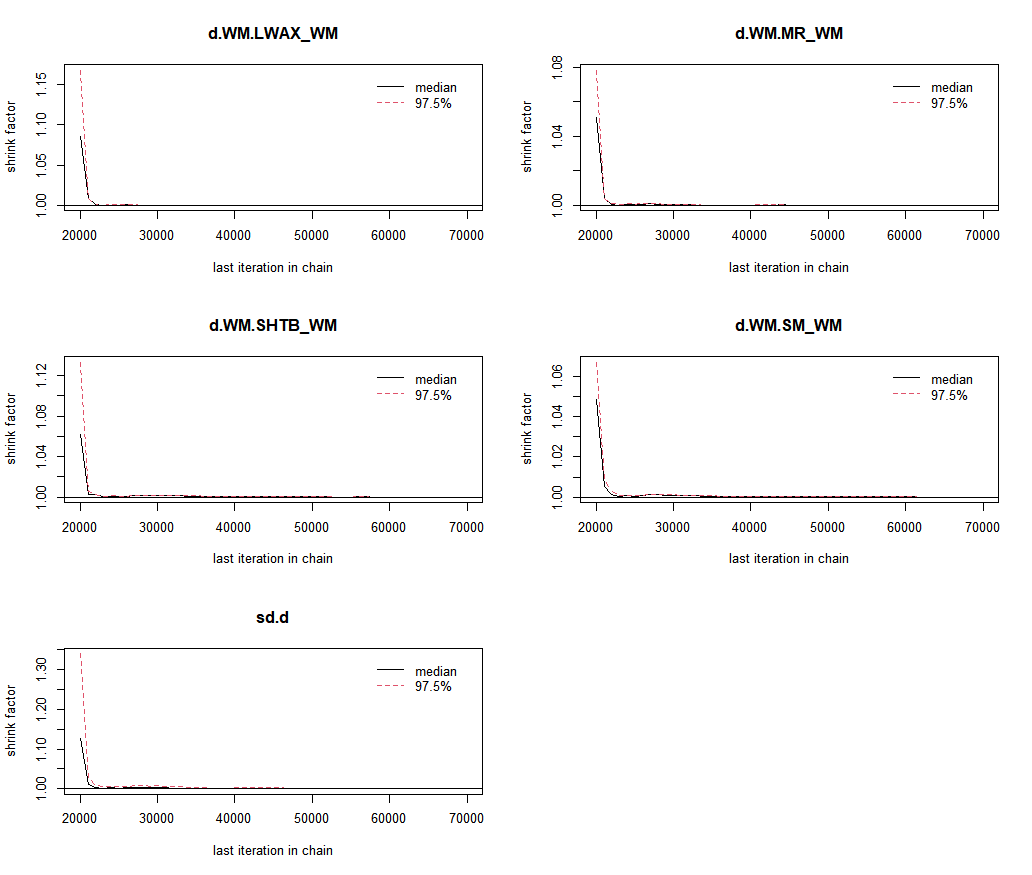

Supplement: Supplementary file 1 [file Data_Sheet_1.zip › Supplementary_Material/Supplementary Figure/defecation frequency score/Figure 3.tiff]

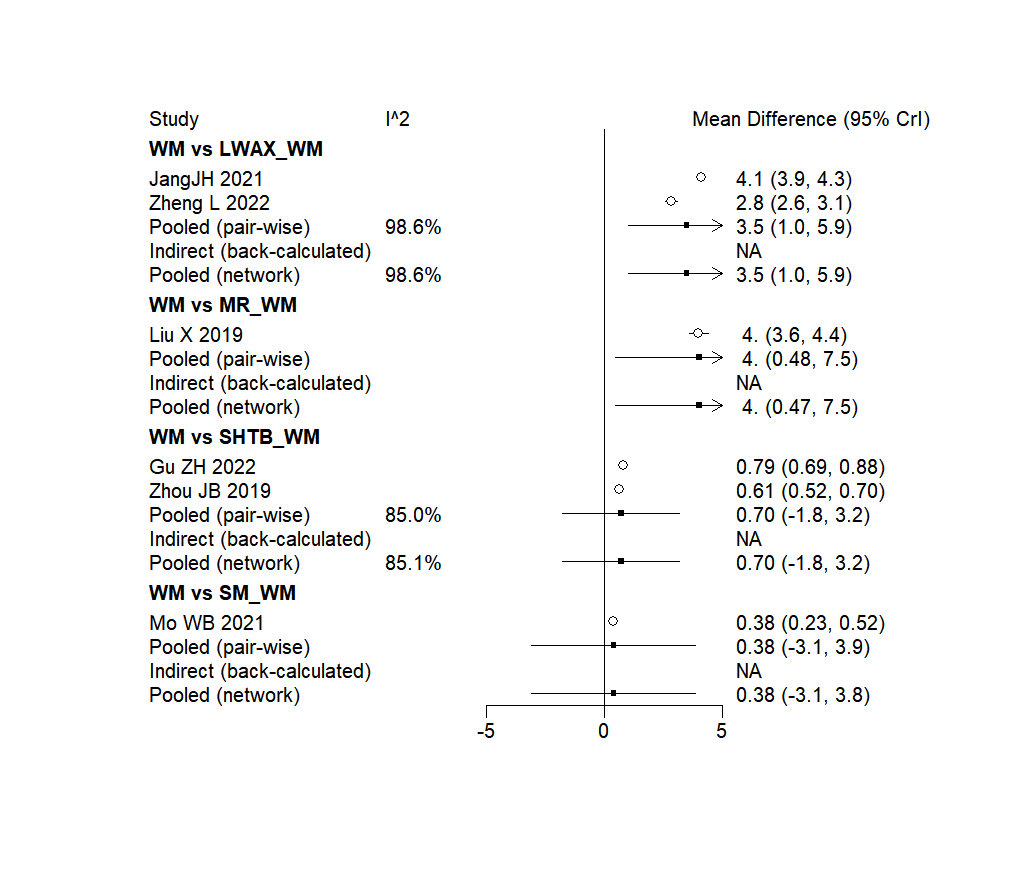

Supplement: Supplementary file 1 [file Data_Sheet_1.zip › Supplementary_Material/Supplementary Figure/defecation frequency score/Figure 4.tiff]

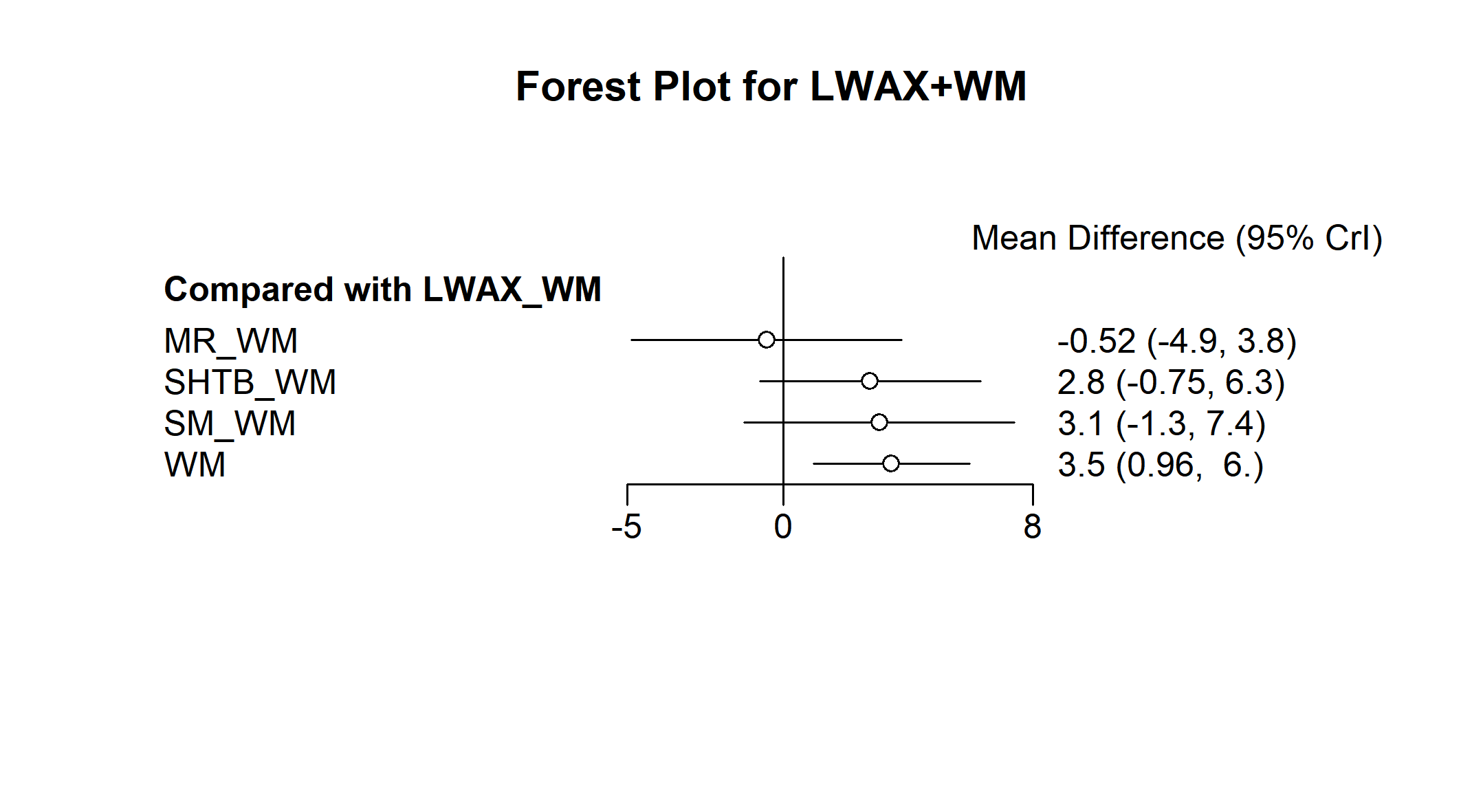

Supplement: Supplementary file 1 [file Data_Sheet_1.zip › Supplementary_Material/Supplementary Figure/defecation frequency score/Figure 5.tiff]

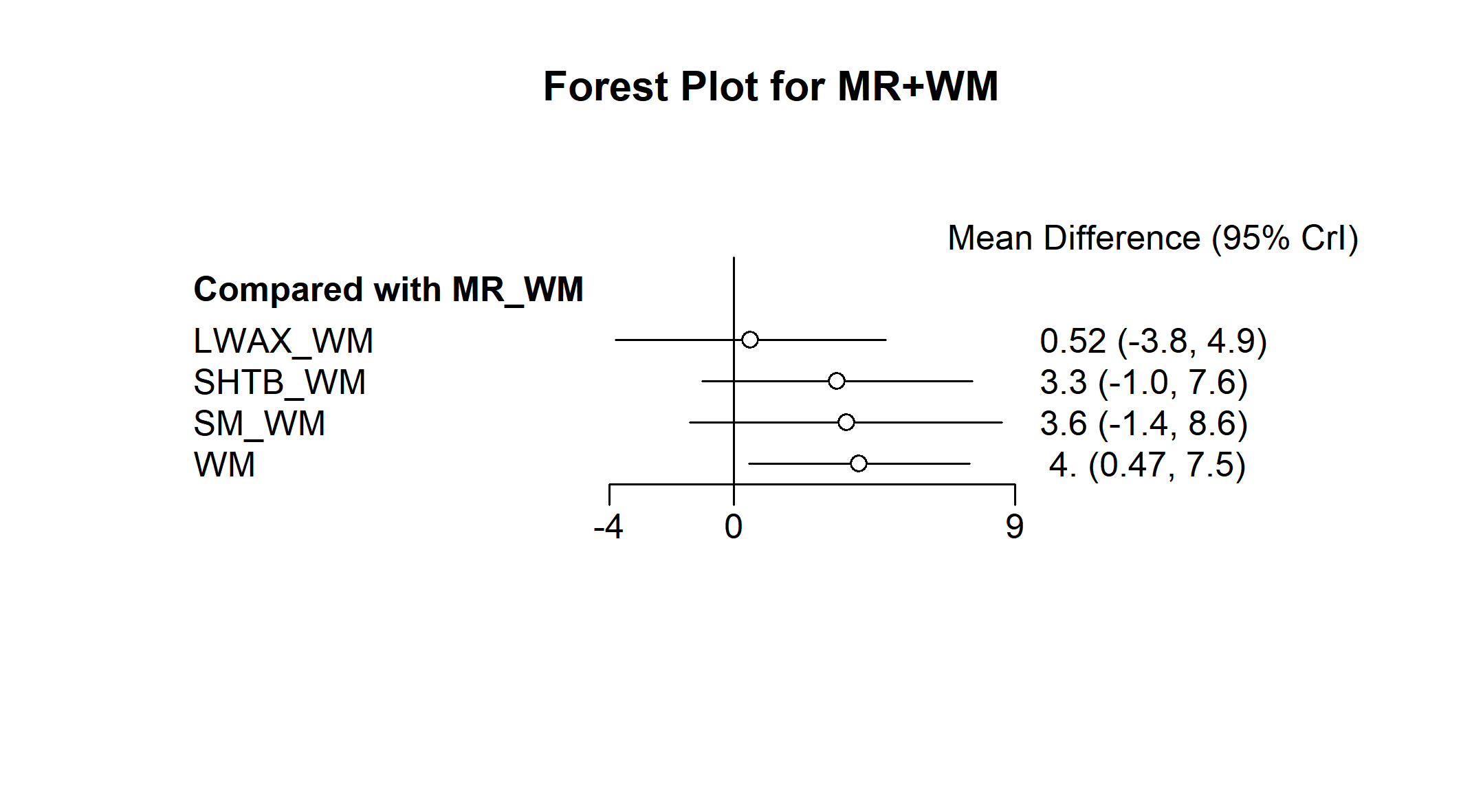

Supplement: Supplementary file 1 [file Data_Sheet_1.zip › Supplementary_Material/Supplementary Figure/defecation frequency score/Figure 6.tiff]

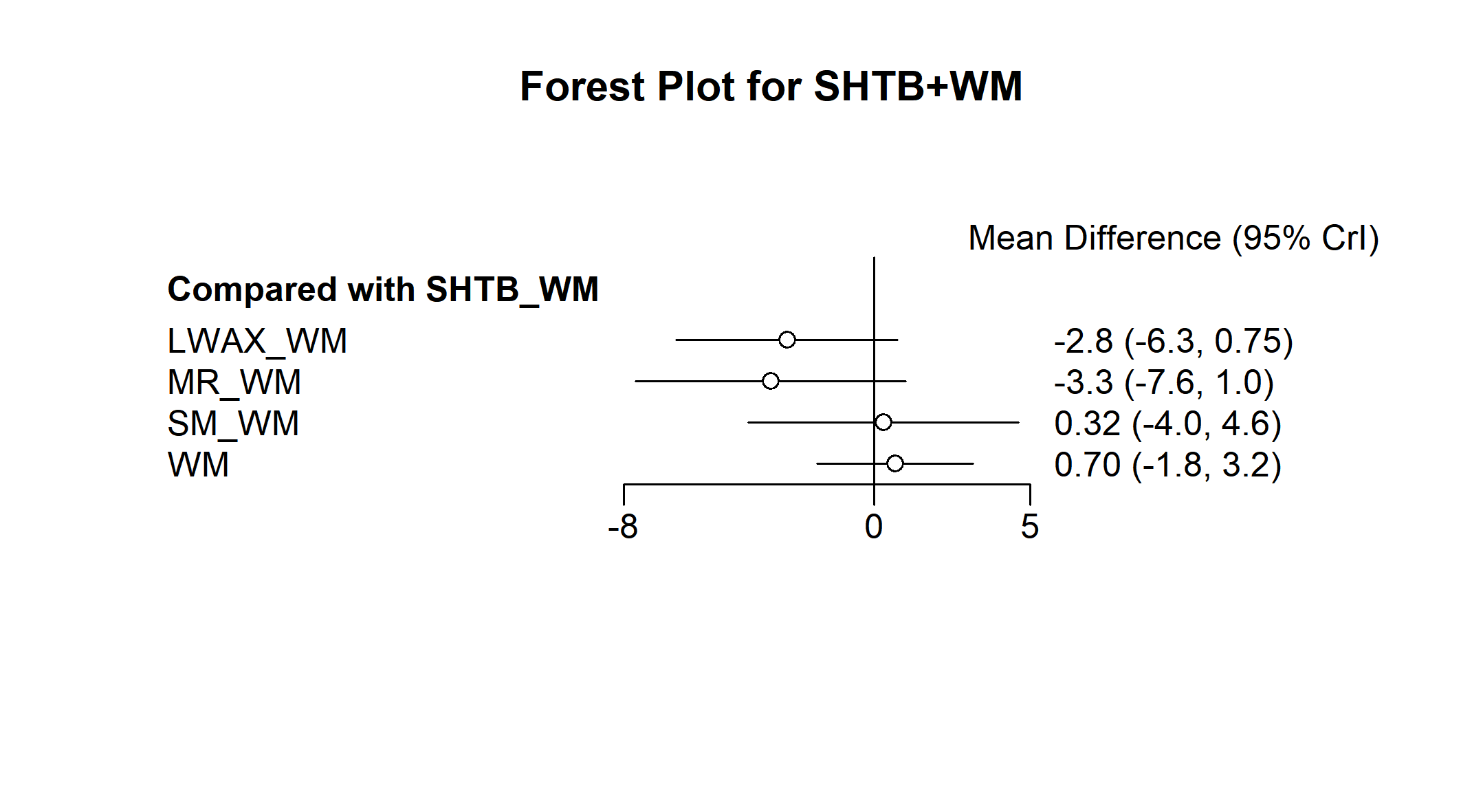

Supplement: Supplementary file 1 [file Data_Sheet_1.zip › Supplementary_Material/Supplementary Figure/defecation frequency score/Figure 7.tiff]

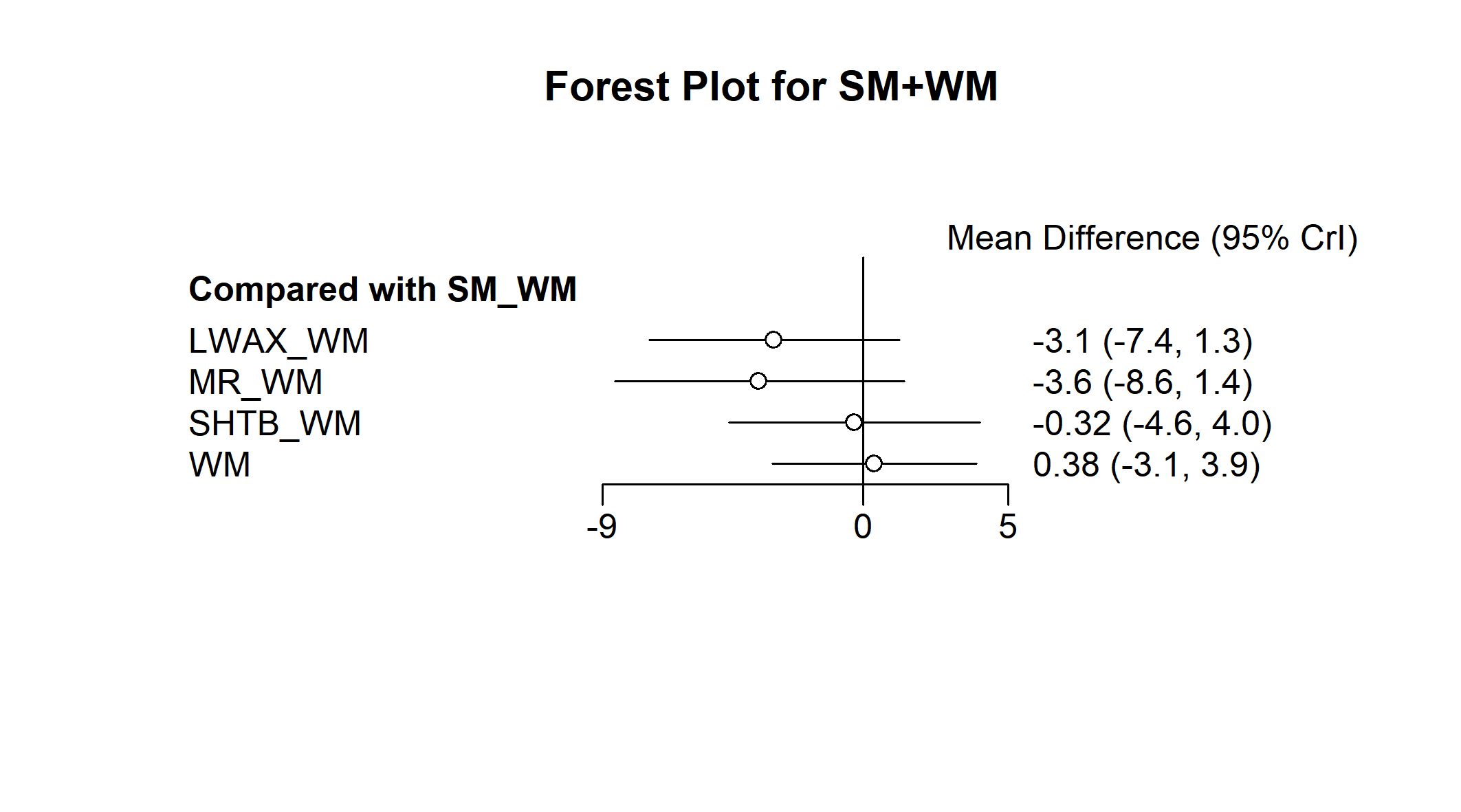

Supplement: Supplementary file 1 [file Data_Sheet_1.zip › Supplementary_Material/Supplementary Figure/defecation frequency score/Figure 8.tiff]

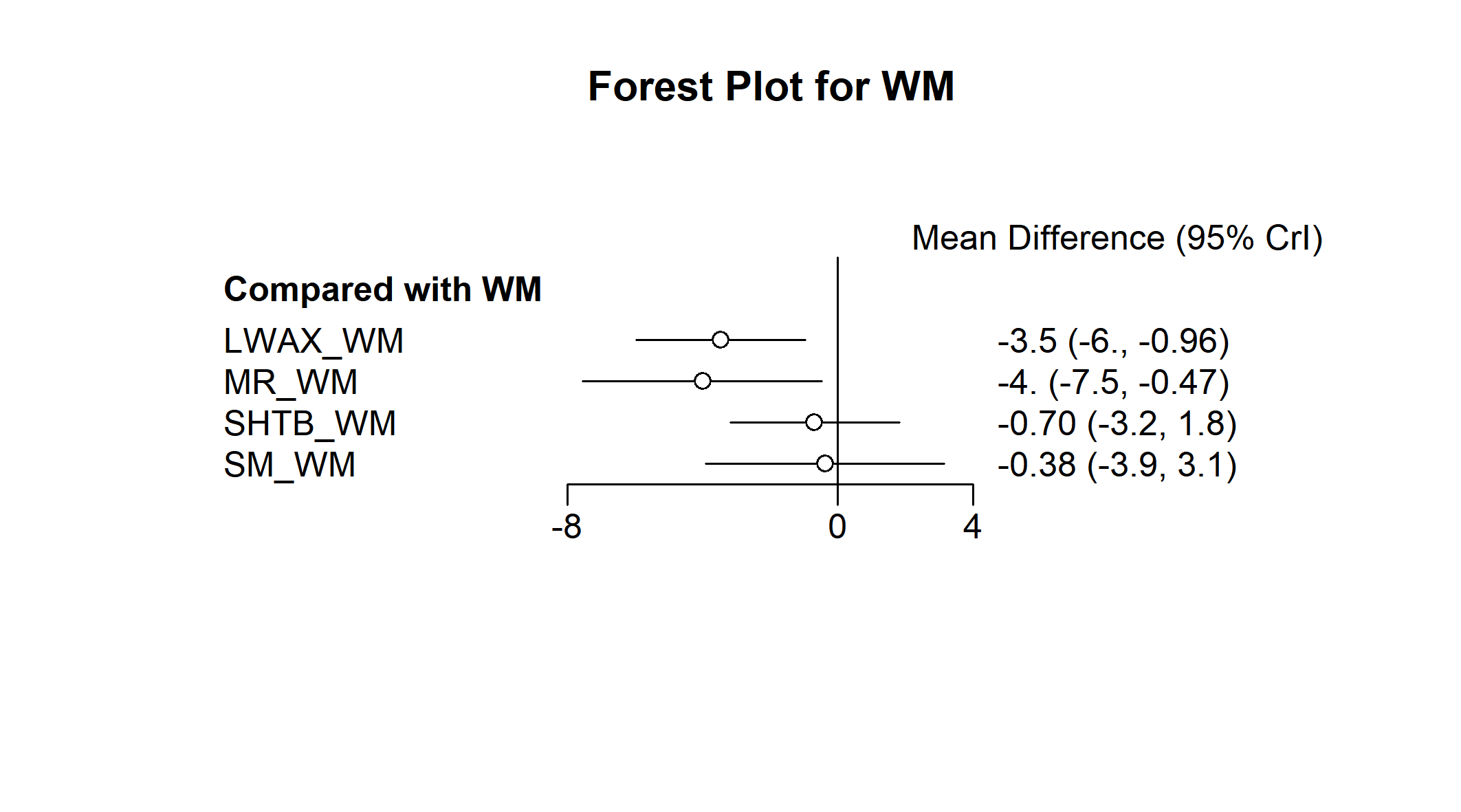

Supplement: Supplementary file 1 [file Data_Sheet_1.zip › Supplementary_Material/Supplementary Figure/defecation frequency score/Figure 9.tiff]

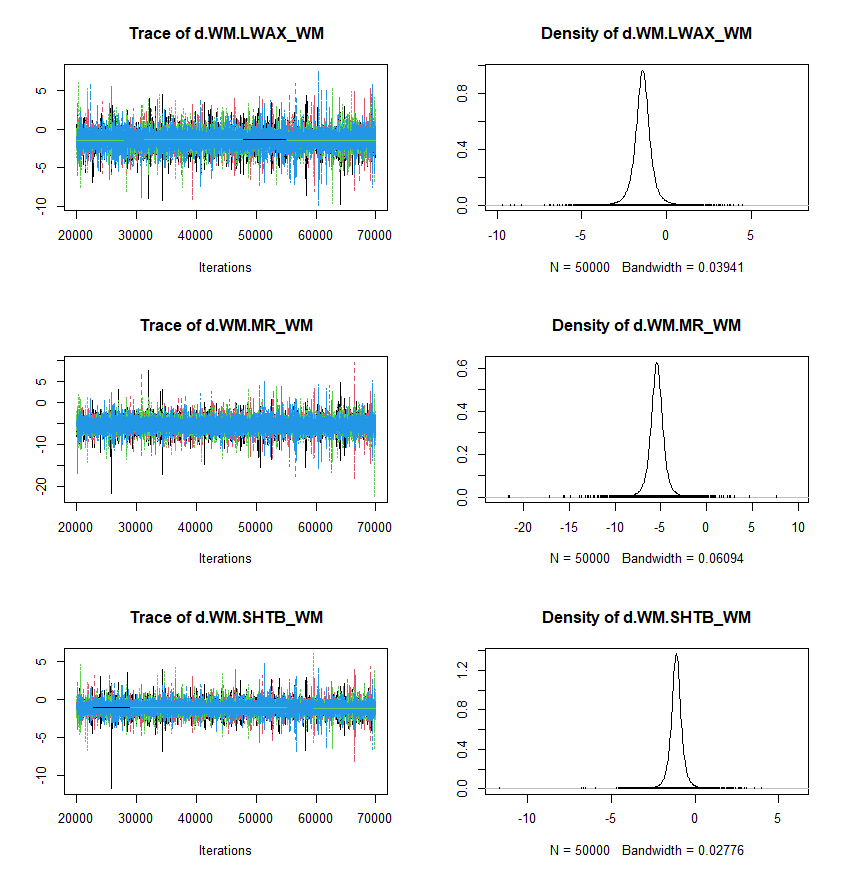

Supplement: Supplementary file 1 [file Data_Sheet_1.zip › Supplementary_Material/Supplementary Figure/difficulty in defecation score/Figure 1.tiff]

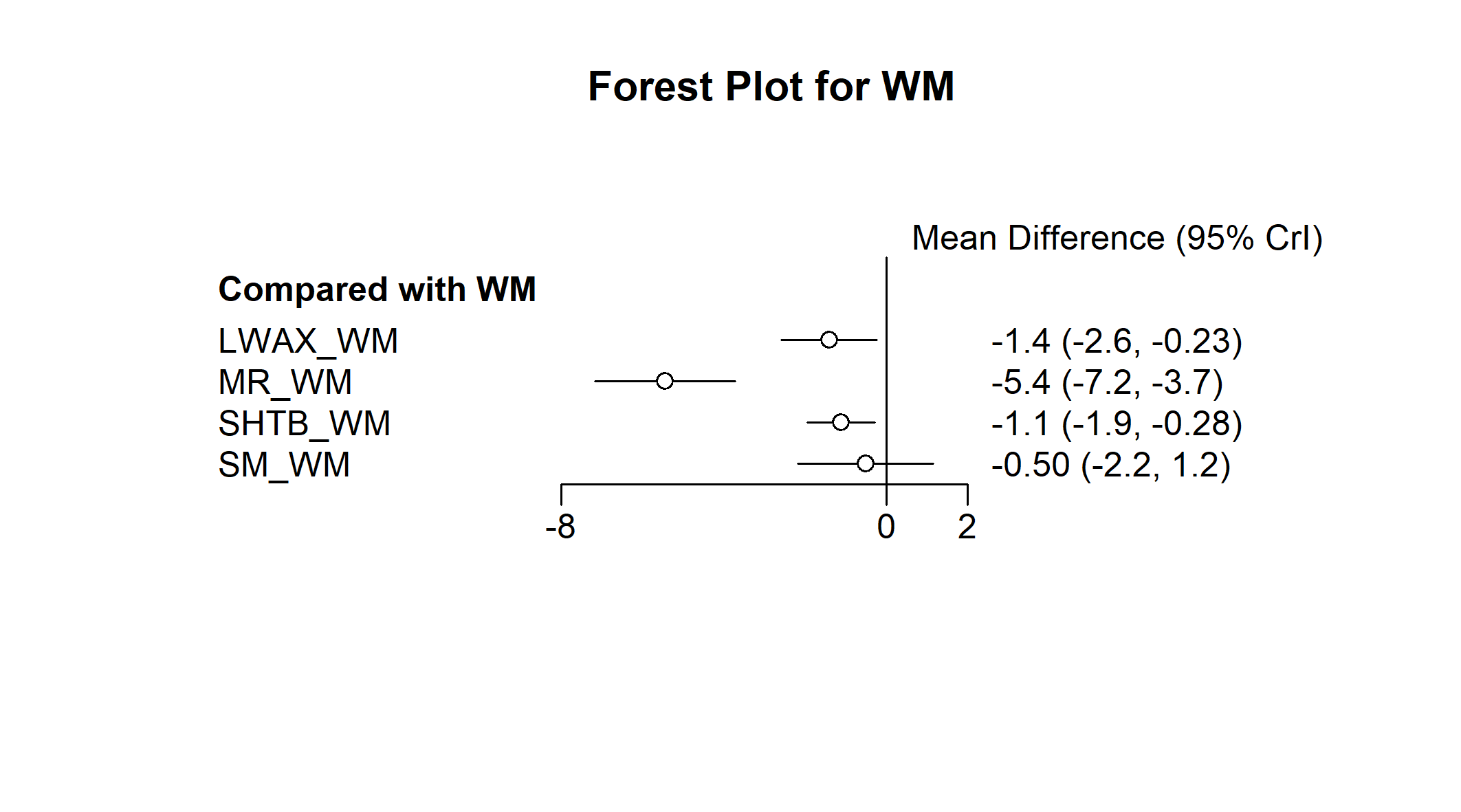

Supplement: Supplementary file 1 [file Data_Sheet_1.zip › Supplementary_Material/Supplementary Figure/difficulty in defecation score/Figure 10.tiff]

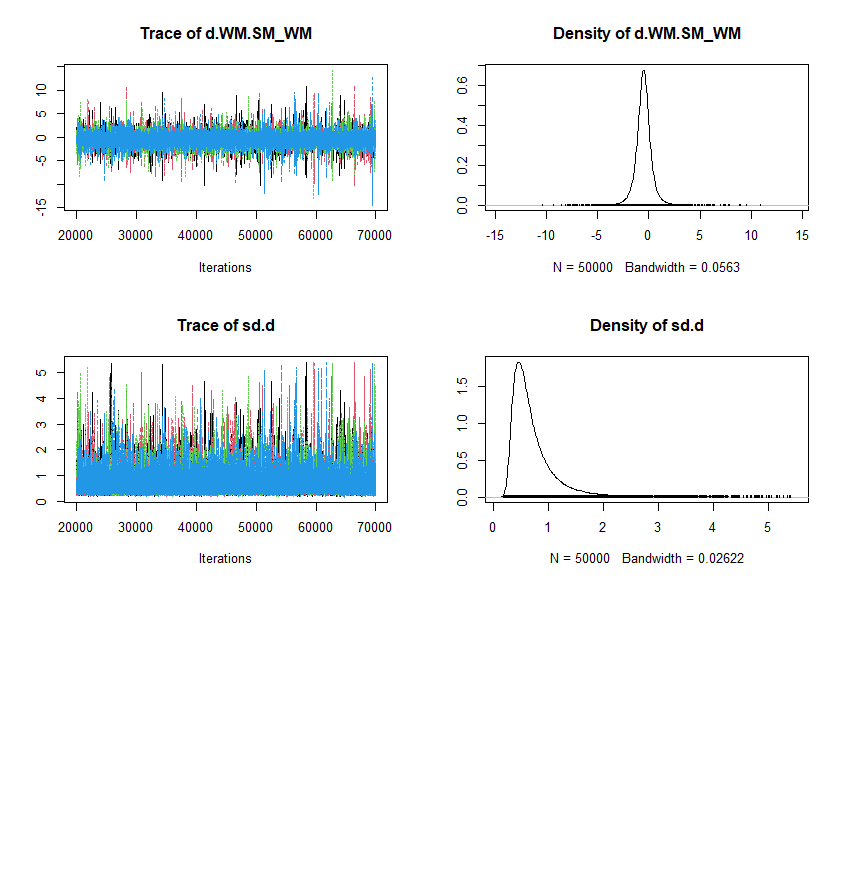

Supplement: Supplementary file 1 [file Data_Sheet_1.zip › Supplementary_Material/Supplementary Figure/difficulty in defecation score/Figure 2.tiff]

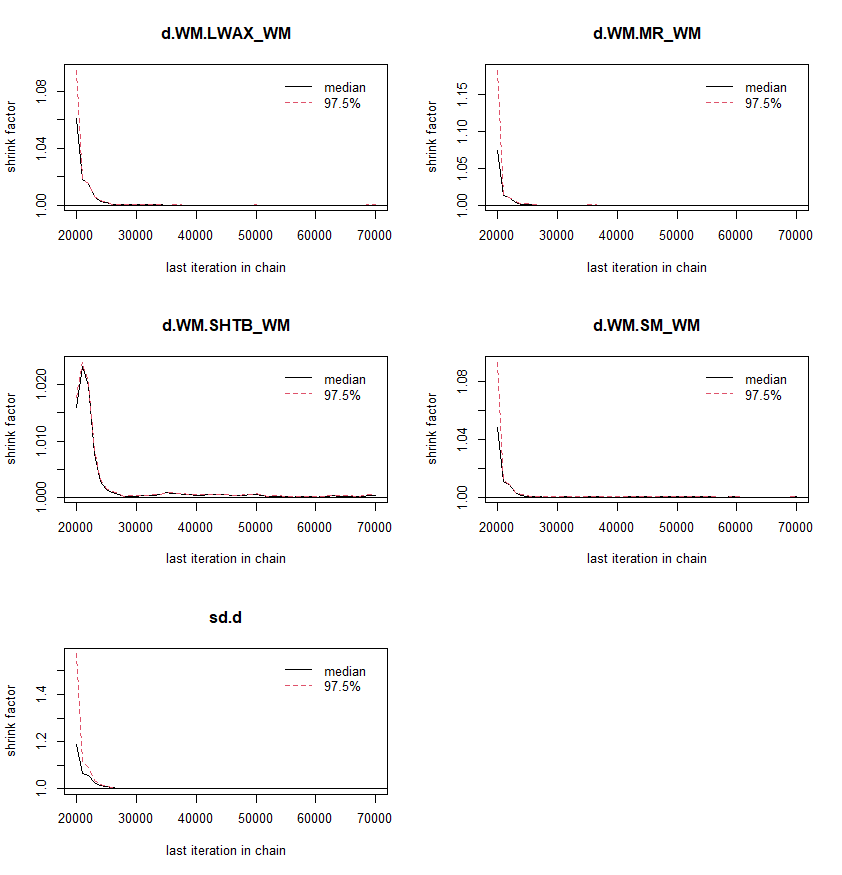

Supplement: Supplementary file 1 [file Data_Sheet_1.zip › Supplementary_Material/Supplementary Figure/difficulty in defecation score/Figure 3.tiff]

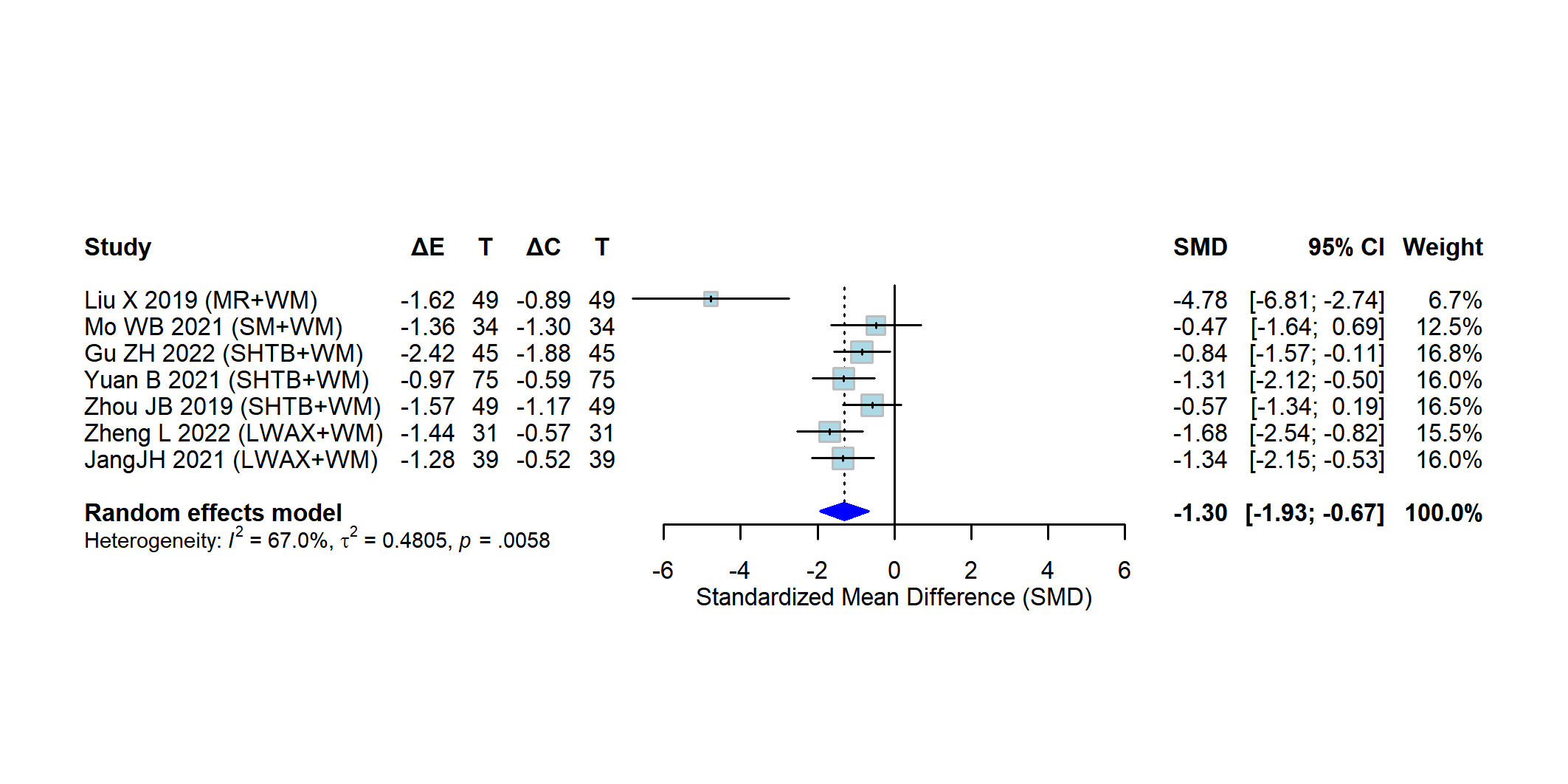

Supplement: Supplementary file 1 [file Data_Sheet_1.zip › Supplementary_Material/Supplementary Figure/difficulty in defecation score/Figure 4.tiff]

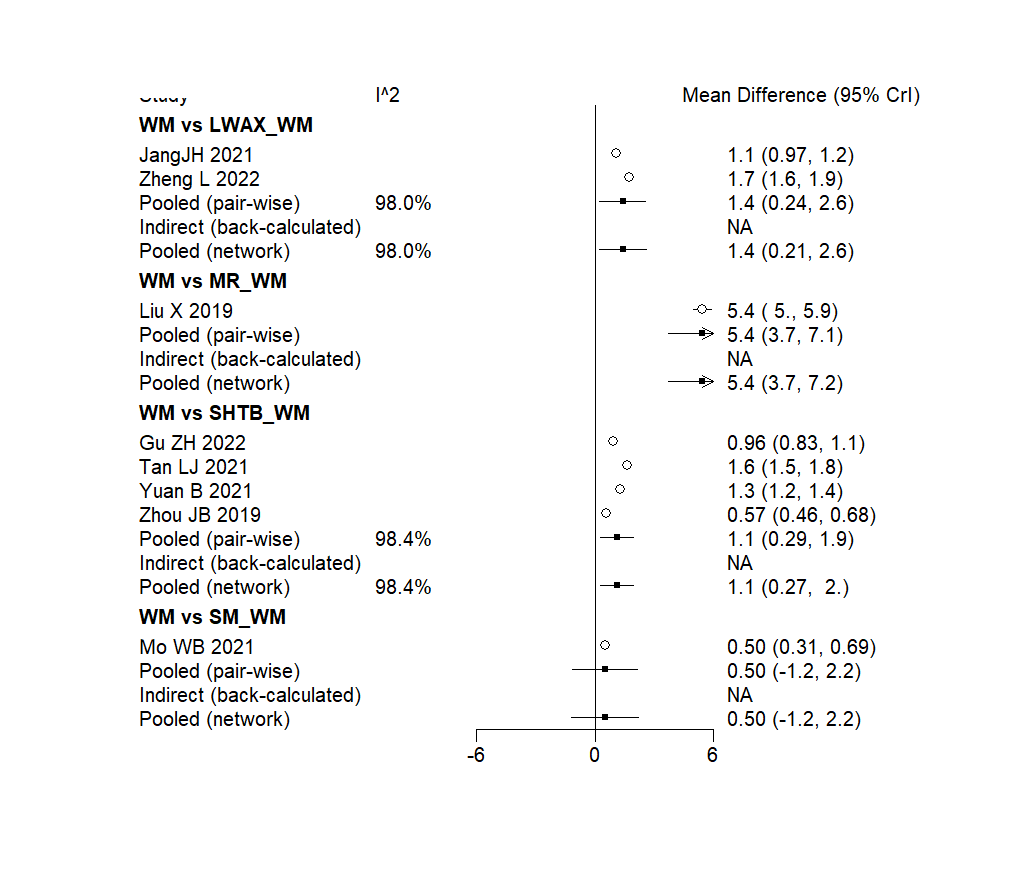

Supplement: Supplementary file 1 [file Data_Sheet_1.zip › Supplementary_Material/Supplementary Figure/difficulty in defecation score/Figure 5.tiff]

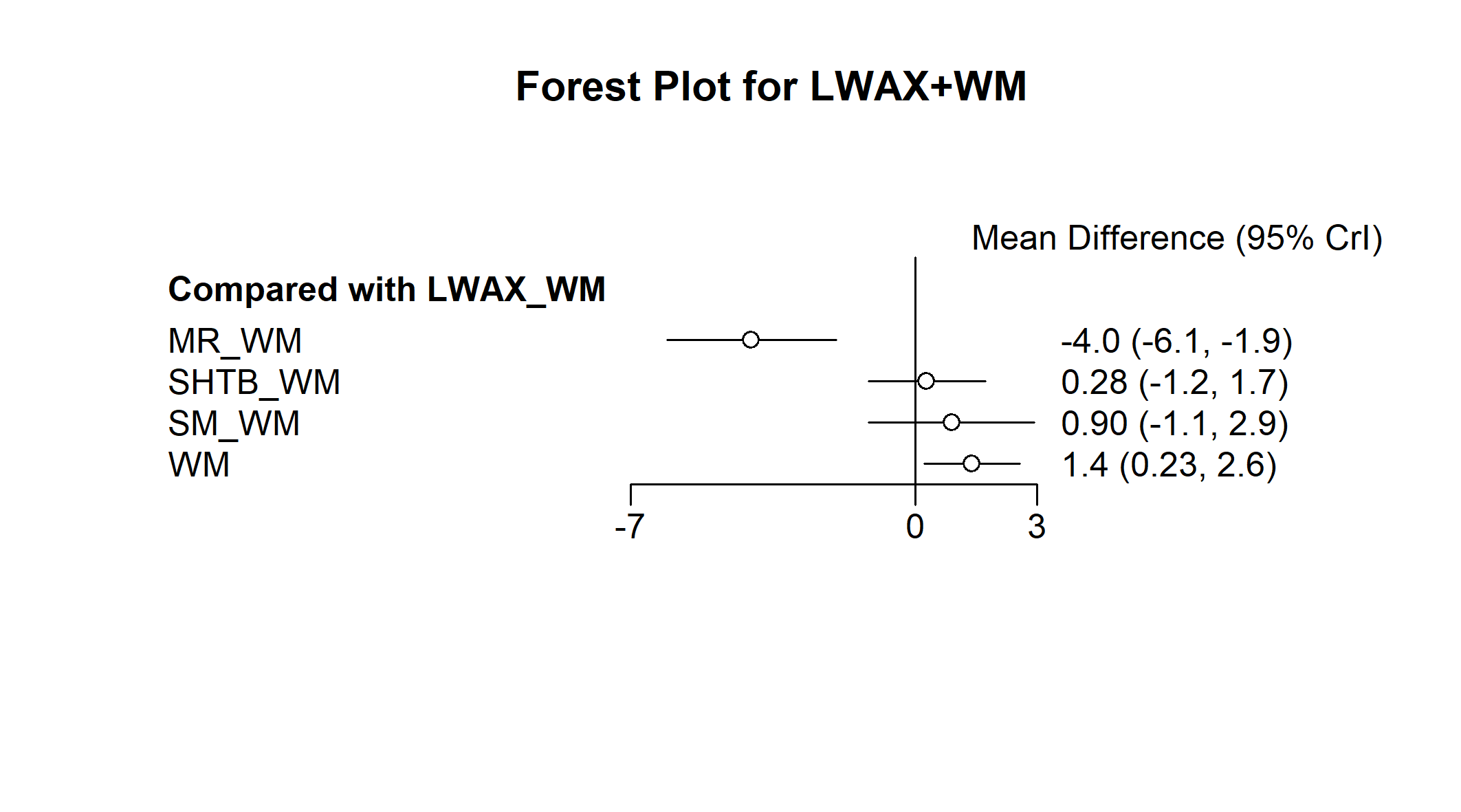

Supplement: Supplementary file 1 [file Data_Sheet_1.zip › Supplementary_Material/Supplementary Figure/difficulty in defecation score/Figure 6.tiff]

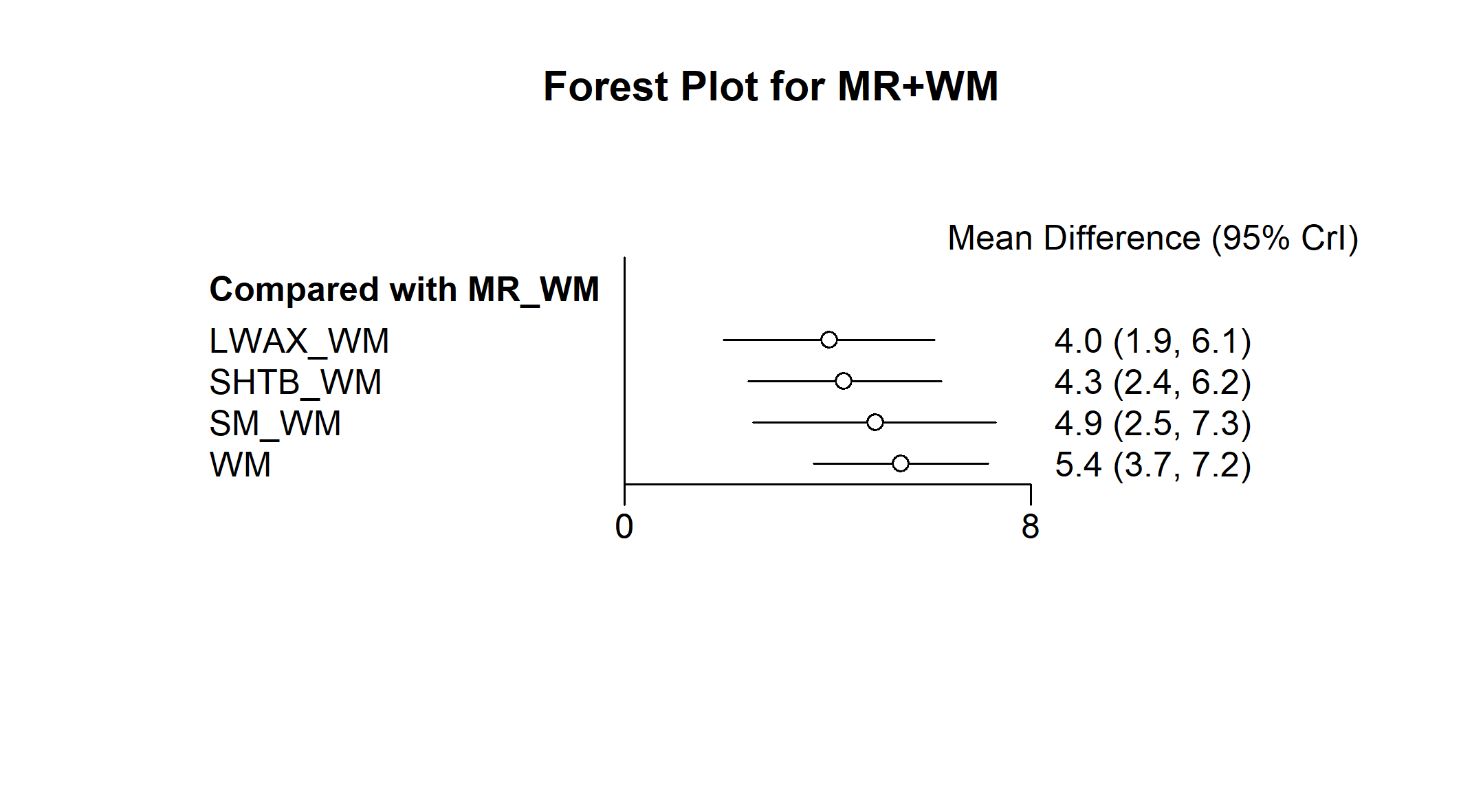

Supplement: Supplementary file 1 [file Data_Sheet_1.zip › Supplementary_Material/Supplementary Figure/difficulty in defecation score/Figure 7.tiff]

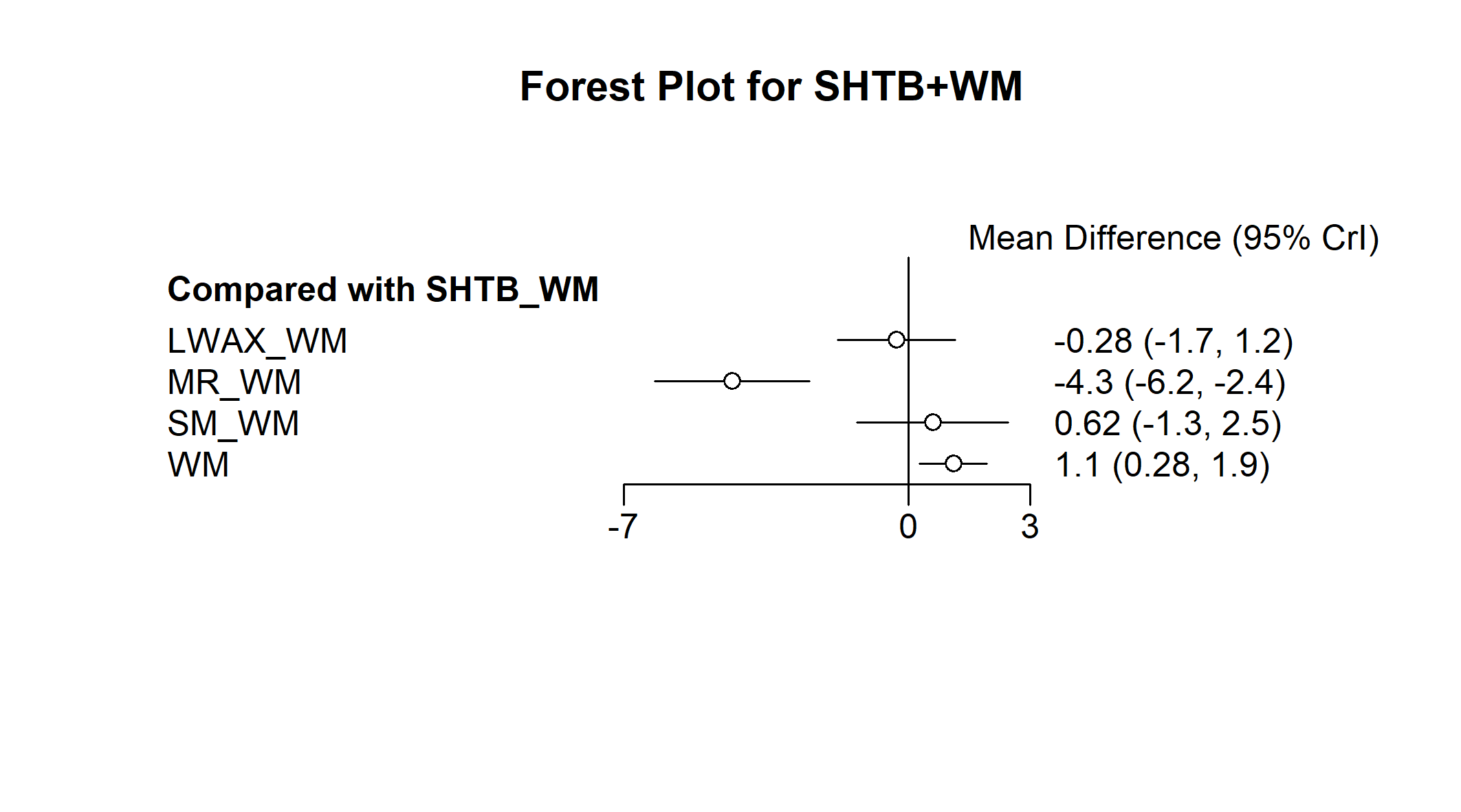

Supplement: Supplementary file 1 [file Data_Sheet_1.zip › Supplementary_Material/Supplementary Figure/difficulty in defecation score/Figure 8.tiff]

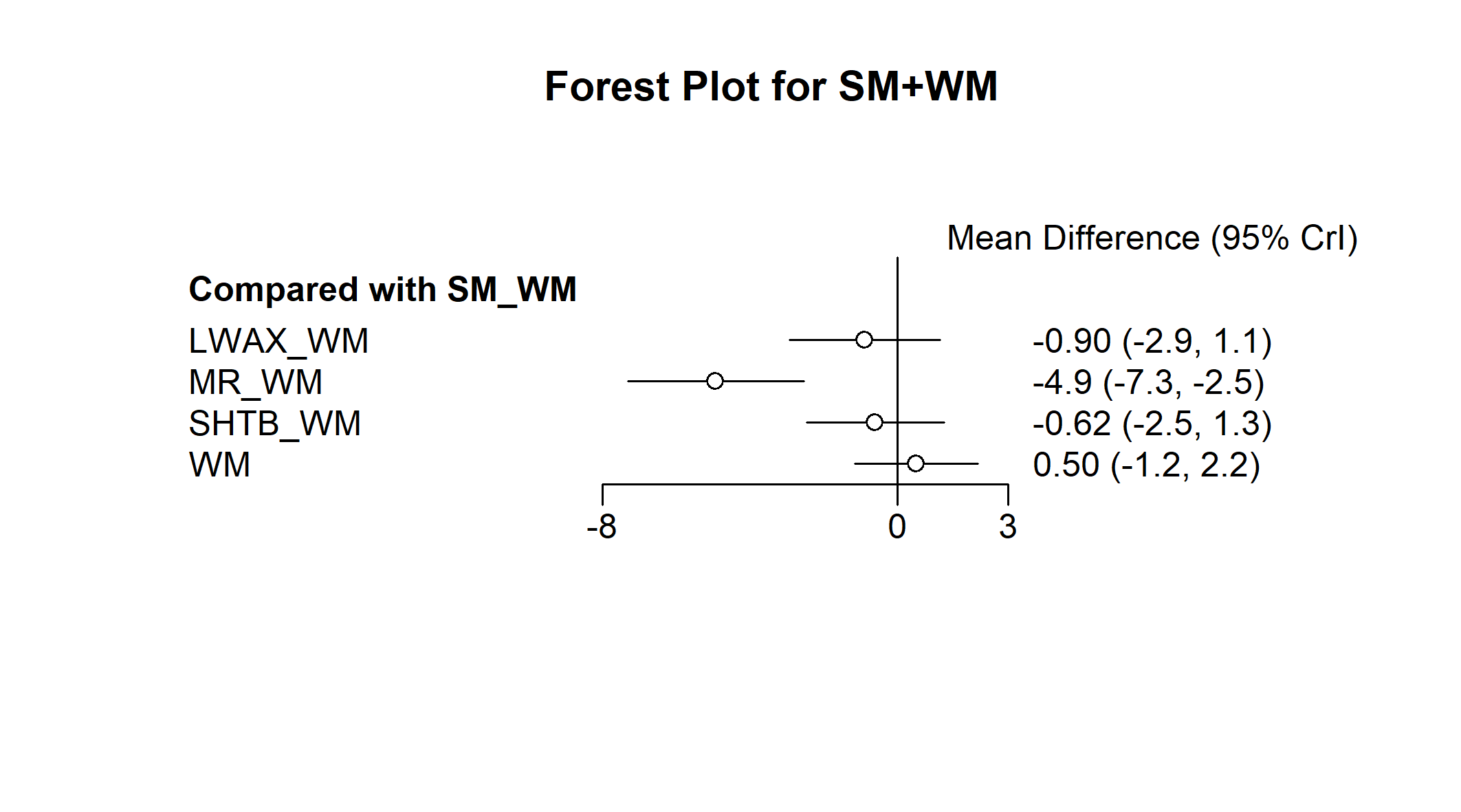

Supplement: Supplementary file 1 [file Data_Sheet_1.zip › Supplementary_Material/Supplementary Figure/difficulty in defecation score/Figure 9.tiff]

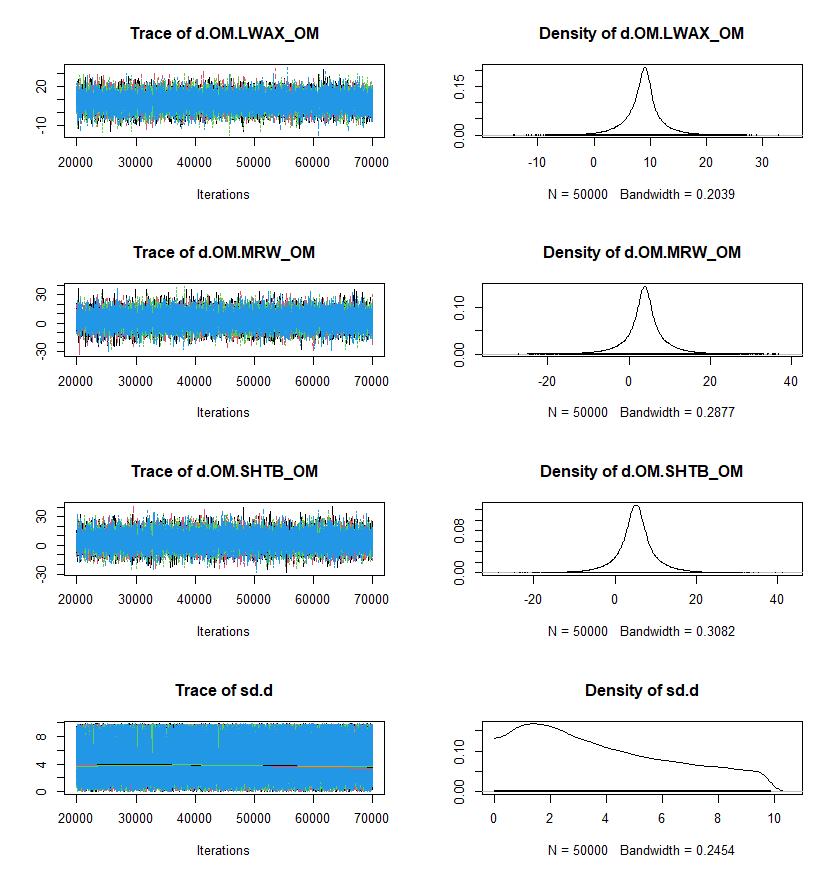

Supplement: Supplementary file 1 [file Data_Sheet_1.zip › Supplementary_Material/Supplementary Figure/GAS/Figure 1.tiff]

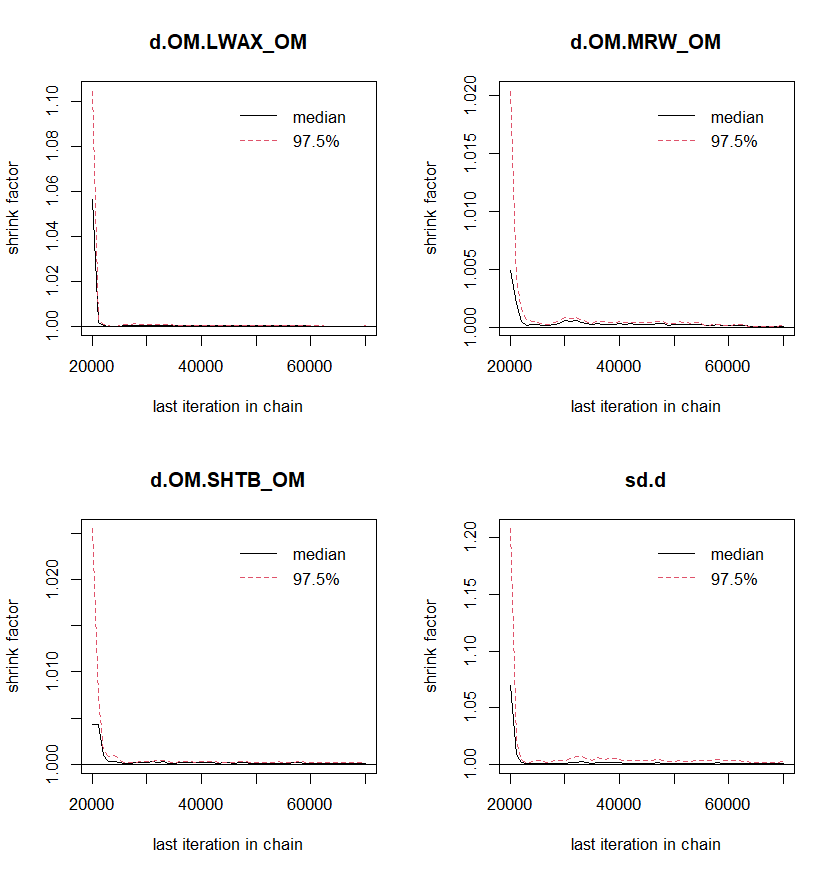

Supplement: Supplementary file 1 [file Data_Sheet_1.zip › Supplementary_Material/Supplementary Figure/GAS/Figure 2.tiff]

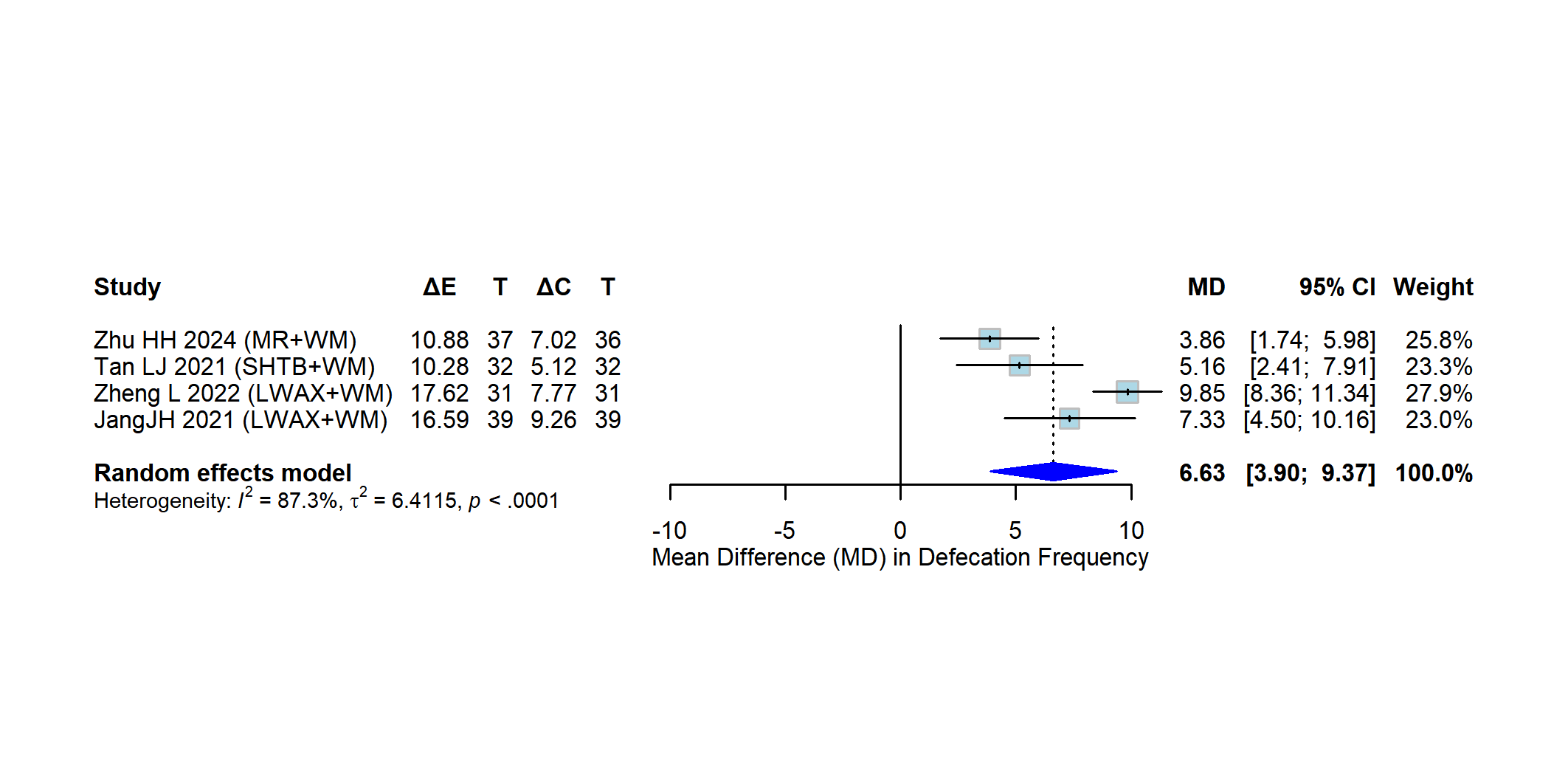

Supplement: Supplementary file 1 [file Data_Sheet_1.zip › Supplementary_Material/Supplementary Figure/GAS/forest_plot_MD.tiff]

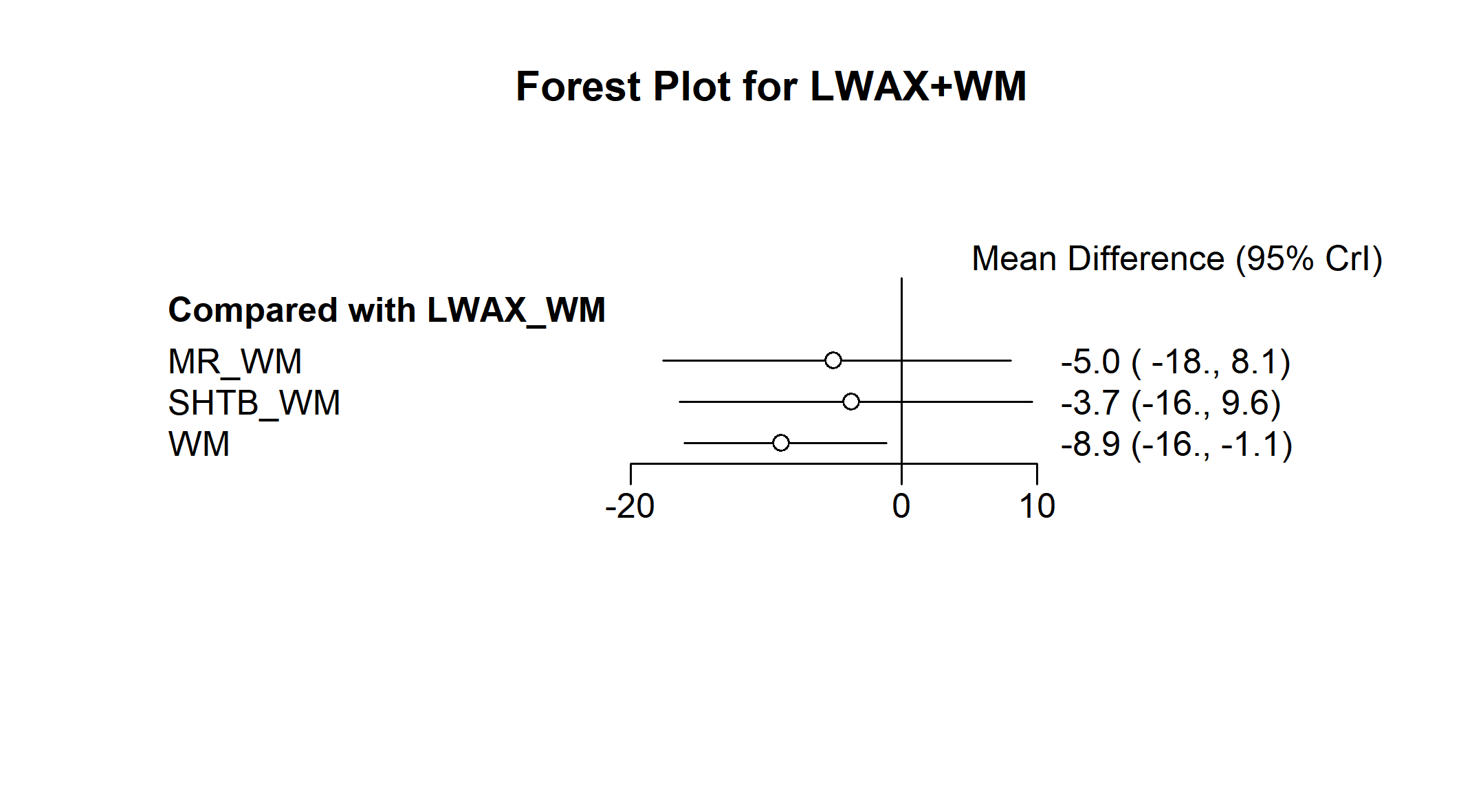

Supplement: Supplementary file 1 [file Data_Sheet_1.zip › Supplementary_Material/Supplementary Figure/GAS/GASforest_LWAX+WM.tiff]

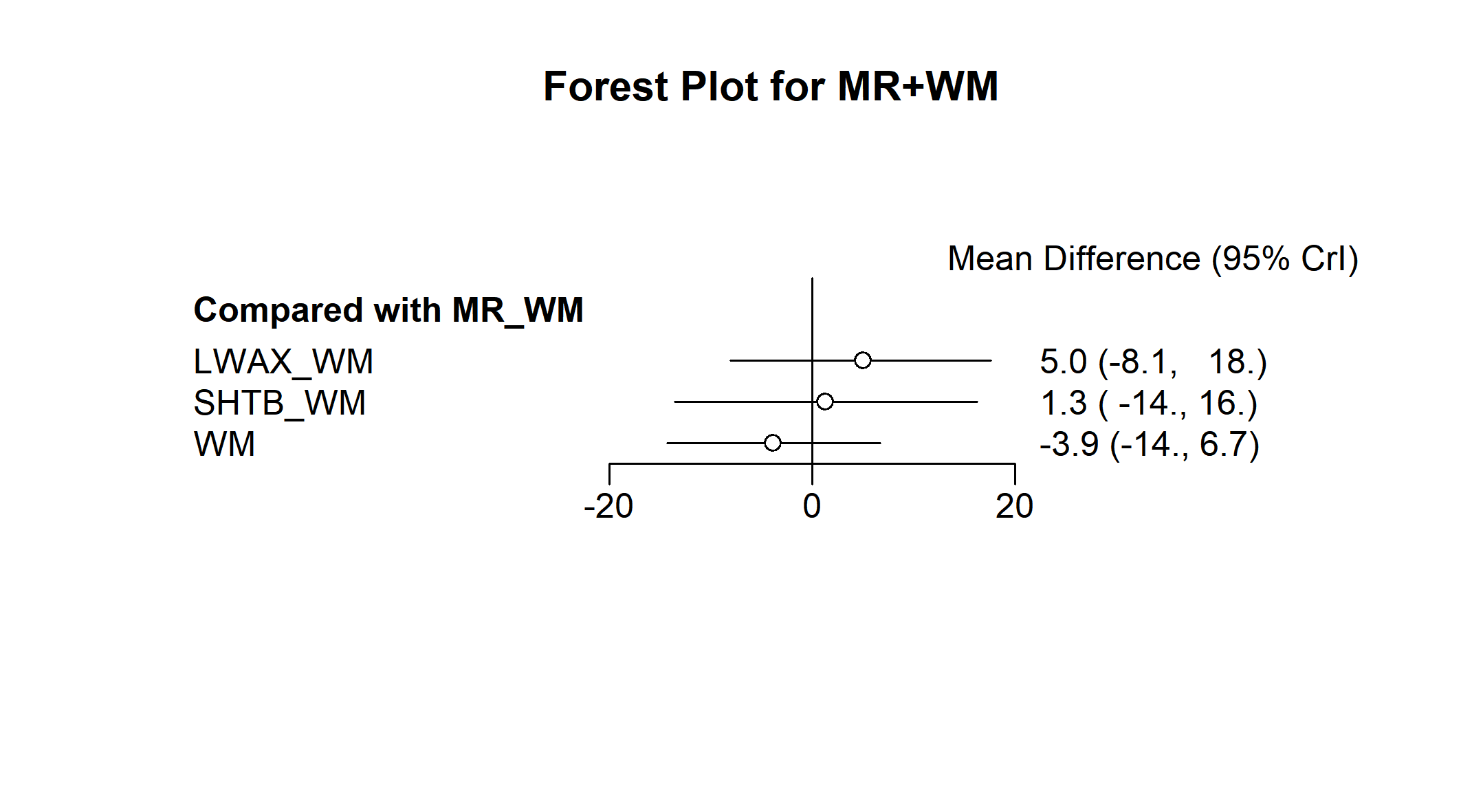

Supplement: Supplementary file 1 [file Data_Sheet_1.zip › Supplementary_Material/Supplementary Figure/GAS/GASforest_MR+WM.tiff]

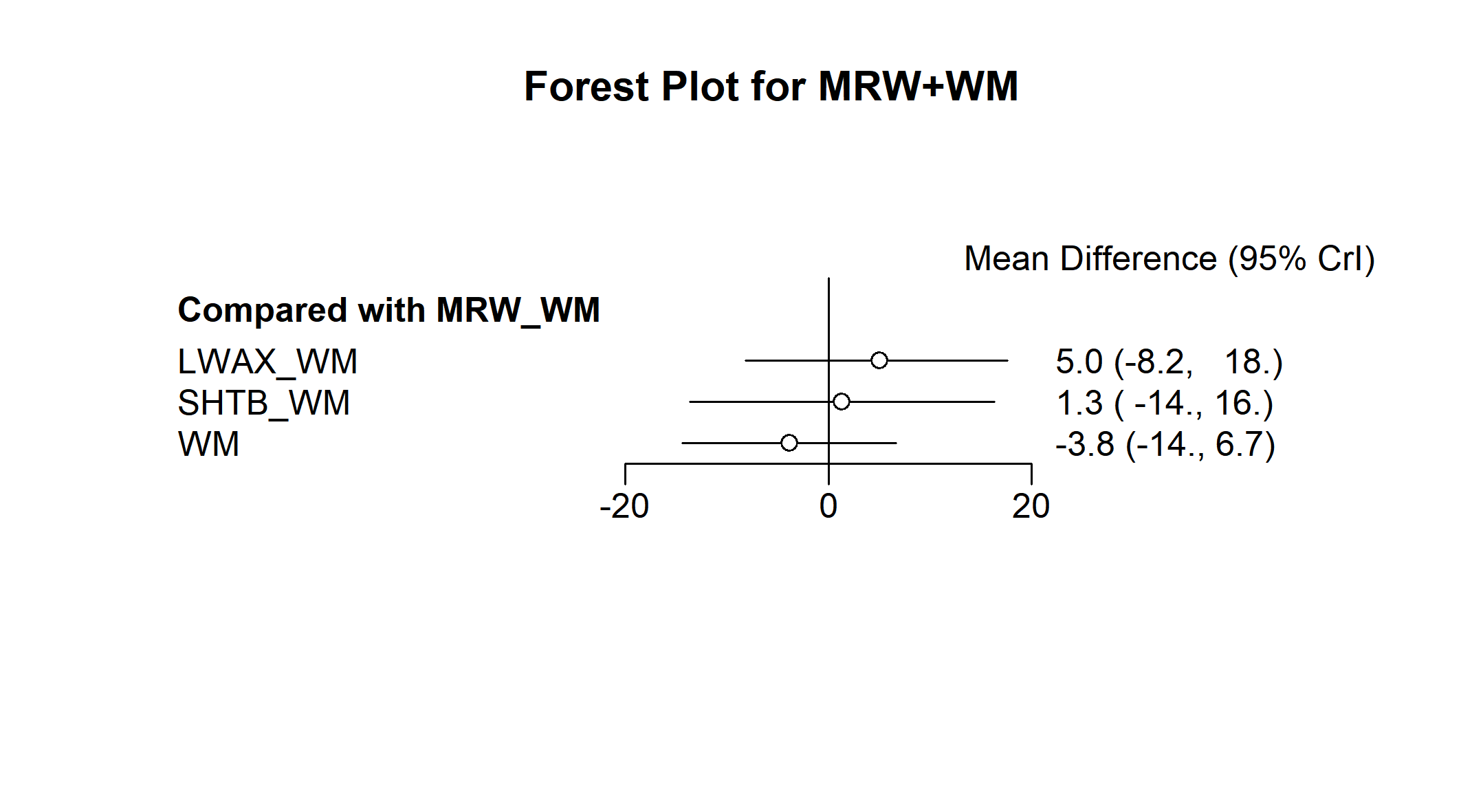

Supplement: Supplementary file 1 [file Data_Sheet_1.zip › Supplementary_Material/Supplementary Figure/GAS/GASforest_MRW+WM.tiff]

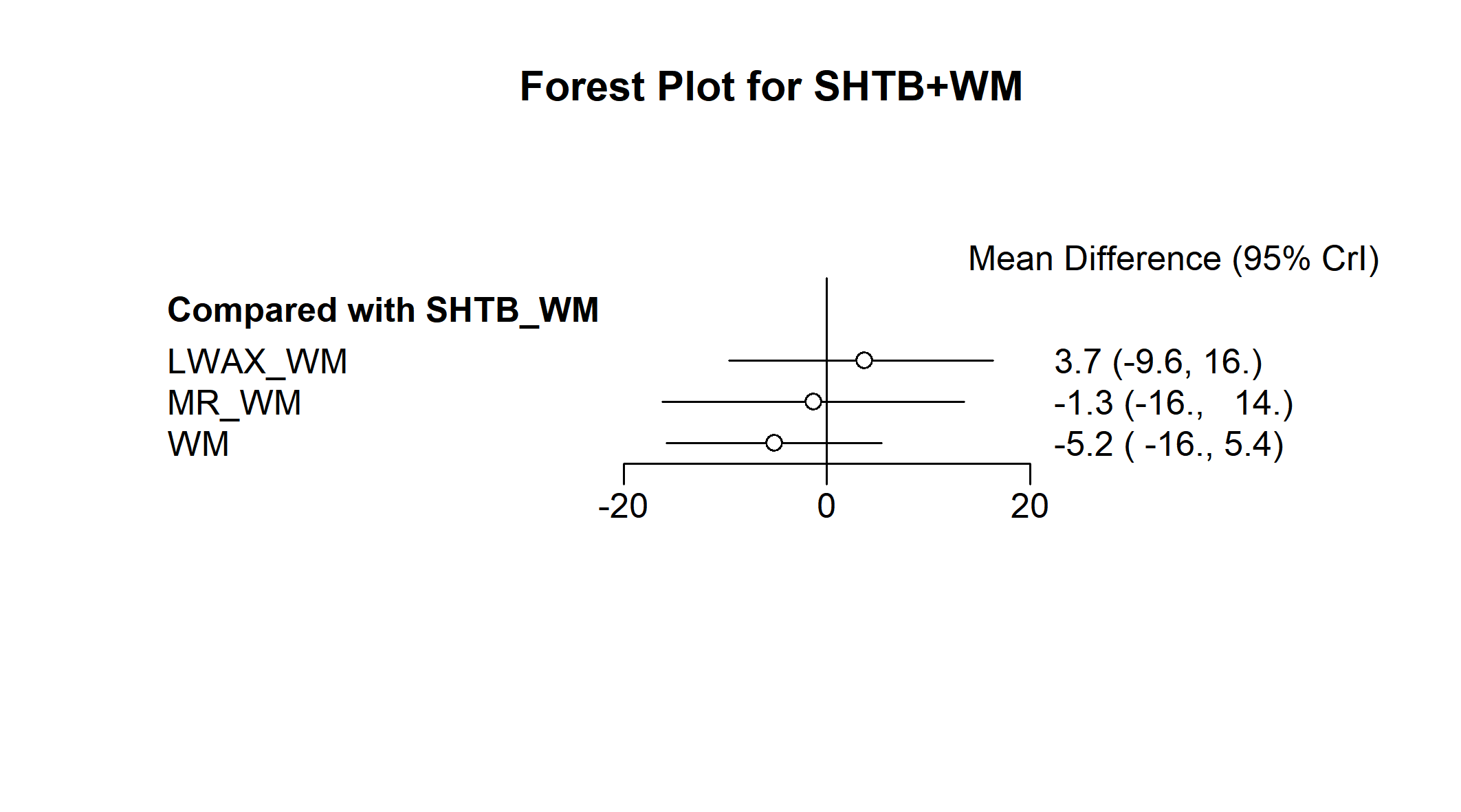

Supplement: Supplementary file 1 [file Data_Sheet_1.zip › Supplementary_Material/Supplementary Figure/GAS/GASforest_SHTB+WM.tiff]

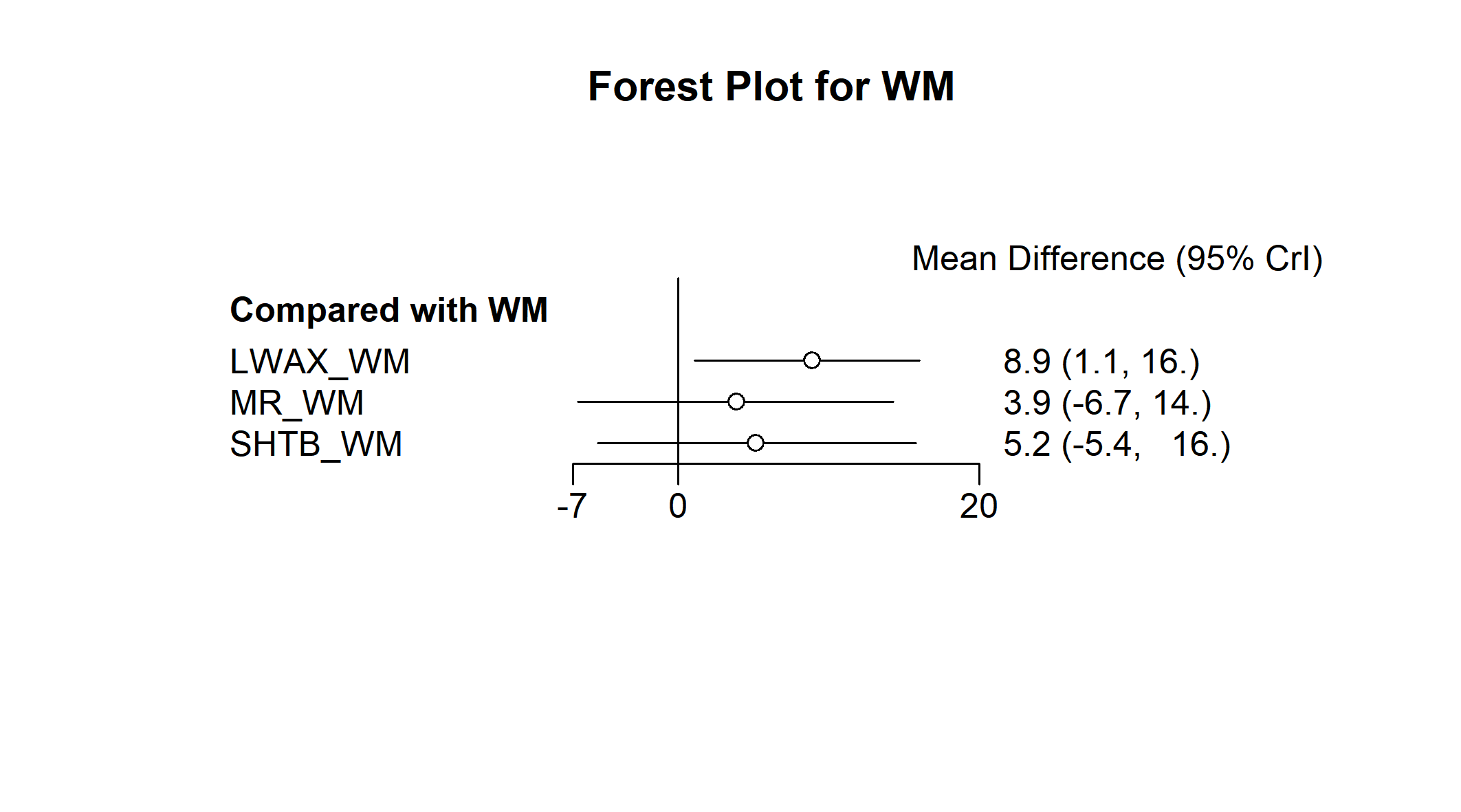

Supplement: Supplementary file 1 [file Data_Sheet_1.zip › Supplementary_Material/Supplementary Figure/GAS/GASforest_WM.tiff]

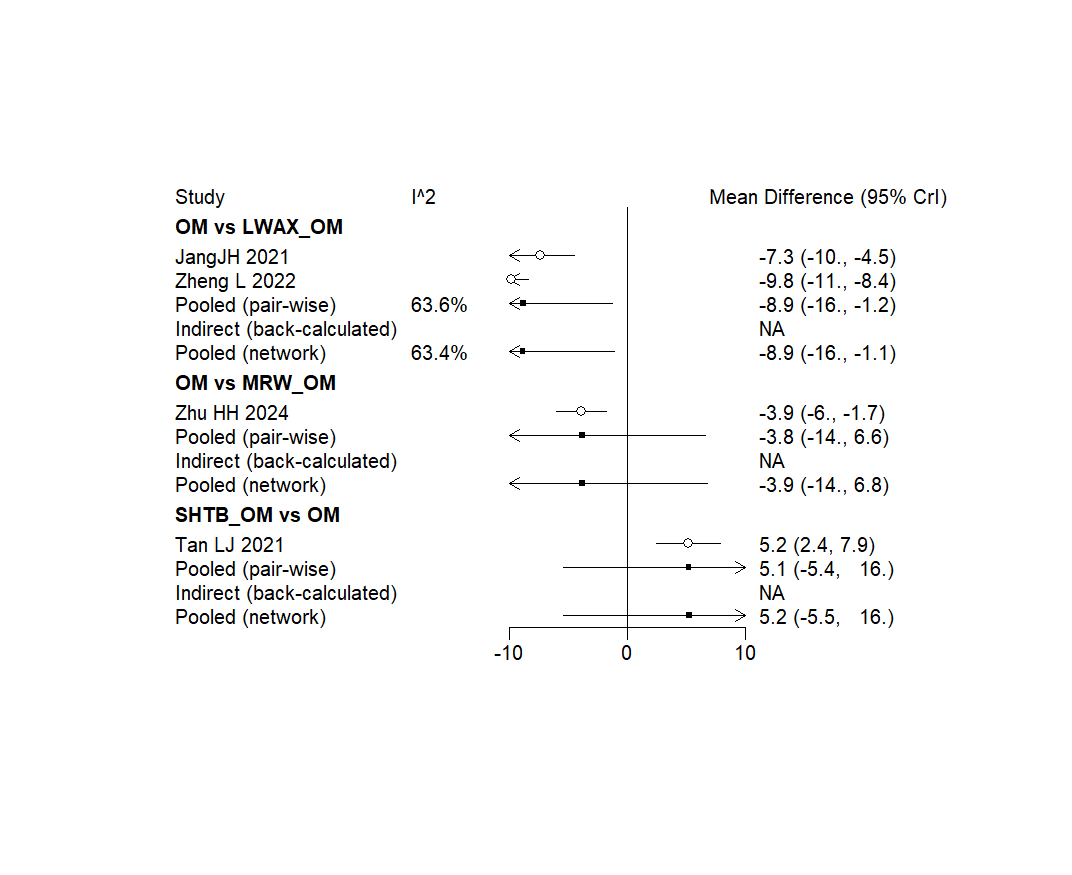

Supplement: Supplementary file 1 [file Data_Sheet_1.zip › Supplementary_Material/Supplementary Figure/GAS/Rplot02.tiff]

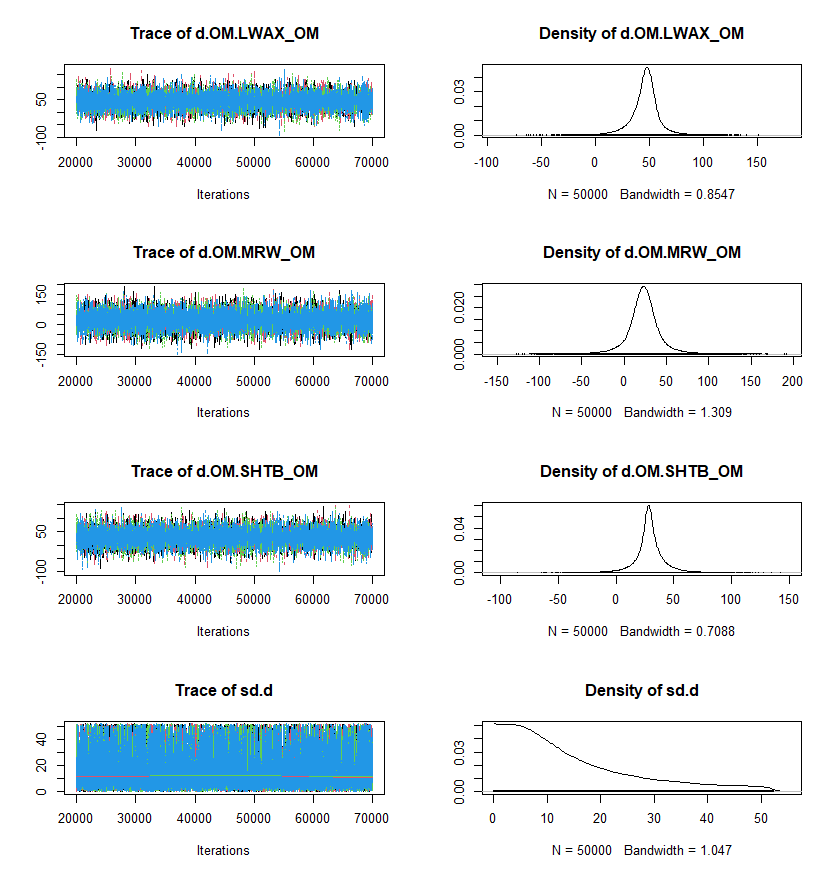

Supplement: Supplementary file 1 [file Data_Sheet_1.zip › Supplementary_Material/Supplementary Figure/MTL/Figure 1.tiff]

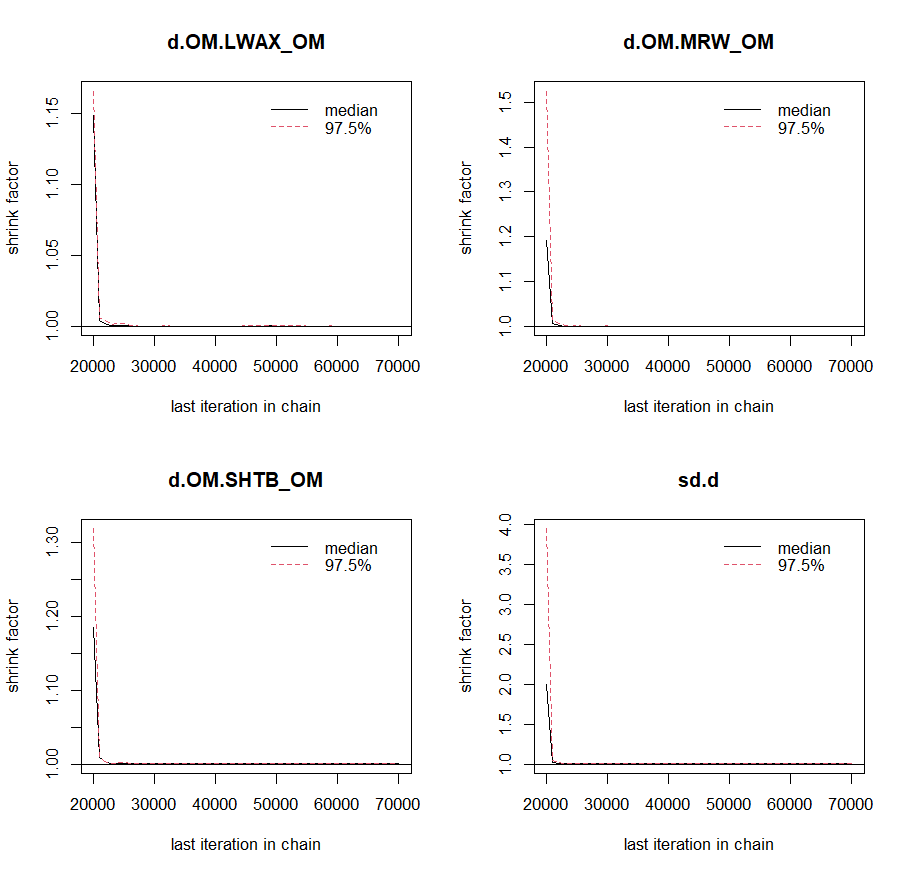

Supplement: Supplementary file 1 [file Data_Sheet_1.zip › Supplementary_Material/Supplementary Figure/MTL/Figure 2.tiff]

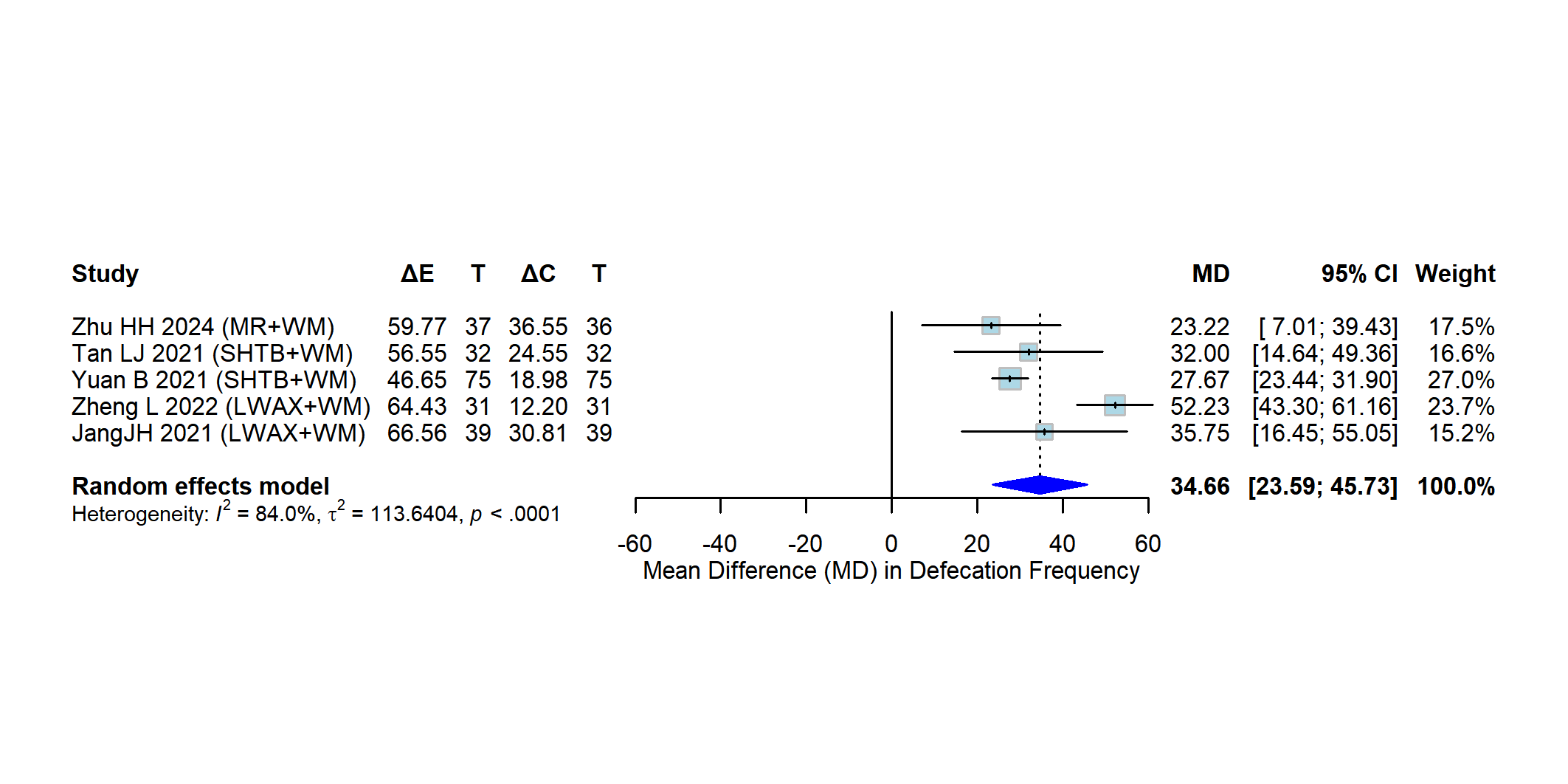

Supplement: Supplementary file 1 [file Data_Sheet_1.zip › Supplementary_Material/Supplementary Figure/MTL/forest_plot_MD.tiff]

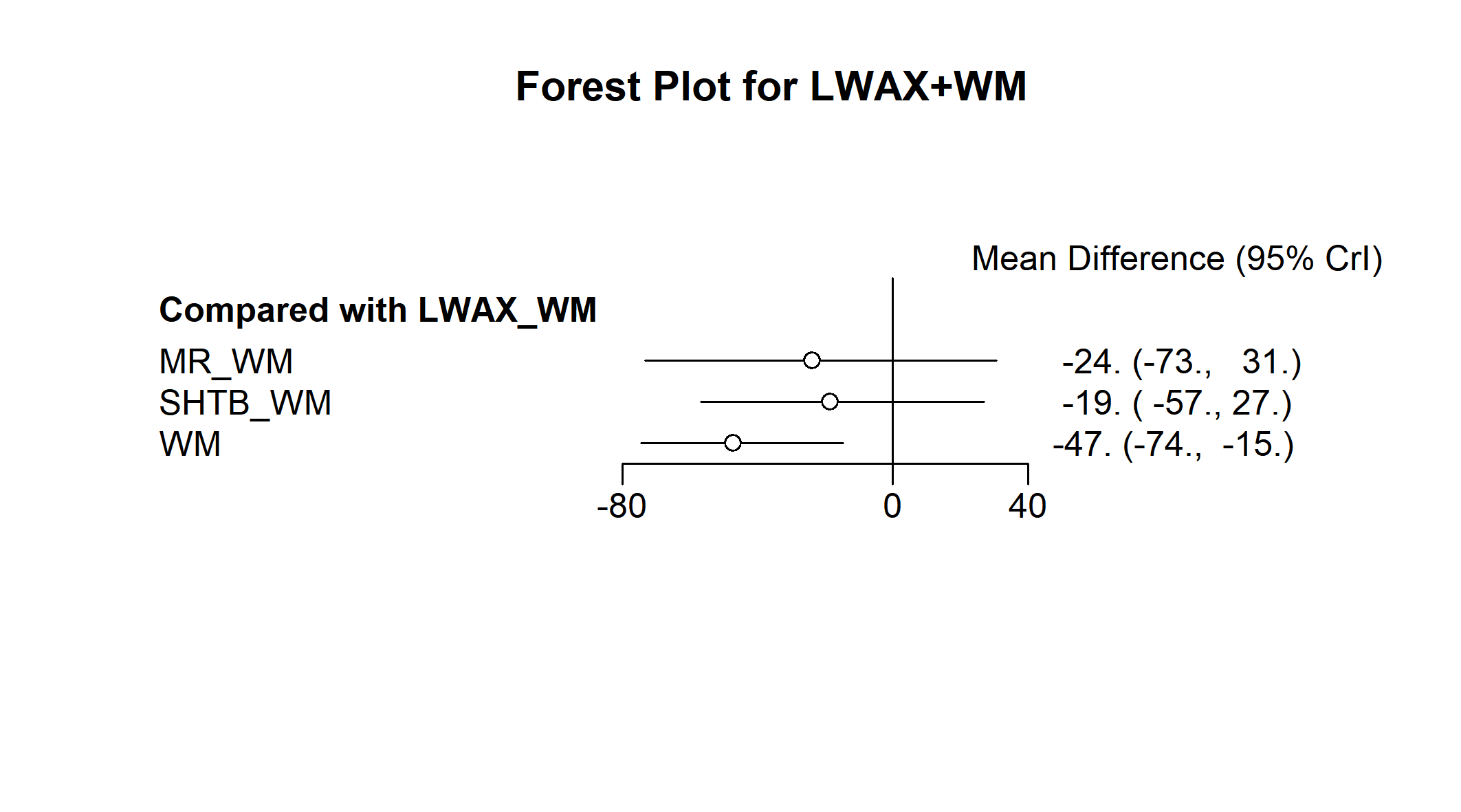

Supplement: Supplementary file 1 [file Data_Sheet_1.zip › Supplementary_Material/Supplementary Figure/MTL/MTLforest_LWAX+WM.tiff]

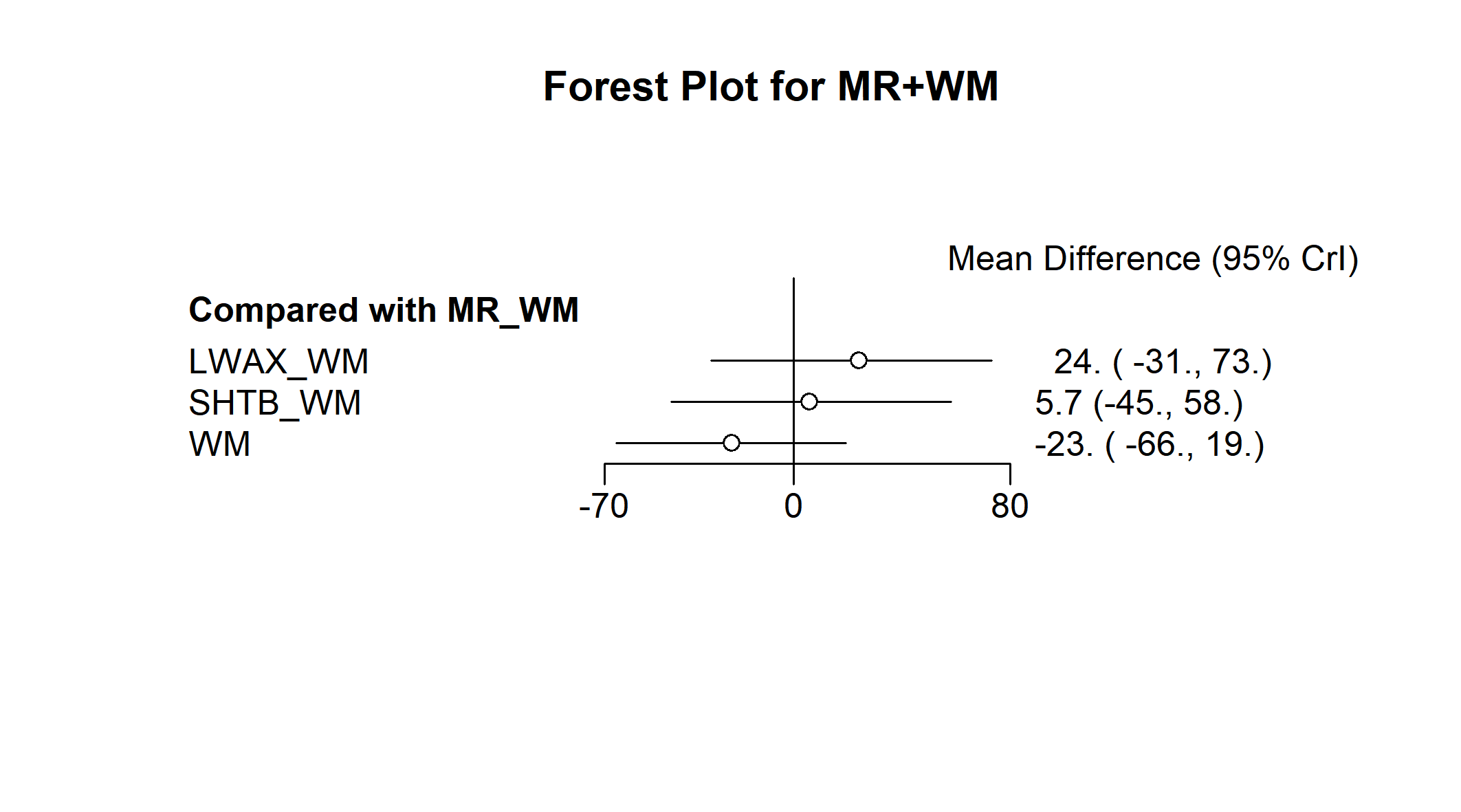

Supplement: Supplementary file 1 [file Data_Sheet_1.zip › Supplementary_Material/Supplementary Figure/MTL/MTLforest_MR+WM.tiff]

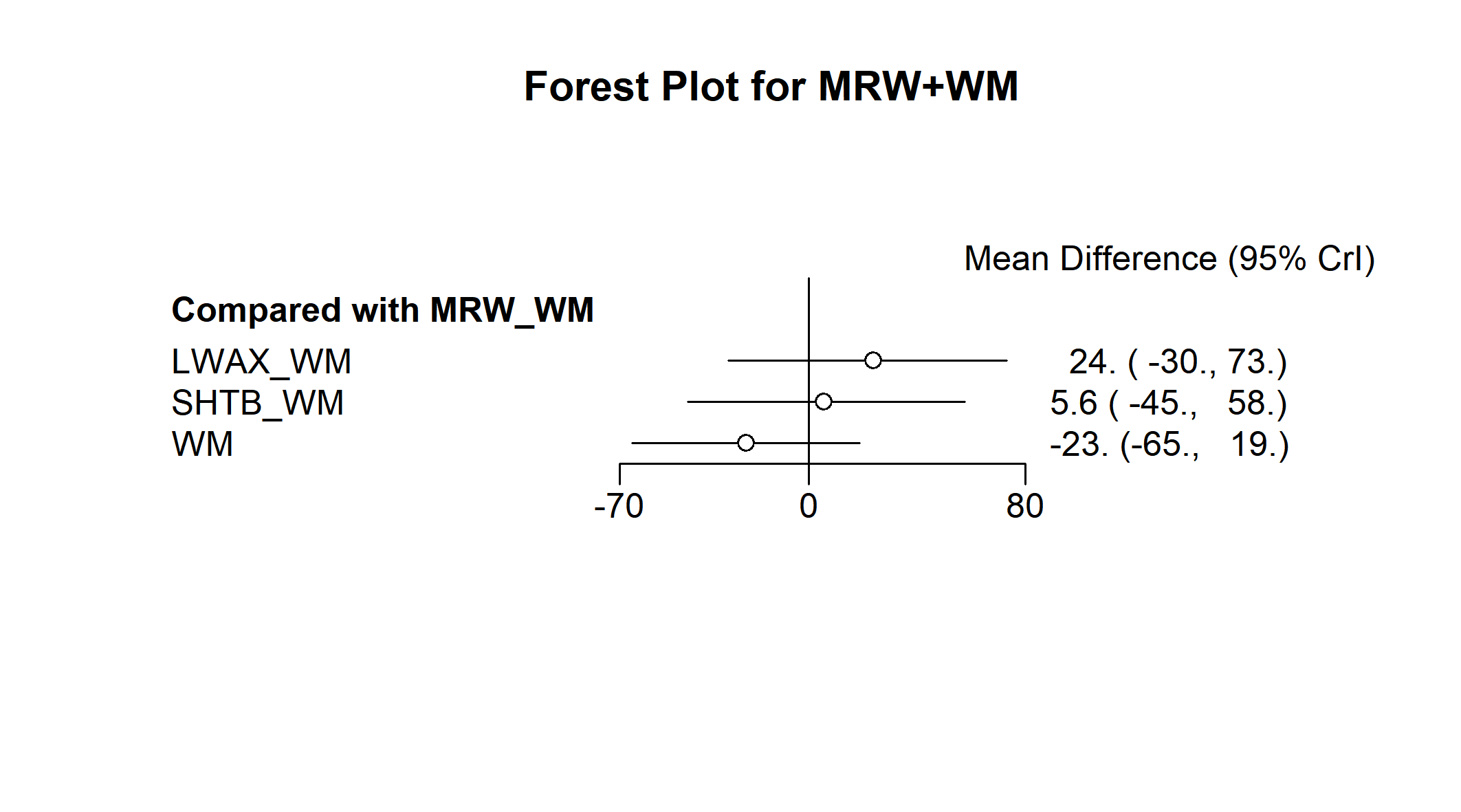

Supplement: Supplementary file 1 [file Data_Sheet_1.zip › Supplementary_Material/Supplementary Figure/MTL/MTLforest_MRW+WM.tiff]

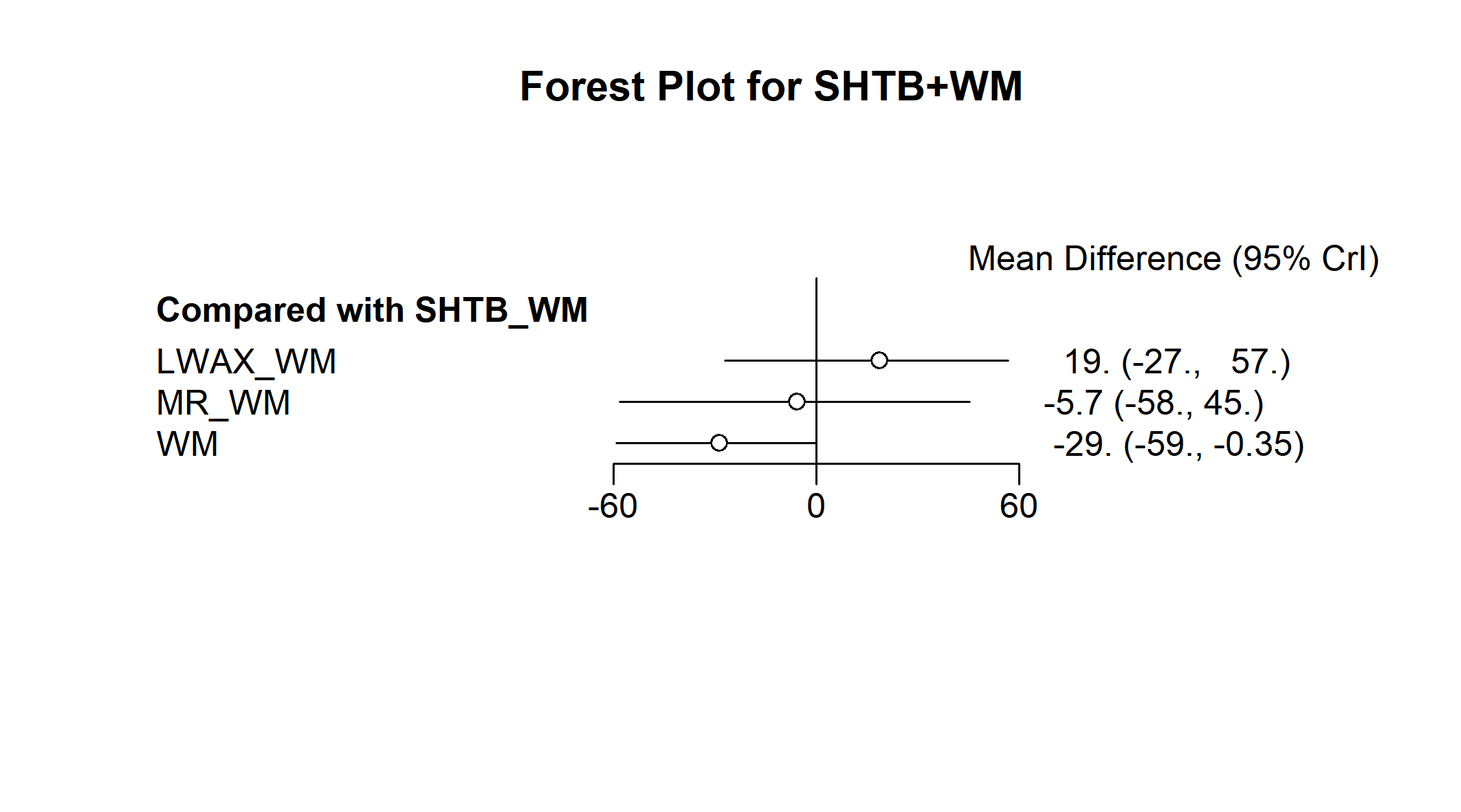

Supplement: Supplementary file 1 [file Data_Sheet_1.zip › Supplementary_Material/Supplementary Figure/MTL/MTLforest_SHTB+WM.tiff]

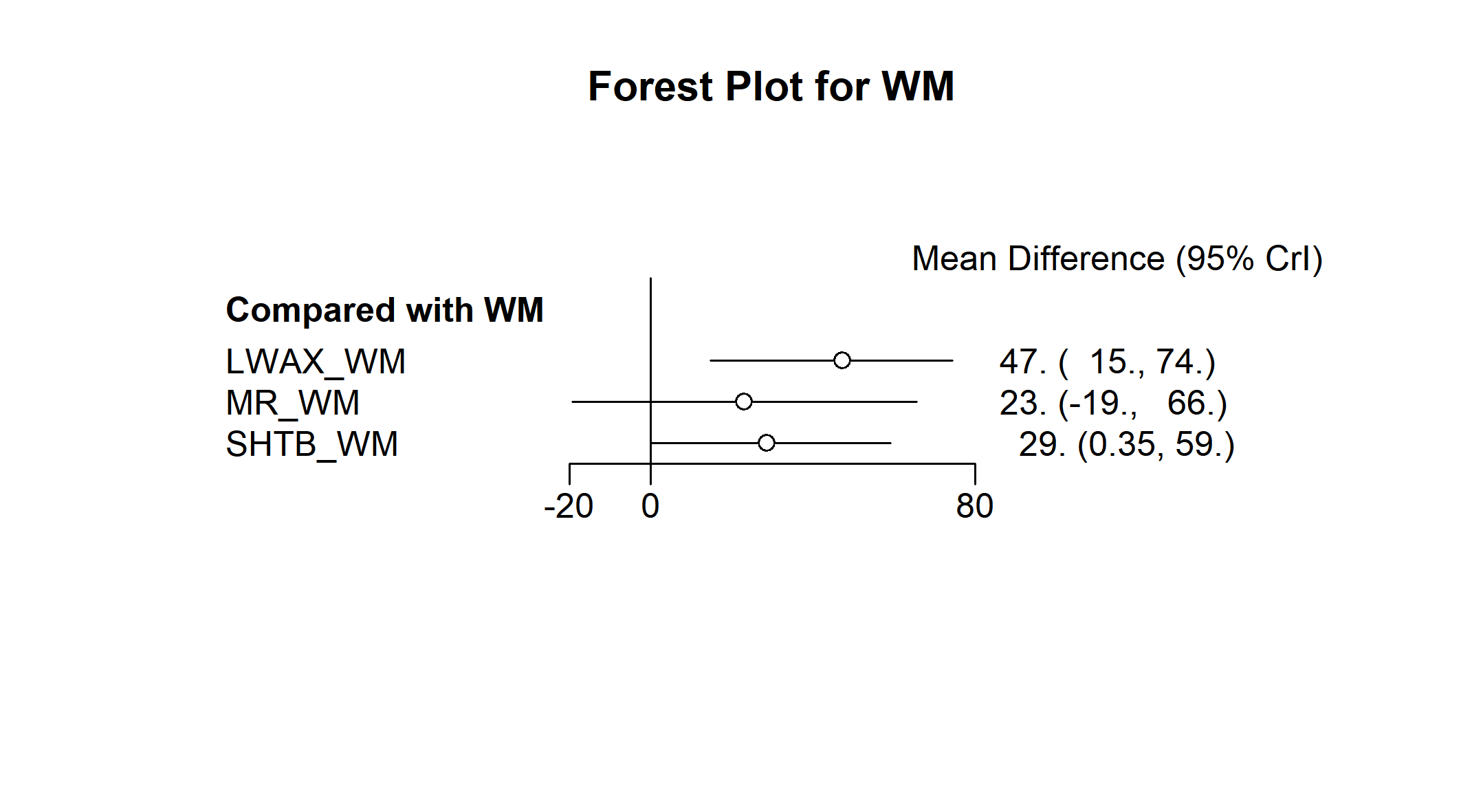

Supplement: Supplementary file 1 [file Data_Sheet_1.zip › Supplementary_Material/Supplementary Figure/MTL/MTLforest_WM.tiff]

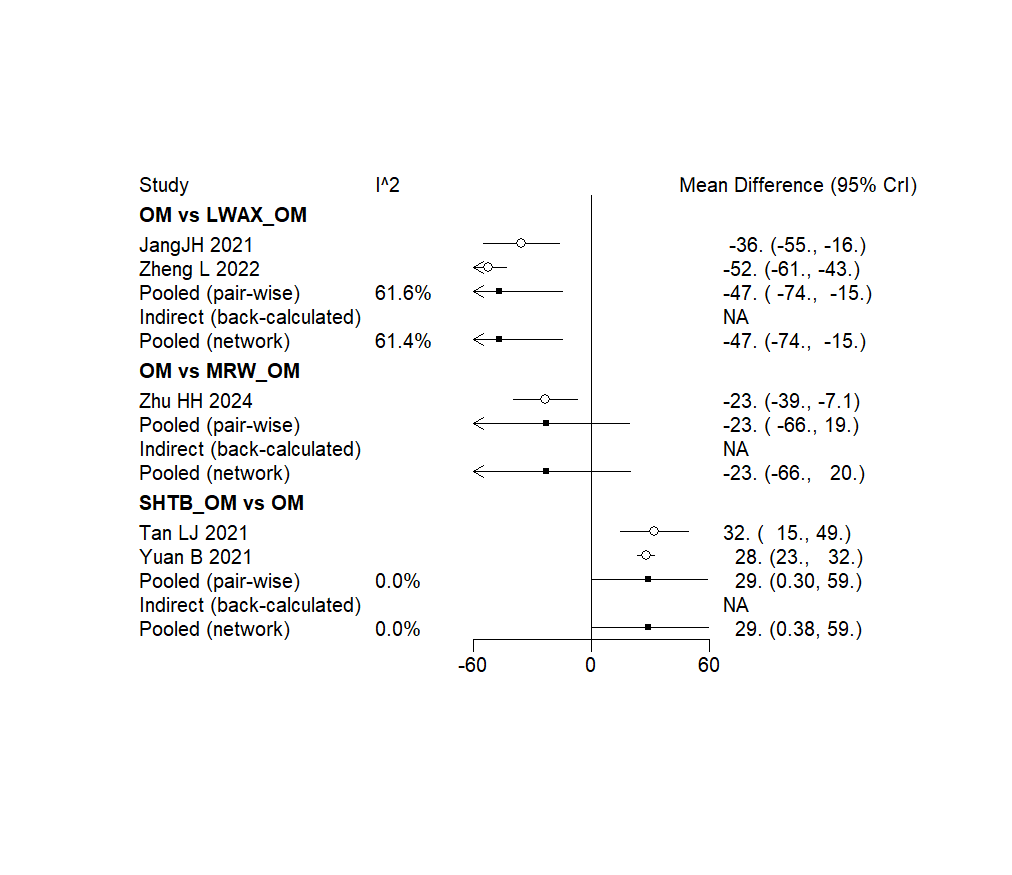

Supplement: Supplementary file 1 [file Data_Sheet_1.zip › Supplementary_Material/Supplementary Figure/MTL/Rplot03.tiff]

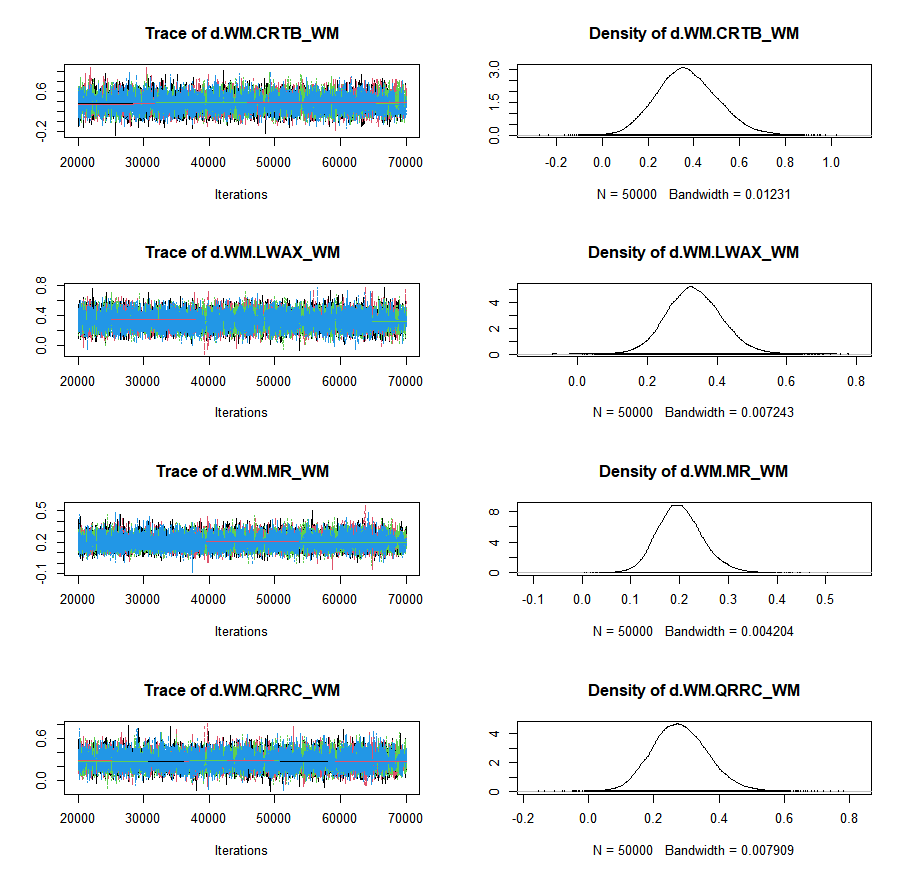

Supplement: Supplementary file 1 [file Data_Sheet_1.zip › Supplementary_Material/Supplementary Figure/overall clinical efficacy rate/Figure 1.tiff]

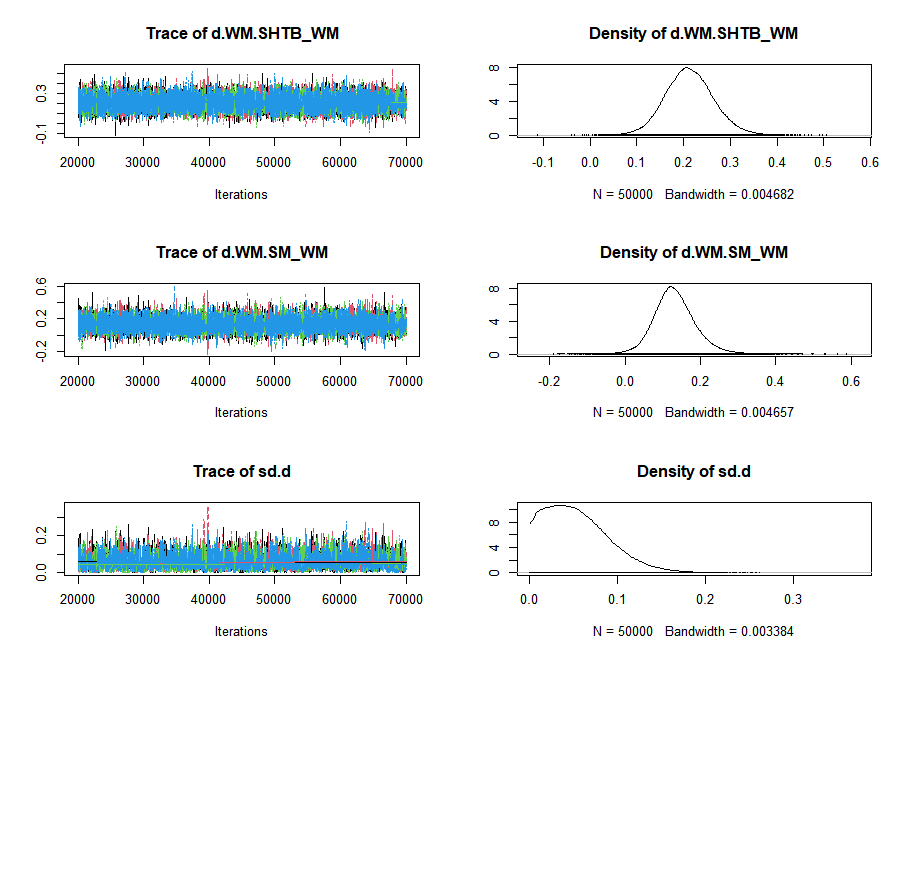

Supplement: Supplementary file 1 [file Data_Sheet_1.zip › Supplementary_Material/Supplementary Figure/overall clinical efficacy rate/Figure 2.tiff]

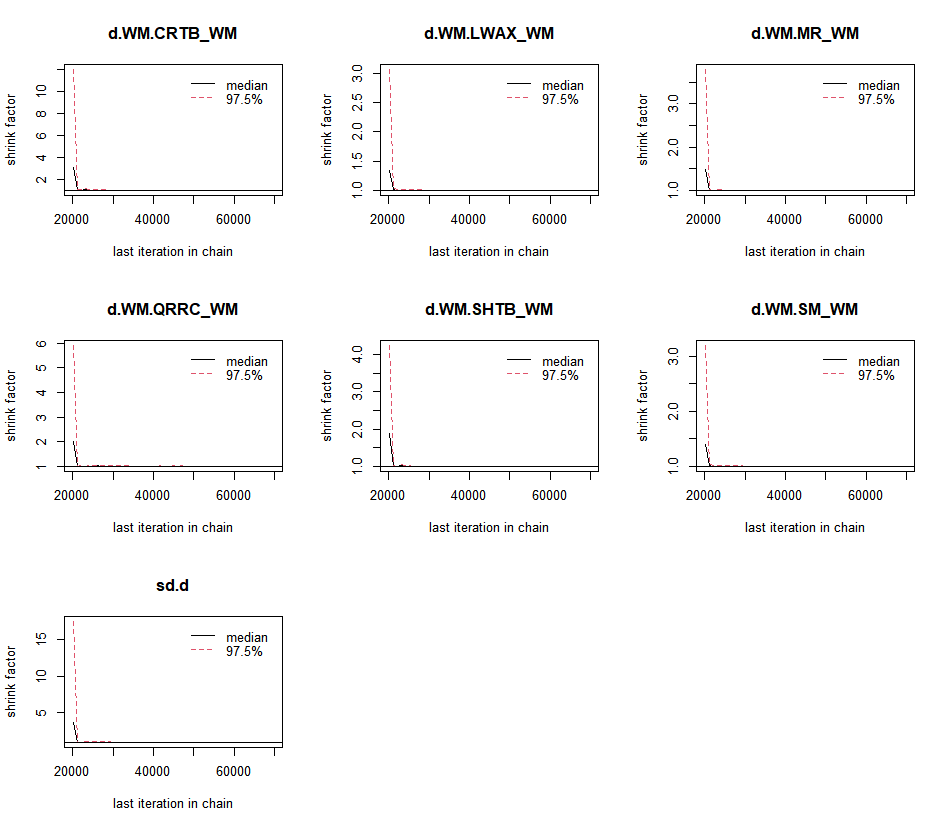

Supplement: Supplementary file 1 [file Data_Sheet_1.zip › Supplementary_Material/Supplementary Figure/overall clinical efficacy rate/Figure 3.tiff]

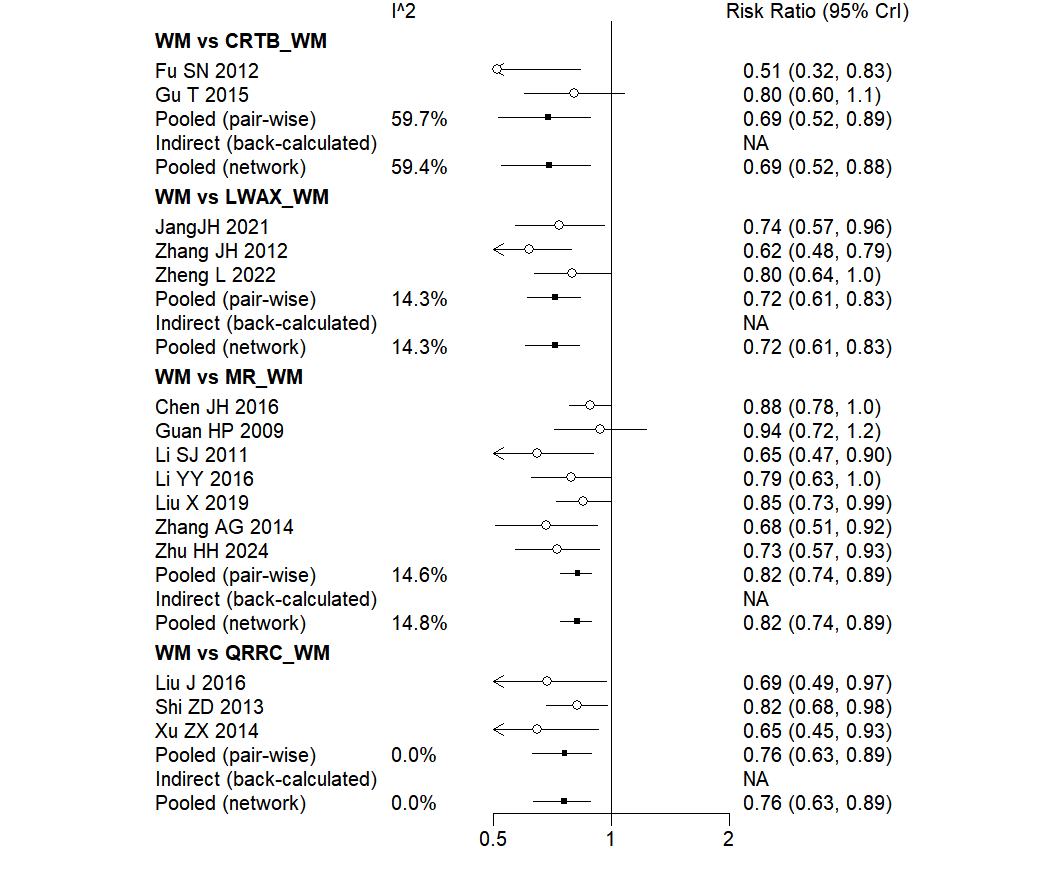

Supplement: Supplementary file 1 [file Data_Sheet_1.zip › Supplementary_Material/Supplementary Figure/overall clinical efficacy rate/Figure 4.tiff]

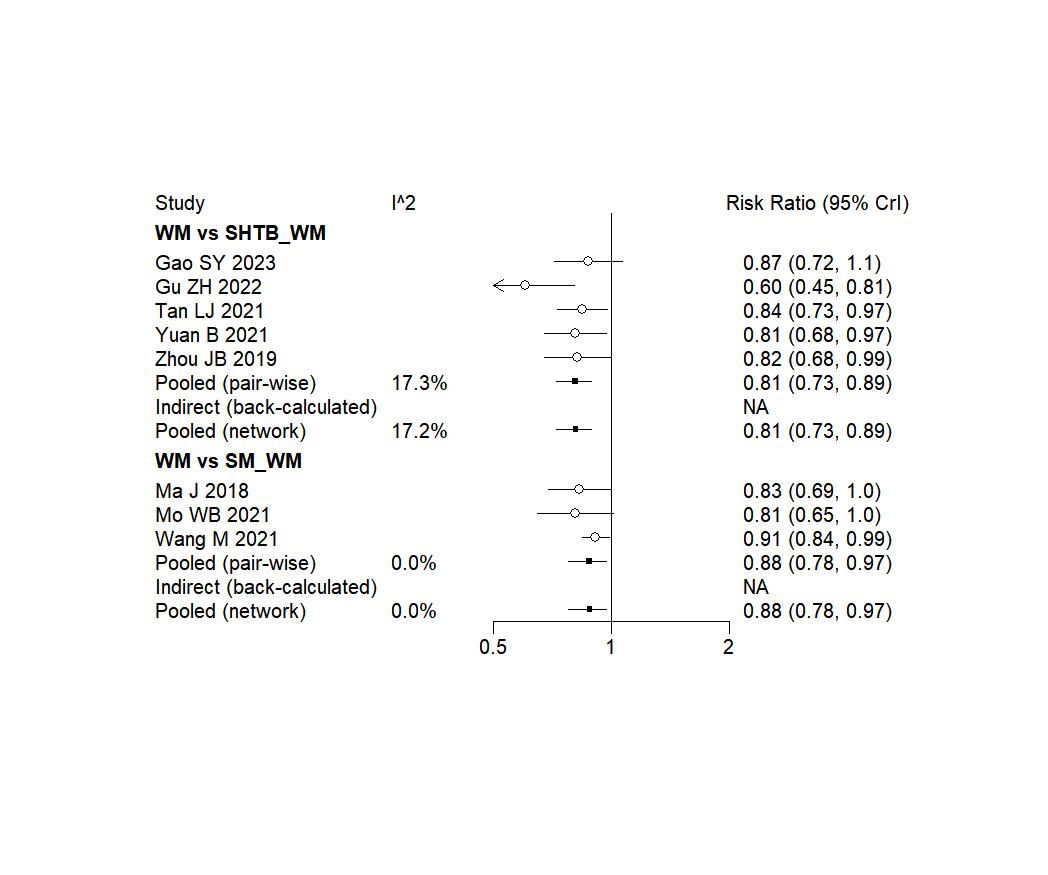

Supplement: Supplementary file 1 [file Data_Sheet_1.zip › Supplementary_Material/Supplementary Figure/overall clinical efficacy rate/Figure 5.tiff]

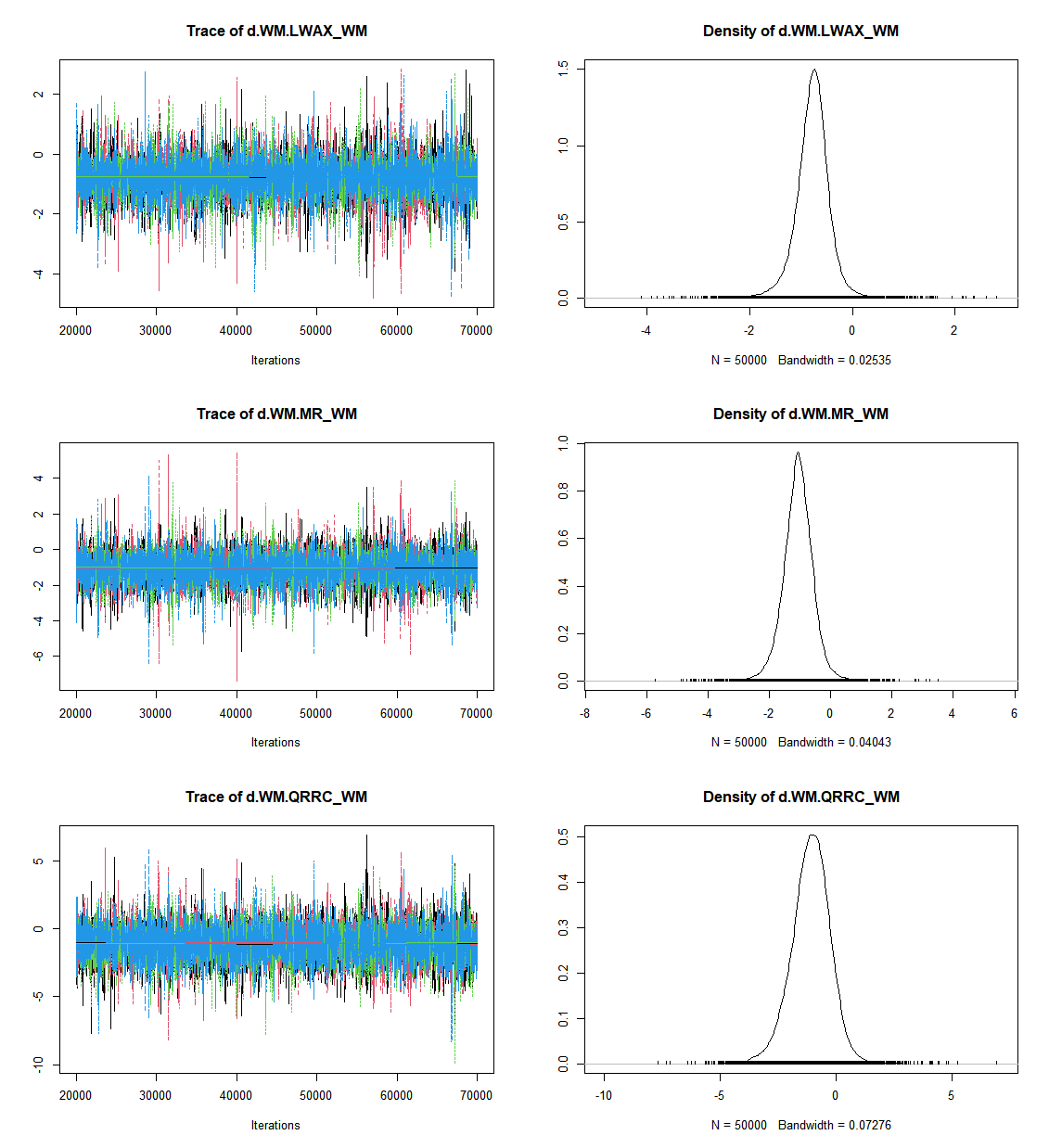

Supplement: Supplementary file 1 [file Data_Sheet_1.zip › Supplementary_Material/Supplementary Figure/recurrence rate/Figure 1.tiff]

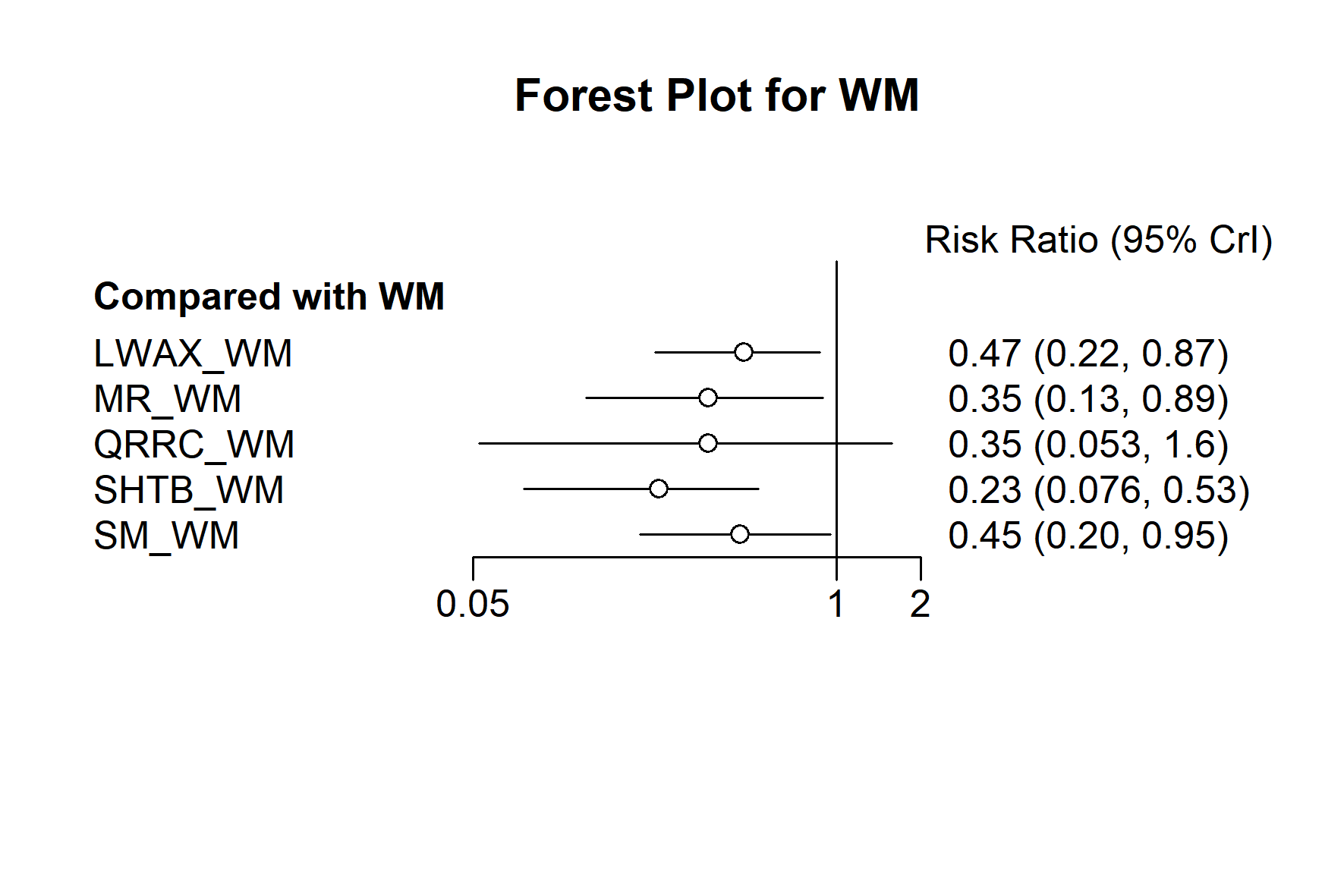

Supplement: Supplementary file 1 [file Data_Sheet_1.zip › Supplementary_Material/Supplementary Figure/recurrence rate/Figure 10.tiff]

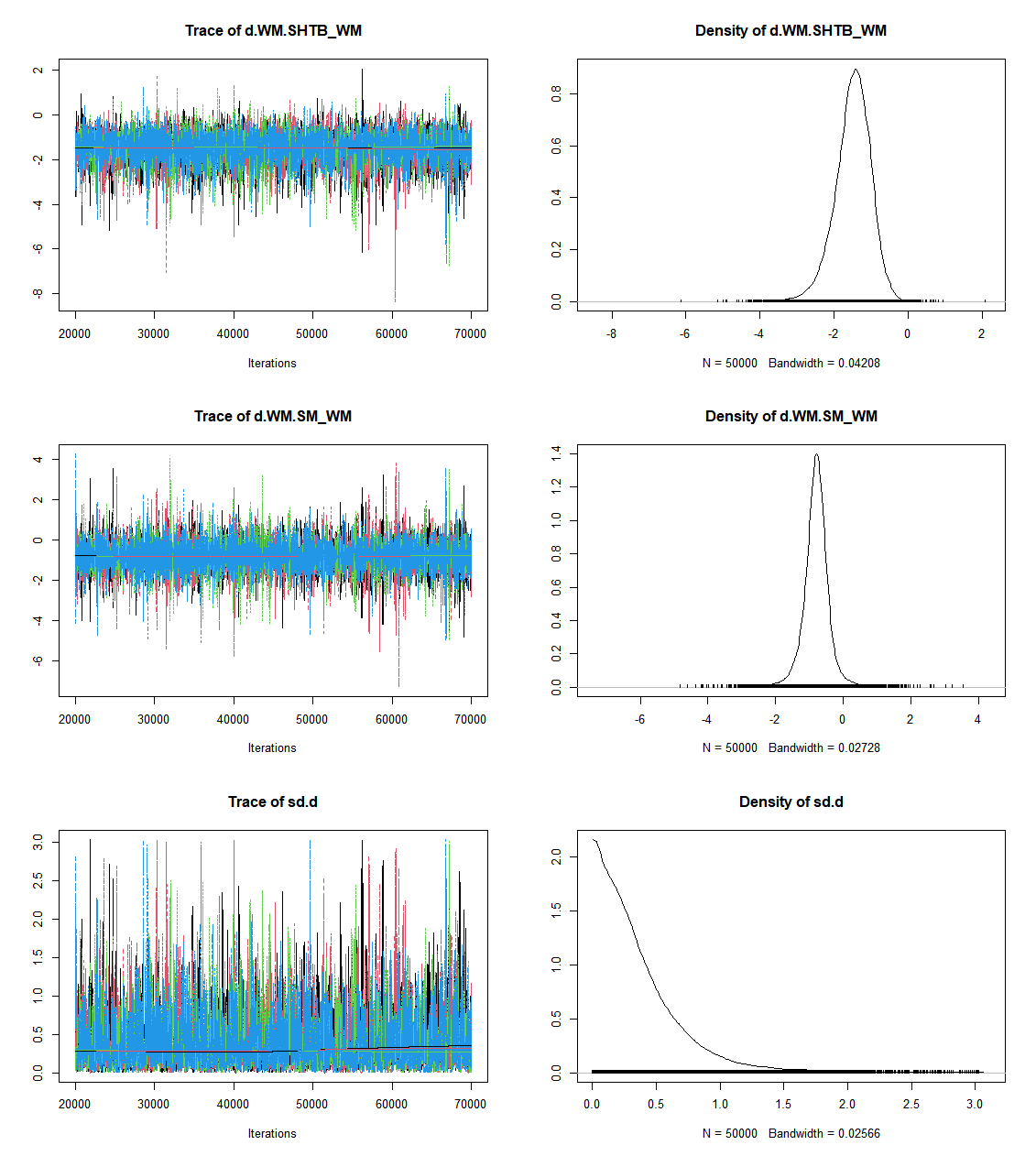

Supplement: Supplementary file 1 [file Data_Sheet_1.zip › Supplementary_Material/Supplementary Figure/recurrence rate/Figure 2.tiff]

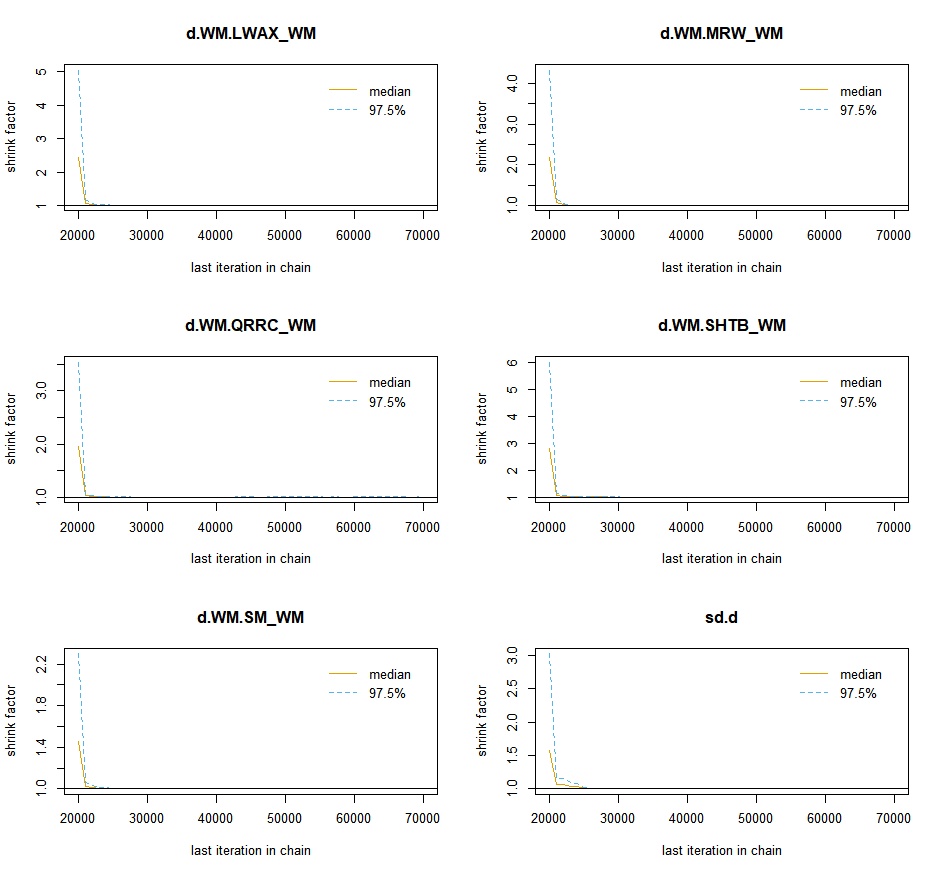

Supplement: Supplementary file 1 [file Data_Sheet_1.zip › Supplementary_Material/Supplementary Figure/recurrence rate/Figure 3.tiff]

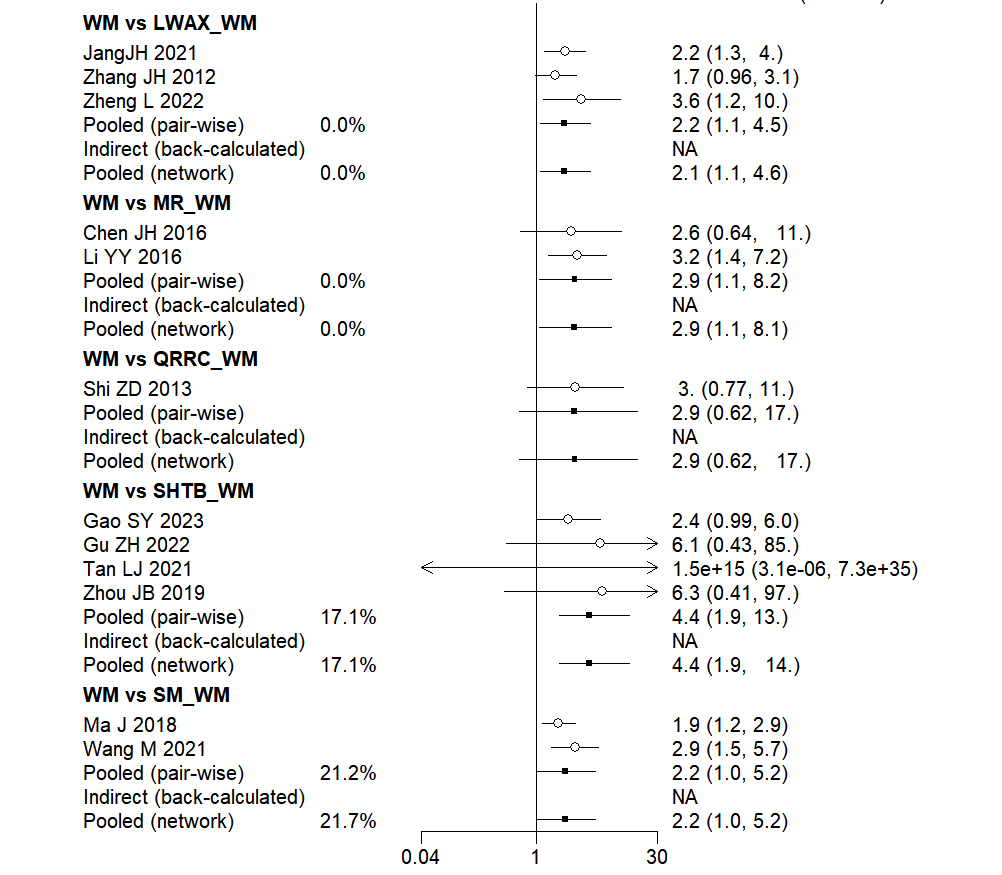

Supplement: Supplementary file 1 [file Data_Sheet_1.zip › Supplementary_Material/Supplementary Figure/recurrence rate/Figure 4.tiff]

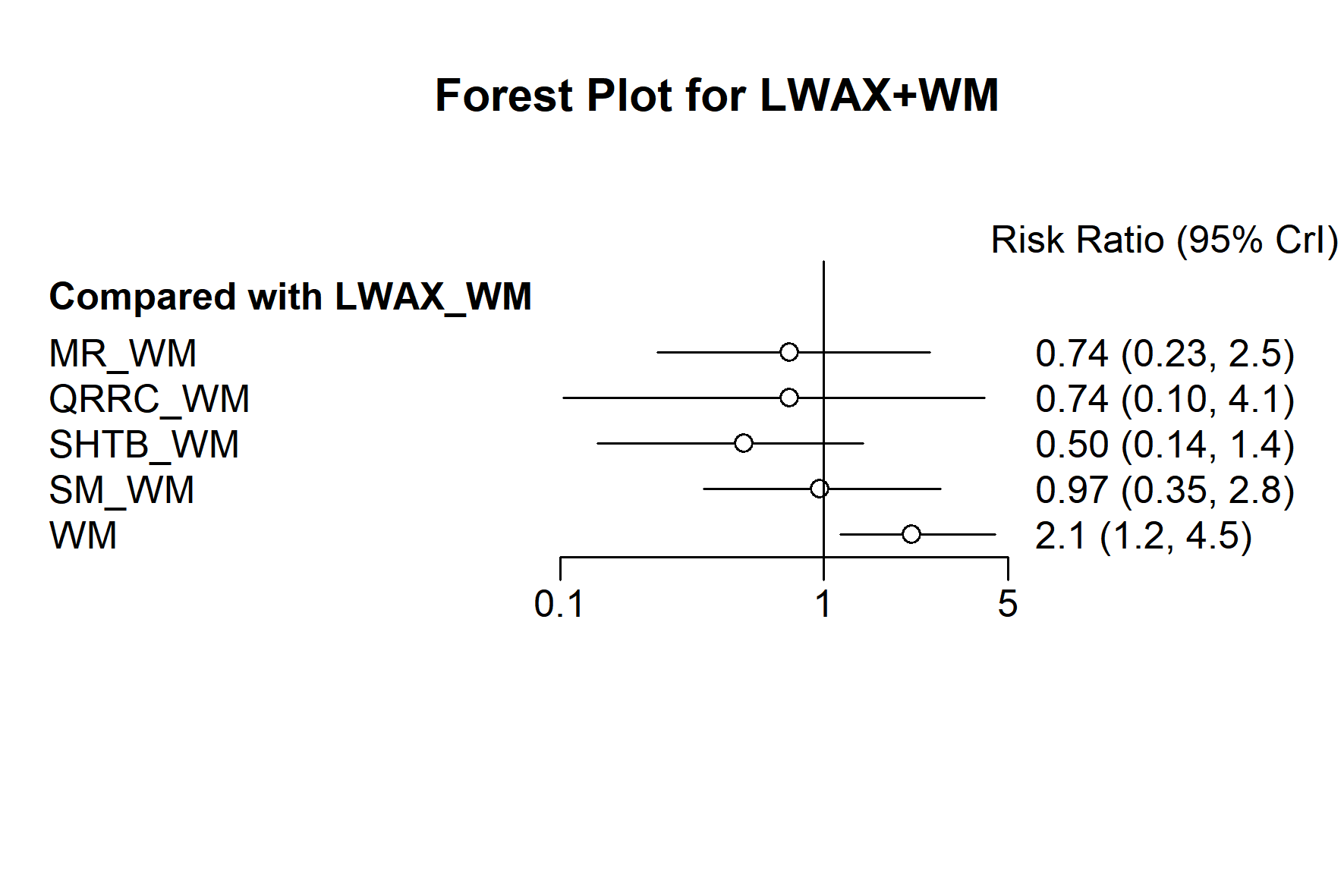

Supplement: Supplementary file 1 [file Data_Sheet_1.zip › Supplementary_Material/Supplementary Figure/recurrence rate/Figure 5.tiff]

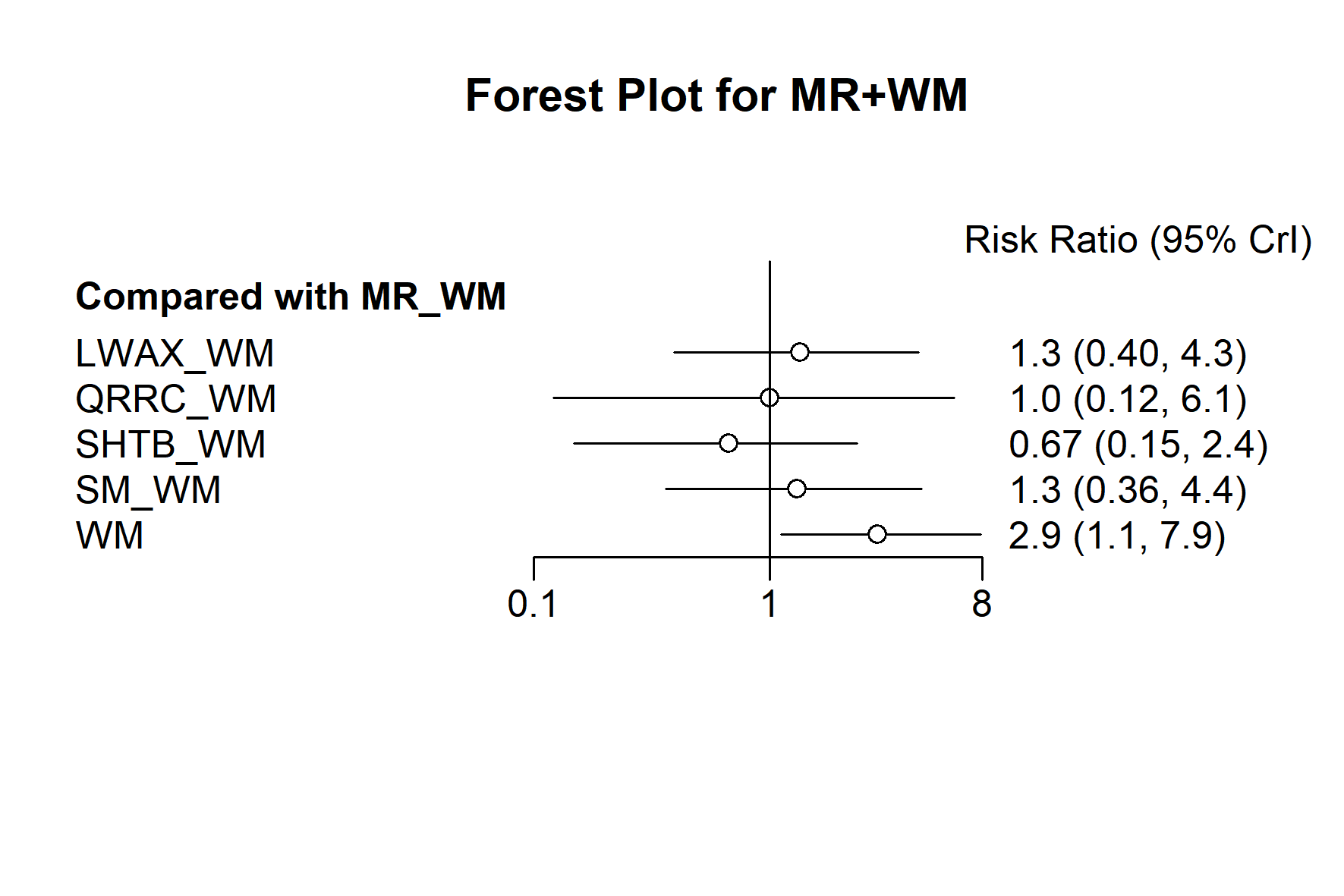

Supplement: Supplementary file 1 [file Data_Sheet_1.zip › Supplementary_Material/Supplementary Figure/recurrence rate/Figure 6.tiff]

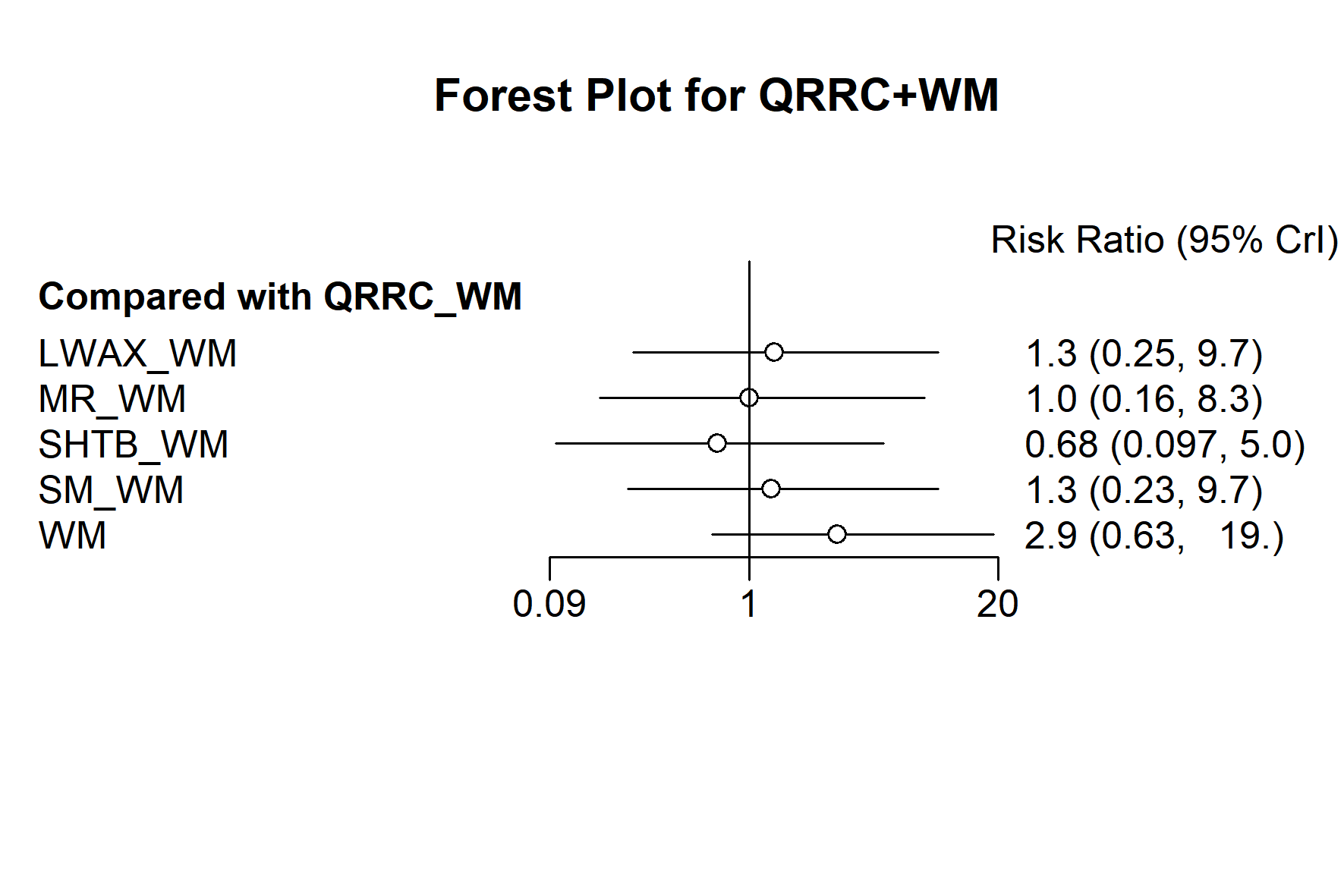

Supplement: Supplementary file 1 [file Data_Sheet_1.zip › Supplementary_Material/Supplementary Figure/recurrence rate/Figure 7.tiff]

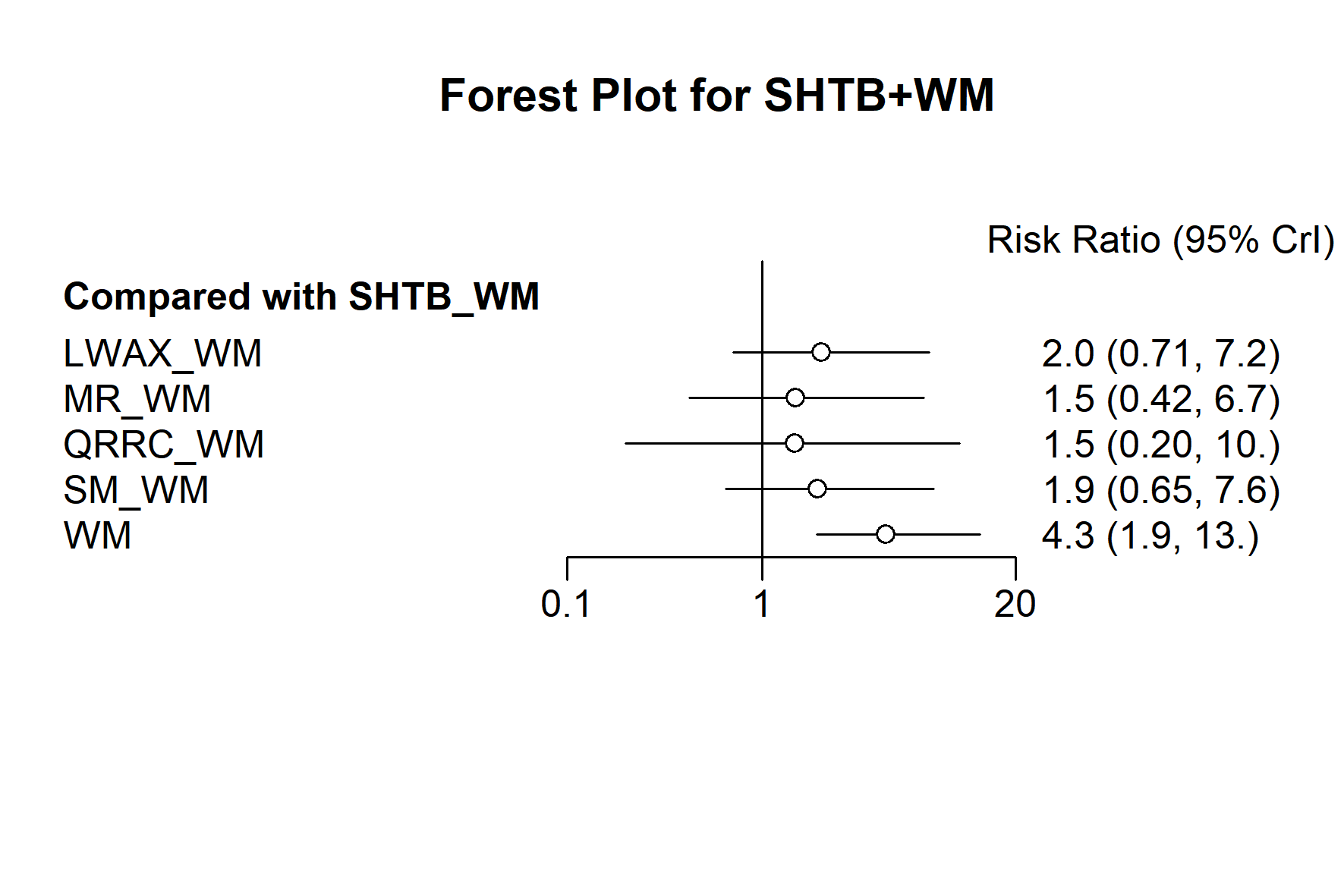

Supplement: Supplementary file 1 [file Data_Sheet_1.zip › Supplementary_Material/Supplementary Figure/recurrence rate/Figure 8.tiff]

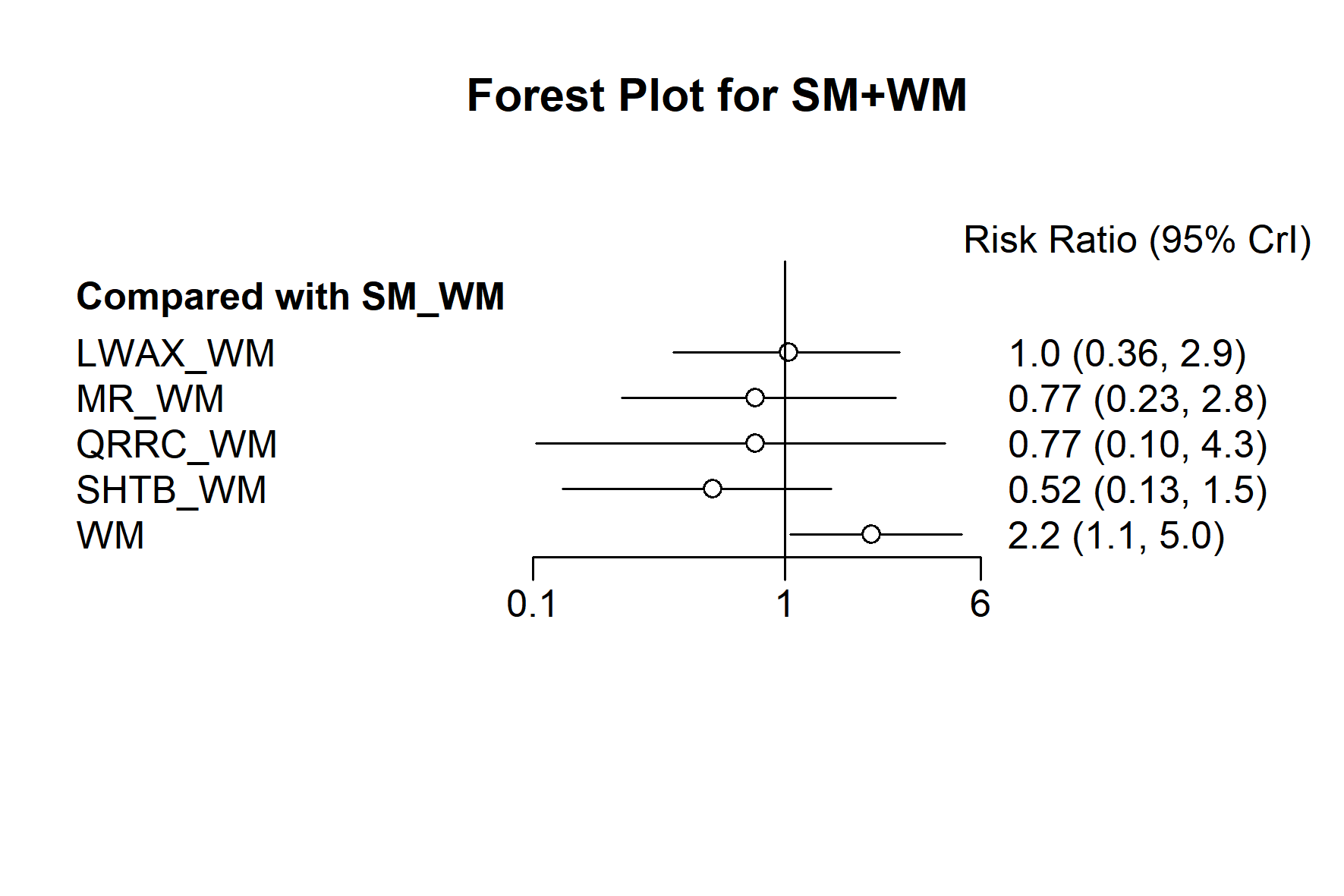

Supplement: Supplementary file 1 [file Data_Sheet_1.zip › Supplementary_Material/Supplementary Figure/recurrence rate/Figure 9.tiff]

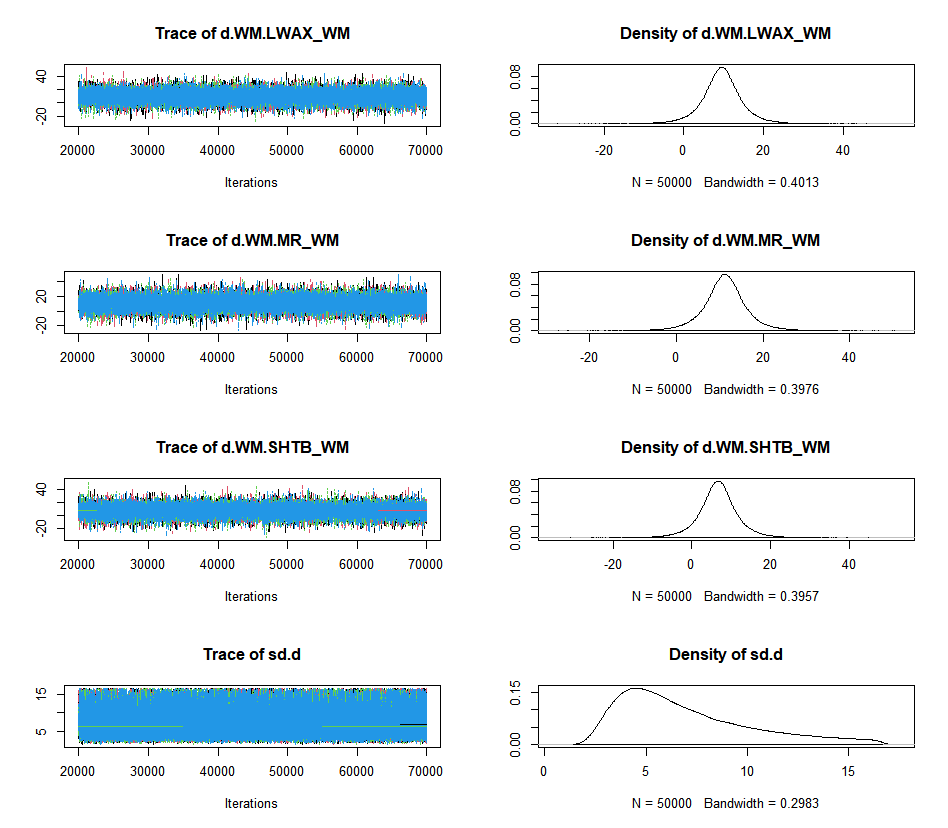

Supplement: Supplementary file 1 [file Data_Sheet_1.zip › Supplementary_Material/Supplementary Figure/SP/Figure 1.tiff]

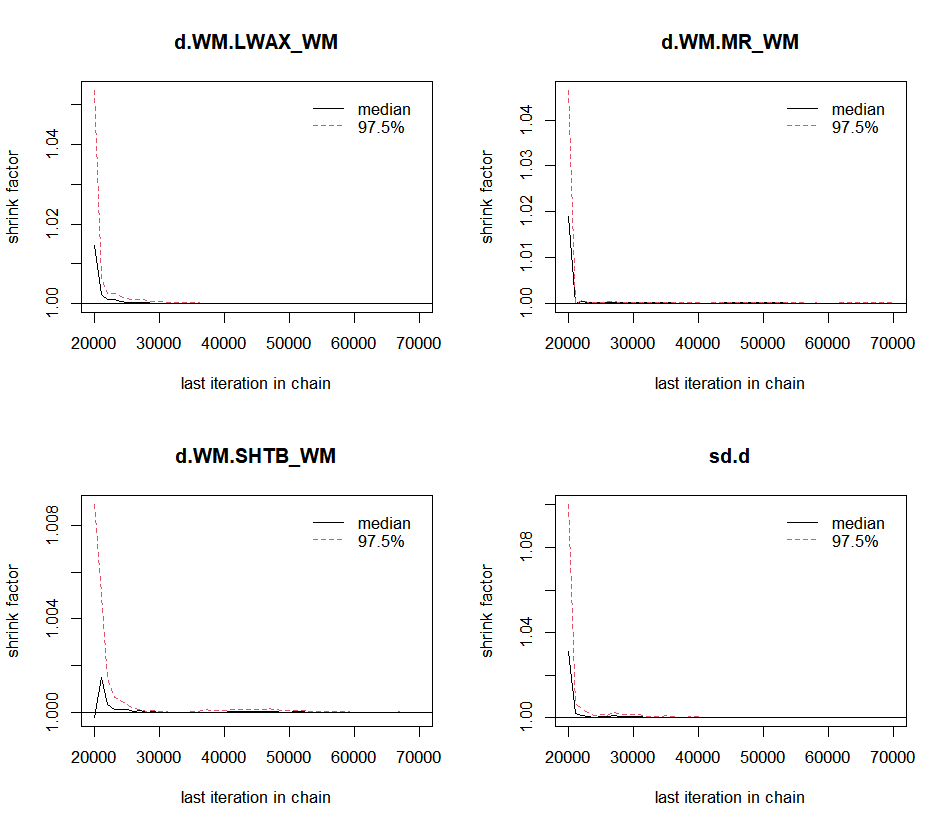

Supplement: Supplementary file 1 [file Data_Sheet_1.zip › Supplementary_Material/Supplementary Figure/SP/Figure 2.tiff]

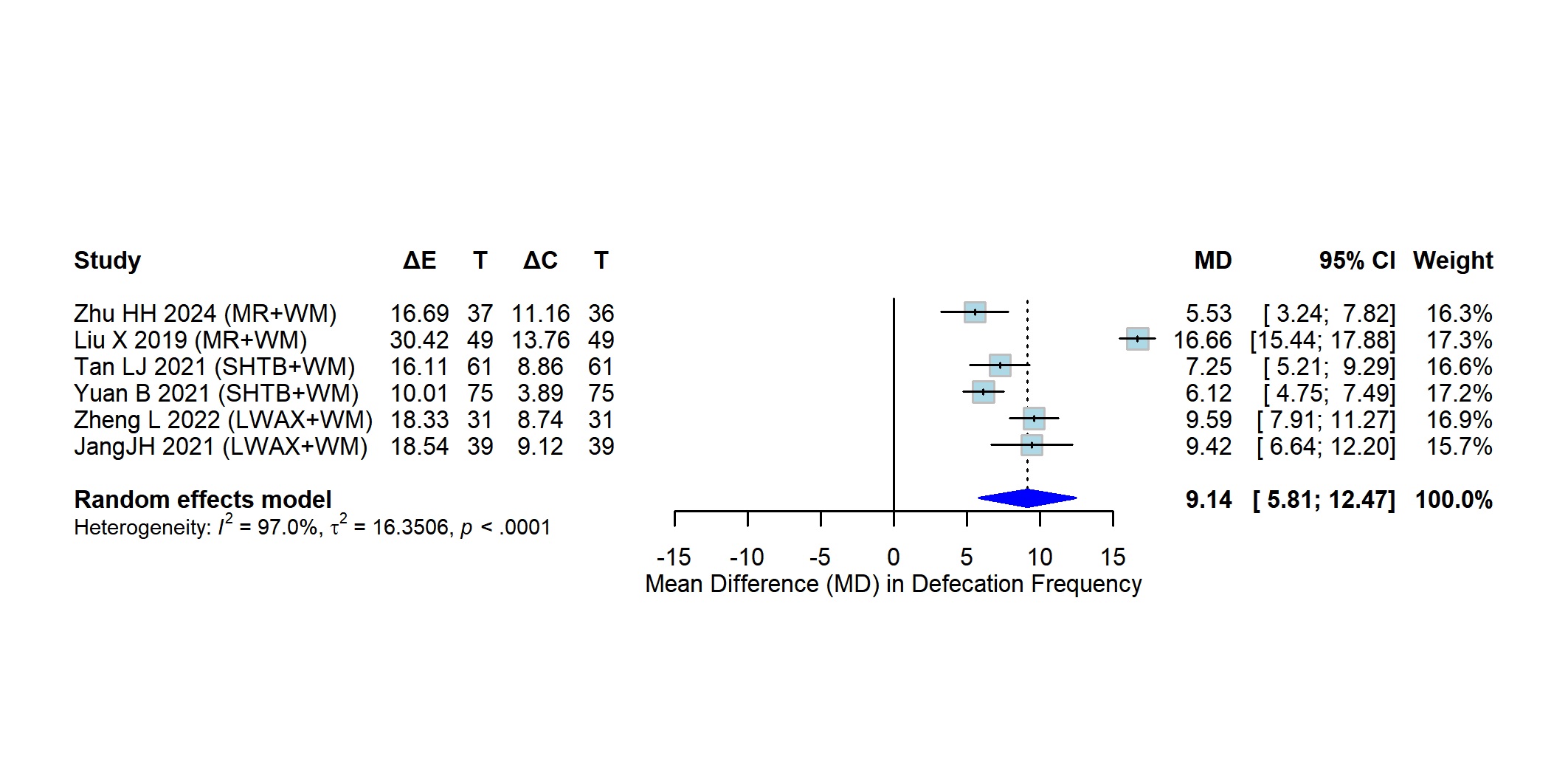

Supplement: Supplementary file 1 [file Data_Sheet_1.zip › Supplementary_Material/Supplementary Figure/SP/forest_plot_MD.tiff]

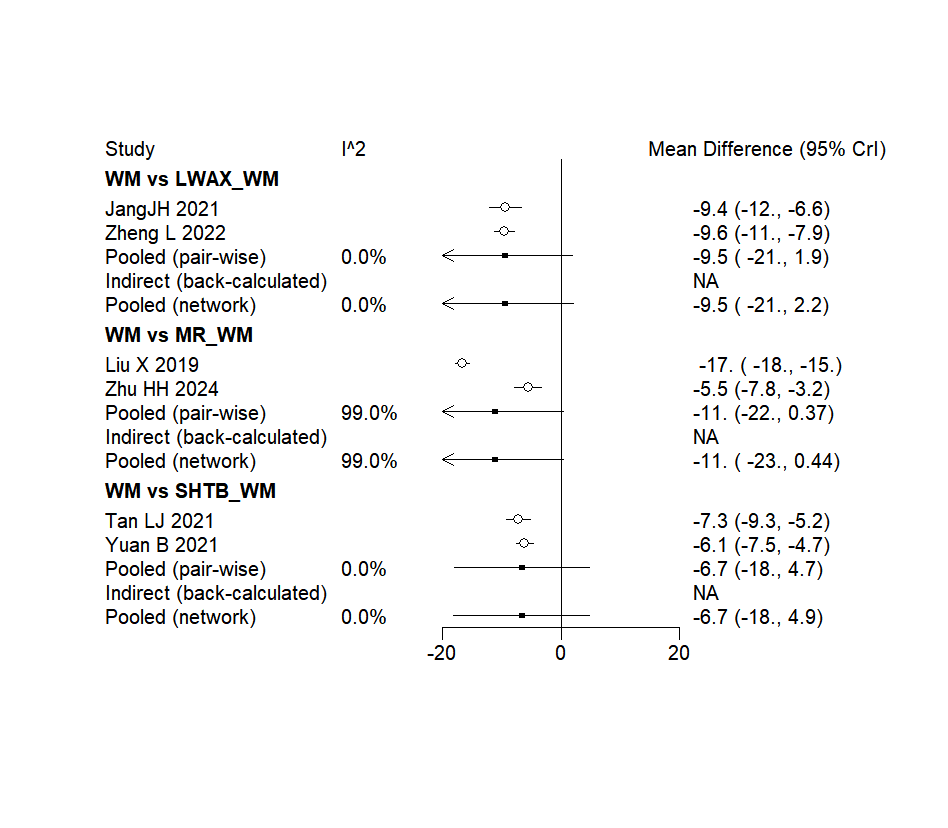

Supplement: Supplementary file 1 [file Data_Sheet_1.zip › Supplementary_Material/Supplementary Figure/SP/Rplot.tiff]

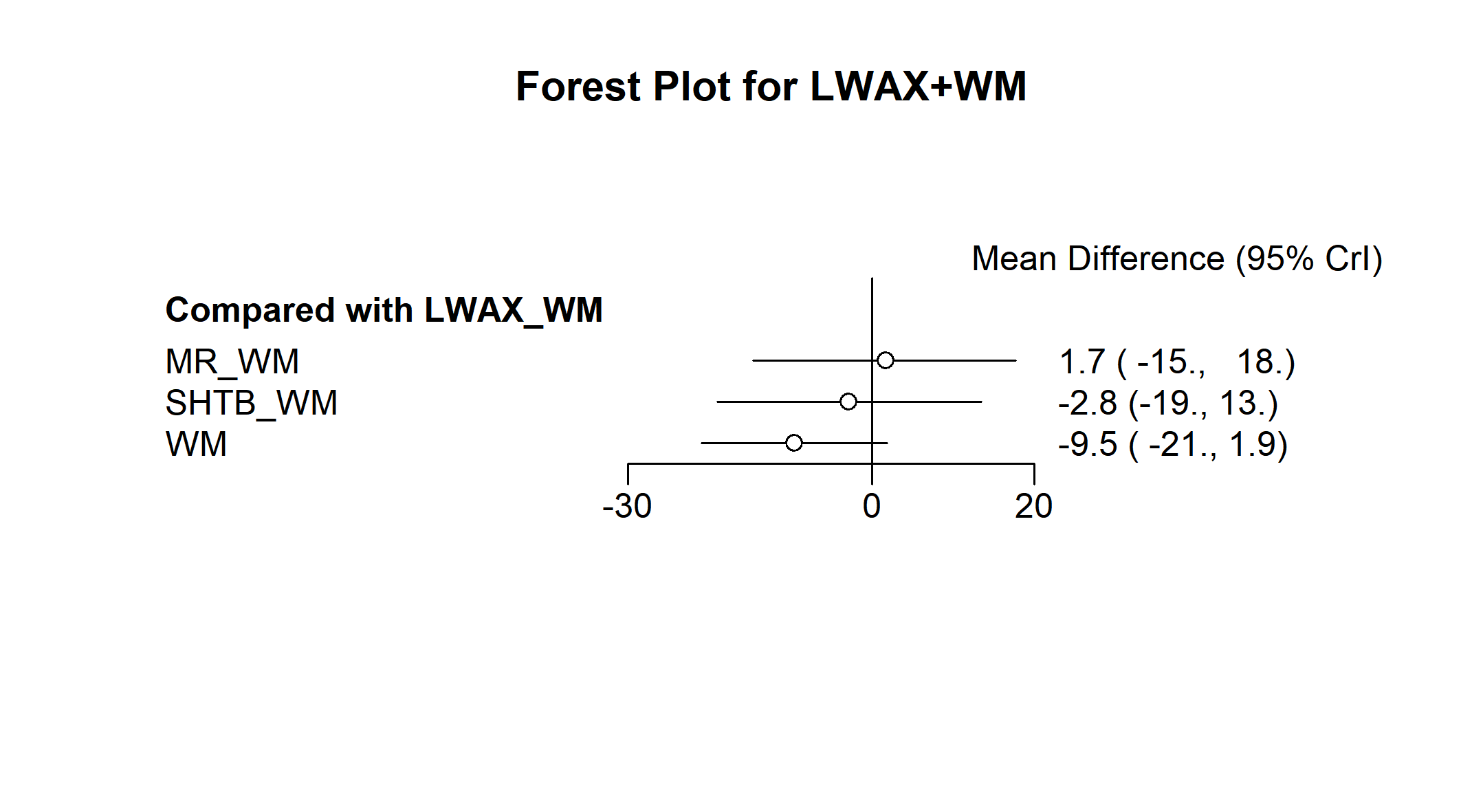

Supplement: Supplementary file 1 [file Data_Sheet_1.zip › Supplementary_Material/Supplementary Figure/SP/SPforest_LWAX+WM.tiff]

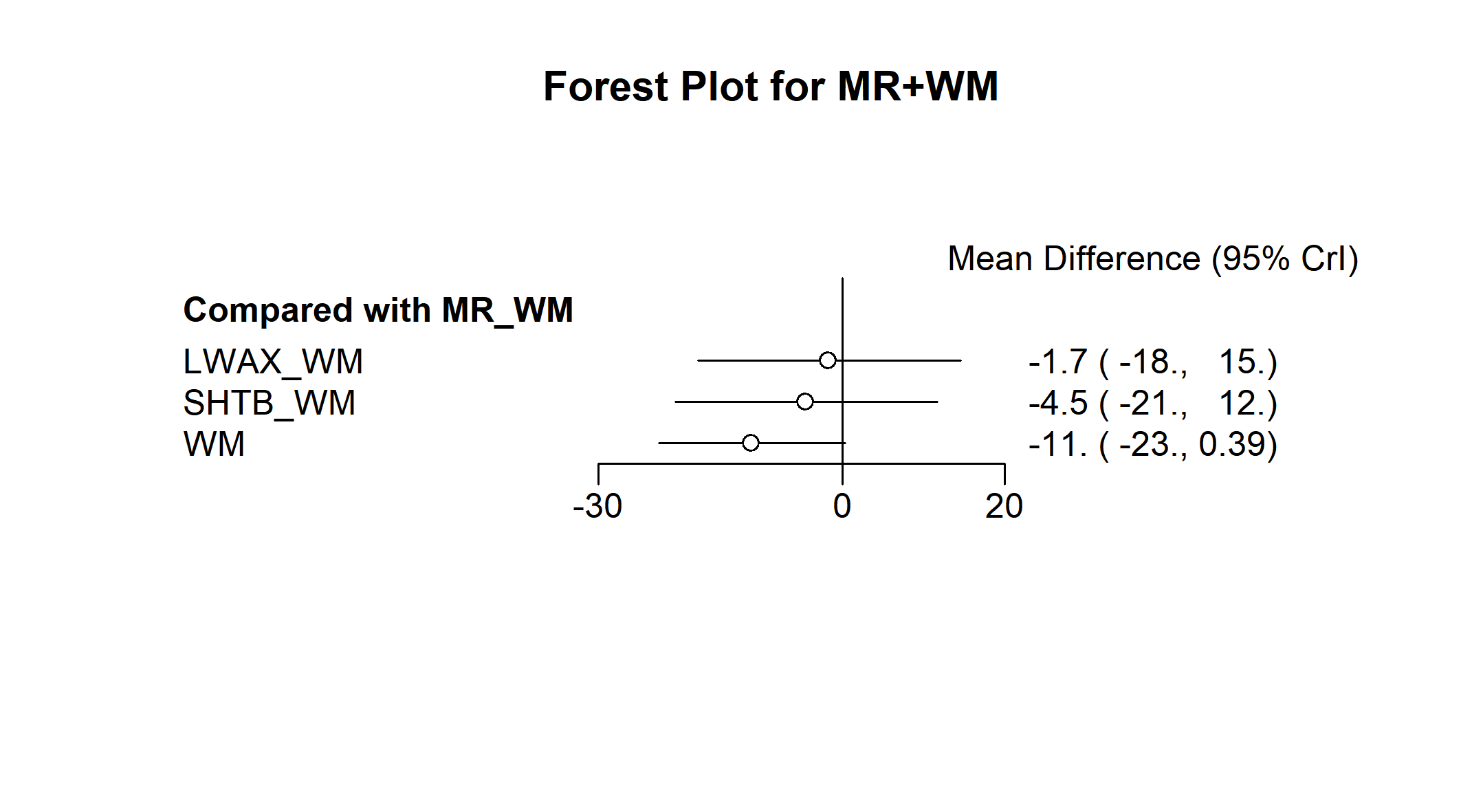

Supplement: Supplementary file 1 [file Data_Sheet_1.zip › Supplementary_Material/Supplementary Figure/SP/SPforest_MR+WM.tiff]

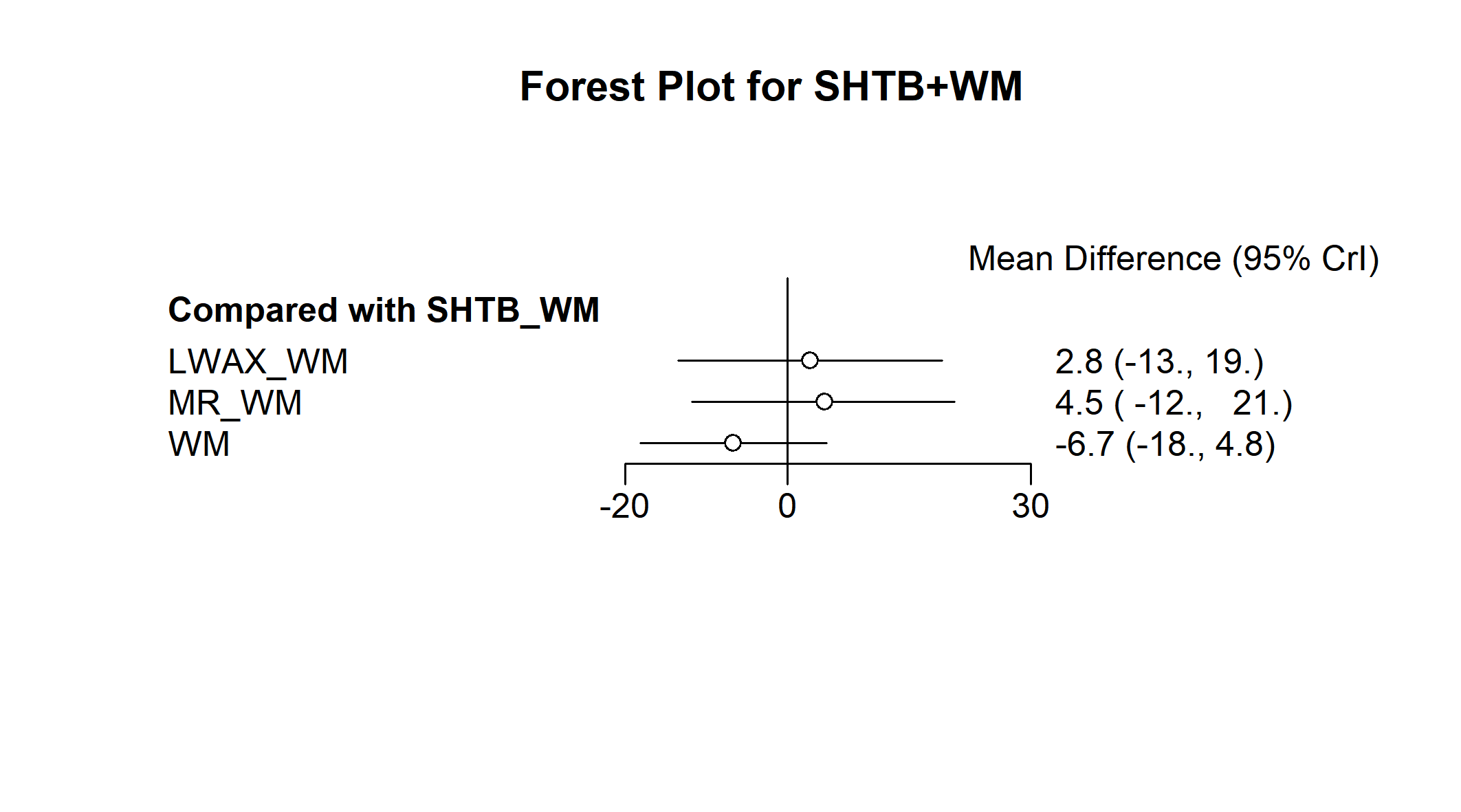

Supplement: Supplementary file 1 [file Data_Sheet_1.zip › Supplementary_Material/Supplementary Figure/SP/SPforest_SHTB+WM.tiff]

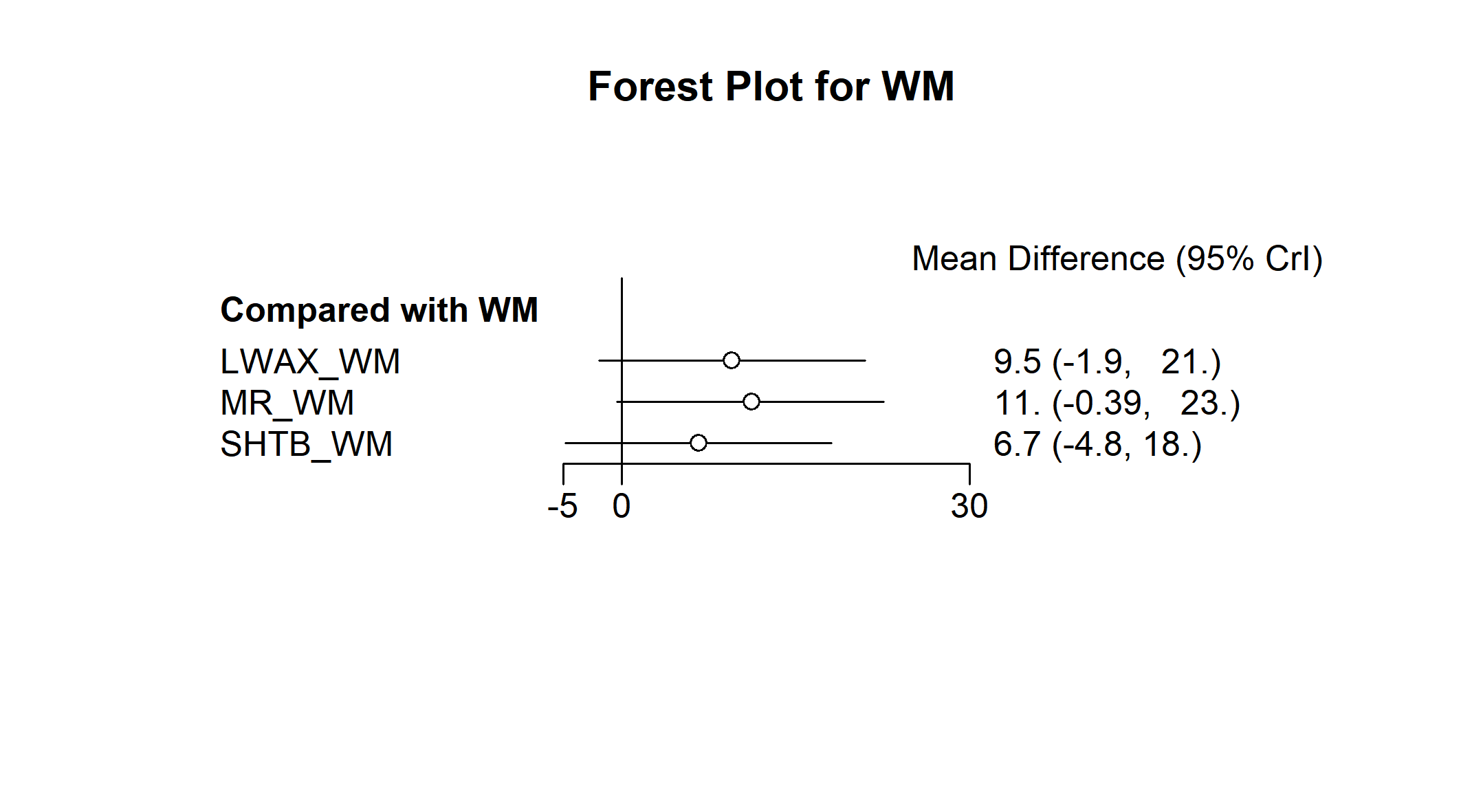

Supplement: Supplementary file 1 [file Data_Sheet_1.zip › Supplementary_Material/Supplementary Figure/SP/SPforest_WM.tiff]

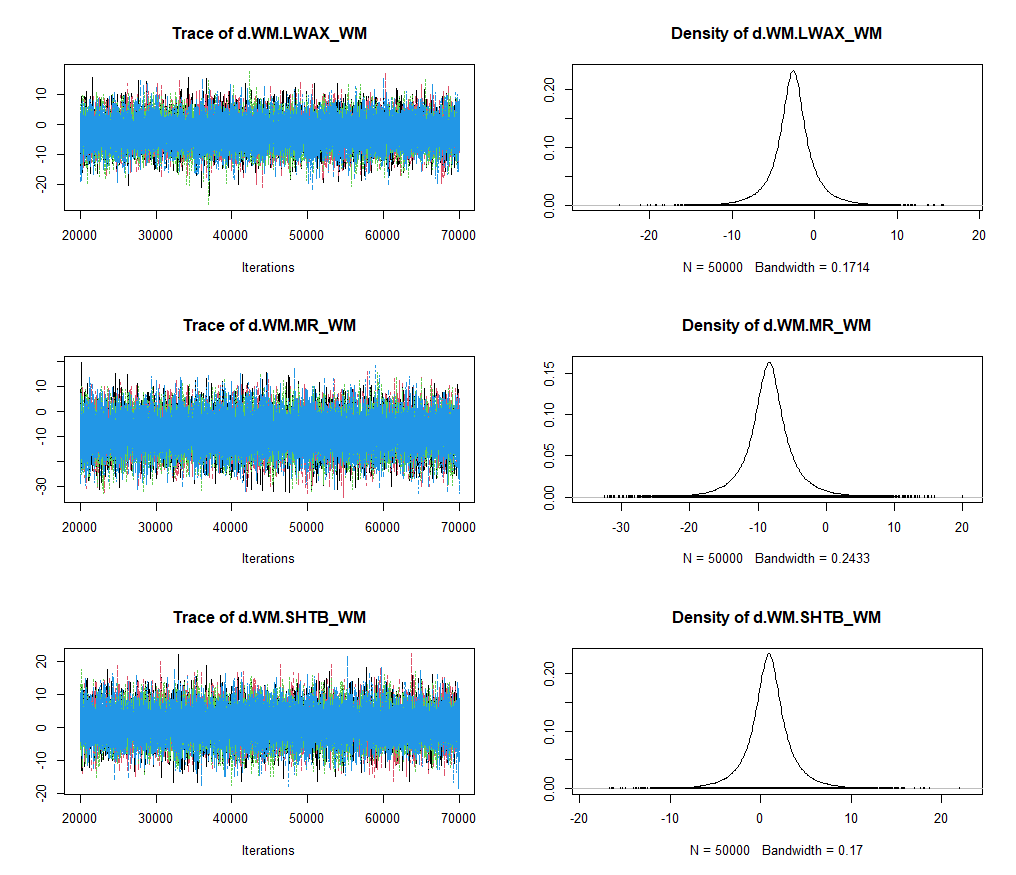

Supplement: Supplementary file 1 [file Data_Sheet_1.zip › Supplementary_Material/Supplementary Figure/stool consistency score/Figure 1.tiff]

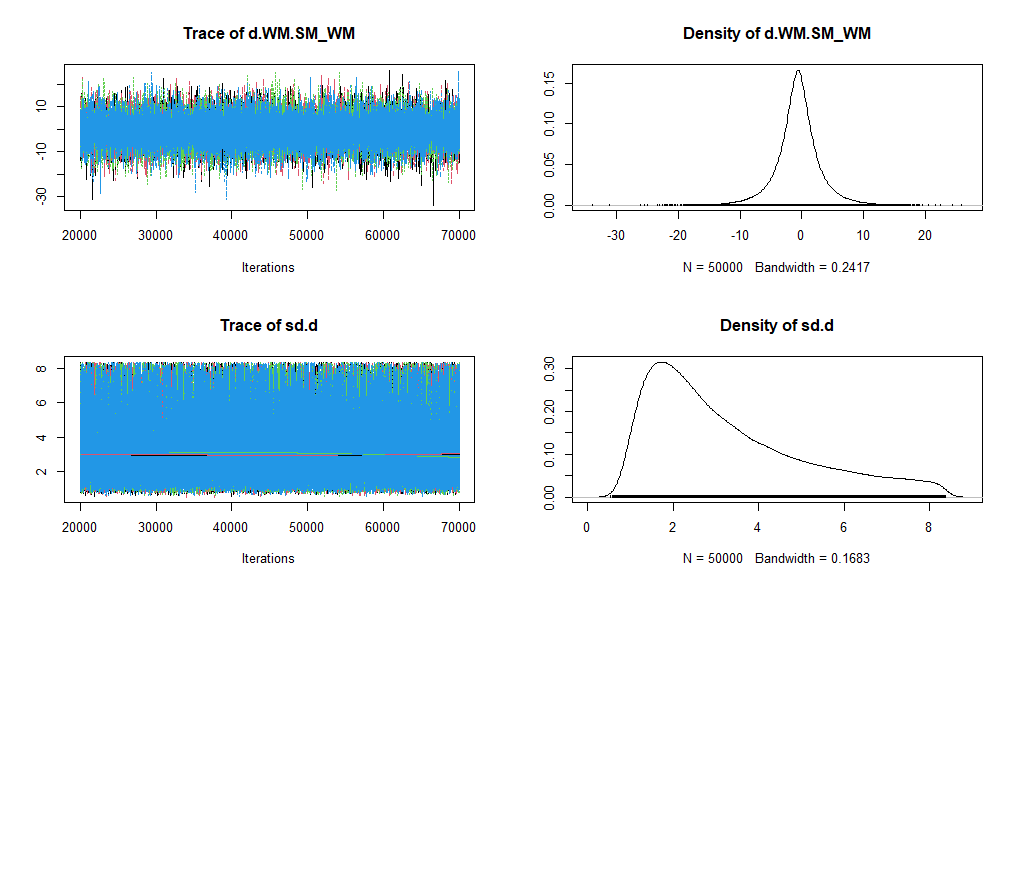

Supplement: Supplementary file 1 [file Data_Sheet_1.zip › Supplementary_Material/Supplementary Figure/stool consistency score/Figure 2.tiff]

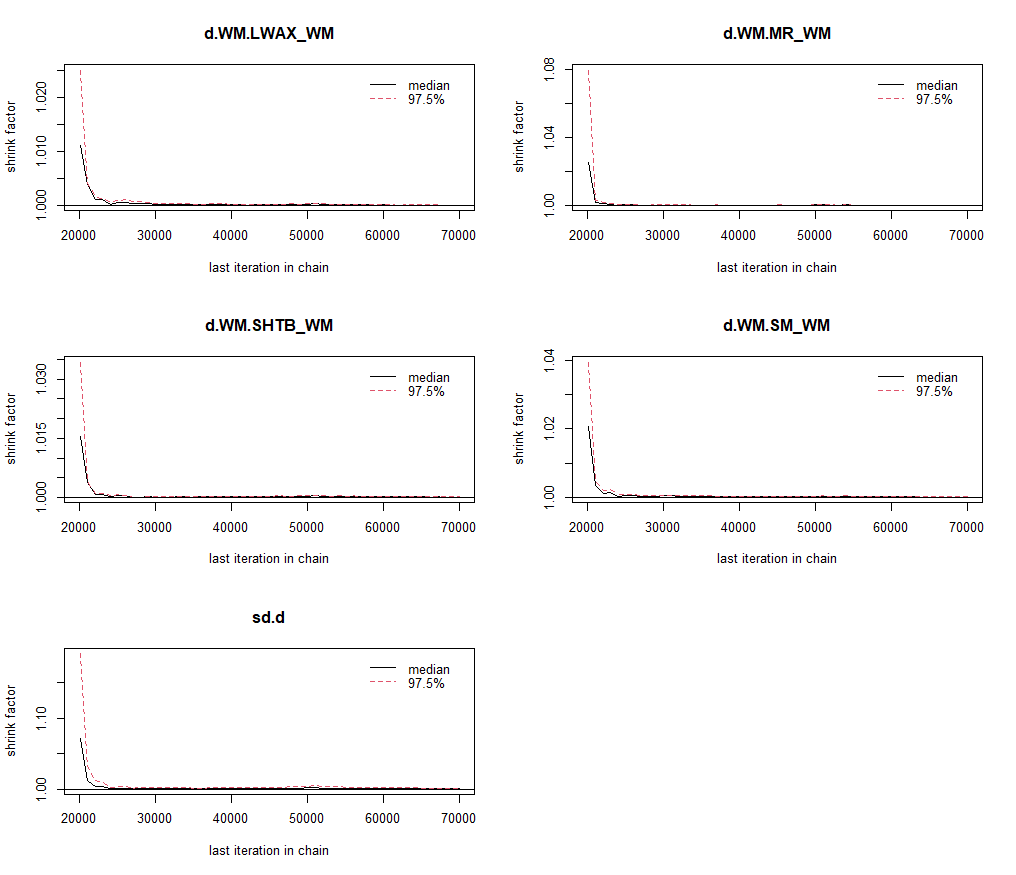

Supplement: Supplementary file 1 [file Data_Sheet_1.zip › Supplementary_Material/Supplementary Figure/stool consistency score/Figure 3.tiff]

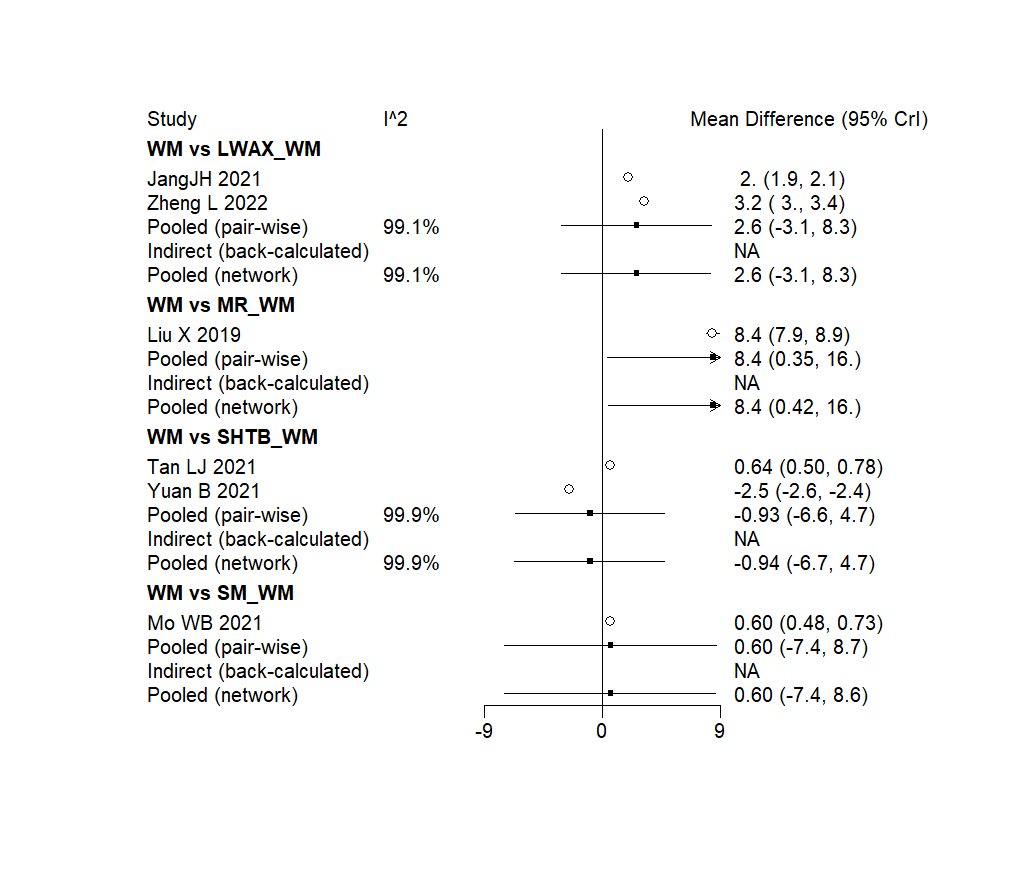

Supplement: Supplementary file 1 [file Data_Sheet_1.zip › Supplementary_Material/Supplementary Figure/stool consistency score/Figure 4.tiff]

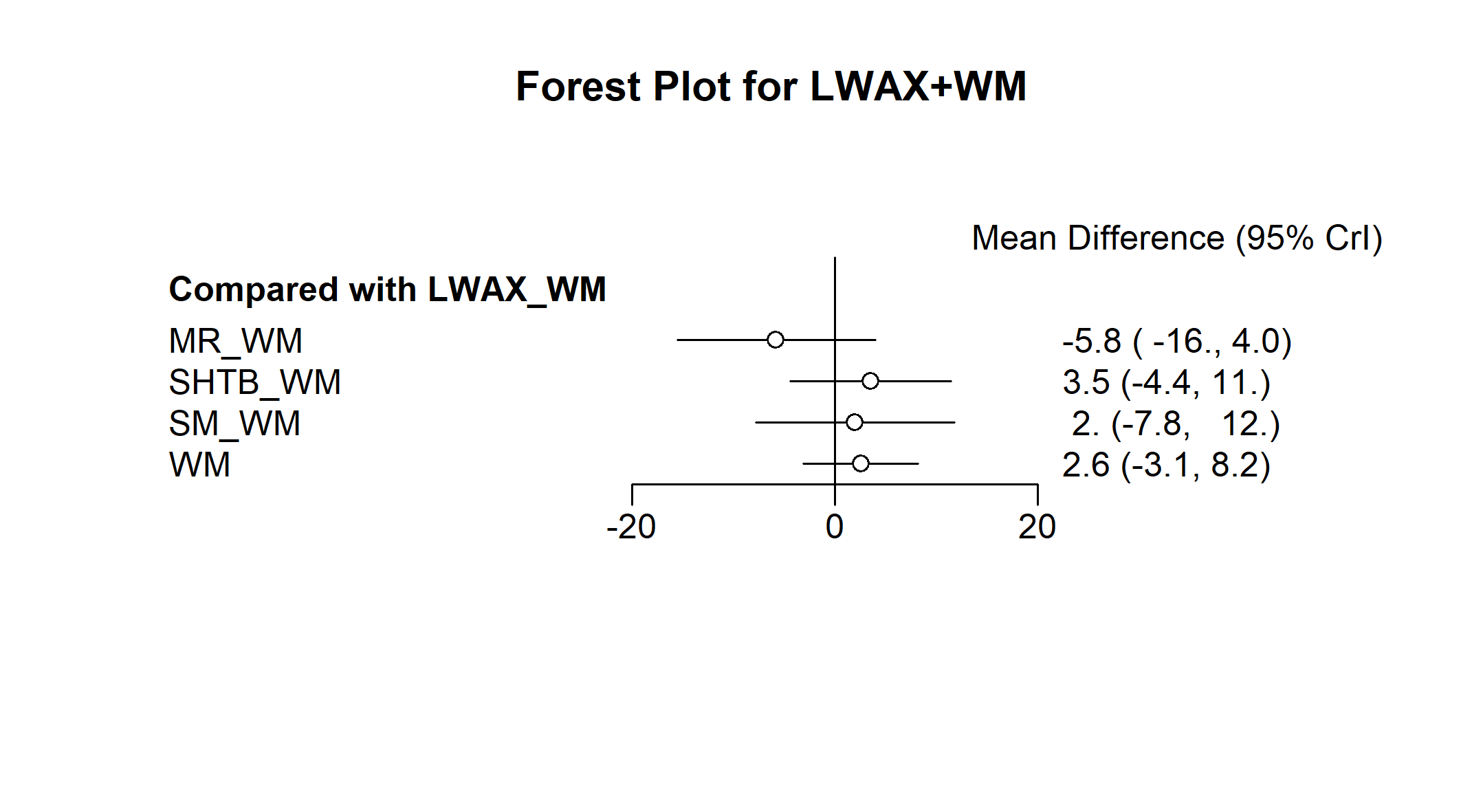

Supplement: Supplementary file 1 [file Data_Sheet_1.zip › Supplementary_Material/Supplementary Figure/stool consistency score/Figure 5.tiff]

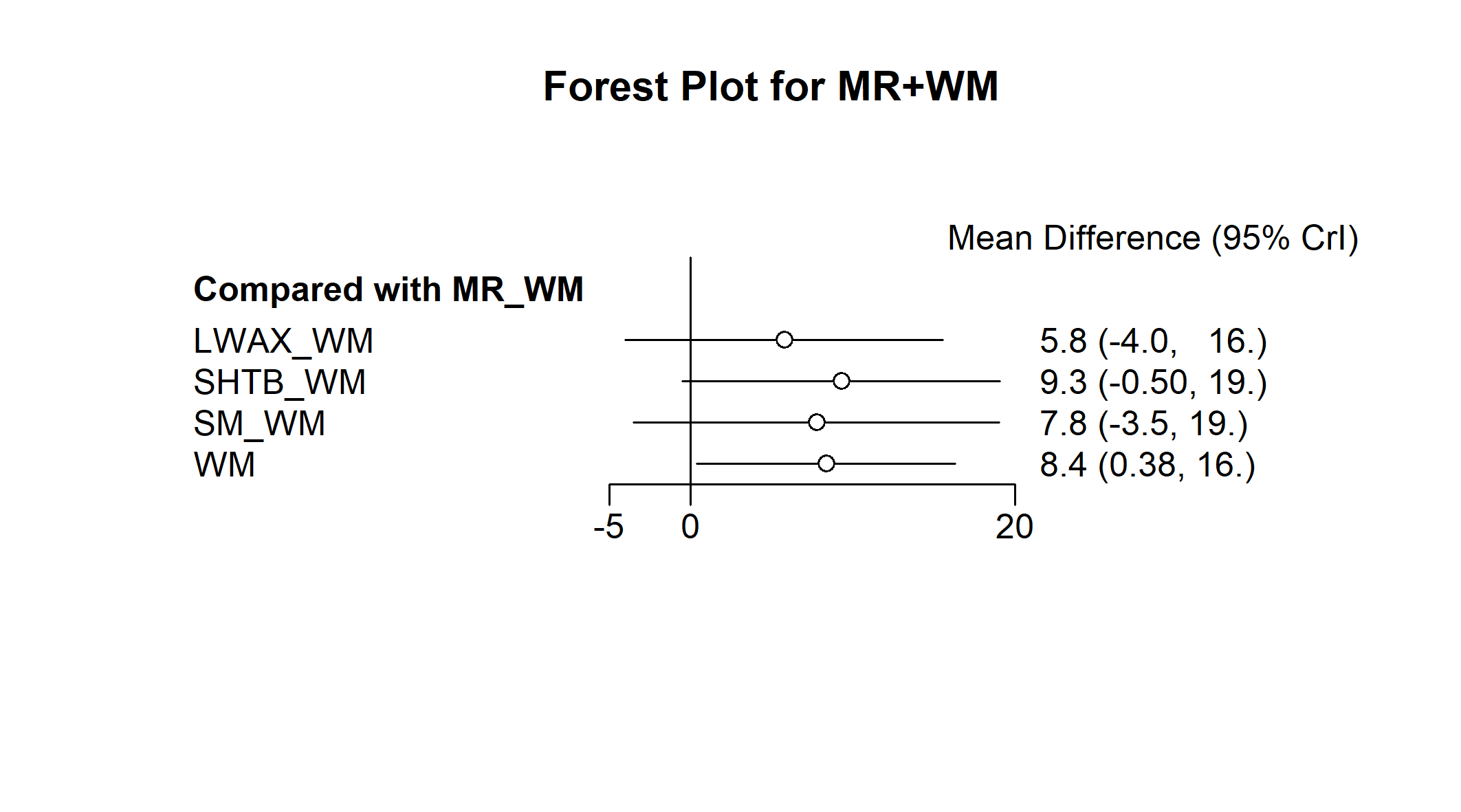

Supplement: Supplementary file 1 [file Data_Sheet_1.zip › Supplementary_Material/Supplementary Figure/stool consistency score/Figure 6.tiff]

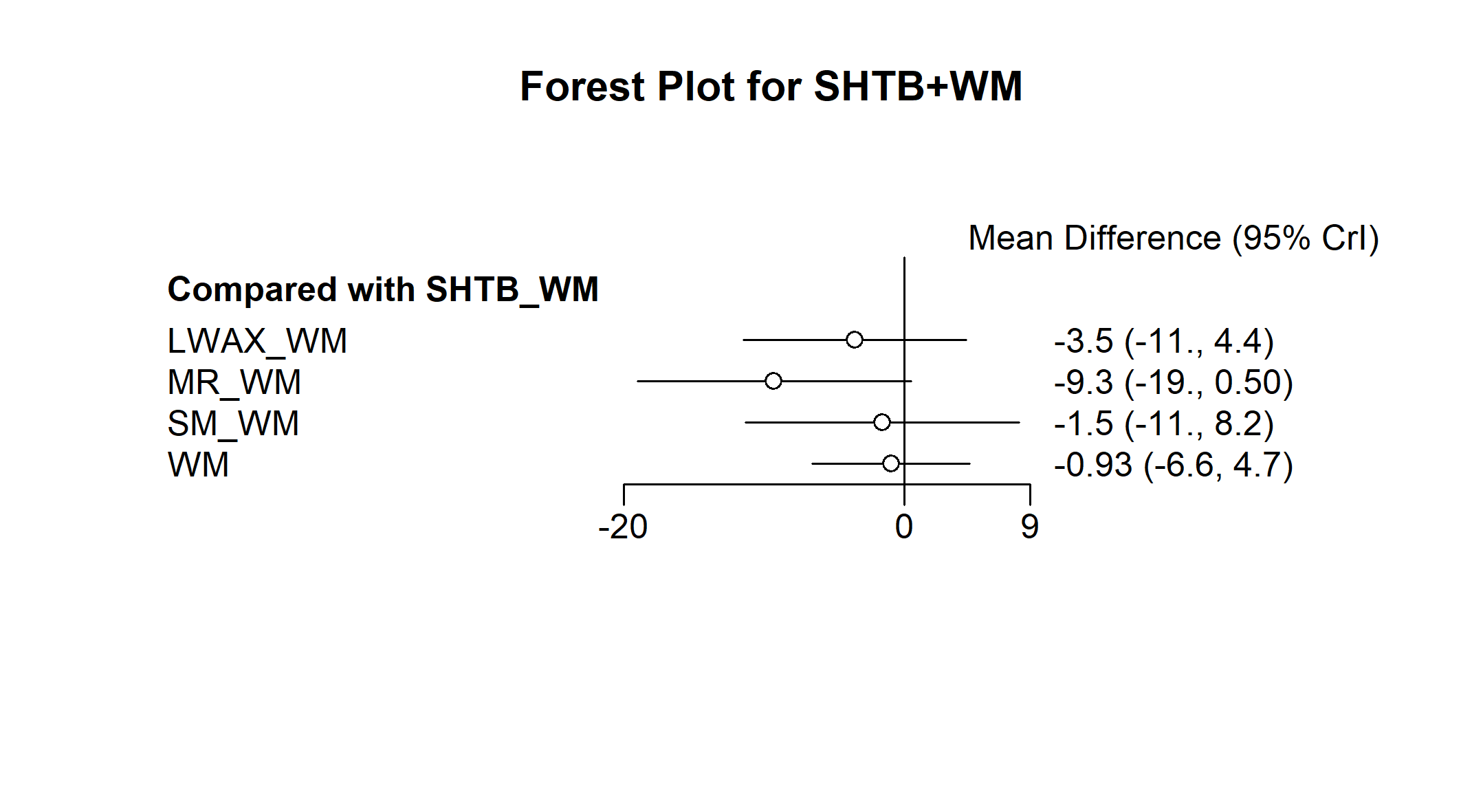

Supplement: Supplementary file 1 [file Data_Sheet_1.zip › Supplementary_Material/Supplementary Figure/stool consistency score/Figure 7.tiff]

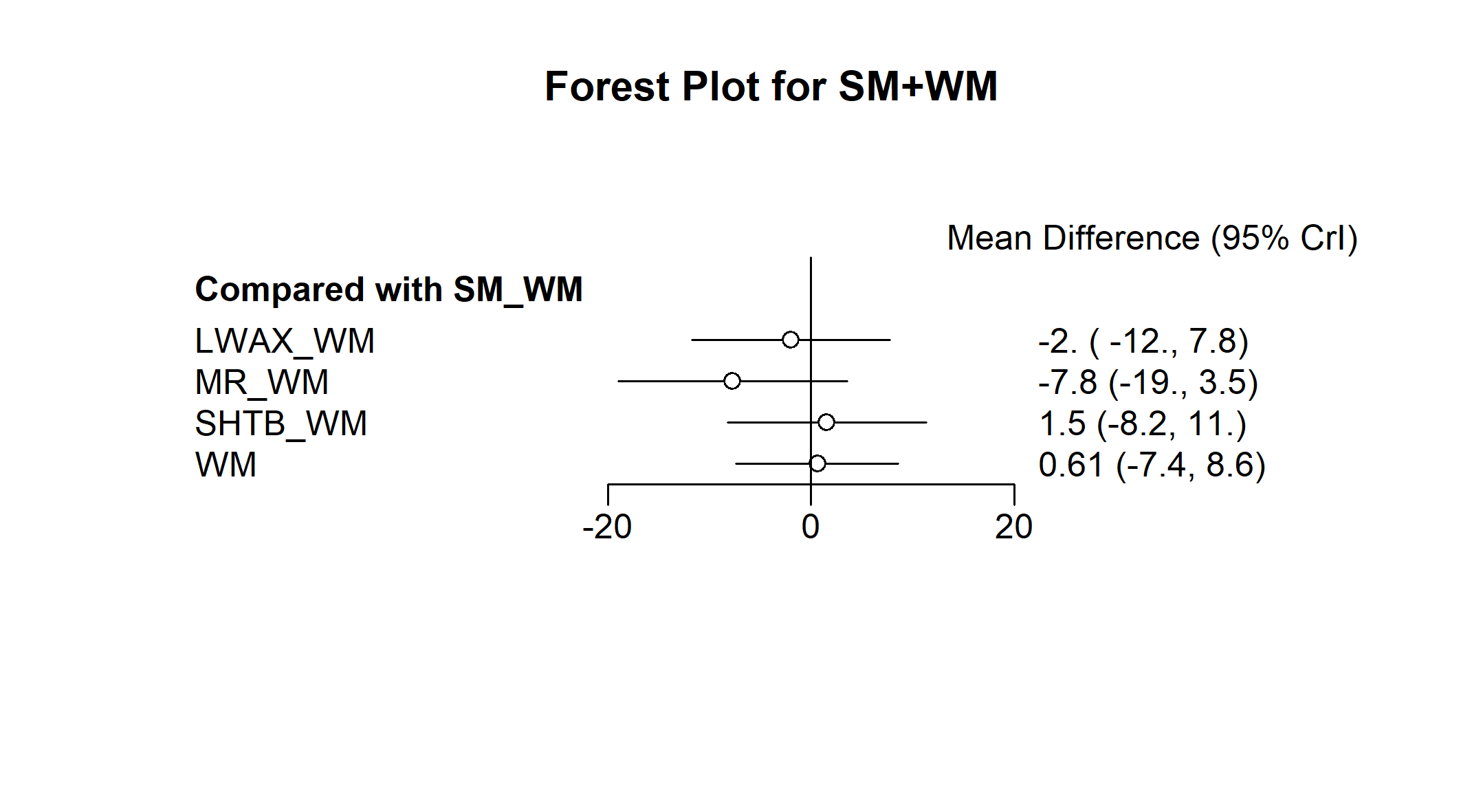

Supplement: Supplementary file 1 [file Data_Sheet_1.zip › Supplementary_Material/Supplementary Figure/stool consistency score/Figure 8.tiff]

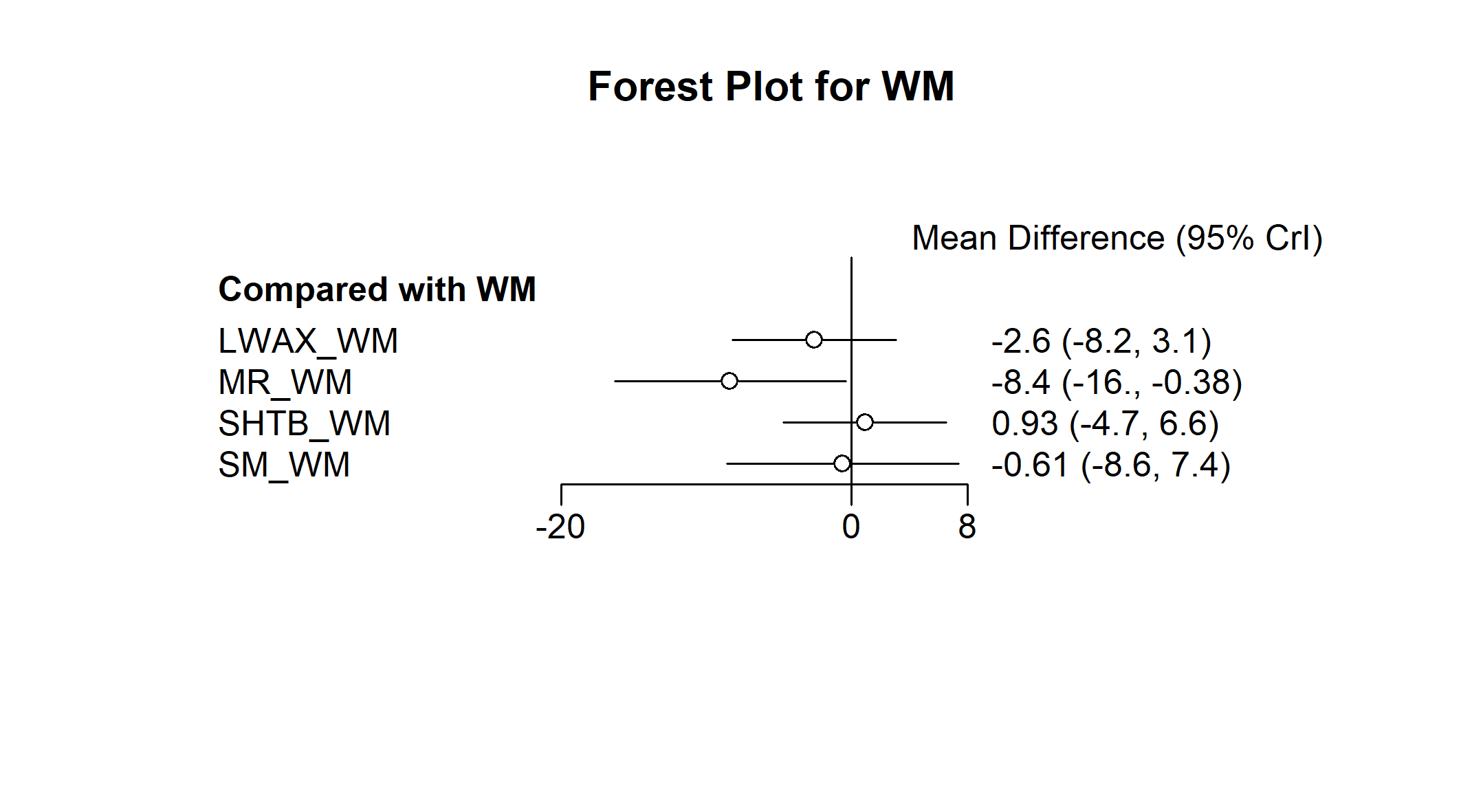

Supplement: Supplementary file 1 [file Data_Sheet_1.zip › Supplementary_Material/Supplementary Figure/stool consistency score/Figure 9.tiff]
